# CC-PROMISE effectively integrates two forms of molecular data with multiple biologically related endpoints

**DOI:** 10.1186/s12859-016-1217-0

**Published:** 2016-10-06

**Authors:** Xueyuan Cao, Kristine R. Crews, James Downing, Jatinder Lamba, Stanley B. Pounds

**Affiliations:** 1 Department of Biostatistics, St. Jude Children’s Research Hospital, 262 Danny Thomas Place, Memphis, 38105 USA; 2 Department of Pharmaceutical Sciences, St. Jude Children’s Research Hospital, 262 Danny Thomas Place, Memphis, 38105 USA; 3 Department of Pathology, St. Jude Children’s Research Hospital, 262 Danny Thomas Place, Memphis, 38105 USA; 4 Department of Pharmacotherapy and Translational Research, University of Florida, 1333 Center Drive, Gainesville, 32610 USA

**Keywords:** Integrated data analysis, Microarray, Sequencing, Projection onto the most interesting statistical evidence, Canonical correlation

## Abstract

**Background:**

As new technologies allow investigators to collect multiple forms of molecular data (genomic, epigenomic, transcriptomic, etc) and multiple endpoints on a clinical trial cohort, it will become necessary to effectively integrate all these data in a way that reliably identifies biologically important genes.

**Methods:**

We introduce CC-PROMISE as an integrated data analysis method that combines components of canonical correlation (CC) and projection onto the most interesting evidence (PROMISE). For each gene, CC-PROMISE first uses CC to compute scores that represent the association of two forms of molecular data with each other. Next, these scores are substituted into PROMISE to evaluate the statistical evidence that the molecular data show a biologically meaningful relationship with the endpoints.

**Results:**

CC-PROMISE shows outstanding performance in simulation studies and an example application involving pediatric leukemia. In simulation studies, CC-PROMISE controls the type I error (misleading significance) rate very near the nominal level across 100 distinct null settings in which no molecular-endpoint association exists. Also, CC-PROMISE has better statistical power than three other methods that control type I error in 396 of 400 (99 %) alternative settings for which a molecular-endpoint association is present; the power advantage of CC-PROMISE exceeds 30 % in 127 of the 400 (32 %) alternative settings. These advantages of CC-PROMISE are also observed in an example application.

**Conclusion:**

CC-PROMISE very effectively identifies genes for which some form of molecular data shows a biologically meaningful association with multiple related endpoints.

**Availability:**

The R package CCPROMISE is currently available from www.stjuderesearch.org/site/depts/biostats/software.

**Electronic supplementary material:**

The online version of this article (doi:10.1186/s12859-016-1217-0) contains supplementary material, which is available to authorized users.

## Background

The advance of microarray and sequencing technologies have empowered the scientific community to economically and rapidly collect multipe forms of molecular ‘omic’ data for large cohorts of patients. These molecular data have provided intriguing insights into the development of many human diseases. Integration of molecular data with clinical endpoints can also identify molecular features that associate with disease prognosis. These exciting possibilities continue to expand as researchers continue to collect more comprehensive molecular data on a larger number of research subjects for a growing number of diseases.

The exponential growth in data acquisition capacity presents many opportunities and challenges in data analysis and interpretation. Innovative methods have been developed to address several interpretational challenges such as how to discover and define disease subgroups [1–3], define statistical significance [4–6] compute statistical power for settings that involve thousands or even millions of variables. Computational methods have been developed to facilitate visualization and process data rapidly without overwhelming technical resources.

Genome-wide association studies (GWAS) have explored the association of one form of molecular data with one clinical endpoint of interest. Typically, a GWAS evaluates the association of each molecular feature with one endpoint of interest and then adjusts the results for multiple testing. GWAS studies and data analyses have yielded many intriguing biological insights as enumerated by the GWAS catalog (https://www.ebi.ac.uk/gwas/).

A natural extension of GWAS is to explore the association of one form of molecular data with multiple biologically related endpoints. One way to do this is to perform a GWAS analysis for each endpoint and then identify genes that appear on each endpoint’s list of most significant results. This list overlap approach can occasionally yield useful findings. However, in many applications, it is impossible to identify any particular gene without relaxing the significance threshold for the lists to the extent that statistical rigor is undermined.

Projection onto the most interesting statistical evidence (PROMISE) is an effective method to integrate one form of molecular data with multiple endpoints [7, 8]. For each molecular feature, PROMISE computes a composite association statistic and *p*-value that evaluates the association of that feature with each endpoint. In this way, PROMISE obtains one list of significant findings thereby avoiding the problem of non-overlapping lists and improving statistical power. PROMISE has been used successfully to evaluate the association of multiple endpoints measuring therapeutic efficacy with SNP genotypes [8] or gene expression [7] in pediatric leukemia.

Most recently, it has become commonplace to collect multiple forms of molecular data (genotype, copy number, methylation, mRNA expression, miRNA expression, etc) and multiple endpoints for a cohort of patients. The Cancer Genome Atlas (cancergenome.nih.gov) and The Pediatric Cancer Genome Project (www.pediatriccancergenomeproject.org) are examples of research projects with multiple forms of molecular data on a common set of subjects. These data present the opportunity to better understand the associations among molecular data and the associations of the molecular data with the endpoints. Canonical correlation (CC) is a classical method used to evaluate the association of two multivariate data sets with one another [9]. CC computes the maximally correlated pair of linear combinations of the two data sets, the correlation of those linear combinations, and a *p*-value for that correlation statistic. The linear combinations define a pair of score values for each patient that represent each molecular data set. Recent research has developed versions of CC that impose sparsity to enhance interpretability of results [10].

To gain the most accurate understanding from these data, there is currently a need to develop methods that effectively perform an integrated analysis of multiple forms of molecular data with multiple endpoints. PROMISE effectively integrates one form of molecular data with multiple endpoints; CC effectively integrates two forms of molecular data with one another. Here, we introduce CC-PROMISE as a method that combines CC and PROMISE to effectively integrate two forms of molecular data with multiple endpoints.

## Methods

The CC-PROMISE method may be used to integrate any two forms of quantitative high-dimensional molecular data with multiple endpoints of diverse data types (quantitative, qualitative, censored time-to-event, etc). Here, we present the method in terms of integrating methylation and RNA expression data as a concrete example. The CC-PROMISE method may be used to integrate other forms of data, such as miRNA and mRNA expression data.

### Setting and notation for data

Suppose methylation and gene expression data have been collected for each of *i*=1,…,*n* subjects. Let *g*=1,…,*G* index the genes for which methylation and expression data are available. For each gene *g*, let *l*_*g*_=1,…,*L*_*g*_ index the loci of markers for which methylation data are collected. Note that the subscript *g* of *l*_*g*_ and *L*_*g*_ is clear by context. Thus, the subscript *g* will be omitted from *l*_*g*_ and *L*_*g*_ for simplicity of notation. Let *m*_*gli*_ represent the methylation of locus *l* of gene *g* for subject *i*. Also, let *f*_*g*_=1,…,*F*_*g*_ index the features of gene *g* for which expression data are available. The subscript *g* of *f*_*g*_ and *F*_*g*_ will be omitted for simplicity of notation. Let *x*_*gfi*_ represent the expression of feature *f* of gene *g* for subject *i*. Also, suppose that we have collected data on endpoints *k*=1,…,*K* for each subject. Let *y*_*ki*_ represent the value of endpoint *k* for subject *i*. A glossary of the mathematical notation is available in the Additional file 1.

### Associate each methylation marker with expression feature

For each gene *g*, it is often interesting to explore the association of each methylation marker with each expression feature. For each gene *g*, let *r*_*gfl*_ represent the observed sample correlation and *ρ*_*gfl*_ represent the true population correlation of the expression *x*_*gf*1_,*x*_*gf*2_,…,*x*_*gfn*_ of feature *f* with the methylation *m*_*gl*1_,*m*_*gl*2_,…,*m*_*gln*_ of locus *l*. Also, let *p*_*gfl*_ be the *p*-value testing the null hypothesis *H*_0_:*ρ*_*gfl*_=0 that the true correlation *ρ*_*gfl*_ is zero.

### Associate each endpoint with each expression feature

For each gene *g*, it is also interesting to explore the association of each expression feature with each endpoint. Thus, for each endpoint *k* and each expression feature *f* of gene *g*, compute a statistic *a*_*kgf*_ that measures the association of the expression *x*_*gf*1_,*x*_*gf*2_,…,*x*_*gfn*_ with the endpoint *y*_*k*1_,*y*_*k*2_,…,*y*_*kn*_. Well-established methods may be used to compute the association statistic. For example, assuming that the expression data are continuous quantitative values, one may use Spearman’s correlation to measure association of expression with a continuous quantitative endpoint, Kendall’s *τ* to measure association of expression with an ordinal endpoint, ANOVA may be used to measure association with a categorical endpoint, and Cox regression modeling may be used to measure association with a censored time-to-event endpoint. We typically use rank-based statistics for endpoint associations due to their well-established robustness against outliers and other forms of noise in the data. We also use rank-based statistics in the example application below. Nevertheless, our framework allows for other methods to be utilized as appropriate for specific applications. The statistical significance (*p*-value) may be computed using those classical methods or via a permutation algorithm described in subsection “Compute permutation *p*-values”. It is important that the association statistics be represented on a common scale for many of the subsequent analyses described below.

### Associate each endpoint with each methylation marker

For each gene *g*, the association of each endpoint with each methylation marker is performed in a very similar manner as described immediately above. For each endpoint *k* and each methylation marker *l* of gene *g*, compute a statistic *a*_*kgl*_ that measures the association of the methylation *m*_*gl*1_,*m*_*gl*2_,…,*m*_*gln*_ with the endpoint *y*_*k*1_,*y*_*k*2_,…,*y*_*kn*_. Again, classical methods may be used here and all association statistics should be represented on a similar scale.

### Define the most interesting statistical evidence

Association statistics can be represented on a correlation-like scale such that values of -1, 0, and +1 respectively indicate a negative deterministic relationship, no association, and a positive deterministic relationship between two variables. On this scale, values of ±1 clearly indicate deterministic associations that are typically of greatest biological interest. Thus, the values ±1 may be considered the *most interesting statistical evidence* for any particular statistic that measures association on a correlation-like scale. Subsequently, the result *a*_*kfg*_=±1 on a correlation-like scale is the most interesting statistical evidence for the association of each endpoint *k* with the expression of feature *f* of gene *g*.

In many applications, biological and mathematical reasoning may be used to define the most interesting statistical evidence for the vector ***a***_*fg*_={*a*_1*fg*_,*a*_2*fg*_,…,*a*_*Kfg*_} of statistics that measure the association of each endpoint *k*=1,…,*K* with the expression of feature *f* of gene *g*. As described above, *a*_*kgf*_=±1 is the most interesting statistical evidence for each endpoint *k*. Therefore, the most interesting statistical evidence for ***a***_*fg*_ must be the set of 2^*K*^ vectors of length *K* with entries ±1. By symmetry, the constraint *a*_1*gf*_=1 is imposed to reduce consideration to a subset of 2^*K*−1^ vectors. Now, suppose that prior knowledge about the endpoints indicates that only one of the remaining 2^*K*−1^ vectors is biologically interesting or plausible. For example, in the application of subsection “Acute myeloid leukemia example”, all three endpoints measure sensitivity of leukemia cells to the chemotherapeutic agent cytarabine. Thus, the most interesting statistical evidence for that application is observing that expression of feature *f* of gene *g* has a deterministic positive (or negative) association with drug sensitivity. With these biological and mathematical considerations, we let ***λ***={*λ*_1_,*λ*_2_,…,*λ*_*k*_} represent the most interesting statistical evidence for the vector ***a***_*fg*_ for each feature *f* of each gene *g*.

Analogous logic indicates that either +***λ*** or −***λ*** is the most interesting statistical evidence for the vector of statistics ***a***_*lg*_={*a*_1*fg*_,*a*_2*fg*_,…,*a*_*Kfg*_} that measure association of methylation with the endpoints. Again, insisting on biological plausibility imposes a constraint on the sign of ***λ***. In particular, the findings are plausible only if the methylation-expression association, methylation-endpoint associations, and expression-endpoint associations are concordant. Thus, sign(*r*_*gfl*_)***λ*** is the most interesting statistical evidence for the vector ***a***_*lg*_ that measures the association of each endpoint *k* with the methylation at each locus *l* of gene *g*.

### Associate all endpoints with each expression feature

To explore the association of each expression feature *f* of each *g* with all endpoints, we compute the *projection onto the most interesting evidence* (PROMISE) statistic as

1 $$ t_{gf} = \sum\limits_{k=1}^{K} \lambda_{k} a_{kgf}.  $$

The magnitude of the PROMISE statistic *t*_*gf*_ measures the evidence indicating that the associations with the individual endpoints align with predefined most interesting statistical evidence. The sign of the PROMISE statistic indicates the direction of the vector of the associations relative to that of the most interesting statistical evidence. Overall, the PROMISE statistic measures the discrepancy between the observed associations and the global null (all associations are zero) along the direction of the most interesting statistical evidence. The statistical significance of the PROMISE statistic is determined by computing a permutation *p*-value as described in subsection “Compute permutation *p*-values”.

### Associate all endpoints with each methylation marker

Similarly, to explore the association of methylation marker *l* of each gene *g* with all endpoints, we compute the PROMISE statistic

2 $$ t_{gl} = \sum\limits_{k=1}^{K} \lambda_{k} a_{kgl}  $$

with an analogous interpretation. Likewise, significance is determined by computing a permutation *p*-value as described in subsection “Compute permutation *p*-values”.

### Associate all endpoints with each pair of one methylation marker and one expression feature

Next, to explore the association of all endpoints with each pair (*l*,*f*) of a methylation marker *l* and expression feature *f* of each gene *g*, we compute the combined PROMISE statistic

3 $$ t^{\star}_{glf} = t_{gf}+\text{sign}(r_{glf})t_{gl}.  $$

**Fig. 1:**
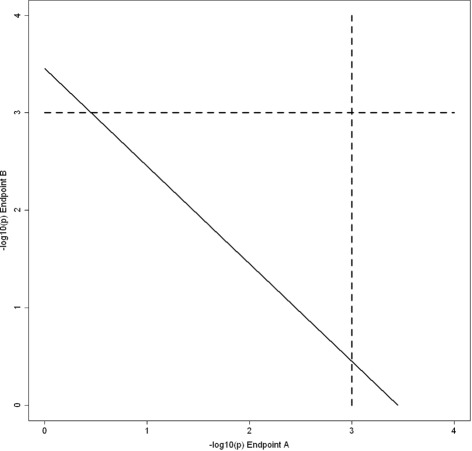
Rejection Regions of PROMISE and List Overlap Methods. The figure illustrates the rejection regions defined by PROMISE and list overlap methods for association of one genomic variable with two endpoint variables. The horizontal axis shows −log_10_(*p*) for association of the genomic variable with one endpoint (*Endpoint A*) and the vertical axis shows −log_10_(*p*) for association of the genomic variable with the other endpoint (*Endpoint B*). The dashed lines at 3 indicate the significance thresholds for −log_10_(*p*) obtained by using *p*=0.001 as the threshold to declare significance for association of one genomic variable with one endpoint. Thus, the list overlap method will only identify genomic variables with −log_10_(*p*) in the top right corner. In contrast, PROMISE will identify genomic variables above and to the right of a diagonal line as significant. The position of the PROMISE threshold line will vary from application to application, but will usually encapsulate the overlap region

This statistic measures the discrepancy between the observed association statistics and the global null (all associations are zero) along the vector defining the most interesting statistical evidence. The sign measures direction in terms of the most interesting statistical evidence and the magnitude measures cumulative weight of the evidence against the global null. Statistical significance is determined by computing permutation *p*-values as described in subsection “Compute permutation *p*-values”. Here, we choose an additive formula to define (3) for simplicity of calculation and interpretation in terms of the rejections regions depicted in Fig. 1. Future research may find that other mathematical definitions of a combined statistic have better performance than the additive formula in some applications.

### Gene-level analyses

Subsections “Associate each methylation marker with expression feature – Associate all endpoints with each pair of one methylation marker and one expression feature” describe analyses performed at the level of individual expression features and individual methylation markers. To perform a gene-level analysis for each gene *g*, we first perform canonical correlation analysis (CCA) on the matrix *M*_*g*_ of the methylation at all loci *l*_*g*_=1,…,*L*_*g*_ and the matrix *X*_*g*_ of the expression of each feature *f*_*g*_=1,…,*F*_*g*_. CCA computes the canonical correlation coefficient $\tilde {r}_{g}$ and formally tests the null hypothesis that the canonical correlation is zero. In this way, CCA performs a gene-level analysis that is analogous to the simple feature-level correlation analysis of subsection “Associate each methylation marker with expression feature”.

CCA also computes a summary score for expression and a summary score for methylation that may be used to perform gene-level analyses analogous to those described in subsections “Associate each endpoint with each expression feature – Associate all endpoints with each pair of one methylation marker and one expression feature”. Given the matrix *X*_*g*_ of expression values for each subject *i*=1,…,*n* and each expression feature *f*_*g*_=1,…,*F*_*g*_ and the matrix *M*_*g*_ of methylation values for each subject *i*=1,…,*n* and each methylation marker *l*_*g*_=1,…,*L*_*g*_, CCA determines the linear combinations of the columns of the matrices that are maximally correlated. As a result, CCA obtains the expression matrix linear combination value $\tilde {x}_{gi}$ and the methylation matrix linear combination value $\tilde {m}_{gi}$ for each subject *i*=1,…,*n*. These linear combination *scores* are variables that can be evaluated using the methods of subsections “Associate each endpoint with each expression feature – Associate all endpoints with each pair of one methylation marker and one expression feature Associate all endpoints with each pair of one methylation marker and one expression feature”. In particular, these analyses can be performed by substituting the expression score values $\tilde {x}_{gi}$ for the individual feature expression values *x*_*gfi*_ into the framework of subsections “Associate each endpoint with each expression feature and Associate all endpoints with each expression feature”, substituting the methylation score values $\tilde {m}_{gi}$ for the individual marker methylation values *m*_*gli*_ into the framework of subsections “Associate each endpoint with each methylation marker and Associate all endpoints with each methylation marker”, and finally substituting the canonical correlation $\tilde {r}_{g}$ for the simple correlation *r*_*gfl*_ into the framework of subsection “Associate all endpoints with each pair of one methylation marker and one expression feature Associate all endpoints with each pair of one methylation marker and one expression feature”.

### Compute permutation *p*-values

The statistical significance of the PROMISE statistic is determined by a permutation procedure. The assignment of endpoint data to the molecular data is permuted and the test statistic recomputed many times. The *p*-value is given by the proportion of permutation repetitions that yield a PROMISE statistic with magnitude greater than or equal to that of the observed PROMISE statistic. An adaptive permutation procedure [8] is used to reduce computing time without compromising the statistical rigor of the results. Briefly, let *t*_0_ be the value of the observed PROMISE statistic, let *b* index permutation repetitions, and let *t*_*b*_ be the PROMISE statistic observed from permutation *b*. (In this section, other subscripts are omitted for simplicity of notation because the same permutation procedure may be used to compute permutation *p*-values for any of the PROMISE statistics defined above.) In each permutation *b*, the adaptive permutation procedure notes whether |*t*_*b*_|≥|*t*_0_| or |*t*_*b*_|<|*t*_0_|. The procedure continues until *B*_0_ permutations obtain |*t*_*b*_|≥|*t*_0_| or a total of *B*_1_ permutations are performed. This allows the permutation procedure to terminate early for genes that clearly are not statistically significant. For example, if *B*_0_=100 of the first 200 permutations obtain |*t*_*b*_|≥|*t*_0_|, the procedure stops to report a *p*-value of $\frac {100}{200} = 0.50$ instead of continuing for 10,000 permutations to report a blatantly insignificant *p*-value to four decimal places. In applications that involve exploring the association of many genes with the endpoints, the adaptive permutation procedure can reduce computing time by 99 % because typically the vast majority of genes do not have a strong association with the endpoint. The user may select the minimum number of permutations *B*_0_ and *B*_1_ to obtain the desired computational efficiency and statistical rigor as described by [8]. We use *B*_0_=100 and *B*_1_=10,000 in the simulations and application described below.

### Conceptual comparison of promise with list-overlap approaches

A very widely used method for integrated data analysis simply identifies genes that appear on multiple lists of the most significant hits from different data analyses. In other words, the analysis identifies the overlap across multiple lists of the most significant genes. This type of *list overlap* approach is popular because it is simple and thus can be used in a very broad spectrum of applications. It has been used with success in several applications.

However, list overlap approaches have several statistical and practical limitations. Each list includes a set of genes that exceed an arbitrary threshold for a test statistic or *p*-value. It can be unclear what statistical properties (false positive and false negative rates) are obtained for various thresholds for those lists. In many cases, there may be no overlap across lists even when each list has a very liberal threshold that allows for a large false positive rate. Additionally, the genes will appear in a different order on each list which often makes it unclear how to derive a final ranking of the genes by strength of empirical evidence.

The PROMISE method overcomes these limitations and brings many additional advantages over list overlap approaches. The PROMISE method provides one comprehensive *p*-value for each gene or feature tested. In this way, the genes are ranked by a common criterion with a clear statistical interpretation in terms of a false discovery rate. Also, with one PROMISE *p*-value per gene, the problem of finding no genes occurs only when no gene meets the chosen significance threshold for the PROMISE *p*-value. Furthermore, previously described [7, 8] and illustrated in Fig. 1, PROMISE provides better statistical power to identify genes with effects on multiple endpoints than do list overlap approaches. Finally, as shown in the simulation studies below, the CC-PROMISE provides similar benefits in the integrated analysis of two forms of high-dimensional molecular data with multiple endpoints.

## Results

### Simulation studies

#### Data generation

We performed simulation studies to evaluate the statistical properties of CC-PROMISE, PROMISE, and list overlap approaches as methods for integrated analysis of two forms of molecular data and multiple endpoints. In our simulations, for each subject we generated data for *K*=2 endpoints and one gene with *M*=10 methylation markers and *F*=2 expression features. For each subject *i*, data was generated in the following way. (Note that the subscript *i* will be omitted for simplicity of notation because the same process is used for each subject *i*.) The methylation *m*_1_ of locus *l*=1 was generated from a standard normal distribution. For the subsequent loci *l*=2,…,10, the methylation values were generated from the autoregressive relationship *m*_*l*_=*β*_*m*_ *m*_*l*−1_+*e* where *e* is a completely independent standard normal variable. The expression values of the *j*=1,2 expression features were generated from the regression relationship *x*_*j*_=*β*_*x*_ *m*_*j*_+*e* where again *e* is an independent standard normal variable. The two endpoints are generated from a similar regression relationship *y*_*j*_=*β*_*y*_ *x*_*j*_+*e* for *j*=1,2. We simulated 1,000 independent data sets for each of the 500 settings defined by combinations of the parameter values *β*_*m*_=−0.5,−0.3,0,+0.3,+0.5, *β*_*x*_=−0.5,−0.3,0,+0.3,+0.5, *β*_*y*_=−0.5,−0.3,0,+0.3,+0.5, and sample size *n*=30,50,100,500. Note that *β*_*x*_≠0 implies that expression and methylation are associated; *β*_*y*_≠0 implies that expression and the endpoints are associated, and that methylation, expression; and the endpoints are all associated with one another when both *β*_*x*_≠0 and *β*_*y*_≠0.

#### Analysis methods

We applied several analysis methods to each simulated data set. We performed a *complete CC-PROMISE analysis* (CCPR), an *expression PROMISE* (XPR) analysis for expression-endpoint associations, a *methylation PROMISE* (MPR) analysis for methylation-endpoint associations, and the overlap of the methylation and expression PROMISE (OVPR) analysis. The CCPR analysis performed canonical correlation analysis to compute one methylation score and one expression score and used those scores to perform the joint PROMISE analysis described in subsection “Gene-level analyses”. The XPR and MPR analyses each performed a feature- or marker-level PROMISE analysis with or without Bonferroni adjustment. The unadjusted expression PROMISE (UXPR) analysis was based on the minimum of the two feature-level *p*-values from the XPR analysis. The adjusted expression PROMISE (AXPR) analysis was based on the minimum Bonferroni-adjusted *p*-value from the two tests. The unadjusted methylation PROMISE (UMPR) and adjusted methylation PROMISE (AMPR) analyses were analogously defined. Finally, we performed an adjusted overalp (AOV) analysis based on the maximum of the AMPR and AXPR *p*-values and an unadjusted overlap (UOV) analysis based on the maximum of the UMPR and UXPR analyses. We did not consider single-endpoint analyses in this simulation study because our previous work has shown that overlaps among single-endpoint analyses typically have much less power than PROMISE in settings with related endpoints [7]. The statistical reasons for this power difference are briefly described in subsection “Conceptual comparison of promise with list-overlap approaches” and in detail by [7].

#### Performance metrics

For each of the 500 settings, we record the proportion of the 1000 simulated data sets for which the gene is declared significant by each method. For the settings with *β*_*y*_=0, the optimal performance is indicated by declaring significance for 1 % of the simulated data sets (Type I error control). For other settings, better performance is indicated by declaring significance for a greater proportion of data sets (statistical power).

#### Simulation results

**Fig. 2:**
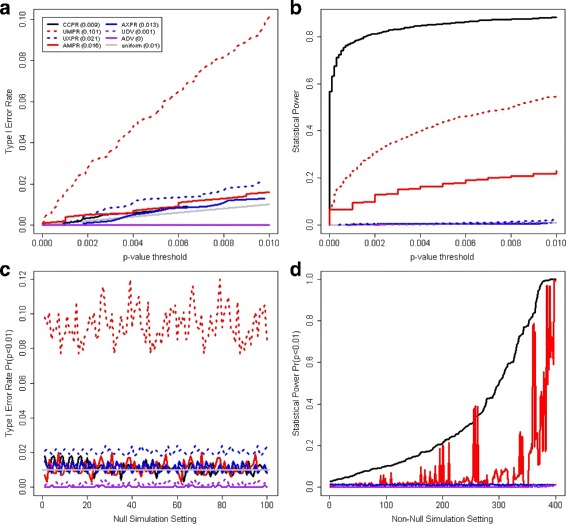
Simulation results. Panel **a** shows the probability of a significant *p*-value as a function of the *p*-value threshold of each method for the null setting *β*_*m*_=*β*_*x*_=*β*_*y*_=0 with *n*=100 subjects. The legend of line color and styles indicate results for the indicated methods and provide the result for the *p*-value threshold 0.01. The same line colors and styles are used in the other three panels. Panel **b** shows similar results for the non-null setting *β*_*m*_=*β*_*x*_=*β*_*y*_=0.5 and sample size *n*=100. Panel **c** shows the observed type I error rate for the methods with *p*-value threshold 0.01 across all 100 null settings with *β*_*y*_=0 indicating the endpoints have no association with the molecular data. Panel **d** shows the statistical power of the methods with adequate type I error rate control for the 400 non-null settings with *β*_*y*_≠0 indicating that the endpoints are associated with at least one element of the molecular data

CCPR clearly showed the best performance in the simulation studies (Fig. 2). Figure 2 a shows the results for the null setting with *β*_*m*_=*β*_*x*_=*β*_*y*_=0 (no associations among any variables) and *n*=100 subjects. The unadjusted analyses UMPR and UXPR have poor type I error control while the other methods have adequate type I error control. Figure 2 b shows the results for the non-null setting with *β*_*m*_=*β*_*x*_=*β*_*y*_=0.5 (strong associations among methylation, expression, and endpoints) and *n*=100 subjects. In this case, the power of CCPR greatly exceeds that of all other methods. These two settings are indicative of most settings in our simulation study. Figure 2 c shows the type I error control at the *p*=0.01 threshold for all 100 null settings in which the endpoints are not associated with expression or methylation (*β*_*y*_=0). In all these null settings, UXPR and UMPR fail to show adequate type I error control. Figure 2 d shows the power estimates for all 400 simulation settings in which the endpoint is associated with the molecular data (*β*_*y*_≠0). In the vast majority of these non-null settings, the power of CCPR greatly exceeded that of all other methods with adequate type I error control. In 4 of the 400 (1 %) non-null settings, the power of AMPR slightly exceeded that of CCPR (Table 1). Complete simulation results are available in the Additional files 2 and 3.

**Table 1:** Four simulation settings in which the observed empirical power of AMPR exceeded that of CCPR

*n*	*β*_*y*_	*β*_*x*_	*β*_*m*_	CCPR Power	AMPR Power
500	-0.5	+0.5	-0.3	0.314	0.366
500	+0.5	+0.5	-0.3	0.323	0.381
500	+0.5	-0.5	+0.3	0.343	0.387
500	-0.5	-0.5	+0.3	0.336	0.392

### Acute myeloid leukemia example

To evaluate the practical utility of the CC-PROMISE method, we applied it to a data set obtained from participants of the multi-center AML02 clinical trial [11](NCT00136084) for pediatric patients diagnosed with acute myeloid leukemia. Our analysis considers three endpoints that measure response of leukemic cells to cytarabine. The LC50 endpoint is the dose of cytarabine required to kill 50 % of a patient’s leukemic cells during an in vitro exposure assay. The minimal residual disease (MRD) is the proportion of cells that a flow cytometry assay identifies as leukemic in a bone marrow sample collected after the patient has completed one course of chemotherapy including cytarabine. The event-free survival (EFS) is the duration of time elapsed from study enrollment (within days of diagnosis) until relapse, death, development of a second malignancy, or other catastrophic failure of chemotherapy including cytarabine. The vast majority of treatment failure events are relapses. As described in subsection “Define the most interesting statistical evidence”, we used established methods to measure the association of molecular data with each endpoint and defined the most interesting evidence as a pattern of association statistics indicating greater expression was associated greater sensitivity as measured by all three endpoints. Additional details on the specific association statistics and definition of the PROMISE statistic are available in the Additional file 4. Here, we describe the results for HOXB6, a gene of well-established relevance to the development and prognosis of AML [12, 13]. Complete analysis results and their biological interpretation will be reported elsewhere.

**Fig. 3:**
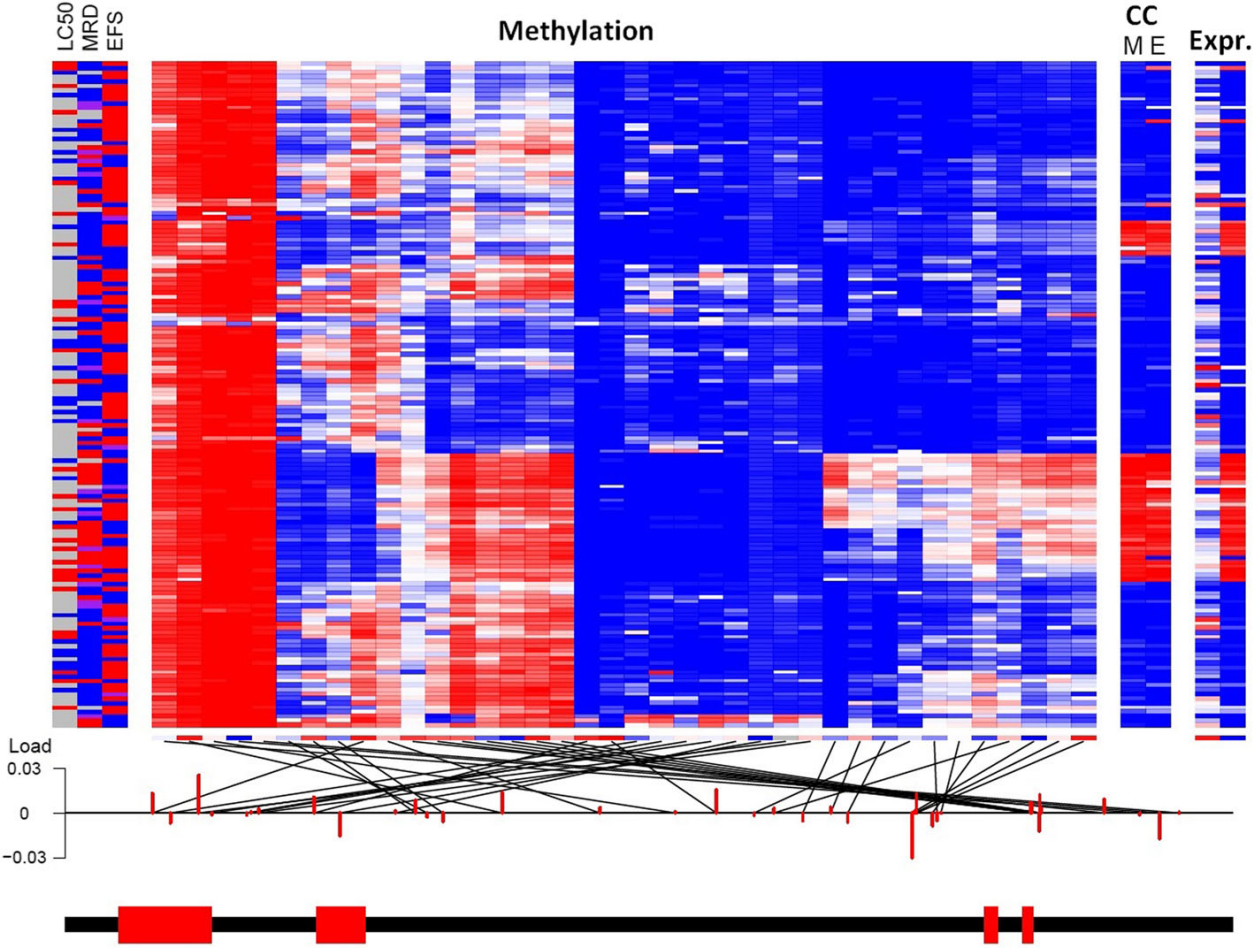
CC-PROMISE Results for HOXB6 in the AML Study. The four heatmaps provide information for each patient in one row and each variable in one column. The leftmost 3-column heatmap provides endpoint data for each subject with values indicating cytarabine resistance in red, values indicating cytarabine sensitivity in blue, intermediate values in purple, and missing values in gray. The large heatmap in the center provides methylation data for each microarray marker with hypermethylation indicated by red and hypomethylation indicated by blue. The genomic locations of the markers are indicated by the lines matching them to genomic position. The rightmost 2-column heatmap provides expression values for each of two microarray probe-sets with greater expression indicated by red and lesser expression indicated by blue. The two-column heatmap in the middle shows the values of the CC scores for methylation and expression with greater values indicated by red and lesser values indicated by blue. The scores show a strong correlation, indicating a strong multivariate correlation between methyaltion and expression

**Fig. 4:**
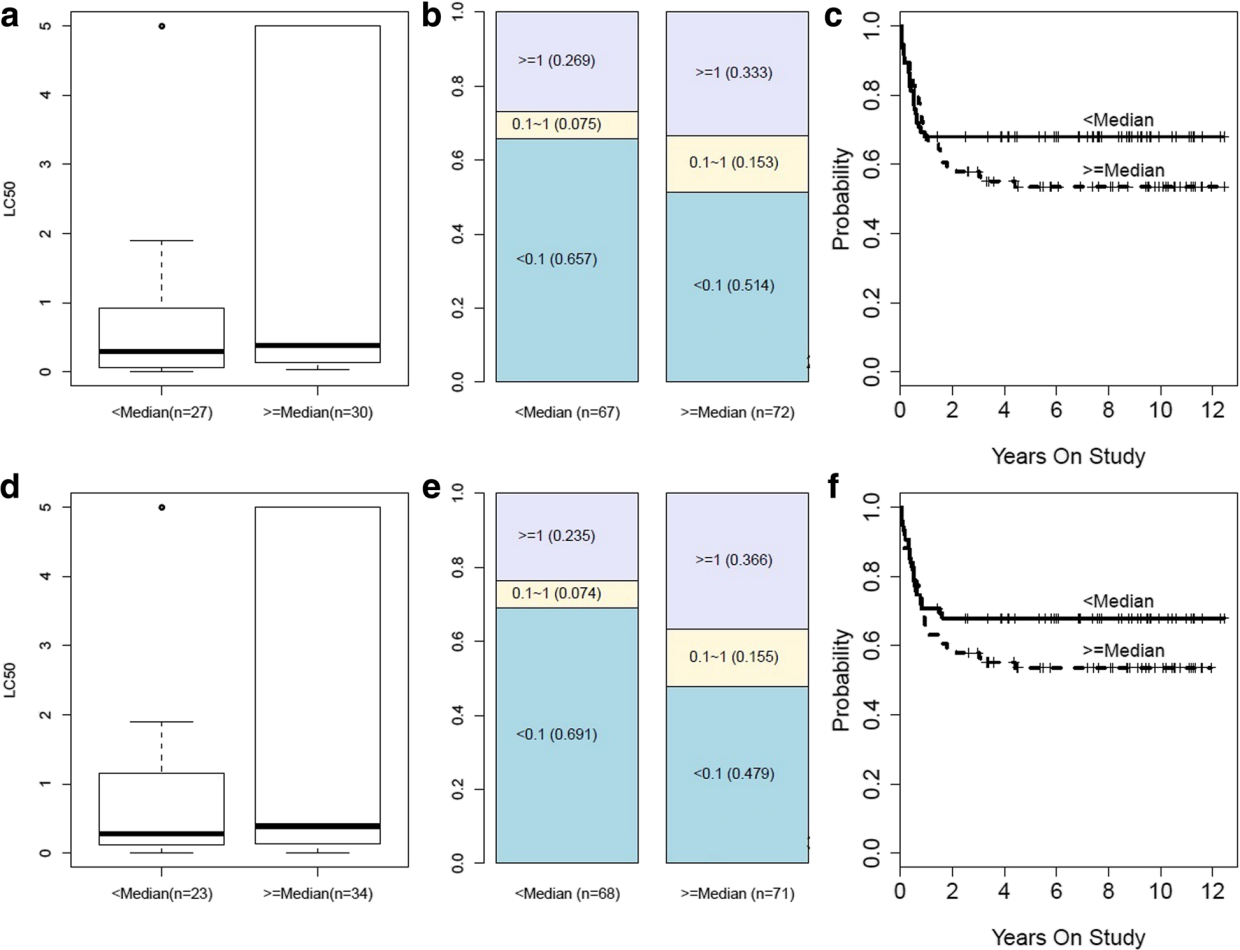
Detailed Endpoint Association Results for HOXB6 in the AML Study. Panel **a** shows a boxplot of LC50 values according to dichotomization of the CC expression scores by the median (low or high). Panel **b** shows the prevalence of undetectable MRD, low MRD, and high MRD according to the dichotomized CC expression score. Panel **c** shows EFS according to the dichotomized CC expression score. Panels **d**, **e**, **f** provide analogous results according to median-dichotomization of the CC methylation score

Figures 3 and 4 show the results for HOXB6. CC found that methylation and expression were very strongly associated with one another ($r_{CC}^{2} = 0.75$; *p*=3.6×10^−15^; CC heatmap in Fig. 3). The complexity of the methylation-expression association is not easily characterized by the biological model that hypermethylation of the promoter suppresses expression. For instance, hypermethylation of a block of markers within the gene body strongly associate with expression (Fig. 3). This finding indicates that CC can help identify novel phenomenon that are not characterized by existing biological models. Increases in the HOXB6 expression CCA score were associated with increased cytarabine resistance (PROMISE *t*=−0.24, *p*=0.00058) as indicated by association with increases in LC50(*p*=0.01, Fig. 4 a), increases in MRD (*p*=0.0034, Fig. 4 b), and reductions in EFS (*p*=0.1038, Fig. 4 c). Also, decreases in the HOXB6 methylation CCA score were associated with increased cytarabine resistance (PROMISE *t*=−0.25, *p*=0.00031) as indicated by association with increases in LC50 (*p*=0.0254, Fig. 4 d), increases in MRD (*p*=0.0021, 4 e), and reduction in EFS (*p*=0.0204, 4 f). Cumulatively, these results strongly indicate that HOXB6 expression and methylation associate with cytarabine response in AML (CC-PROMISE *t*=−0.24, *p*=0.00012).

**Fig. 5:**
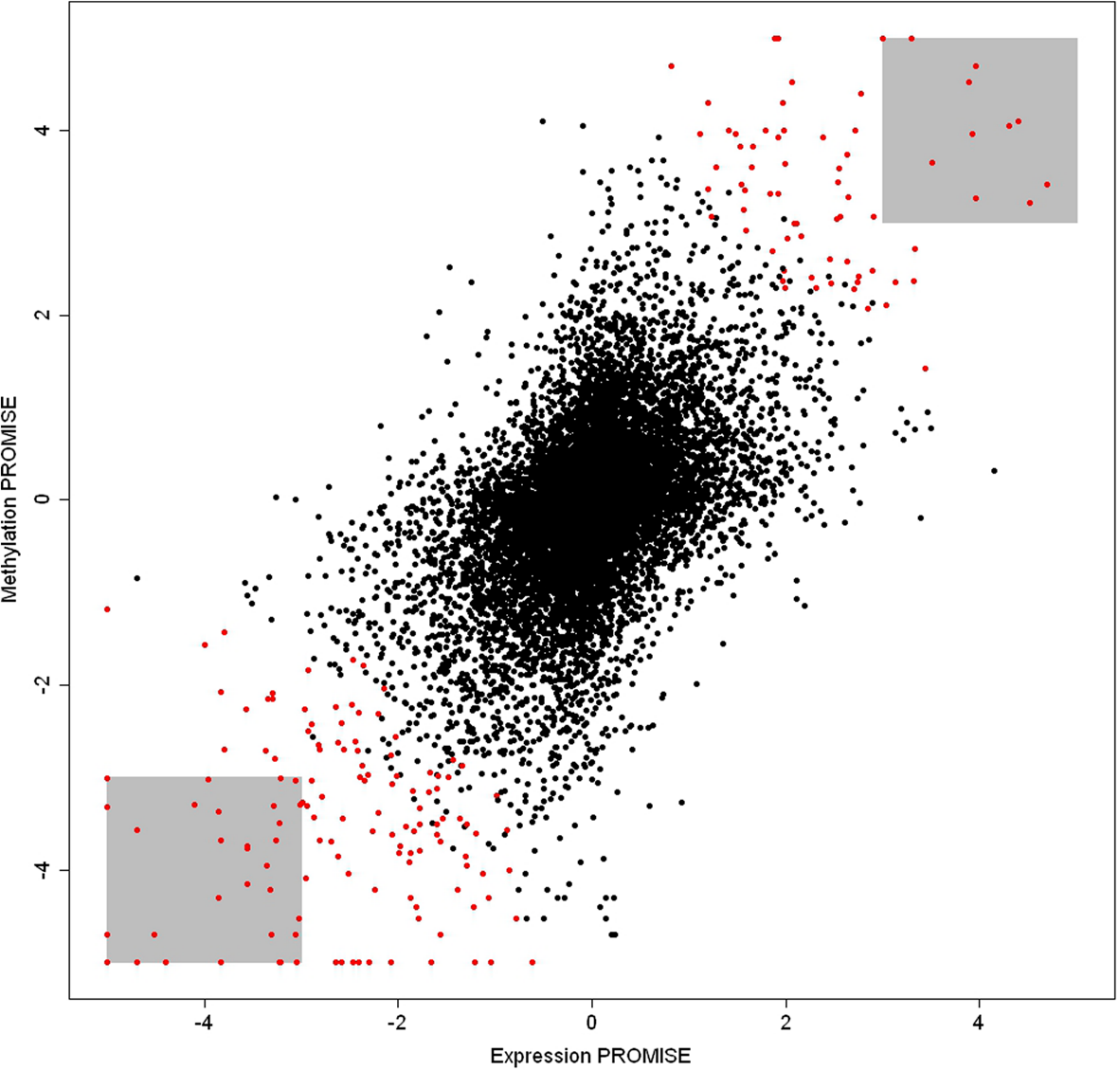
CC-PROMISE Identifies More Genes than Does Overlap of Expression PROMISE and Methylation PROMISE. The figure shows a scatterplot of the signed log_10_(*p*) statistics from the expression PROMISE and methylation PROMISE for each of 11,620 genes with methylation and expression data. The gray rectangles capture the 46 genes significant at *p*≤0.001 by both expression PROMISE and methylation PROMISE. The points colored in red correspond to the 204 genes identified as significant at the *p*≤0.001 level by CC-PROMISE

Figure 5 shows that CC-PROMISE identifies more genes as significant than does overlap of the methylation and expression PROMISE analyses. A total of 46 genes were identified as significant with *p*≤0.001 in both the methylation PROMISE and expression PROMISE analyses. CC-PROMISE identified 204 genes as significant at the *p*≤0.001 level including all 46 genes identified by overlap of the methylation PROMISE and expression PROMISE results. In this application, CC-PROMISE achieved better statistical power than overlap of individual PROMISE analyses by defining a rejection region that encompasses the overlap rejection region as described in subsection “Define the most interesting statistical evidence” and illustrated in Fig. 1.

We recognize that experimental validation of the findings is necessary, but are still confident that many of the 204 genes identified by CC-PROMISE are biologically meaningful. We expect using a *p*-value threshold of 0.001 for testing 11,620 genes to yield only 0.001×11,620=11.6 false discoveries. Thus, most of the 204 genes identified by CC-PROMISE are expected to be authentic discoveries. The *p*-values were computed by permutation, which is widely recognized for rigorous control of Type I errors (incorrect rejection of the null). Additionally, the *p*-values of all three PROMISE analyses were computed using the same set of permutations so all three methods were provided identical protection against Type I errors. Thus, it is statistically meaningful that CC-PROMISE identified more genes than did other methods.

## Discussion and conclusion

Effective integrated data analysis methods are essential to the success of biomedical research that collects multiple forms of molecular data and multiple endpoints from subjects. Simplistic list overlap approaches have been used successfully in some studies. However, it is clear that the statistical limitations of list overlap approaches will impede the success of other studies. Therefore, it is imperative that the scientific community develop and routinely apply innovative methods for integrated analysis of multiple forms of molecular data with multiple clinical endpoints.

Projection onto the most interesting statistical evidence (PROMISE) is an effective method to integrate one form of molecular data with multiple clinical endpoints. The PROMISE method is a statistically rigorous and robust method that overcomes many of the limitations of widely used list-overlap approaches. Here, we use canonical correlation analysis and PROMISE to develop CC-PROMISE as an effective method for integrating two forms of molecular data with multiple clinical endpoints. In our simulation studies and example application, CC-PROMISE shows similar benefits relative to list-overlap approaches.

In subsection “Define the most interesting statistical evidence”, this work provides the first algorithmic procedure to determine the coefficients *λ*_*k*_ of the endpoint association statistics that define a PROMISE statistic. This provides one method to objectively define the PROMISE statistic for future applications. In some other applications involving the PROMISE statistic, the selection of coefficients in the PROMISE statistic has been arbitrary. Still, defining coefficients that accurately characterize the true biological associations of the endpoints to one another is a critical element of successfully using PROMISE to make authentic biological discoveries in practice. Therefore, further research should develop and evaluate methods to define the coefficients in a biologically meaningful and objective manner.

There are several other opportunities to explore in future research. One direction that is very closely related to this work would be to extend the PROMISE framework to integrate multiple endpoints with more than two forms of molecular data. Some methods that generalize canonical correlation analysis to analysis of more than two multivariate data sets [14, 15] may be useful building blocks for such approaches. Another interesting direction would be to develop methods that use the data to empirically define coefficients for defining the PROMISE statistic. It may also be worthwhile to develop methods based on fundamentally different conceptual frameworks for integrated data analysis such as formal joint modeling of multiple forms of genomic data [16]. Such joint-modeling methods are mathematically elegant and incorporating biological knowledge of endpoint-endpoint relationships may provide substantial practical and statistical benefits.

## Additional files

Additional file 1 This PDF file provides a notation glossary in tabular form. It provides notation and interpretation of most of the mathematical symbols used in the manuscript. (PDF 60 kb)

Additional file 2 This PDF file provides plots of the probability of significance as a function of the *p*-value threshold for all seven analysis methods in each of the 500 simulation settings. The plots have a similar interpretation as those in Fig. 2. (PDF 1263 kb)

Additional file 3 Each row of this table provides power estimates for each method in one simulated setting. The columns labeled *setting*, *n*, *beta.y*, *beta.x*, and *beta.m* provide the setting index number, the sample size *n*, and the values of the parameters *β*_*y*_, *β*_*x*_, and *β*_*m*_, respectively. (XLSX 41 kb)

Additional file 4 This PDF file provides technical details regarding the CC-PROMISE analysis performed for the example application involving pediatric acute myeloid leukemia described in subsection “Acute myeloid leukemia example”. Details include description of the specific association statistics used for each molecular data set and each endpoint, the coefficients used in the analysis, and the number of permutations performed. (PDF 266 kb)
