# Supplementary material for: CC-PROMISE effectively integrates two forms of molecular data with multiple biologically related endpoints
Source: BMC Bioinformatics. 2016 Oct 6;17(Suppl 13):382. doi: 10.1186/s12859-016-1217-0 (PMC5073973; doi:10.1186/s12859-016-1217-0)
Supplement: Additional file 1 — This PDF file provides a notation glossary in tabular form. It provides notation and interpretation of most of the mathematical symbols used in the manuscript. (PDF 60 kb) [file 12859_2016_1217_MOESM1_ESM.pdf]

Table 1: Glossary of Mathematical Notation.

Notation	Meaning
$i = 1, \ldots, n$	indexes subjects
$g = 1, \ldots, G$	indexes genes
$l_g = 1, \ldots, L_g$	indexes loci of methylation markers of gene g; subscript g often omitted for simplicity
m_{gli}	methylation of locus l_g of gene g for subject i
$f_g = 1, \ldots, F_g$	indexes expression features of gene g; subscript g often omitted for simplicity
x_{gfi}	expression of feature f of gene g for subject i
$k = 1, \ldots, K$	indexes endpoints or phenotypes
y_{ki}	value of endpoint k for subject i
r_{gfl}	the correlation of the expression of feature f of gene g with the methylation of locus l of gene g
p_{gfl}	the p-value for testing the null hypothesis that the expression of feature f of gene g is not correlated with the methylation of locus l
a_{kgf}	association of endpoint k with expression of feature f of gene g
a_{kgl}	association of endpoint k with methylation of locus l of gene g
λ_k	the coefficient of a_{kgl} or a_{kgf} defining the projection onto the most interesting statistical evidence (PROMISE)
$t_{gf} = \sum_{k=1}^{K} \lambda_k a_{kgf}$	the PROMISE statistic for expression of feature f of gene g
$t_{gl} = \sum_{k=1}^{K} \lambda_k a_{kgl}$	the PROMISE statistic for methylation of locus l of gene g
$t^{\star}_{glf} = t_{gf} + \text{sign}(r_{gfl}) t_{gl}$	the combined PROMISE statistic for methylation locus l and expression feature f of gene g
M_g	the matrix of m_{gli} values for all loci l_g and all subjects i
X_g	the matrix of x_{gfi} values for all features f_g and all subjects i
$\tilde{r}_g$	the canonical correlation of the methylation M_g and expression X_g of gene g
$\tilde{p}_g$	the p-value for testing that the canonical correlation of the methylation M_g and X_g is zero.
$\tilde{m}_g$	the methylation score (first canonical correlate) obtained from canonical correlation analysis of M_g and X_g
$\tilde{x}_g$	the expression score (first canonical correlate) obtained from canonical correlation of M_g and X_g
$\tilde{a}_{kgm}$	the association of endpoint k with the methylation score $\tilde{m}_g$ of gene g
$\tilde{a}_{kgx}$	the association of endpoint k with the expression score $\tilde{x}_g$ of gene g
$\tilde{t}_{gx} = \sum_{k=1}^{K} \lambda_k \tilde{a}_{kgx}$	the PROMISE statistic for the expression score $\tilde{x}_g$ of gene g
$\tilde{t}_{gm} = \sum_{k=1}^{K} \lambda_k \tilde{a}_{kgm}$	the PROMISE statistic for the methylation score $\tilde{m}$ of gene g
$\tilde{t}^{\star}_g = \tilde{t}^{\star}_{gx} + \text{sign}(\tilde{r}_g) \tilde{t}^{\star}_{gm}$	the combined PROMISE statistic for methylation and expression of gene g
