# Supplementary material for: CC-PROMISE effectively integrates two forms of molecular data with multiple biologically related endpoints
Source: BMC Bioinformatics. 2016 Oct 6;17(Suppl 13):382. doi: 10.1186/s12859-016-1217-0 (PMC5073973; doi:10.1186/s12859-016-1217-0)
Supplement: Additional file 2 — This PDF file provides plots of the probability of significance as a function of the p-value threshold for all seven analysis methods in each of the 500 simulation settings. The plots have a similar interpretation as those in Fig. 2. (PDF 1263 kb) [file 12859_2016_1217_MOESM2_ESM.pdf]

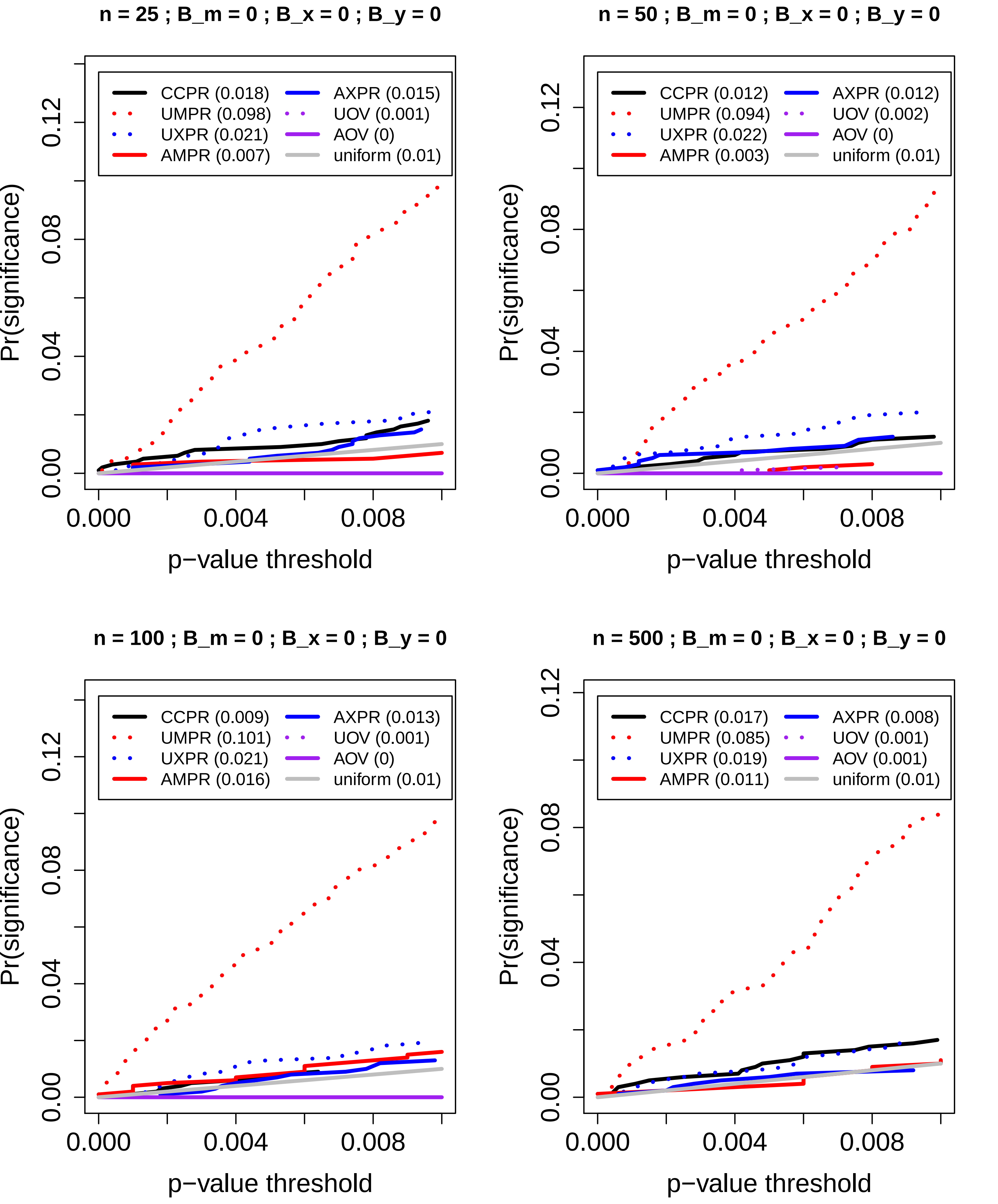

n = 25 ; B_m = 0 ; B_x = 0 ; B_y = 0
CCPR (0.018)
UMPR (0.098)
UXPR (0.021)
AMPR (0.007)
AXPR (0.015)
UOV (0.001)
AOV (0)
uniform (0.01)
n = 50 ; B_m = 0 ; B_x = 0 ; B_y = 0
CCPR (0.012)
UMPR (0.094)
UXPR (0.022)
AMPR (0.003)
AXPR (0.012)
UOV (0.002)
AOV (0)
uniform (0.01)
n = 100 ; B_m = 0 ; B_x = 0 ; B_y = 0
CCPR (0.009)
UMPR (0.101)
UXPR (0.021)
AMPR (0.016)
AXPR (0.013)
UOV (0.001)
AOV (0)
uniform (0.01)
n = 500 ; B_m = 0 ; B_x = 0 ; B_y = 0
CCPR (0.017)
UMPR (0.085)
UXPR (0.019)
AMPR (0.011)
AXPR (0.008)
UOV (0.001)
AOV (0.001)
uniform (0.01)
Pr(significance)
p−value threshold
0.00
0.04
0.08
0.12
0.000
0.004
0.008

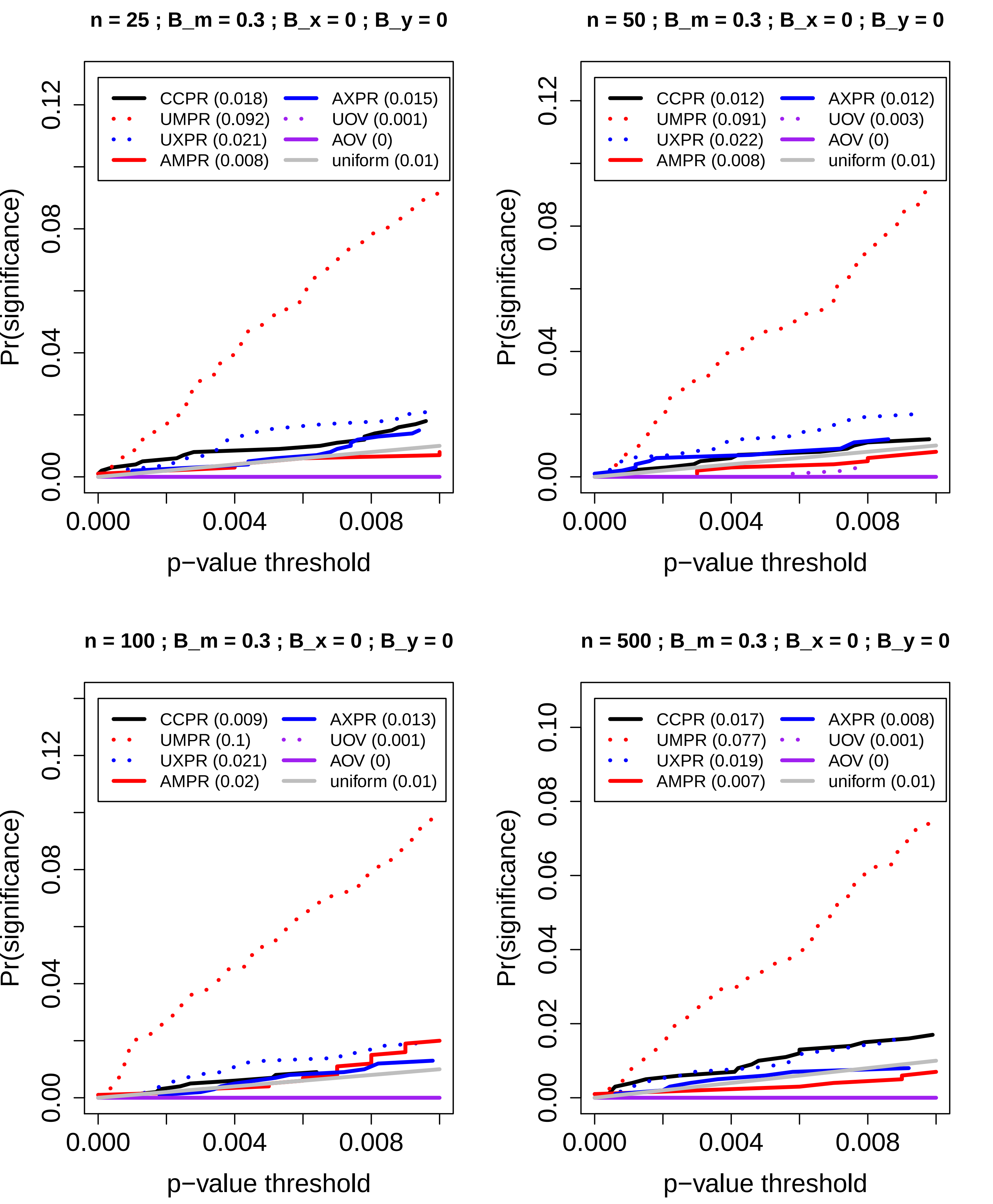
n = 25 ; B_m = 0.3 ; B_x = 0 ; B_y = 0
CCPR (0.018)
UMPR (0.092)
UXPR (0.021)
AMPR (0.008)
AXPR (0.015)
UOV (0.001)
AOV (0)
uniform (0.01)
n = 50 ; B_m = 0.3 ; B_x = 0 ; B_y = 0
CCPR (0.012)
UMPR (0.091)
UXPR (0.022)
AMPR (0.008)
AXPR (0.012)
UOV (0.003)
AOV (0)
uniform (0.01)
n = 100 ; B_m = 0.3 ; B_x = 0 ; B_y = 0
CCPR (0.009)
UMPR (0.1)
UXPR (0.021)
AMPR (0.02)
AXPR (0.013)
UOV (0.001)
AOV (0)
uniform (0.01)
n = 500 ; B_m = 0.3 ; B_x = 0 ; B_y = 0
CCPR (0.017)
UMPR (0.077)
UXPR (0.019)
AMPR (0.007)
AXPR (0.008)
UOV (0.001)
AOV (0)
uniform (0.01)
Pr(significance)
p−value threshold
0.000
0.004
0.008
0.00
0.02
0.04
0.06
0.08
0.10
0.12

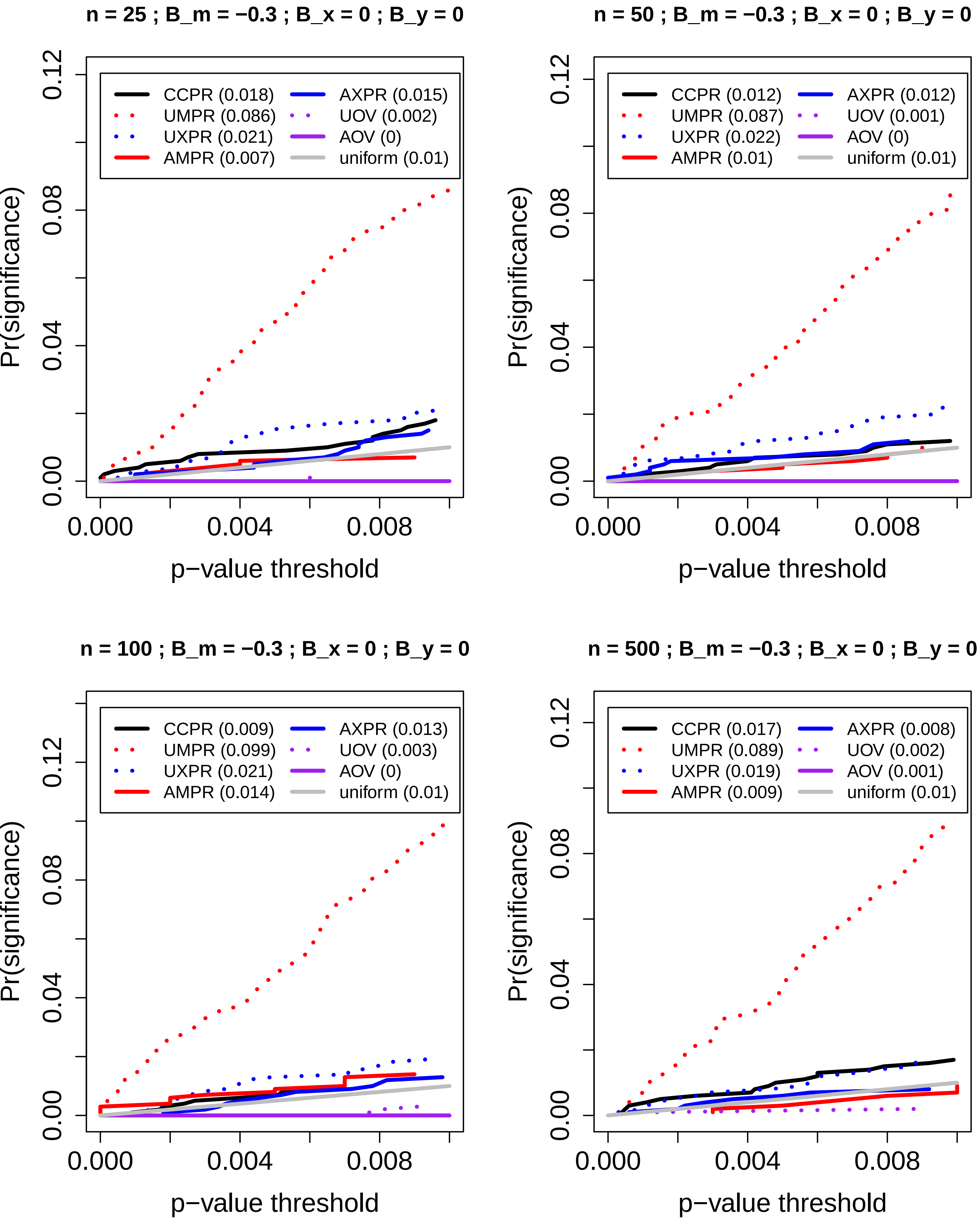

n = 25 ; B_m = −0.3 ; B_x = 0 ; B_y = 0
CCPR (0.018)
UMPR (0.086)
UXPR (0.021)
AMPR (0.007)
AXPR (0.015)
UOV (0.002)
AOV (0)
uniform (0.01)
Pr(significance)
p−value threshold
n = 50 ; B_m = −0.3 ; B_x = 0 ; B_y = 0
CCPR (0.012)
UMPR (0.087)
UXPR (0.022)
AMPR (0.01)
AXPR (0.012)
UOV (0.001)
AOV (0)
uniform (0.01)
Pr(significance)
p−value threshold
n = 100 ; B_m = −0.3 ; B_x = 0 ; B_y = 0
CCPR (0.009)
UMPR (0.099)
UXPR (0.021)
AMPR (0.014)
AXPR (0.013)
UOV (0.003)
AOV (0)
uniform (0.01)
Pr(significance)
p−value threshold
n = 500 ; B_m = −0.3 ; B_x = 0 ; B_y = 0
CCPR (0.017)
UMPR (0.089)
UXPR (0.019)
AMPR (0.009)
AXPR (0.008)
UOV (0.002)
AOV (0.001)
uniform (0.01)
Pr(significance)
p−value threshold

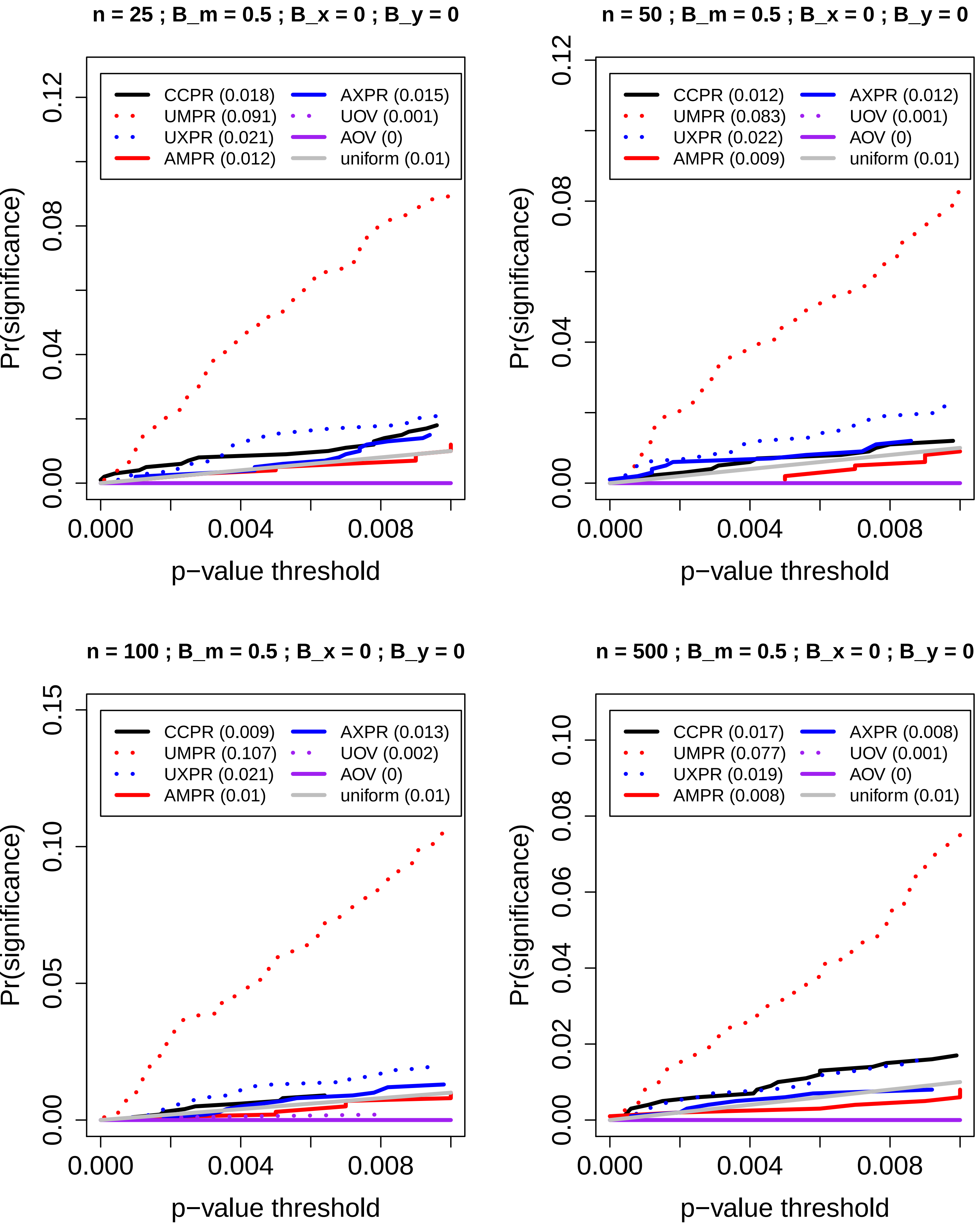

n = 25 ; B_m = 0.5 ; B_x = 0 ; B_y = 0
CCPR (0.018)
UMPR (0.091)
UXPR (0.021)
AMPR (0.012)
AXPR (0.015)
UOV (0.001)
AOV (0)
uniform (0.01)
Pr(significance)
p−value threshold
n = 50 ; B_m = 0.5 ; B_x = 0 ; B_y = 0
CCPR (0.012)
UMPR (0.083)
UXPR (0.022)
AMPR (0.009)
AXPR (0.012)
UOV (0.001)
AOV (0)
uniform (0.01)
Pr(significance)
p−value threshold
n = 100 ; B_m = 0.5 ; B_x = 0 ; B_y = 0
CCPR (0.009)
UMPR (0.107)
UXPR (0.021)
AMPR (0.01)
AXPR (0.013)
UOV (0.002)
AOV (0)
uniform (0.01)
Pr(significance)
p−value threshold
n = 500 ; B_m = 0.5 ; B_x = 0 ; B_y = 0
CCPR (0.017)
UMPR (0.077)
UXPR (0.019)
AMPR (0.008)
AXPR (0.008)
UOV (0.001)
AOV (0)
uniform (0.01)
Pr(significance)
p−value threshold

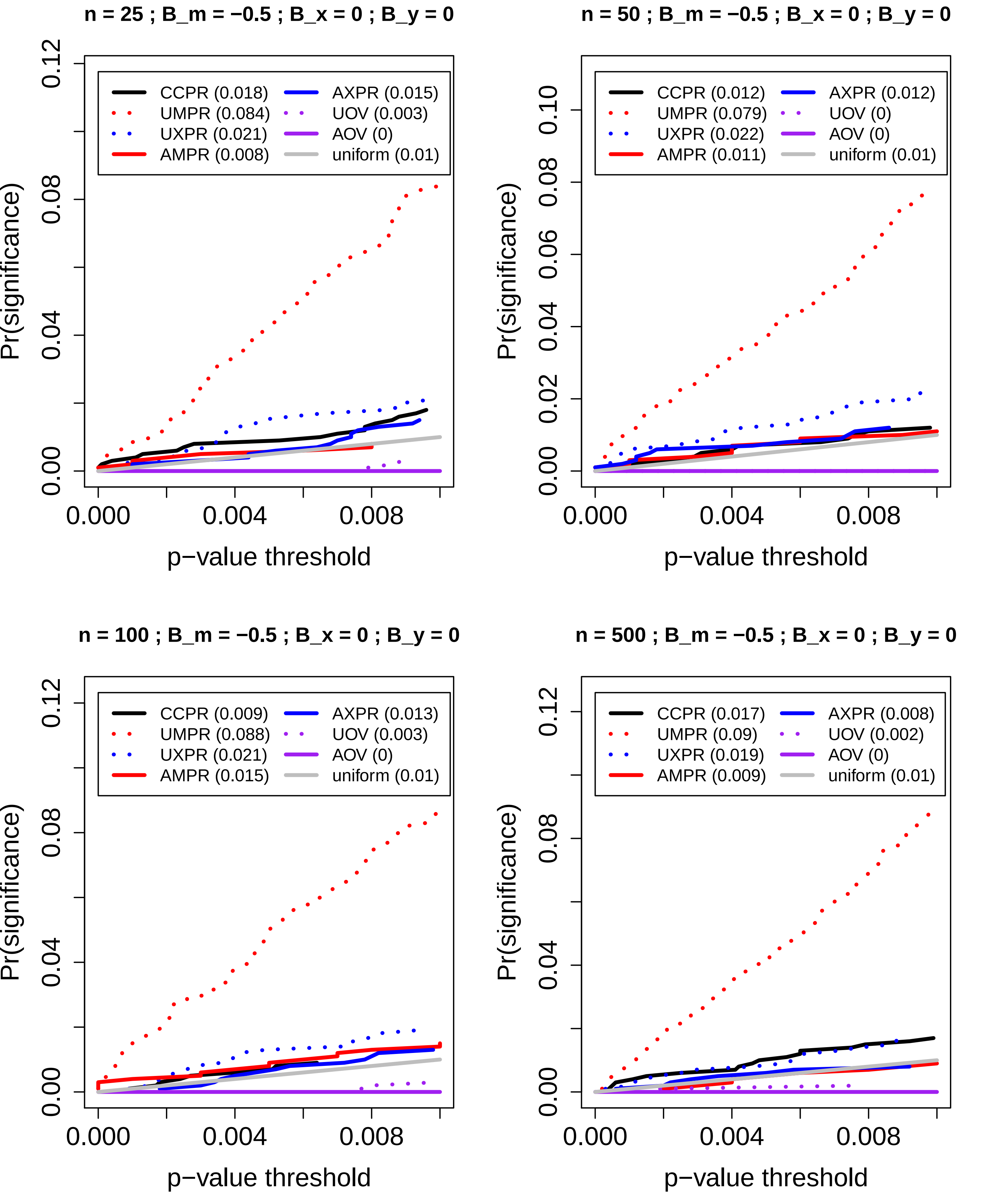

n = 25 ; B_m = −0.5 ; B_x = 0 ; B_y = 0
CCPR (0.018)
UMPR (0.084)
UXPR (0.021)
AMPR (0.008)
AXPR (0.015)
UOV (0.003)
AOV (0)
uniform (0.01)
n = 50 ; B_m = −0.5 ; B_x = 0 ; B_y = 0
CCPR (0.012)
UMPR (0.079)
UXPR (0.022)
AMPR (0.011)
AXPR (0.012)
UOV (0)
AOV (0)
uniform (0.01)
n = 100 ; B_m = −0.5 ; B_x = 0 ; B_y = 0
CCPR (0.009)
UMPR (0.088)
UXPR (0.021)
AMPR (0.015)
AXPR (0.013)
UOV (0.003)
AOV (0)
uniform (0.01)
n = 500 ; B_m = −0.5 ; B_x = 0 ; B_y = 0
CCPR (0.017)
UMPR (0.09)
UXPR (0.019)
AMPR (0.009)
AXPR (0.008)
UOV (0.002)
AOV (0)
uniform (0.01)
Pr(significance)
p−value threshold
0.000
0.004
0.008
0.00
0.02
0.04
0.06
0.08
0.10
0.12

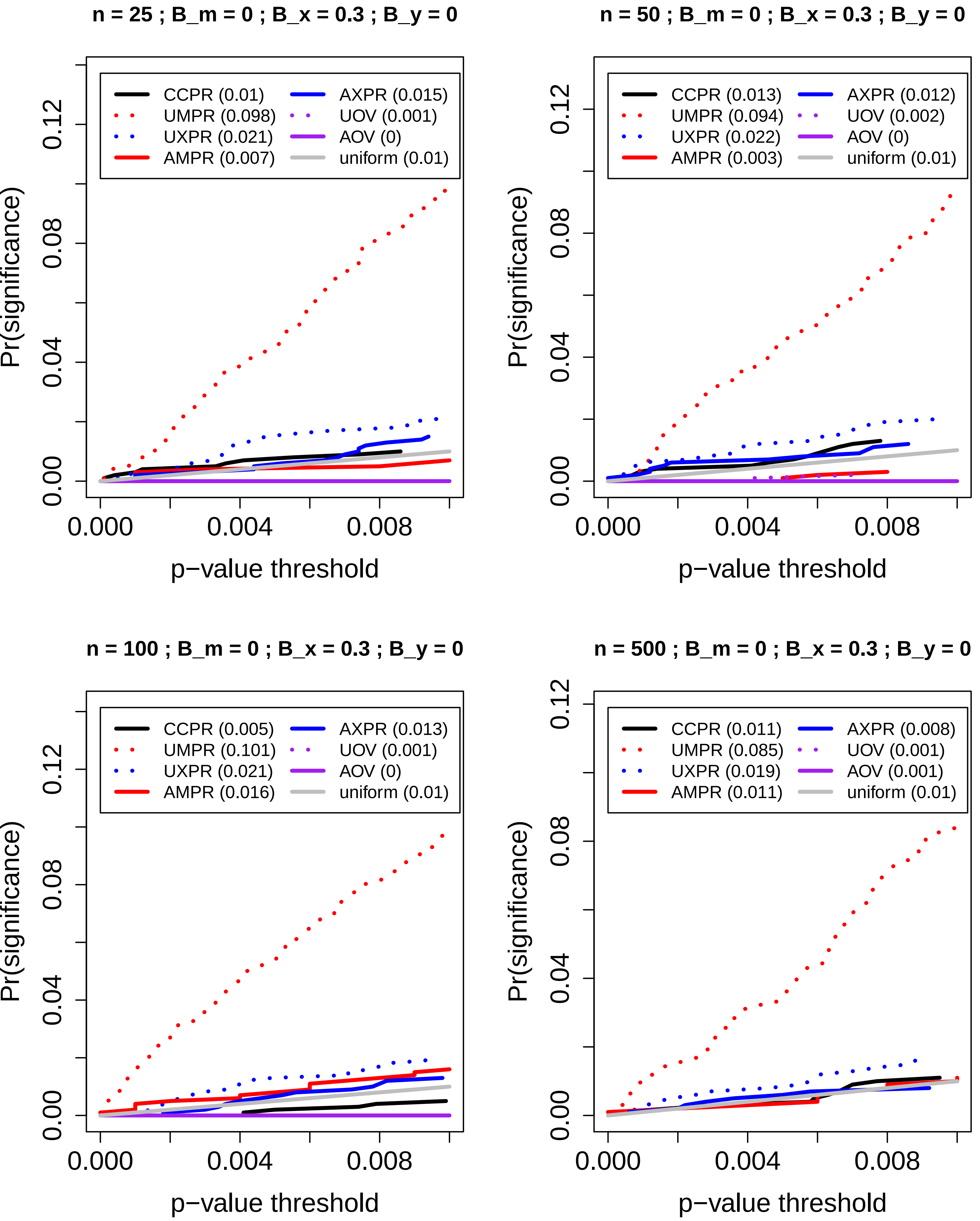

n = 25 ; B_m = 0 ; B_x = 0.3 ; B_y = 0
CCPR (0.01)
UMPR (0.098)
UXPR (0.021)
AMPR (0.007)
AXPR (0.015)
UOV (0.001)
AOV (0)
uniform (0.01)
n = 50 ; B_m = 0 ; B_x = 0.3 ; B_y = 0
CCPR (0.013)
UMPR (0.094)
UXPR (0.022)
AMPR (0.003)
AXPR (0.012)
UOV (0.002)
AOV (0)
uniform (0.01)
n = 100 ; B_m = 0 ; B_x = 0.3 ; B_y = 0
CCPR (0.005)
UMPR (0.101)
UXPR (0.021)
AMPR (0.016)
AXPR (0.013)
UOV (0.001)
AOV (0)
uniform (0.01)
n = 500 ; B_m = 0 ; B_x = 0.3 ; B_y = 0
CCPR (0.011)
UMPR (0.085)
UXPR (0.019)
AMPR (0.011)
AXPR (0.008)
UOV (0.001)
AOV (0.001)
uniform (0.01)
Pr(significance)
0.00
0.04
0.08
0.12
p−value threshold
0.000
0.004
0.008

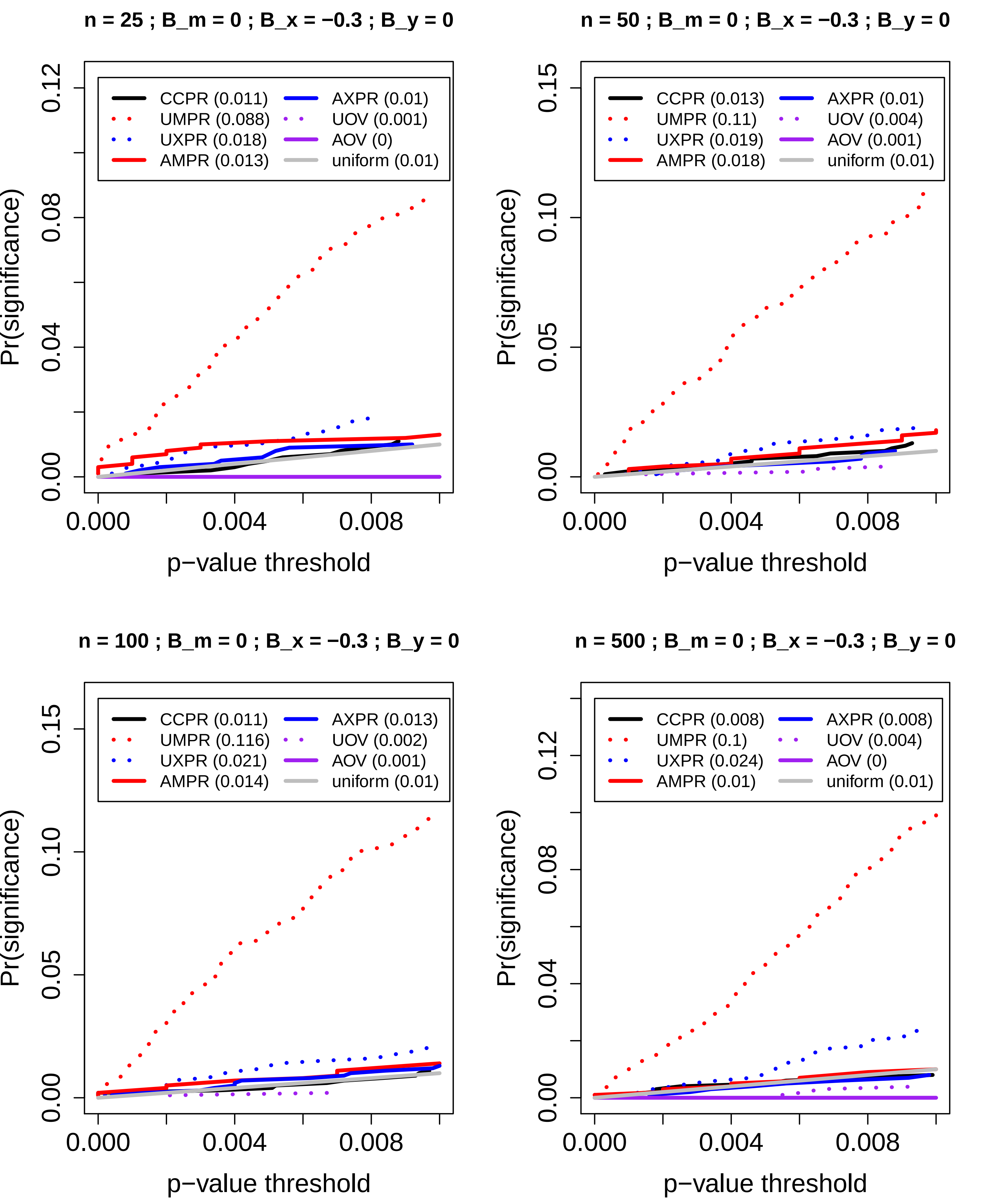
n = 25 ; B_m = 0 ; B_x = −0.3 ; B_y = 0
CCPR (0.011)
UMPR (0.088)
UXPR (0.018)
AMPR (0.013)
AXPR (0.01)
UOV (0.001)
AOV (0)
uniform (0.01)
Pr(significance)
p−value threshold
n = 50 ; B_m = 0 ; B_x = −0.3 ; B_y = 0
CCPR (0.013)
UMPR (0.11)
UXPR (0.019)
AMPR (0.018)
AXPR (0.01)
UOV (0.004)
AOV (0.001)
uniform (0.01)
Pr(significance)
p−value threshold
n = 100 ; B_m = 0 ; B_x = −0.3 ; B_y = 0
CCPR (0.011)
UMPR (0.116)
UXPR (0.021)
AMPR (0.014)
AXPR (0.013)
UOV (0.002)
AOV (0.001)
uniform (0.01)
Pr(significance)
p−value threshold
n = 500 ; B_m = 0 ; B_x = −0.3 ; B_y = 0
CCPR (0.008)
UMPR (0.1)
UXPR (0.024)
AMPR (0.01)
AXPR (0.008)
UOV (0.004)
AOV (0)
uniform (0.01)
Pr(significance)
p−value threshold

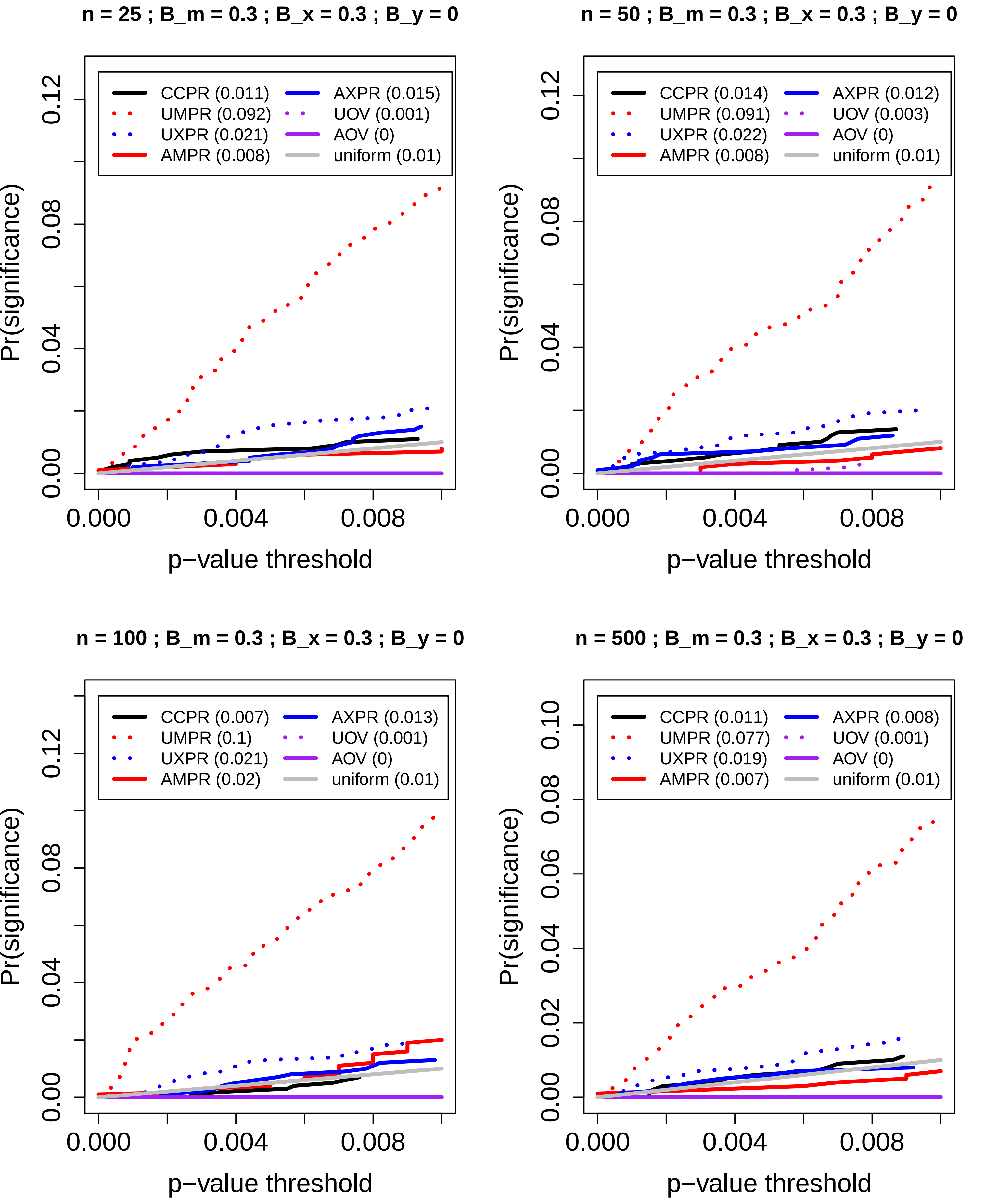
n = 25 ; B_m = 0.3 ; B_x = 0.3 ; B_y = 0
CCPR (0.011)
UMPR (0.092)
UXPR (0.021)
AMPR (0.008)
AXPR (0.015)
UOV (0.001)
AOV (0)
uniform (0.01)
n = 50 ; B_m = 0.3 ; B_x = 0.3 ; B_y = 0
CCPR (0.014)
UMPR (0.091)
UXPR (0.022)
AMPR (0.008)
AXPR (0.012)
UOV (0.003)
AOV (0)
uniform (0.01)
n = 100 ; B_m = 0.3 ; B_x = 0.3 ; B_y = 0
CCPR (0.007)
UMPR (0.1)
UXPR (0.021)
AMPR (0.02)
AXPR (0.013)
UOV (0.001)
AOV (0)
uniform (0.01)
n = 500 ; B_m = 0.3 ; B_x = 0.3 ; B_y = 0
CCPR (0.011)
UMPR (0.077)
UXPR (0.019)
AMPR (0.007)
AXPR (0.008)
UOV (0.001)
AOV (0)
uniform (0.01)
Pr(significance)
p−value threshold
0.000
0.004
0.008
0.00
0.02
0.04
0.06
0.08
0.10
0.12

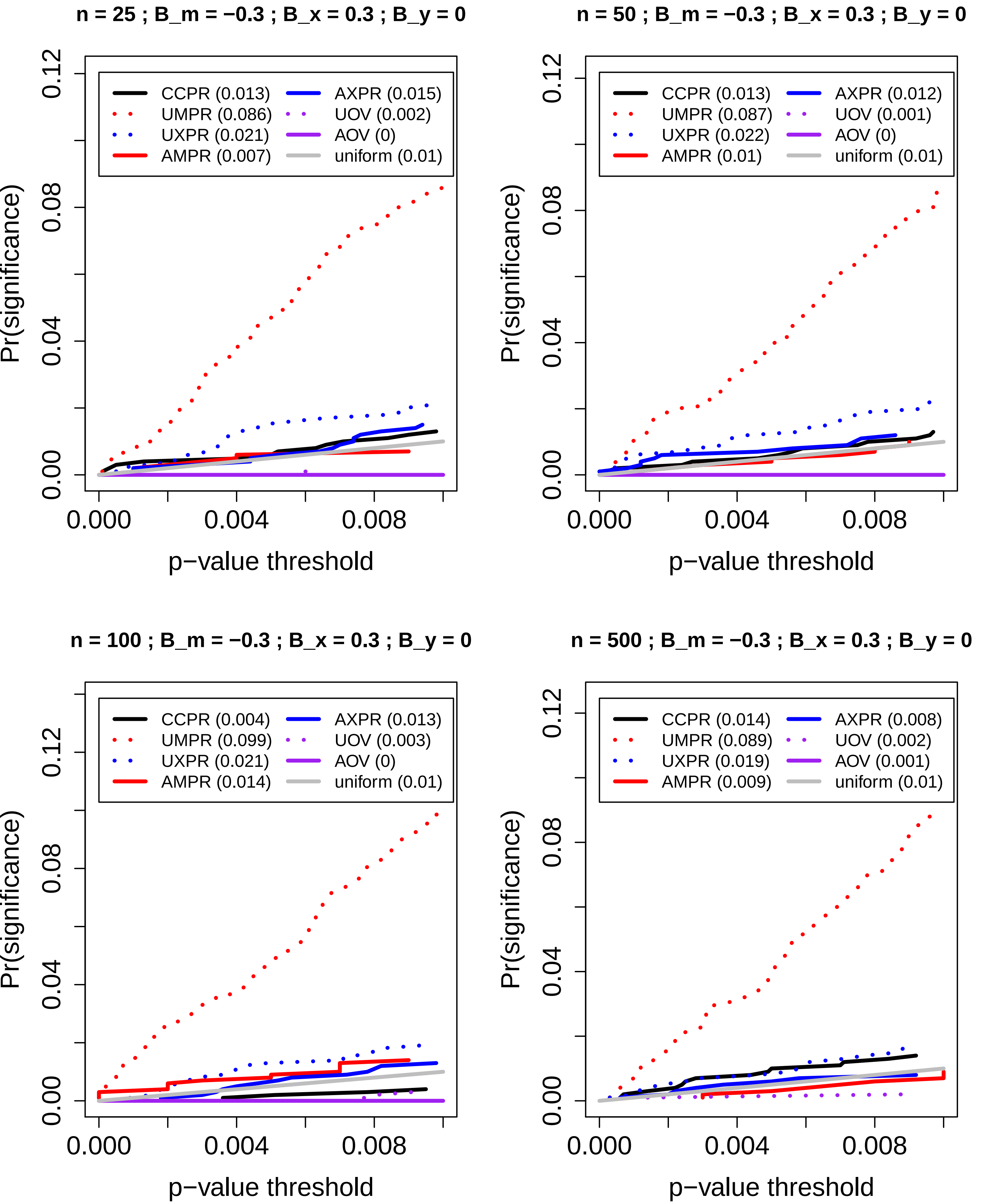

n = 25 ; B_m = −0.3 ; B_x = 0.3 ; B_y = 0
CCPR (0.013)
UMPR (0.086)
UXPR (0.021)
AMPR (0.007)
AXPR (0.015)
UOV (0.002)
AOV (0)
uniform (0.01)
n = 50 ; B_m = −0.3 ; B_x = 0.3 ; B_y = 0
CCPR (0.013)
UMPR (0.087)
UXPR (0.022)
AMPR (0.01)
AXPR (0.012)
UOV (0.001)
AOV (0)
uniform (0.01)
n = 100 ; B_m = −0.3 ; B_x = 0.3 ; B_y = 0
CCPR (0.004)
UMPR (0.099)
UXPR (0.021)
AMPR (0.014)
AXPR (0.013)
UOV (0.003)
AOV (0)
uniform (0.01)
n = 500 ; B_m = −0.3 ; B_x = 0.3 ; B_y = 0
CCPR (0.014)
UMPR (0.089)
UXPR (0.019)
AMPR (0.009)
AXPR (0.008)
UOV (0.002)
AOV (0.001)
uniform (0.01)
Pr(significance)
p−value threshold
0.00
0.04
0.08
0.12
0.000
0.004
0.008

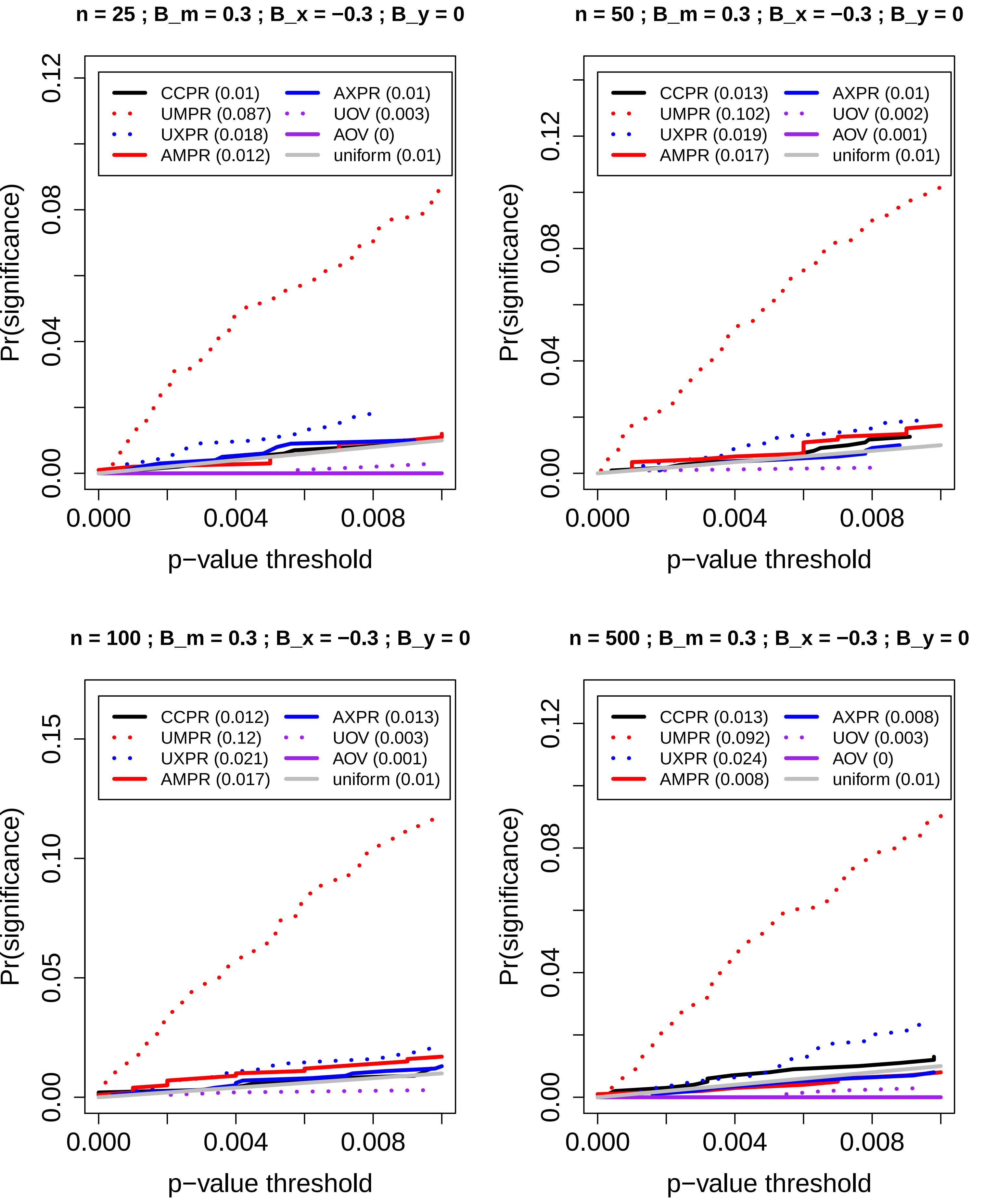
n = 25 ; B_m = 0.3 ; B_x = −0.3 ; B_y = 0
CCPR (0.01)
UMPR (0.087)
UXPR (0.018)
AMPR (0.012)
AXPR (0.01)
UOV (0.003)
AOV (0)
uniform (0.01)
n = 50 ; B_m = 0.3 ; B_x = −0.3 ; B_y = 0
CCPR (0.013)
UMPR (0.102)
UXPR (0.019)
AMPR (0.017)
AXPR (0.01)
UOV (0.002)
AOV (0.001)
uniform (0.01)
n = 100 ; B_m = 0.3 ; B_x = −0.3 ; B_y = 0
CCPR (0.012)
UMPR (0.12)
UXPR (0.021)
AMPR (0.017)
AXPR (0.013)
UOV (0.003)
AOV (0.001)
uniform (0.01)
n = 500 ; B_m = 0.3 ; B_x = −0.3 ; B_y = 0
CCPR (0.013)
UMPR (0.092)
UXPR (0.024)
AMPR (0.008)
AXPR (0.008)
UOV (0.003)
AOV (0)
uniform (0.01)
Pr(significance)
p−value threshold
0.000
0.004
0.008
0.00
0.04
0.08
0.12
0.05
0.10
0.15

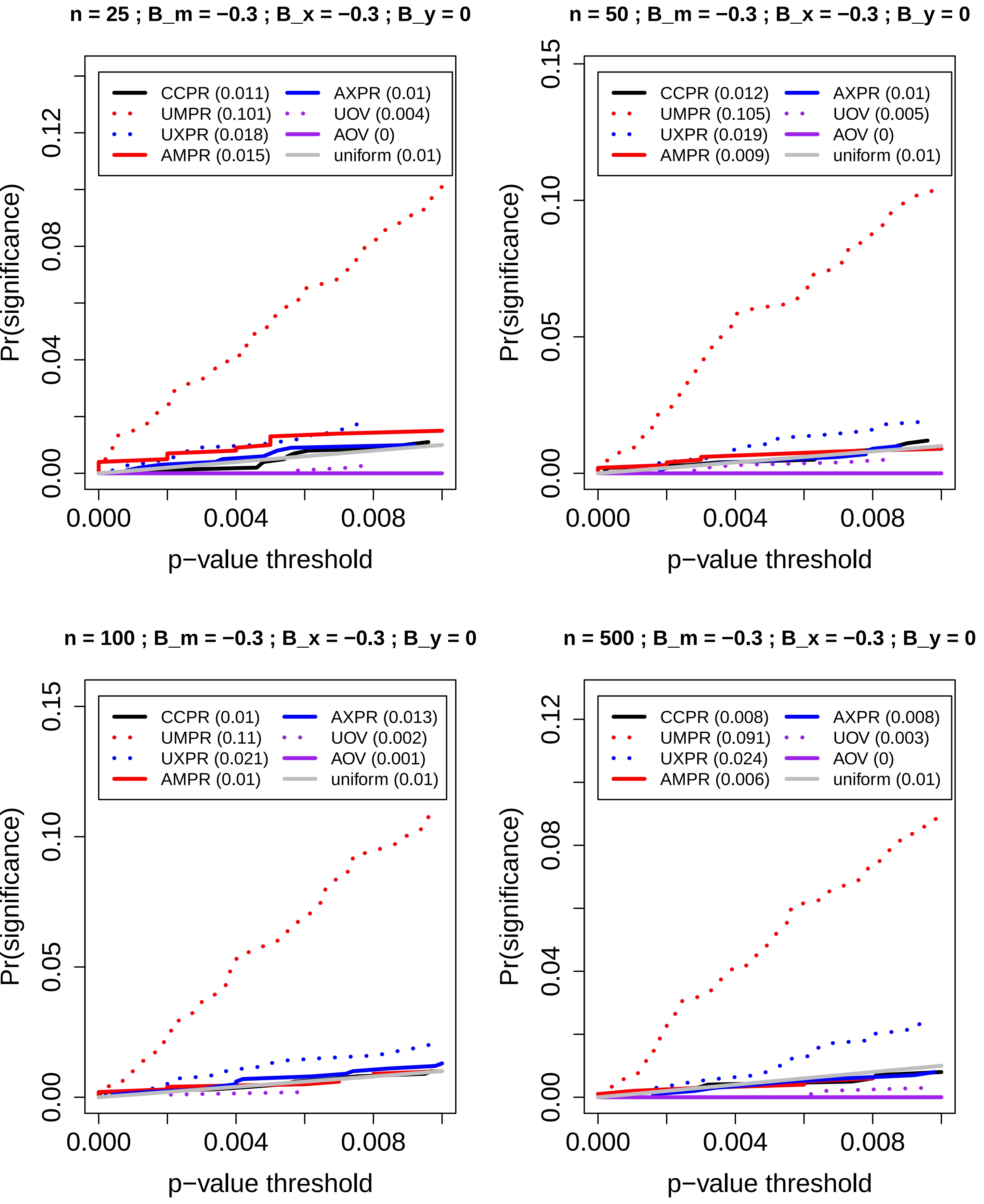

n = 25 ; B_m = −0.3 ; B_x = −0.3 ; B_y = 0
CCPR (0.011)
UMPR (0.101)
UXPR (0.018)
AMPR (0.015)
AXPR (0.01)
UOV (0.004)
AOV (0)
uniform (0.01)
Pr(significance)
p−value threshold
n = 50 ; B_m = −0.3 ; B_x = −0.3 ; B_y = 0
CCPR (0.012)
UMPR (0.105)
UXPR (0.019)
AMPR (0.009)
AXPR (0.01)
UOV (0.005)
AOV (0)
uniform (0.01)
Pr(significance)
p−value threshold
n = 100 ; B_m = −0.3 ; B_x = −0.3 ; B_y = 0
CCPR (0.01)
UMPR (0.11)
UXPR (0.021)
AMPR (0.01)
AXPR (0.013)
UOV (0.002)
AOV (0.001)
uniform (0.01)
Pr(significance)
p−value threshold
n = 500 ; B_m = −0.3 ; B_x = −0.3 ; B_y = 0
CCPR (0.008)
UMPR (0.091)
UXPR (0.024)
AMPR (0.006)
AXPR (0.008)
UOV (0.003)
AOV (0)
uniform (0.01)
Pr(significance)
p−value threshold

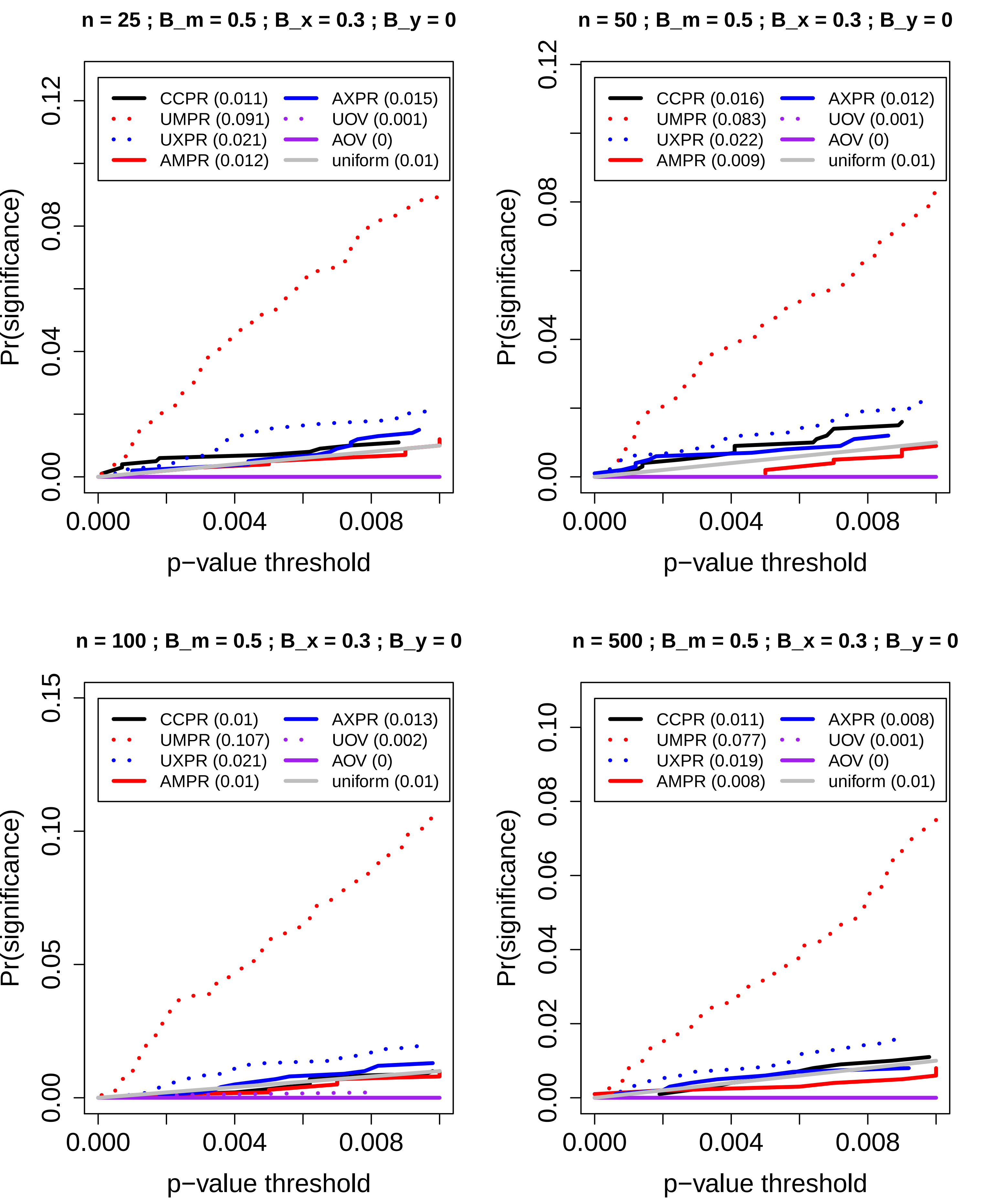

n = 25 ; B_m = 0.5 ; B_x = 0.3 ; B_y = 0
CCPR (0.011)
UMPR (0.091)
UXPR (0.021)
AMPR (0.012)
AXPR (0.015)
UOV (0.001)
AOV (0)
uniform (0.01)
Pr(significance)
p−value threshold
n = 50 ; B_m = 0.5 ; B_x = 0.3 ; B_y = 0
CCPR (0.016)
UMPR (0.083)
UXPR (0.022)
AMPR (0.009)
AXPR (0.012)
UOV (0.001)
AOV (0)
uniform (0.01)
Pr(significance)
p−value threshold
n = 100 ; B_m = 0.5 ; B_x = 0.3 ; B_y = 0
CCPR (0.01)
UMPR (0.107)
UXPR (0.021)
AMPR (0.01)
AXPR (0.013)
UOV (0.002)
AOV (0)
uniform (0.01)
Pr(significance)
p−value threshold
n = 500 ; B_m = 0.5 ; B_x = 0.3 ; B_y = 0
CCPR (0.011)
UMPR (0.077)
UXPR (0.019)
AMPR (0.008)
AXPR (0.008)
UOV (0.001)
AOV (0)
uniform (0.01)
Pr(significance)
p−value threshold

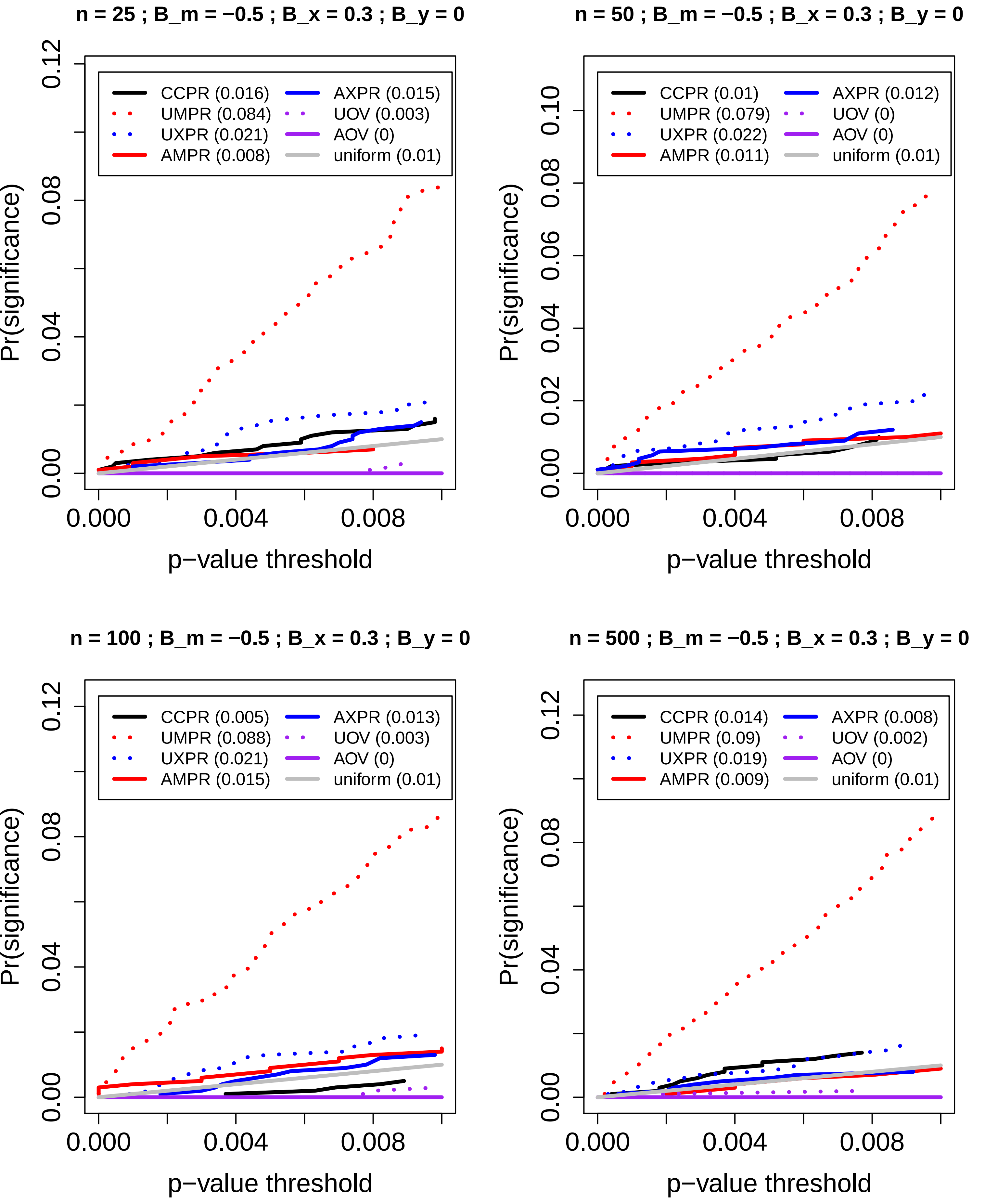
n = 25 ; B_m = −0.5 ; B_x = 0.3 ; B_y = 0
CCPR (0.016)
UMPR (0.084)
UXPR (0.021)
AMPR (0.008)
AXPR (0.015)
UOV (0.003)
AOV (0)
uniform (0.01)
Pr(significance)
0.00
0.04
0.08
0.12
0.000
0.004
0.008
p−value threshold
n = 50 ; B_m = −0.5 ; B_x = 0.3 ; B_y = 0
CCPR (0.01)
UMPR (0.079)
UXPR (0.022)
AMPR (0.011)
AXPR (0.012)
UOV (0)
AOV (0)
uniform (0.01)
Pr(significance)
0.00
0.02
0.04
0.06
0.08
0.10
0.000
0.004
0.008
p−value threshold
n = 100 ; B_m = −0.5 ; B_x = 0.3 ; B_y = 0
CCPR (0.005)
UMPR (0.088)
UXPR (0.021)
AMPR (0.015)
AXPR (0.013)
UOV (0.003)
AOV (0)
uniform (0.01)
Pr(significance)
0.00
0.04
0.08
0.12
0.000
0.004
0.008
p−value threshold
n = 500 ; B_m = −0.5 ; B_x = 0.3 ; B_y = 0
CCPR (0.014)
UMPR (0.09)
UXPR (0.019)
AMPR (0.009)
AXPR (0.008)
UOV (0.002)
AOV (0)
uniform (0.01)
Pr(significance)
0.00
0.04
0.08
0.12
0.000
0.004
0.008
p−value threshold

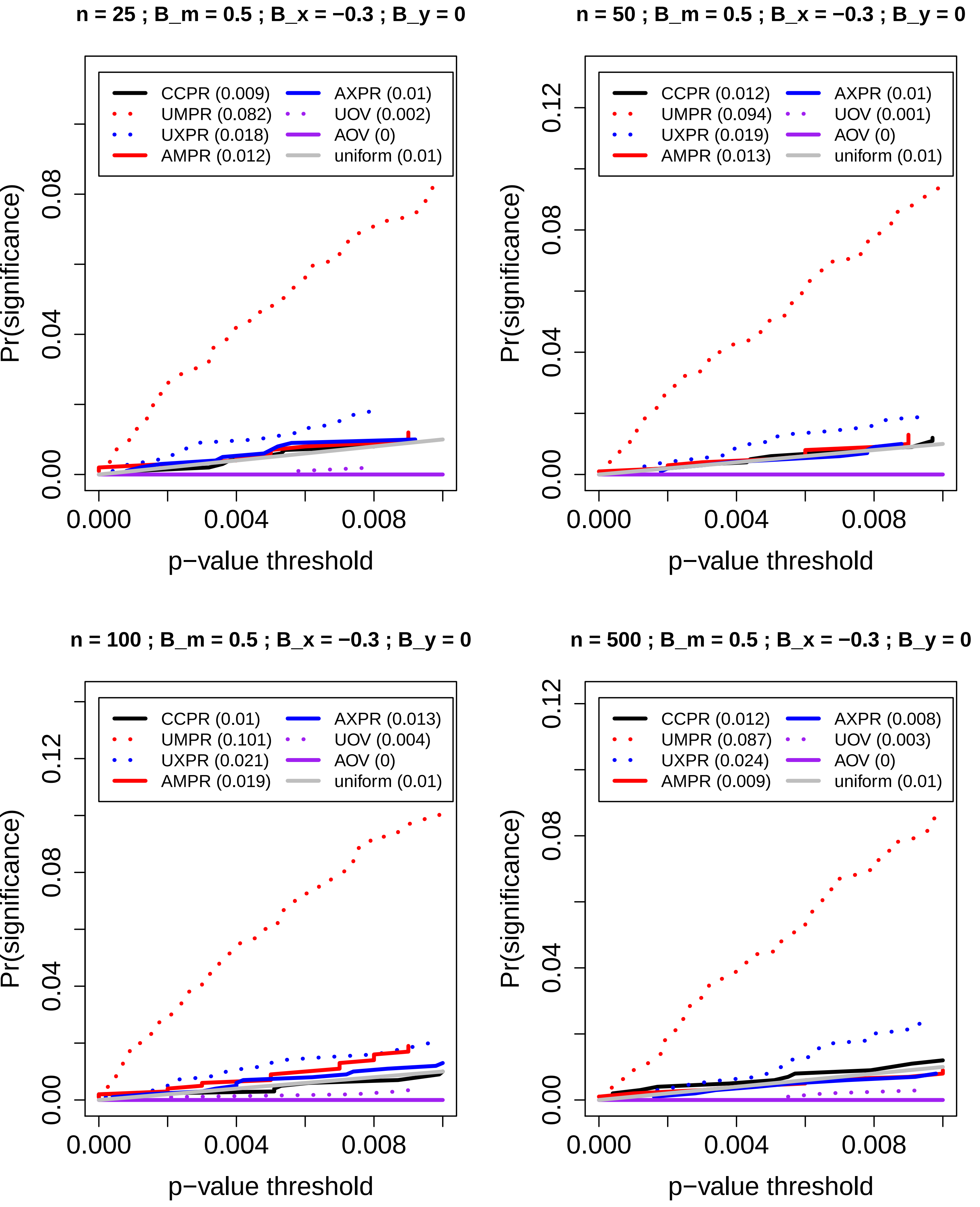

n = 25 ; B_m = 0.5 ; B_x = −0.3 ; B_y = 0
CCPR (0.009)
UMPR (0.082)
UXPR (0.018)
AMPR (0.012)
AXPR (0.01)
UOV (0.002)
AOV (0)
uniform (0.01)
Pr(significance)
0.00
0.04
0.08
0.000
0.004
0.008
p−value threshold
n = 50 ; B_m = 0.5 ; B_x = −0.3 ; B_y = 0
CCPR (0.012)
UMPR (0.094)
UXPR (0.019)
AMPR (0.013)
AXPR (0.01)
UOV (0.001)
AOV (0)
uniform (0.01)
Pr(significance)
0.00
0.04
0.08
0.12
0.000
0.004
0.008
p−value threshold
n = 100 ; B_m = 0.5 ; B_x = −0.3 ; B_y = 0
CCPR (0.01)
UMPR (0.101)
UXPR (0.021)
AMPR (0.019)
AXPR (0.013)
UOV (0.004)
AOV (0)
uniform (0.01)
Pr(significance)
0.00
0.04
0.08
0.12
0.000
0.004
0.008
p−value threshold
n = 500 ; B_m = 0.5 ; B_x = −0.3 ; B_y = 0
CCPR (0.012)
UMPR (0.087)
UXPR (0.024)
AMPR (0.009)
AXPR (0.008)
UOV (0.003)
AOV (0)
uniform (0.01)
Pr(significance)
0.00
0.04
0.08
0.12
0.000
0.004
0.008
p−value threshold

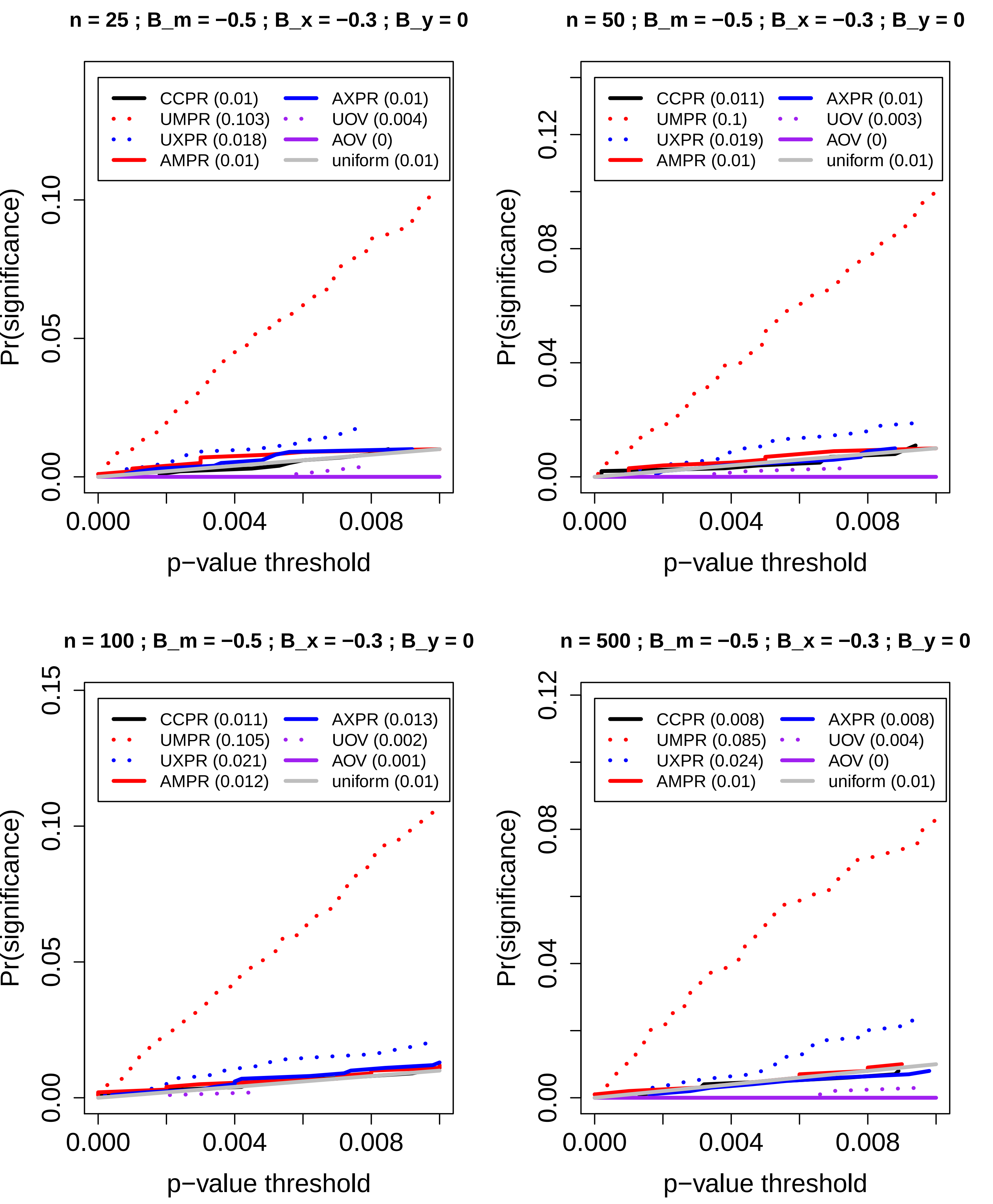

n = 25 ; B_m = −0.5 ; B_x = −0.3 ; B_y = 0
CCPR (0.01)
UMPR (0.103)
UXPR (0.018)
AMPR (0.01)
AXPR (0.01)
UOV (0.004)
AOV (0)
uniform (0.01)
Pr(significance)
0.00
0.05
0.10
0.000
0.004
0.008
p−value threshold
n = 50 ; B_m = −0.5 ; B_x = −0.3 ; B_y = 0
CCPR (0.011)
UMPR (0.1)
UXPR (0.019)
AMPR (0.01)
AXPR (0.01)
UOV (0.003)
AOV (0)
uniform (0.01)
Pr(significance)
0.00
0.04
0.08
0.12
0.000
0.004
0.008
p−value threshold
n = 100 ; B_m = −0.5 ; B_x = −0.3 ; B_y = 0
CCPR (0.011)
UMPR (0.105)
UXPR (0.021)
AMPR (0.012)
AXPR (0.013)
UOV (0.002)
AOV (0.001)
uniform (0.01)
Pr(significance)
0.00
0.05
0.10
0.15
0.000
0.004
0.008
p−value threshold
n = 500 ; B_m = −0.5 ; B_x = −0.3 ; B_y = 0
CCPR (0.008)
UMPR (0.085)
UXPR (0.024)
AMPR (0.01)
AXPR (0.008)
UOV (0.004)
AOV (0)
uniform (0.01)
Pr(significance)
0.00
0.04
0.08
0.12
0.000
0.004
0.008
p−value threshold

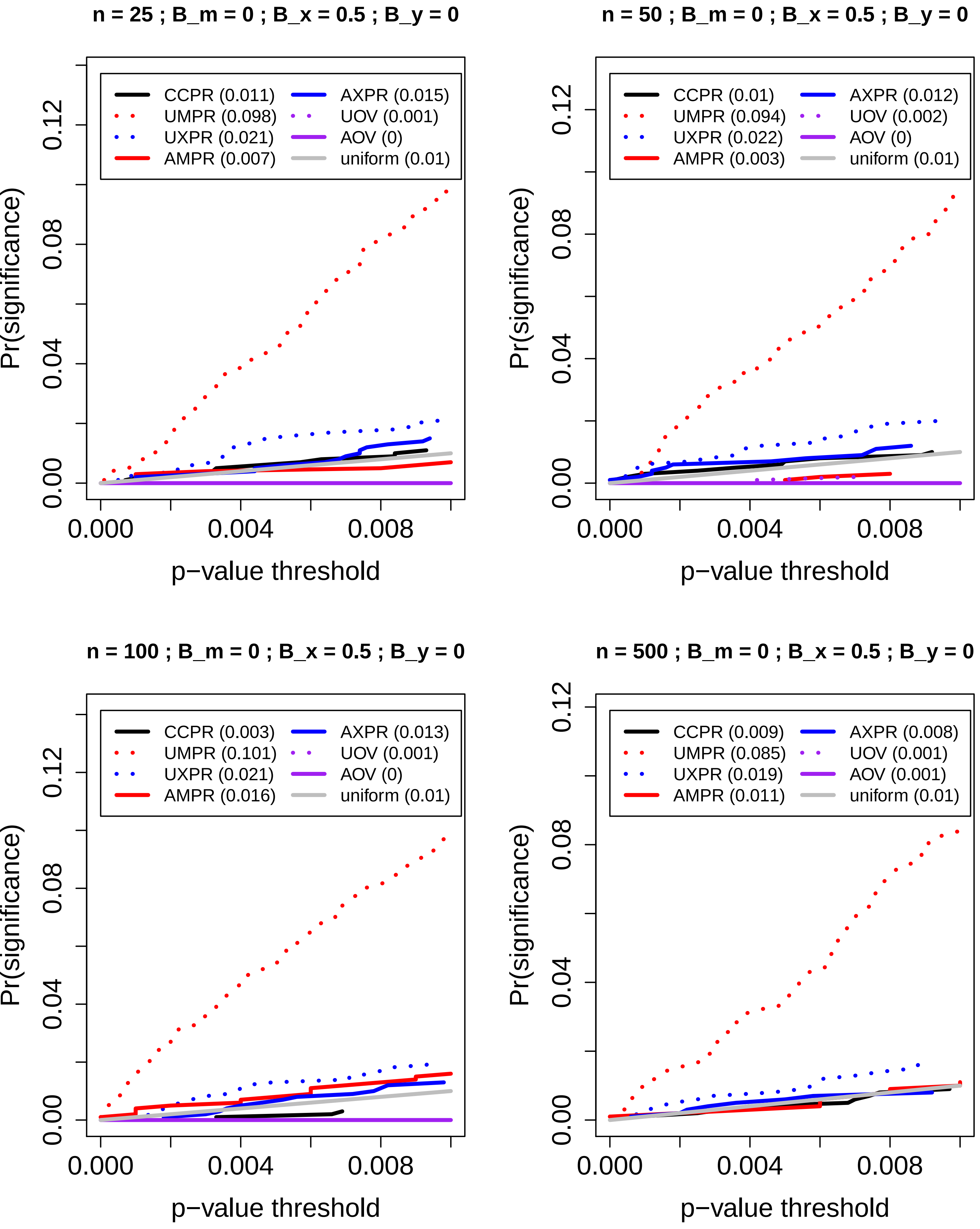

n = 25 ; B_m = 0 ; B_x = 0.5 ; B_y = 0
CCPR (0.011)
UMPR (0.098)
UXPR (0.021)
AMPR (0.007)
AXPR (0.015)
UOV (0.001)
AOV (0)
uniform (0.01)
n = 50 ; B_m = 0 ; B_x = 0.5 ; B_y = 0
CCPR (0.01)
UMPR (0.094)
UXPR (0.022)
AMPR (0.003)
AXPR (0.012)
UOV (0.002)
AOV (0)
uniform (0.01)
n = 100 ; B_m = 0 ; B_x = 0.5 ; B_y = 0
CCPR (0.003)
UMPR (0.101)
UXPR (0.021)
AMPR (0.016)
AXPR (0.013)
UOV (0.001)
AOV (0)
uniform (0.01)
n = 500 ; B_m = 0 ; B_x = 0.5 ; B_y = 0
CCPR (0.009)
UMPR (0.085)
UXPR (0.019)
AMPR (0.011)
AXPR (0.008)
UOV (0.001)
AOV (0.001)
uniform (0.01)
Pr(significance)
0.00
0.04
0.08
0.12
p−value threshold
0.000
0.004
0.008

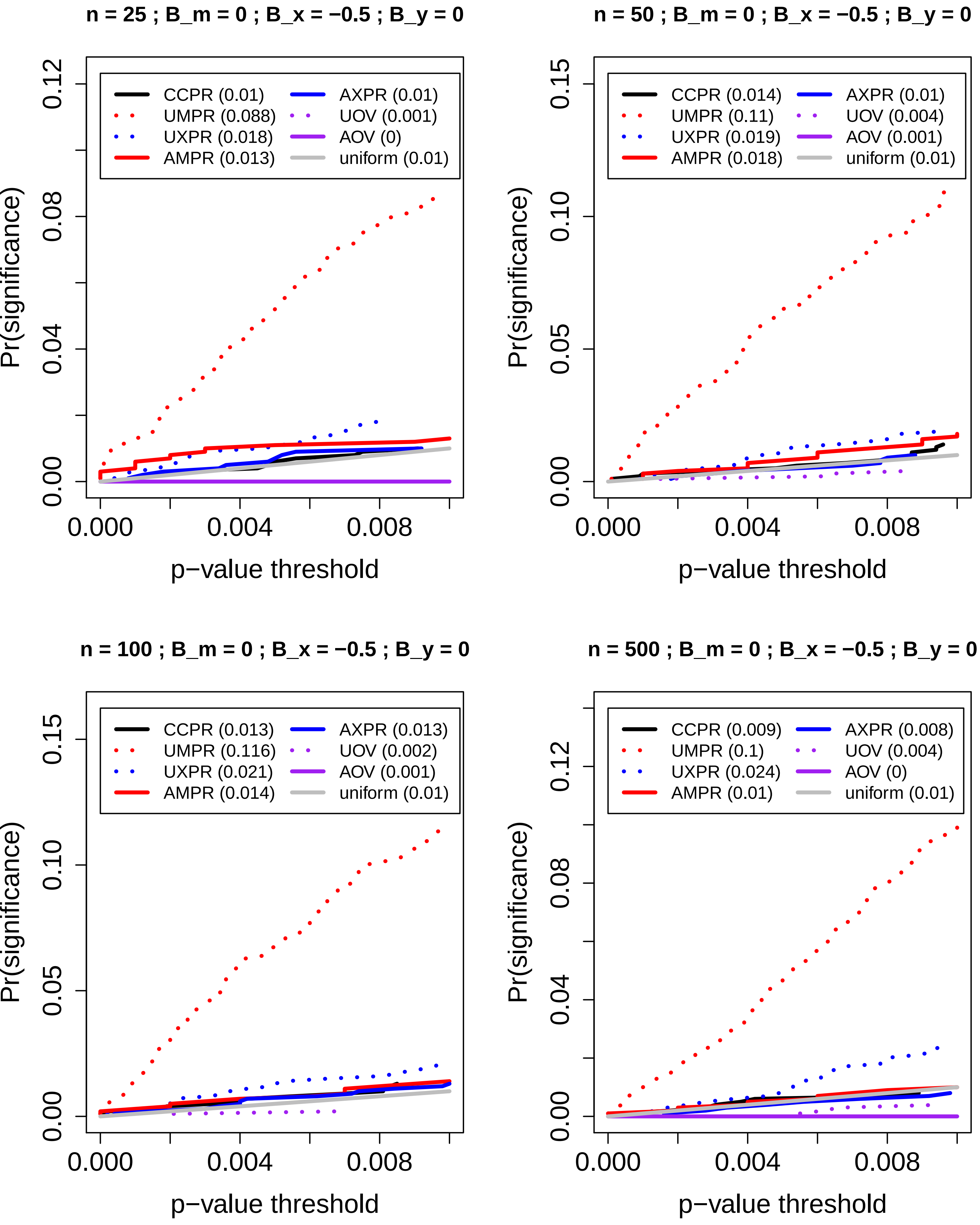
n = 25 ; B_m = 0 ; B_x = −0.5 ; B_y = 0
CCPR (0.01)
UMPR (0.088)
UXPR (0.018)
AMPR (0.013)
AXPR (0.01)
UOV (0.001)
AOV (0)
uniform (0.01)
Pr(significance)
p−value threshold
n = 50 ; B_m = 0 ; B_x = −0.5 ; B_y = 0
CCPR (0.014)
UMPR (0.11)
UXPR (0.019)
AMPR (0.018)
AXPR (0.01)
UOV (0.004)
AOV (0.001)
uniform (0.01)
Pr(significance)
p−value threshold
n = 100 ; B_m = 0 ; B_x = −0.5 ; B_y = 0
CCPR (0.013)
UMPR (0.116)
UXPR (0.021)
AMPR (0.014)
AXPR (0.013)
UOV (0.002)
AOV (0.001)
uniform (0.01)
Pr(significance)
p−value threshold
n = 500 ; B_m = 0 ; B_x = −0.5 ; B_y = 0
CCPR (0.009)
UMPR (0.1)
UXPR (0.024)
AMPR (0.01)
AXPR (0.008)
UOV (0.004)
AOV (0)
uniform (0.01)
Pr(significance)
p−value threshold

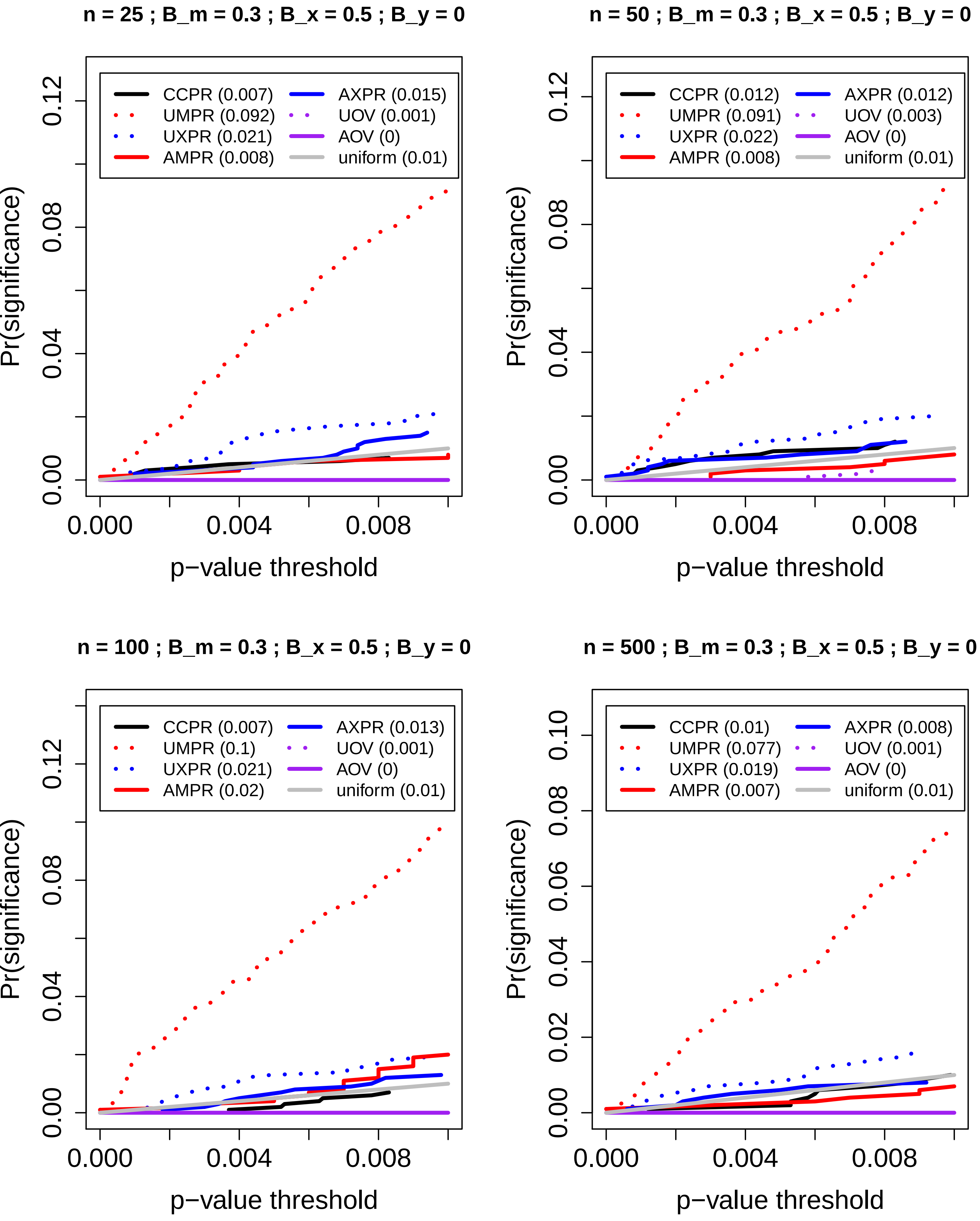

n = 25 ; B_m = 0.3 ; B_x = 0.5 ; B_y = 0
CCPR (0.007)
UMPR (0.092)
UXPR (0.021)
AMPR (0.008)
AXPR (0.015)
UOV (0.001)
AOV (0)
uniform (0.01)
n = 50 ; B_m = 0.3 ; B_x = 0.5 ; B_y = 0
CCPR (0.012)
UMPR (0.091)
UXPR (0.022)
AMPR (0.008)
AXPR (0.012)
UOV (0.003)
AOV (0)
uniform (0.01)
n = 100 ; B_m = 0.3 ; B_x = 0.5 ; B_y = 0
CCPR (0.007)
UMPR (0.1)
UXPR (0.021)
AMPR (0.02)
AXPR (0.013)
UOV (0.001)
AOV (0)
uniform (0.01)
n = 500 ; B_m = 0.3 ; B_x = 0.5 ; B_y = 0
CCPR (0.01)
UMPR (0.077)
UXPR (0.019)
AMPR (0.007)
AXPR (0.008)
UOV (0.001)
AOV (0)
uniform (0.01)
Pr(significance)
p−value threshold
0.000
0.004
0.008
0.00
0.02
0.04
0.06
0.08
0.10
0.12

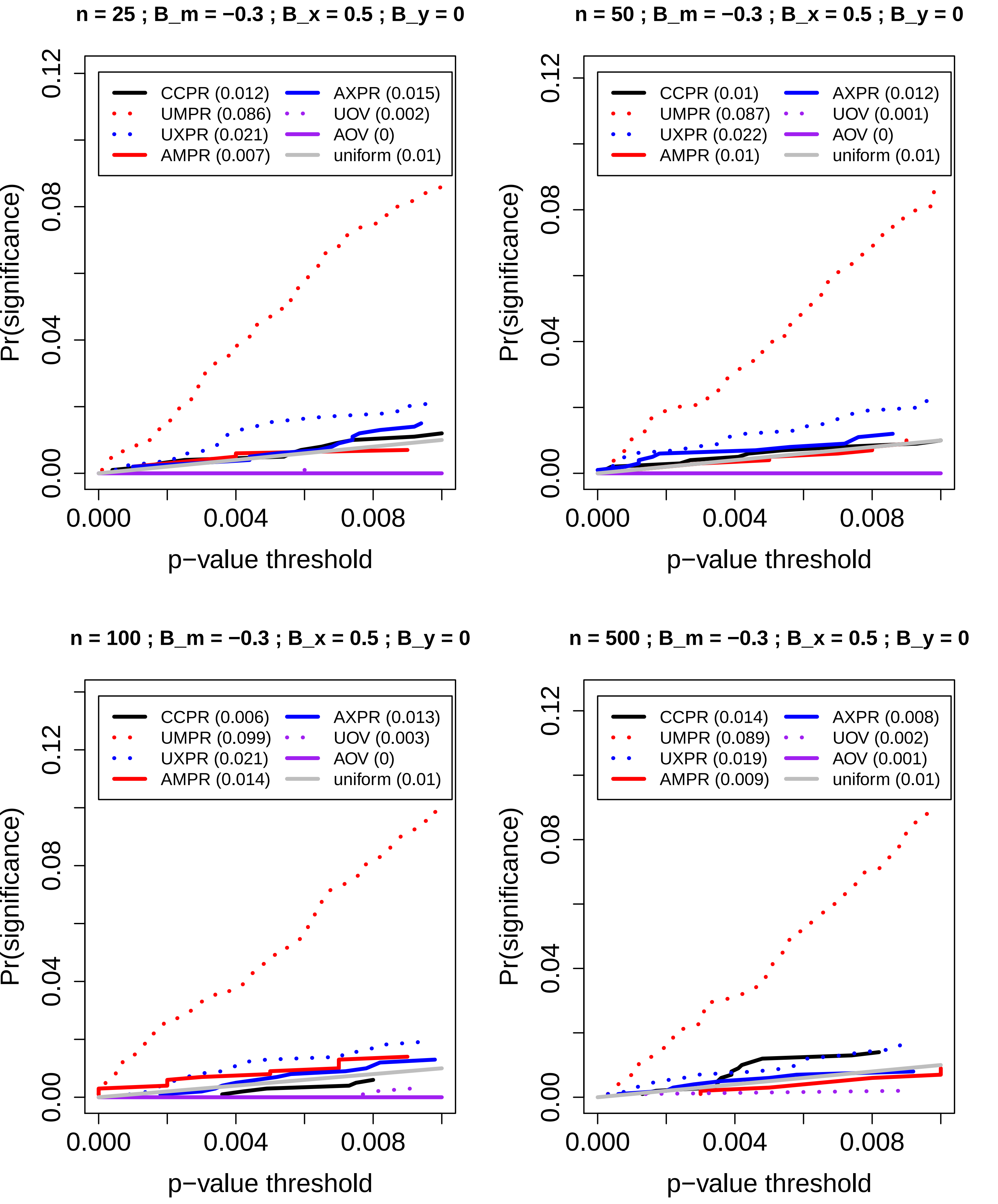
n = 25 ; B_m = −0.3 ; B_x = 0.5 ; B_y = 0
CCPR (0.012)
UMPR (0.086)
UXPR (0.021)
AMPR (0.007)
AXPR (0.015)
UOV (0.002)
AOV (0)
uniform (0.01)
Pr(significance)
p−value threshold
n = 50 ; B_m = −0.3 ; B_x = 0.5 ; B_y = 0
CCPR (0.01)
UMPR (0.087)
UXPR (0.022)
AMPR (0.01)
AXPR (0.012)
UOV (0.001)
AOV (0)
uniform (0.01)
Pr(significance)
p−value threshold
n = 100 ; B_m = −0.3 ; B_x = 0.5 ; B_y = 0
CCPR (0.006)
UMPR (0.099)
UXPR (0.021)
AMPR (0.014)
AXPR (0.013)
UOV (0.003)
AOV (0)
uniform (0.01)
Pr(significance)
p−value threshold
n = 500 ; B_m = −0.3 ; B_x = 0.5 ; B_y = 0
CCPR (0.014)
UMPR (0.089)
UXPR (0.019)
AMPR (0.009)
AXPR (0.008)
UOV (0.002)
AOV (0.001)
uniform (0.01)
Pr(significance)
p−value threshold

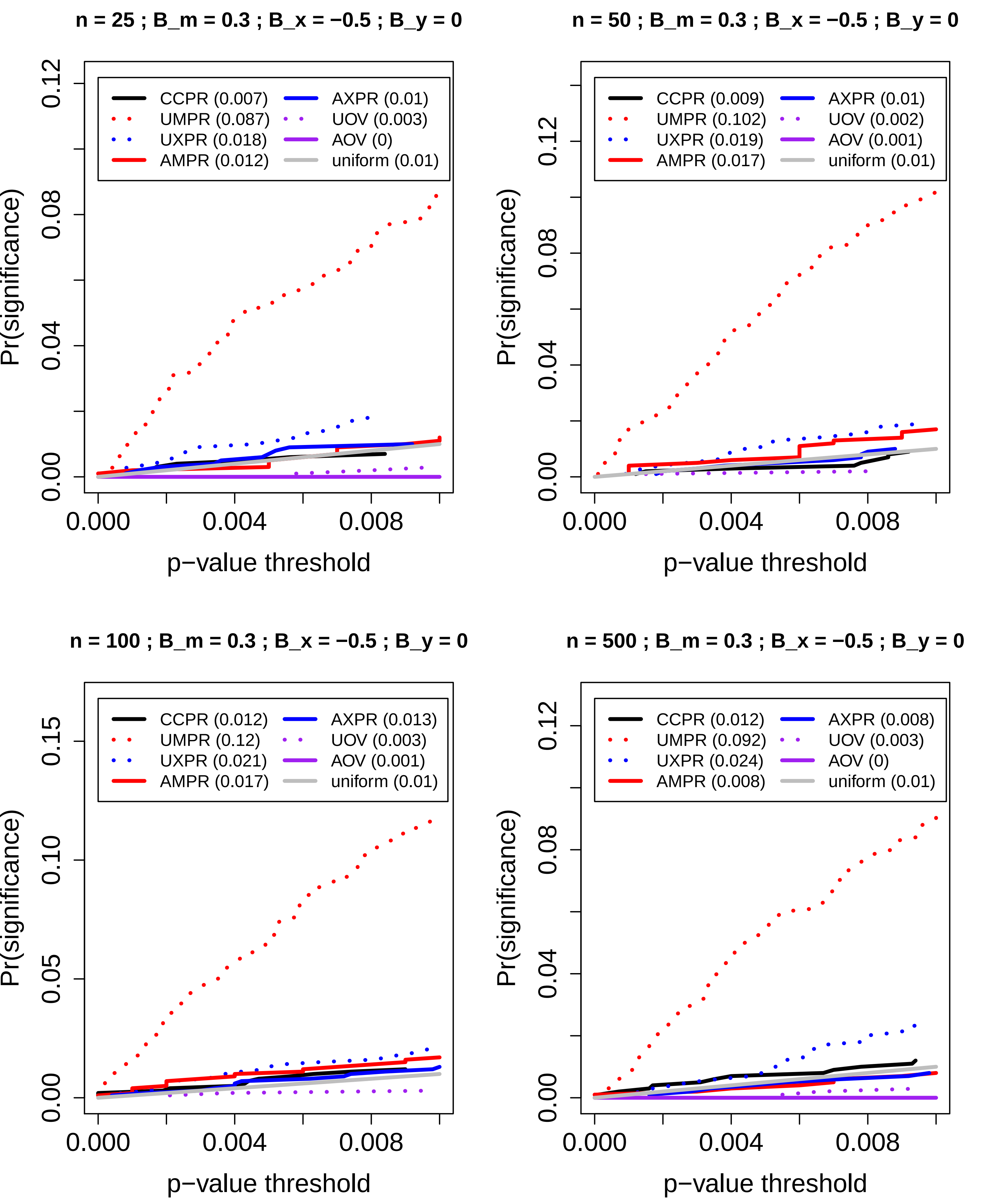
n = 25 ; B_m = 0.3 ; B_x = −0.5 ; B_y = 0
CCPR (0.007)
UMPR (0.087)
UXPR (0.018)
AMPR (0.012)
AXPR (0.01)
UOV (0.003)
AOV (0)
uniform (0.01)
n = 50 ; B_m = 0.3 ; B_x = −0.5 ; B_y = 0
CCPR (0.009)
UMPR (0.102)
UXPR (0.019)
AMPR (0.017)
AXPR (0.01)
UOV (0.002)
AOV (0.001)
uniform (0.01)
n = 100 ; B_m = 0.3 ; B_x = −0.5 ; B_y = 0
CCPR (0.012)
UMPR (0.12)
UXPR (0.021)
AMPR (0.017)
AXPR (0.013)
UOV (0.003)
AOV (0.001)
uniform (0.01)
n = 500 ; B_m = 0.3 ; B_x = −0.5 ; B_y = 0
CCPR (0.012)
UMPR (0.092)
UXPR (0.024)
AMPR (0.008)
AXPR (0.008)
UOV (0.003)
AOV (0)
uniform (0.01)
Pr(significance)
p−value threshold
0.00
0.04
0.08
0.12
0.05
0.10
0.15
0.000
0.004
0.008

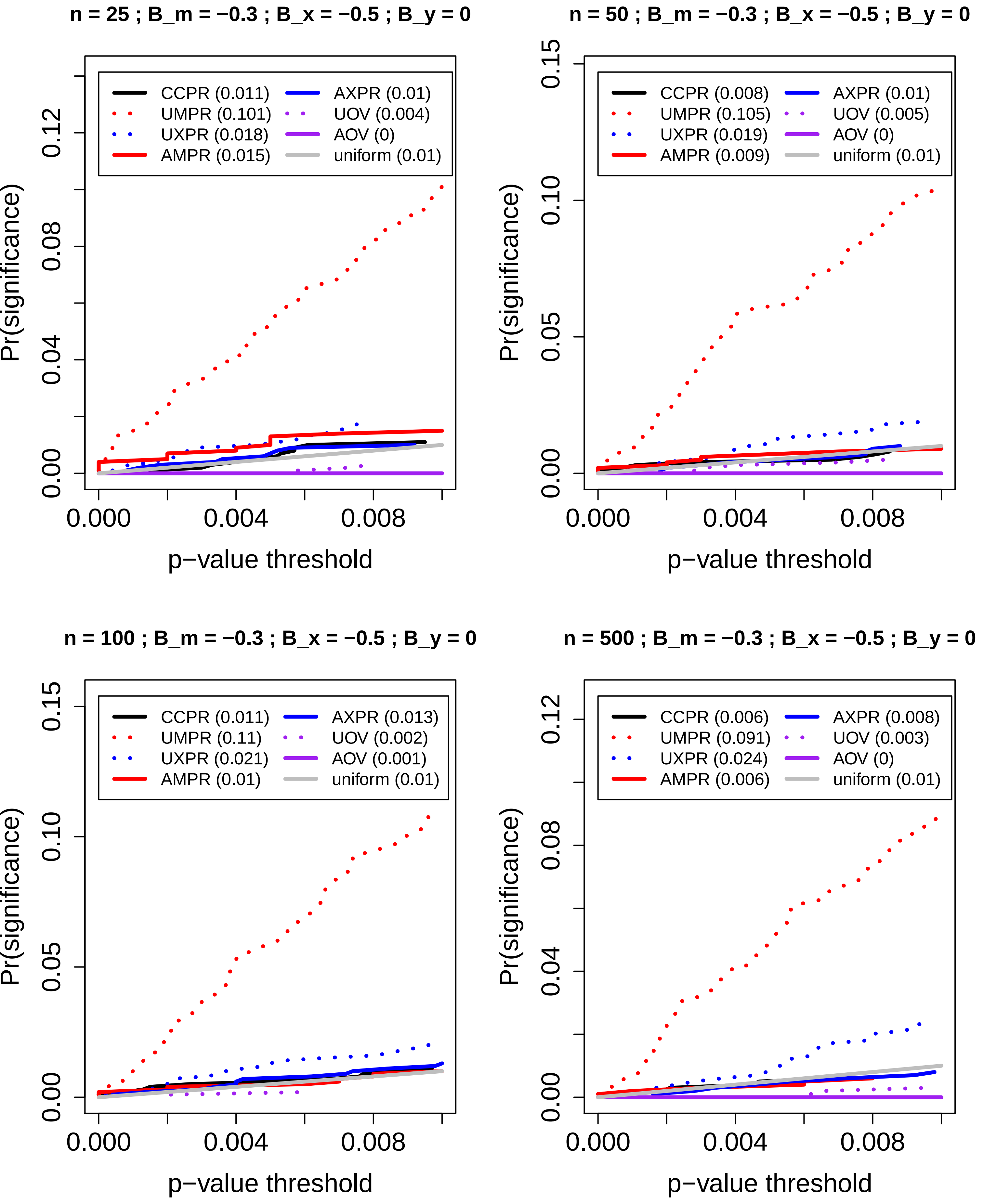

n = 25 ; B_m = −0.3 ; B_x = −0.5 ; B_y = 0
CCPR (0.011)
UMPR (0.101)
UXPR (0.018)
AMPR (0.015)
AXPR (0.01)
UOV (0.004)
AOV (0)
uniform (0.01)
Pr(significance)
p−value threshold
n = 50 ; B_m = −0.3 ; B_x = −0.5 ; B_y = 0
CCPR (0.008)
UMPR (0.105)
UXPR (0.019)
AMPR (0.009)
AXPR (0.01)
UOV (0.005)
AOV (0)
uniform (0.01)
Pr(significance)
p−value threshold
n = 100 ; B_m = −0.3 ; B_x = −0.5 ; B_y = 0
CCPR (0.011)
UMPR (0.11)
UXPR (0.021)
AMPR (0.01)
AXPR (0.013)
UOV (0.002)
AOV (0.001)
uniform (0.01)
Pr(significance)
p−value threshold
n = 500 ; B_m = −0.3 ; B_x = −0.5 ; B_y = 0
CCPR (0.006)
UMPR (0.091)
UXPR (0.024)
AMPR (0.006)
AXPR (0.008)
UOV (0.003)
AOV (0)
uniform (0.01)
Pr(significance)
p−value threshold

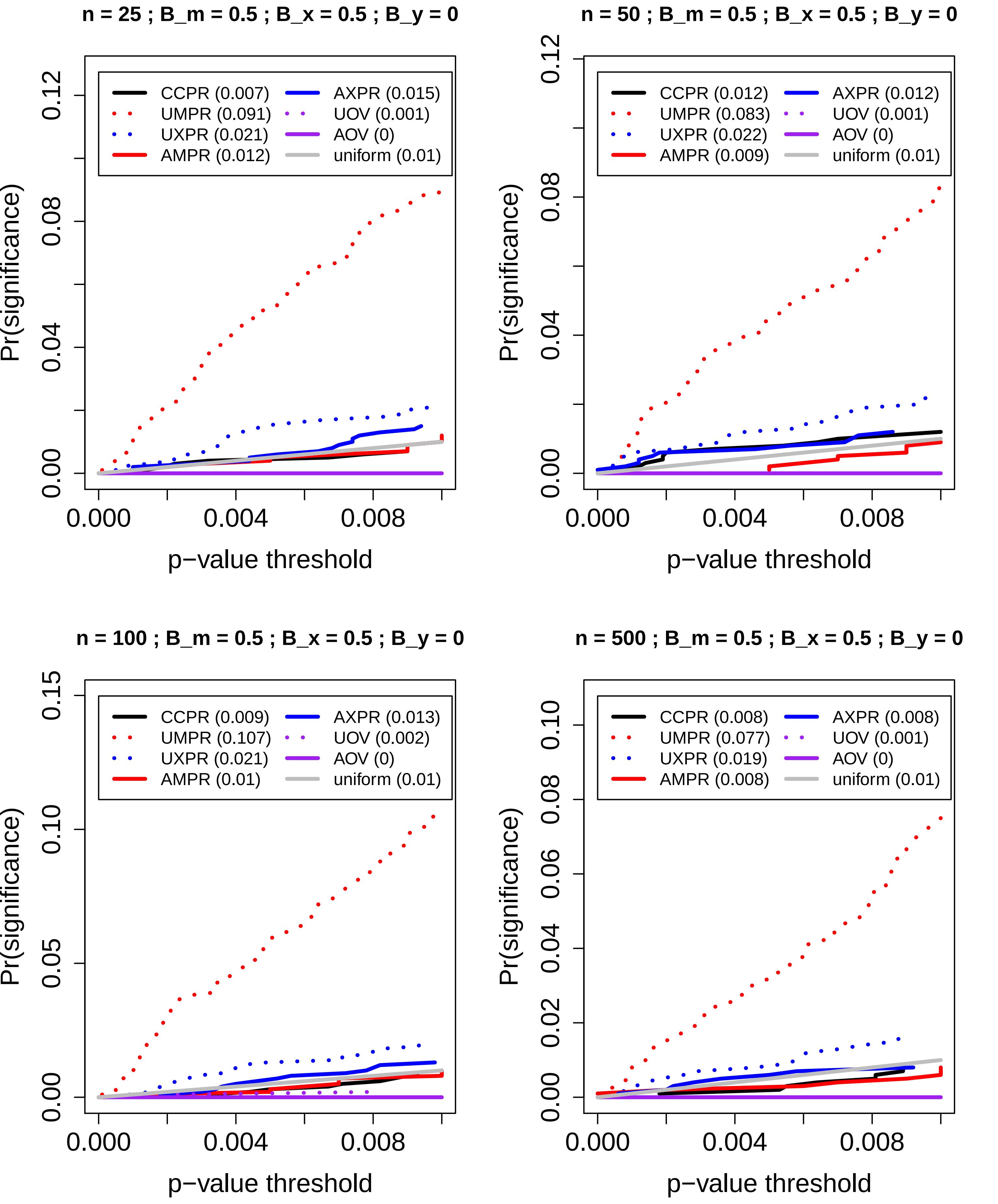

n = 25 ; B_m = 0.5 ; B_x = 0.5 ; B_y = 0
CCPR (0.007)
UMPR (0.091)
UXPR (0.021)
AMPR (0.012)
AXPR (0.015)
UOV (0.001)
AOV (0)
uniform (0.01)
Pr(significance)
p−value threshold
0.00
0.04
0.08
0.12
0.000
0.004
0.008
n = 50 ; B_m = 0.5 ; B_x = 0.5 ; B_y = 0
CCPR (0.012)
UMPR (0.083)
UXPR (0.022)
AMPR (0.009)
AXPR (0.012)
UOV (0.001)
AOV (0)
uniform (0.01)
n = 100 ; B_m = 0.5 ; B_x = 0.5 ; B_y = 0
CCPR (0.009)
UMPR (0.107)
UXPR (0.021)
AMPR (0.01)
AXPR (0.013)
UOV (0.002)
AOV (0)
uniform (0.01)
0.00
0.05
0.10
0.15
n = 500 ; B_m = 0.5 ; B_x = 0.5 ; B_y = 0
CCPR (0.008)
UMPR (0.077)
UXPR (0.019)
AMPR (0.008)
AXPR (0.008)
UOV (0.001)
AOV (0)
uniform (0.01)
0.00
0.02
0.04
0.06
0.08
0.10

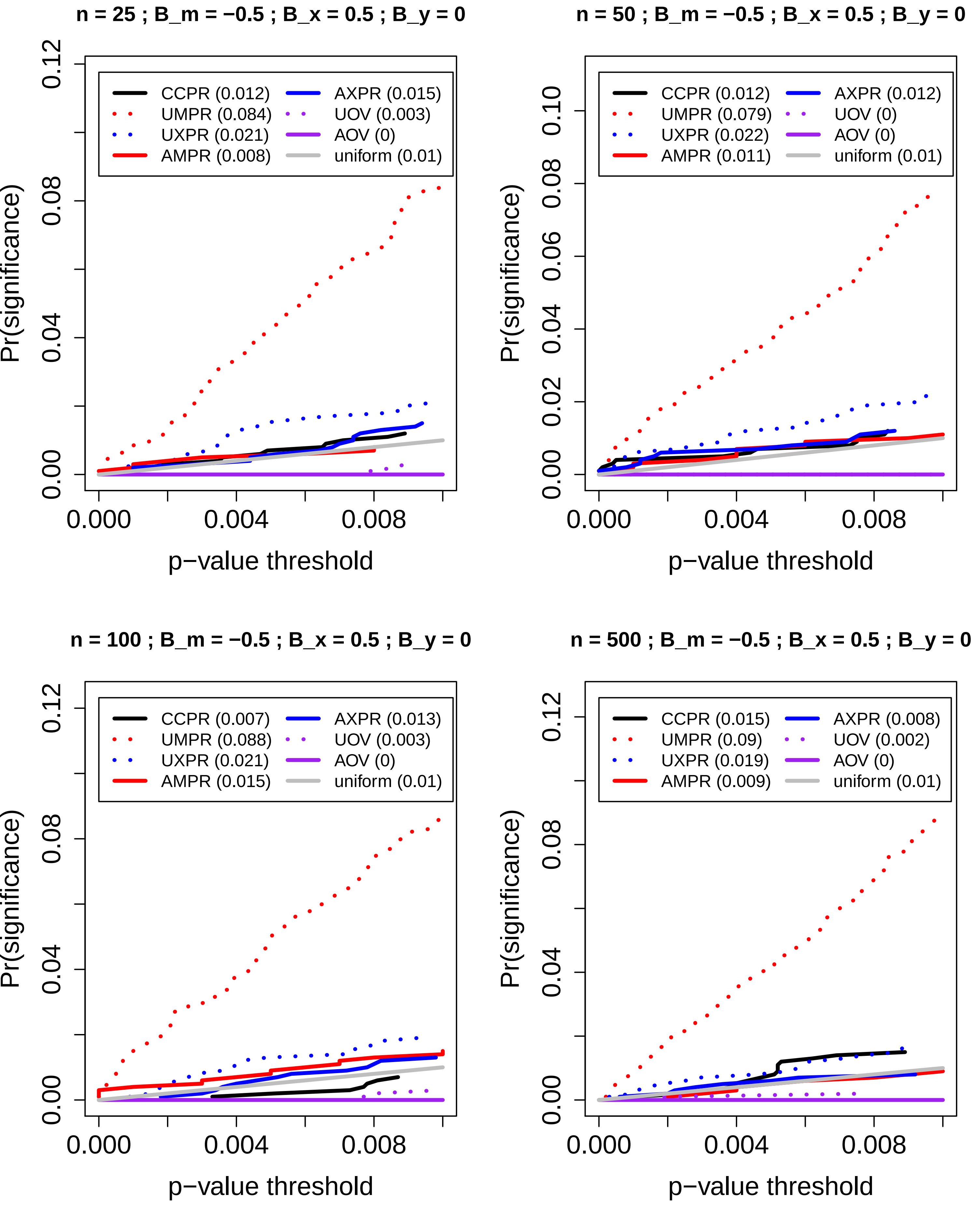

n = 25 ; B_m = −0.5 ; B_x = 0.5 ; B_y = 0
CCPR (0.012)
UMPR (0.084)
UXPR (0.021)
AMPR (0.008)
AXPR (0.015)
UOV (0.003)
AOV (0)
uniform (0.01)
n = 50 ; B_m = −0.5 ; B_x = 0.5 ; B_y = 0
CCPR (0.012)
UMPR (0.079)
UXPR (0.022)
AMPR (0.011)
AXPR (0.012)
UOV (0)
AOV (0)
uniform (0.01)
n = 100 ; B_m = −0.5 ; B_x = 0.5 ; B_y = 0
CCPR (0.007)
UMPR (0.088)
UXPR (0.021)
AMPR (0.015)
AXPR (0.013)
UOV (0.003)
AOV (0)
uniform (0.01)
n = 500 ; B_m = −0.5 ; B_x = 0.5 ; B_y = 0
CCPR (0.015)
UMPR (0.09)
UXPR (0.019)
AMPR (0.009)
AXPR (0.008)
UOV (0.002)
AOV (0)
uniform (0.01)
Pr(significance)
p−value threshold

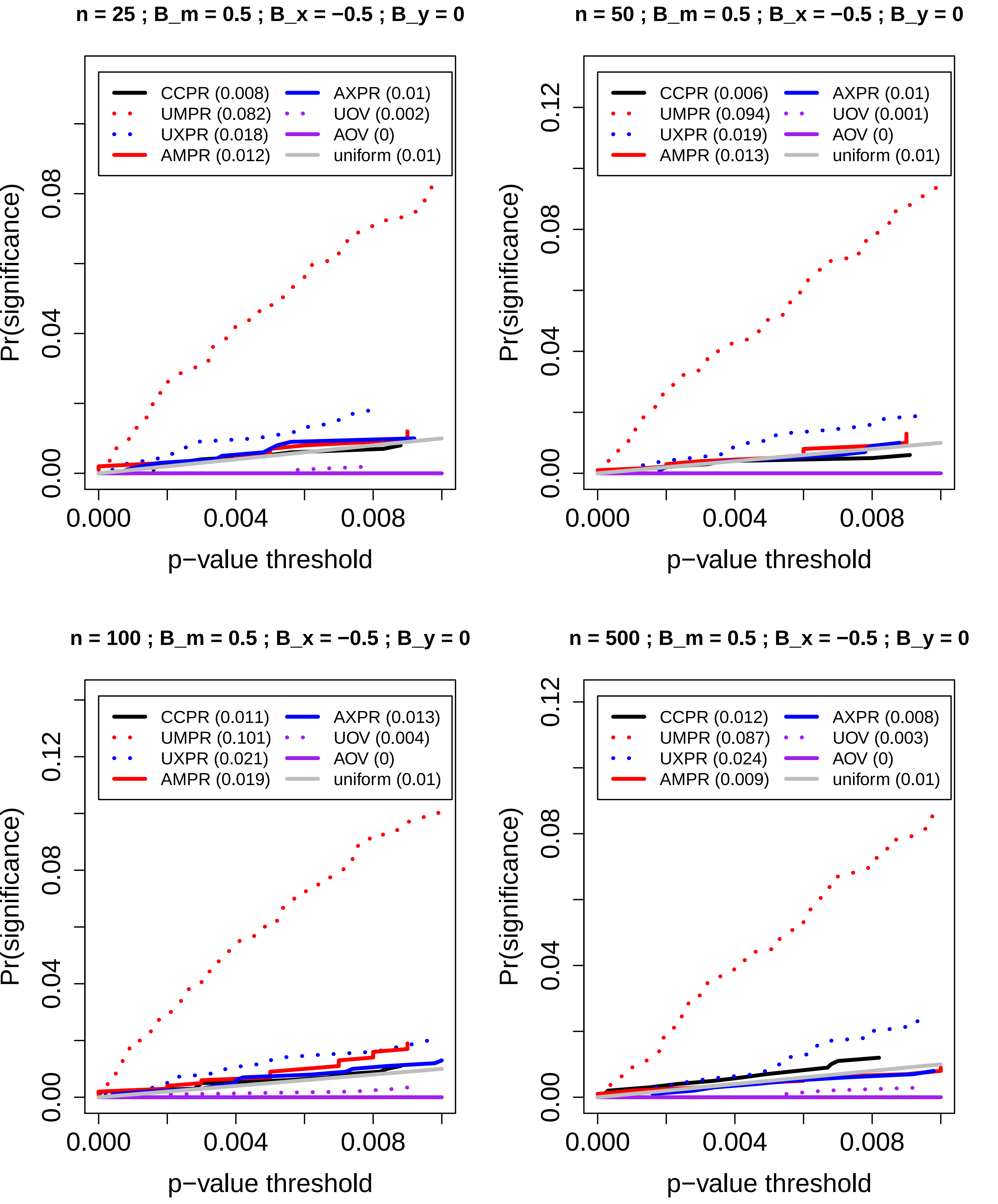

n = 25 ; B_m = 0.5 ; B_x = −0.5 ; B_y = 0
CCPR (0.008)
UMPR (0.082)
UXPR (0.018)
AMPR (0.012)
AXPR (0.01)
UOV (0.002)
AOV (0)
uniform (0.01)
Pr(significance)
p−value threshold
n = 50 ; B_m = 0.5 ; B_x = −0.5 ; B_y = 0
CCPR (0.006)
UMPR (0.094)
UXPR (0.019)
AMPR (0.013)
AXPR (0.01)
UOV (0.001)
AOV (0)
uniform (0.01)
Pr(significance)
p−value threshold
n = 100 ; B_m = 0.5 ; B_x = −0.5 ; B_y = 0
CCPR (0.011)
UMPR (0.101)
UXPR (0.021)
AMPR (0.019)
AXPR (0.013)
UOV (0.004)
AOV (0)
uniform (0.01)
Pr(significance)
p−value threshold
n = 500 ; B_m = 0.5 ; B_x = −0.5 ; B_y = 0
CCPR (0.012)
UMPR (0.087)
UXPR (0.024)
AMPR (0.009)
AXPR (0.008)
UOV (0.003)
AOV (0)
uniform (0.01)
Pr(significance)
p−value threshold

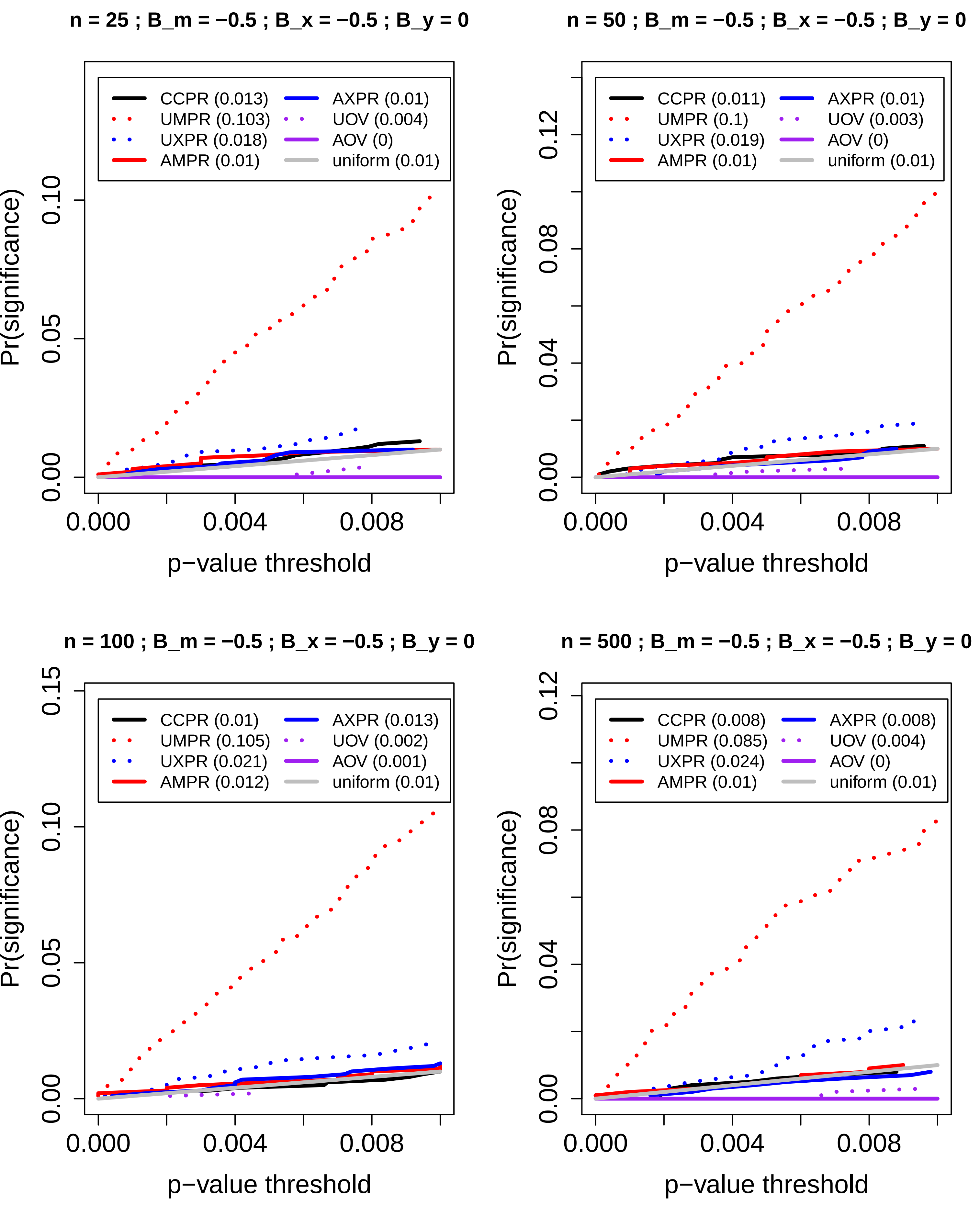

n = 25 ; B_m = −0.5 ; B_x = −0.5 ; B_y = 0
CCPR (0.013)
UMPR (0.103)
UXPR (0.018)
AMPR (0.01)
AXPR (0.01)
UOV (0.004)
AOV (0)
uniform (0.01)
n = 50 ; B_m = −0.5 ; B_x = −0.5 ; B_y = 0
CCPR (0.011)
UMPR (0.1)
UXPR (0.019)
AMPR (0.01)
AXPR (0.01)
UOV (0.003)
AOV (0)
uniform (0.01)
n = 100 ; B_m = −0.5 ; B_x = −0.5 ; B_y = 0
CCPR (0.01)
UMPR (0.105)
UXPR (0.021)
AMPR (0.012)
AXPR (0.013)
UOV (0.002)
AOV (0.001)
uniform (0.01)
n = 500 ; B_m = −0.5 ; B_x = −0.5 ; B_y = 0
CCPR (0.008)
UMPR (0.085)
UXPR (0.024)
AMPR (0.01)
AXPR (0.008)
UOV (0.004)
AOV (0)
uniform (0.01)
Pr(significance)
p−value threshold
0.000
0.004
0.008
0.00
0.05
0.10
0.15
0.04
0.08
0.12

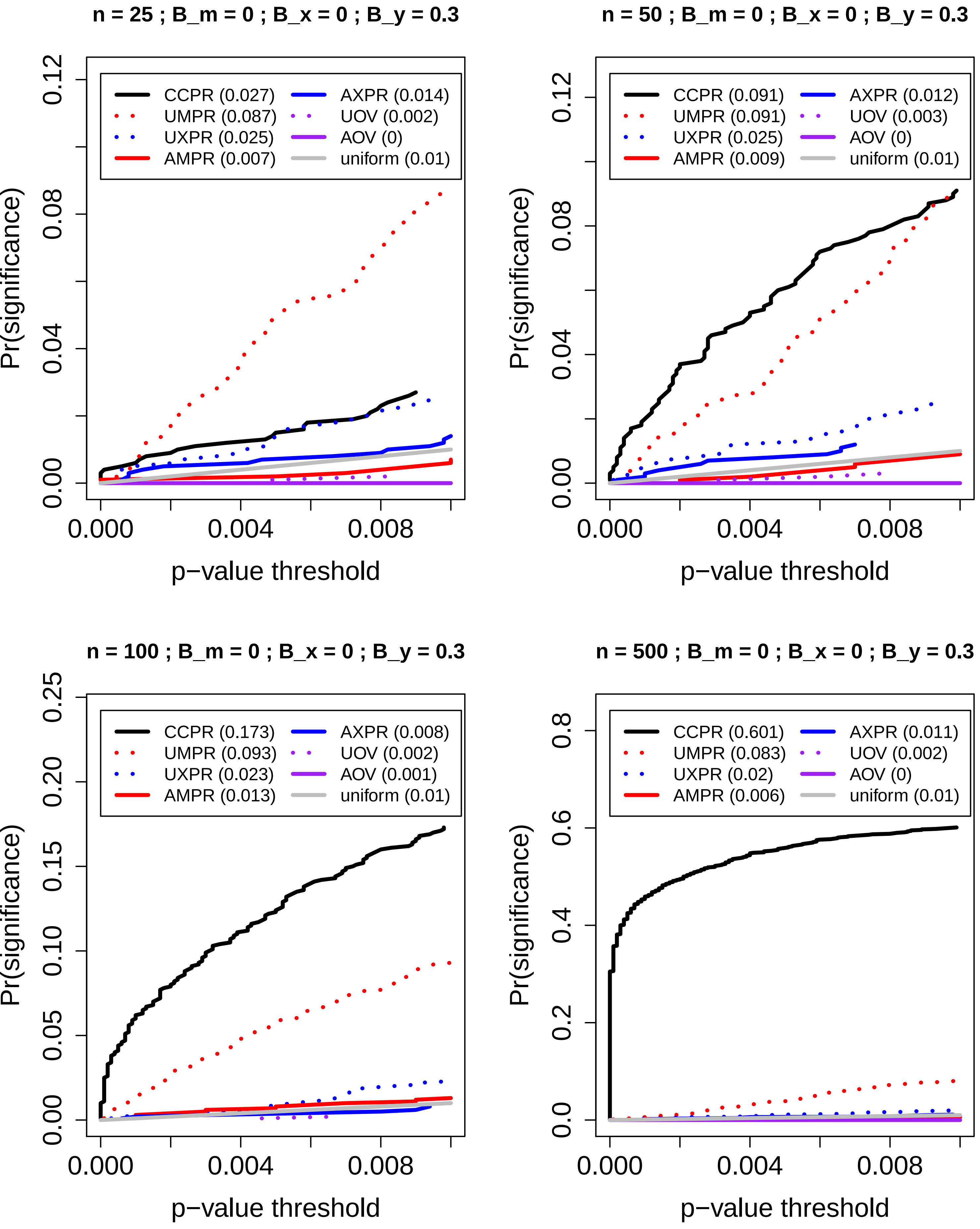

n = 25 ; B_m = 0 ; B_x = 0 ; B_y = 0.3
CCPR (0.027)
UMPR (0.087)
UXPR (0.025)
AMPR (0.007)
AXPR (0.014)
UOV (0.002)
AOV (0)
uniform (0.01)
Pr(significance)
0.00
0.04
0.08
0.12
0.000
0.004
0.008
p−value threshold
n = 50 ; B_m = 0 ; B_x = 0 ; B_y = 0.3
CCPR (0.091)
UMPR (0.091)
UXPR (0.025)
AMPR (0.009)
AXPR (0.012)
UOV (0.003)
AOV (0)
uniform (0.01)
Pr(significance)
0.00
0.04
0.08
0.12
0.000
0.004
0.008
p−value threshold
n = 100 ; B_m = 0 ; B_x = 0 ; B_y = 0.3
CCPR (0.173)
UMPR (0.093)
UXPR (0.023)
AMPR (0.013)
AXPR (0.008)
UOV (0.002)
AOV (0.001)
uniform (0.01)
Pr(significance)
0.00
0.05
0.10
0.15
0.20
0.25
0.000
0.004
0.008
p−value threshold
n = 500 ; B_m = 0 ; B_x = 0 ; B_y = 0.3
CCPR (0.601)
UMPR (0.083)
UXPR (0.02)
AMPR (0.006)
AXPR (0.011)
UOV (0.002)
AOV (0)
uniform (0.01)
Pr(significance)
0.0
0.2
0.4
0.6
0.8
0.000
0.004
0.008
p−value threshold

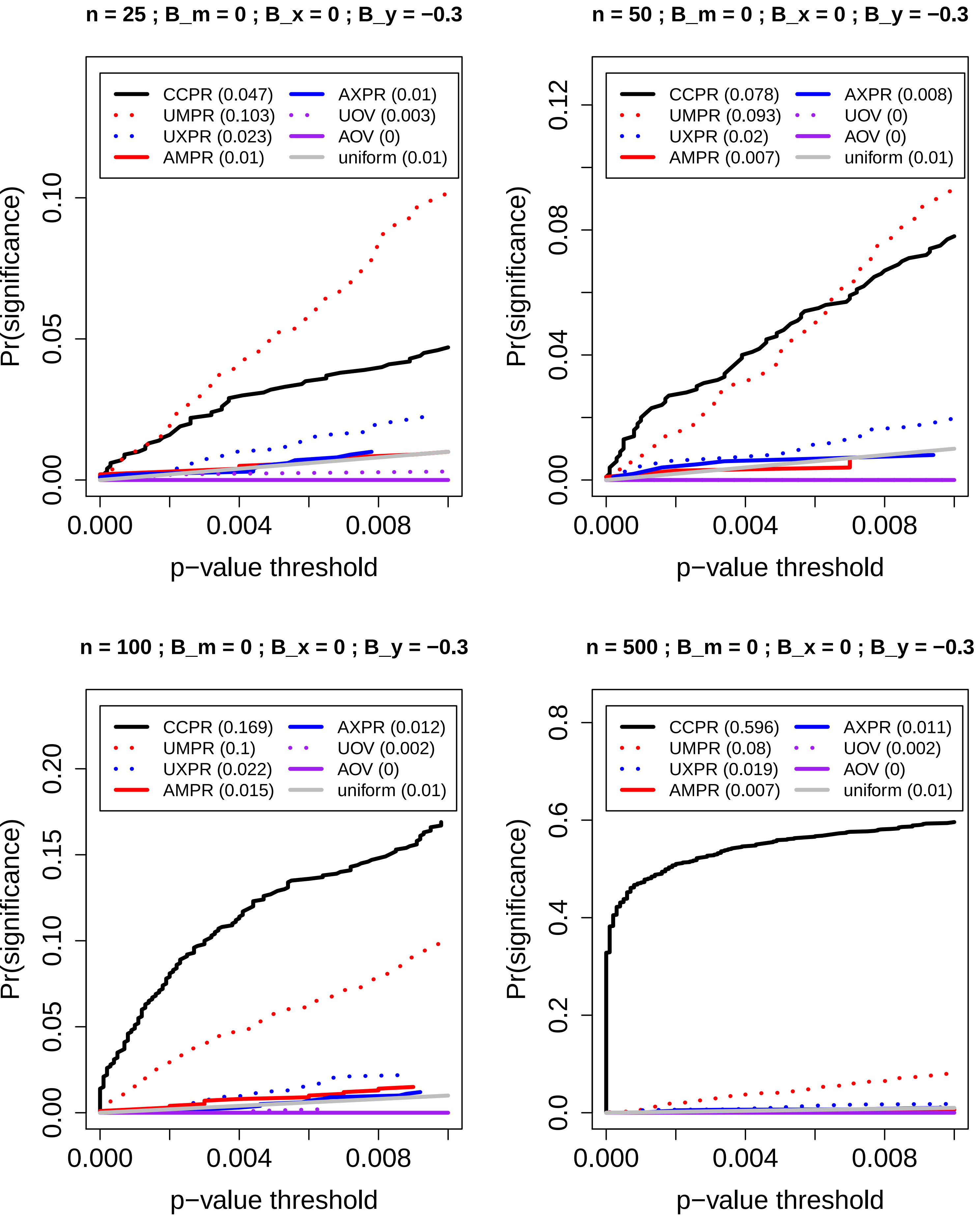
n = 25 ; B_m = 0 ; B_x = 0 ; B_y = −0.3
CCPR (0.047)
UMPR (0.103)
UXPR (0.023)
AMPR (0.01)
AXPR (0.01)
UOV (0.003)
AOV (0)
uniform (0.01)
n = 50 ; B_m = 0 ; B_x = 0 ; B_y = −0.3
CCPR (0.078)
UMPR (0.093)
UXPR (0.02)
AMPR (0.007)
AXPR (0.008)
UOV (0)
AOV (0)
uniform (0.01)
n = 100 ; B_m = 0 ; B_x = 0 ; B_y = −0.3
CCPR (0.169)
UMPR (0.1)
UXPR (0.022)
AMPR (0.015)
AXPR (0.012)
UOV (0.002)
AOV (0)
uniform (0.01)
n = 500 ; B_m = 0 ; B_x = 0 ; B_y = −0.3
CCPR (0.596)
UMPR (0.08)
UXPR (0.019)
AMPR (0.007)
AXPR (0.011)
UOV (0.002)
AOV (0)
uniform (0.01)
Pr(significance)
p−value threshold
0.000
0.004
0.008

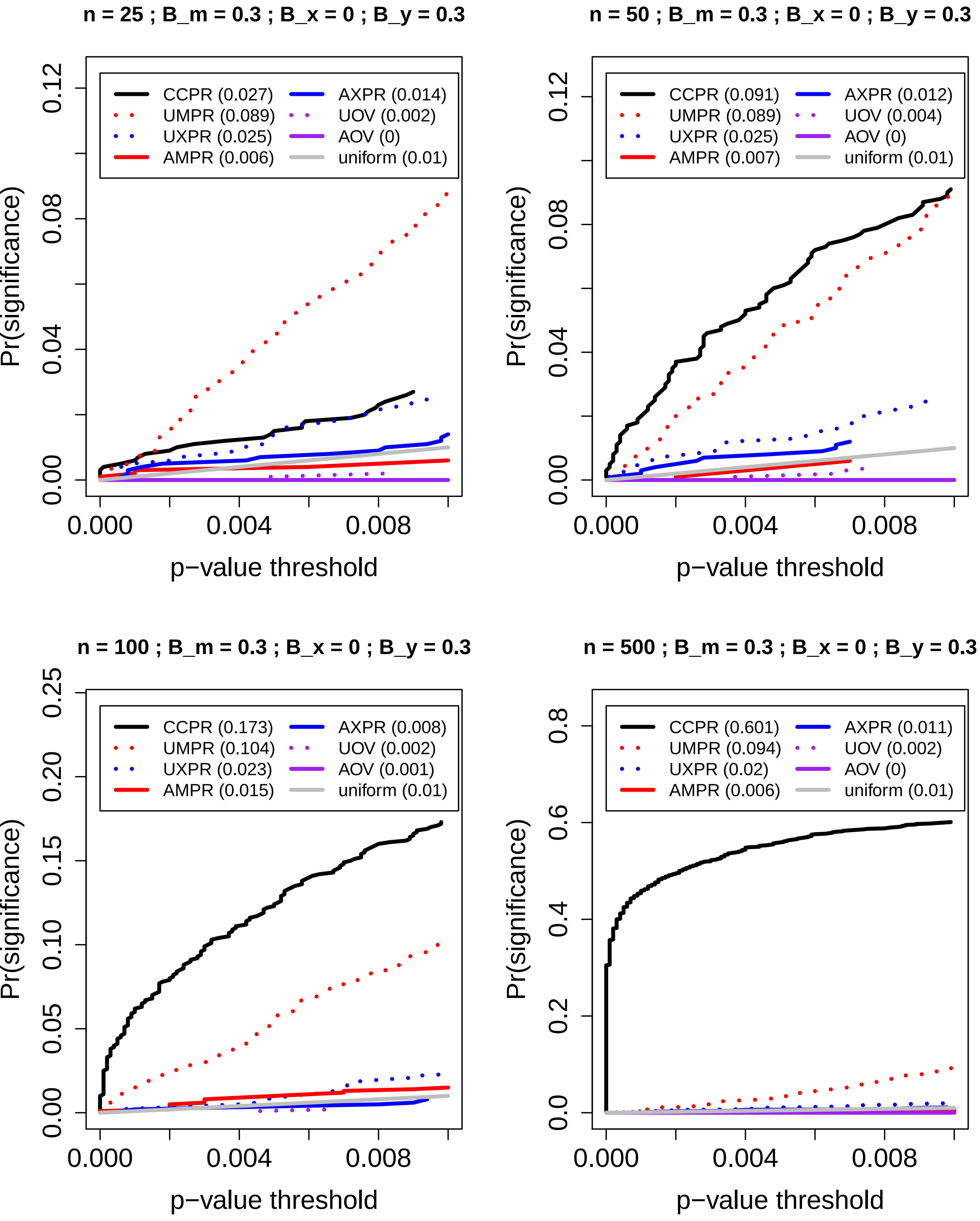

n = 25 ; B_m = 0.3 ; B_x = 0 ; B_y = 0.3
CCPR (0.027)
UMPR (0.089)
UXPR (0.025)
AMPR (0.006)
AXPR (0.014)
UOV (0.002)
AOV (0)
uniform (0.01)
n = 50 ; B_m = 0.3 ; B_x = 0 ; B_y = 0.3
CCPR (0.091)
UMPR (0.089)
UXPR (0.025)
AMPR (0.007)
AXPR (0.012)
UOV (0.004)
AOV (0)
uniform (0.01)
n = 100 ; B_m = 0.3 ; B_x = 0 ; B_y = 0.3
CCPR (0.173)
UMPR (0.104)
UXPR (0.023)
AMPR (0.015)
AXPR (0.008)
UOV (0.002)
AOV (0.001)
uniform (0.01)
n = 500 ; B_m = 0.3 ; B_x = 0 ; B_y = 0.3
CCPR (0.601)
UMPR (0.094)
UXPR (0.02)
AMPR (0.006)
AXPR (0.011)
UOV (0.002)
AOV (0)
uniform (0.01)
Pr(significance)
p−value threshold

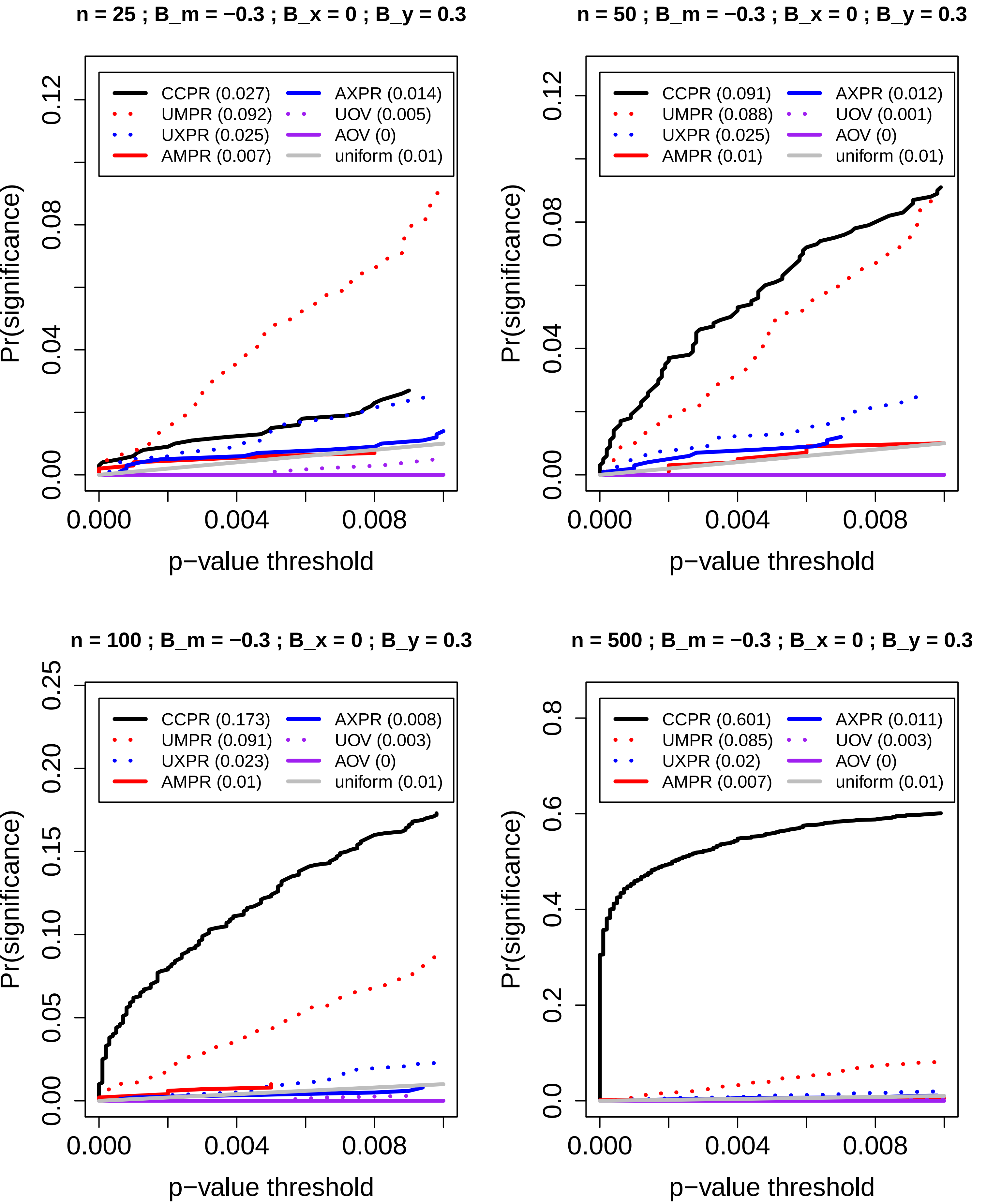
n = 25 ; B_m = −0.3 ; B_x = 0 ; B_y = 0.3
CCPR (0.027)
UMPR (0.092)
UXPR (0.025)
AMPR (0.007)
AXPR (0.014)
UOV (0.005)
AOV (0)
uniform (0.01)
Pr(significance)
p−value threshold
n = 50 ; B_m = −0.3 ; B_x = 0 ; B_y = 0.3
CCPR (0.091)
UMPR (0.088)
UXPR (0.025)
AMPR (0.01)
AXPR (0.012)
UOV (0.001)
AOV (0)
uniform (0.01)
Pr(significance)
p−value threshold
n = 100 ; B_m = −0.3 ; B_x = 0 ; B_y = 0.3
CCPR (0.173)
UMPR (0.091)
UXPR (0.023)
AMPR (0.01)
AXPR (0.008)
UOV (0.003)
AOV (0)
uniform (0.01)
Pr(significance)
p−value threshold
n = 500 ; B_m = −0.3 ; B_x = 0 ; B_y = 0.3
CCPR (0.601)
UMPR (0.085)
UXPR (0.02)
AMPR (0.007)
AXPR (0.011)
UOV (0.003)
AOV (0)
uniform (0.01)
Pr(significance)
p−value threshold

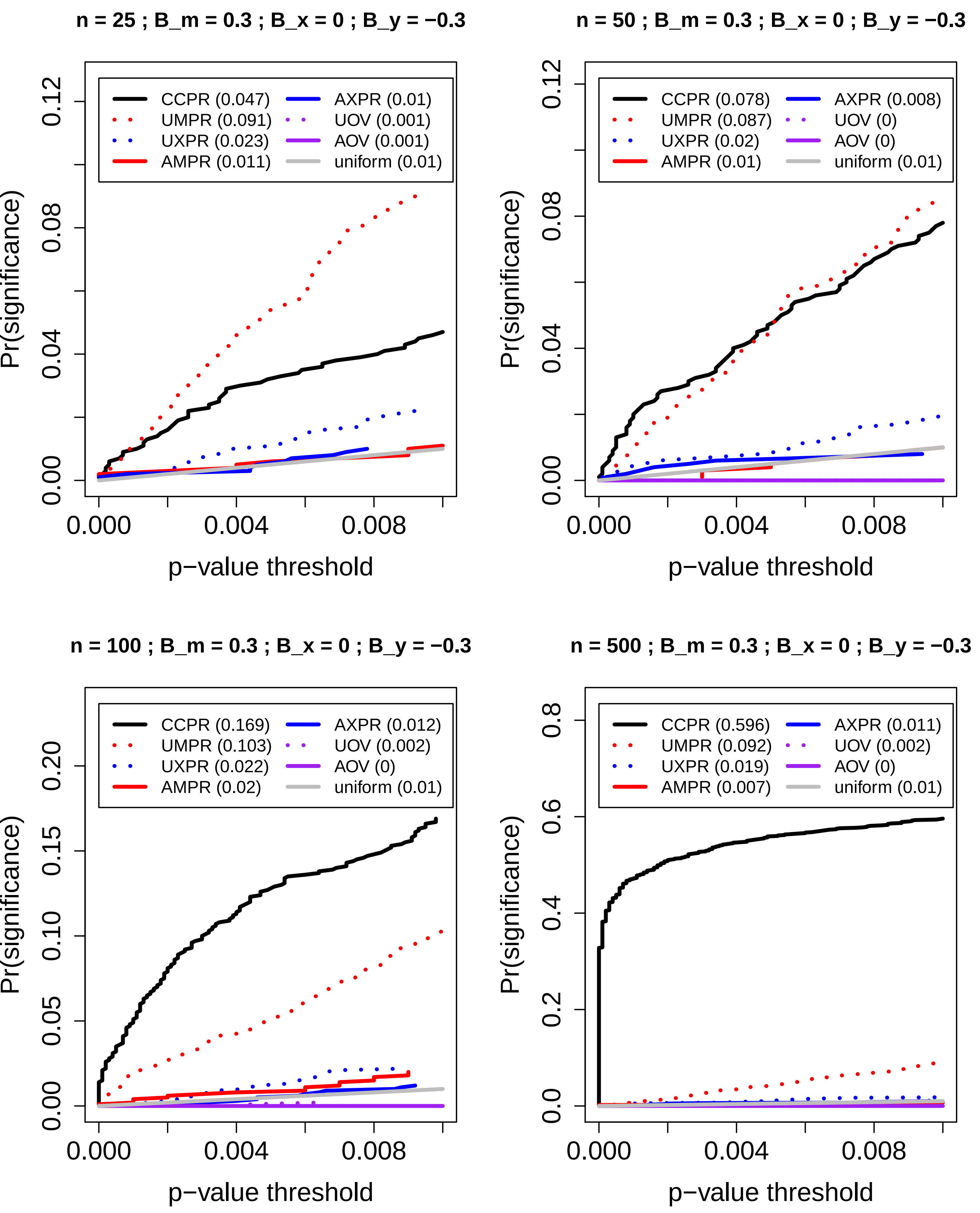

n = 25 ; B_m = 0.3 ; B_x = 0 ; B_y = −0.3
CCPR (0.047)
UMPR (0.091)
UXPR (0.023)
AMPR (0.011)
AXPR (0.01)
UOV (0.001)
AOV (0.001)
uniform (0.01)
n = 50 ; B_m = 0.3 ; B_x = 0 ; B_y = −0.3
CCPR (0.078)
UMPR (0.087)
UXPR (0.02)
AMPR (0.01)
AXPR (0.008)
UOV (0)
AOV (0)
uniform (0.01)
n = 100 ; B_m = 0.3 ; B_x = 0 ; B_y = −0.3
CCPR (0.169)
UMPR (0.103)
UXPR (0.022)
AMPR (0.02)
AXPR (0.012)
UOV (0.002)
AOV (0)
uniform (0.01)
n = 500 ; B_m = 0.3 ; B_x = 0 ; B_y = −0.3
CCPR (0.596)
UMPR (0.092)
UXPR (0.019)
AMPR (0.007)
AXPR (0.011)
UOV (0.002)
AOV (0)
uniform (0.01)
Pr(significance)
p−value threshold

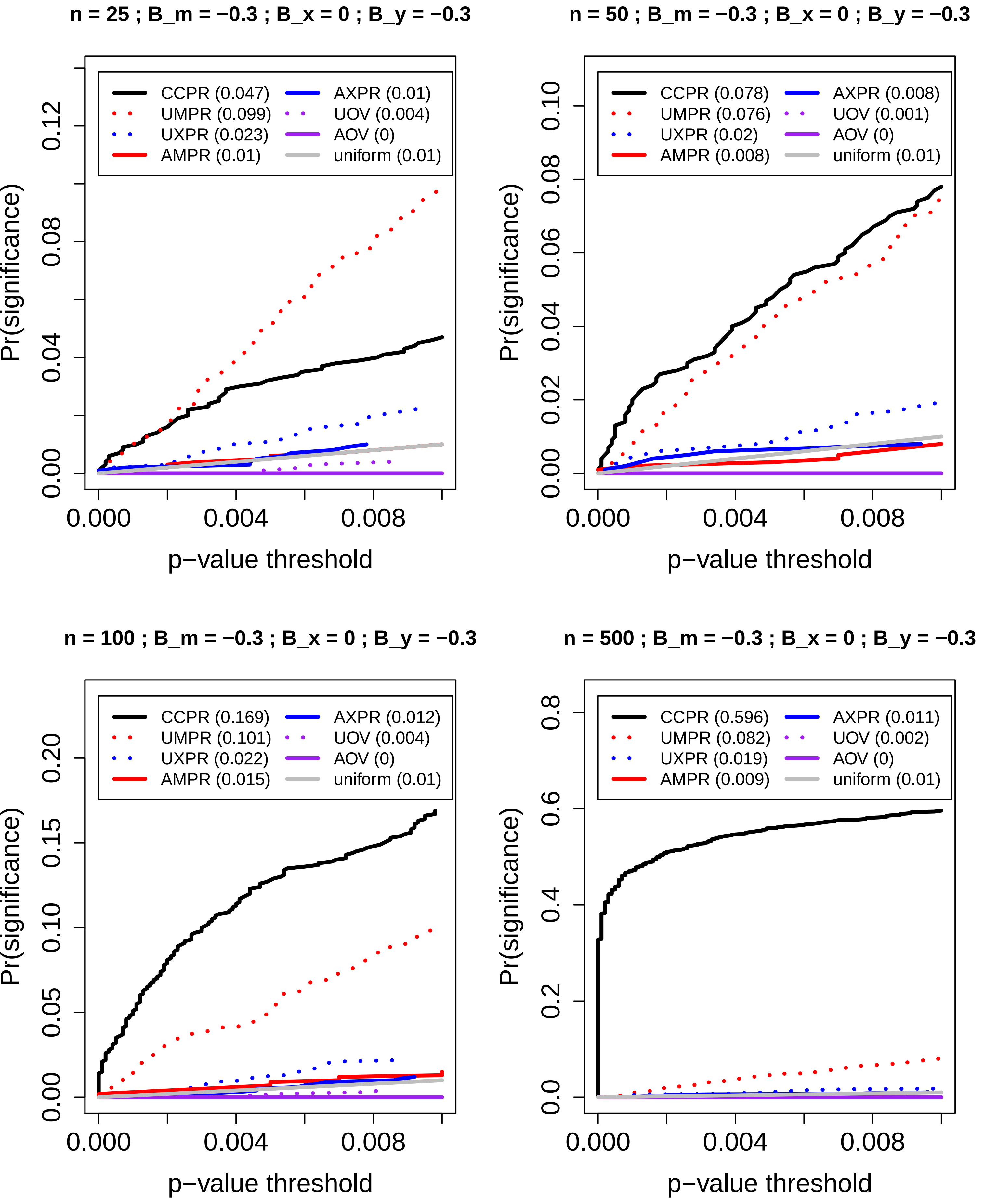
n = 25 ; B_m = −0.3 ; B_x = 0 ; B_y = −0.3
CCPR (0.047)
UMPR (0.099)
UXPR (0.023)
AMPR (0.01)
AXPR (0.01)
UOV (0.004)
AOV (0)
uniform (0.01)
n = 50 ; B_m = −0.3 ; B_x = 0 ; B_y = −0.3
CCPR (0.078)
UMPR (0.076)
UXPR (0.02)
AMPR (0.008)
AXPR (0.008)
UOV (0.001)
AOV (0)
uniform (0.01)
n = 100 ; B_m = −0.3 ; B_x = 0 ; B_y = −0.3
CCPR (0.169)
UMPR (0.101)
UXPR (0.022)
AMPR (0.015)
AXPR (0.012)
UOV (0.004)
AOV (0)
uniform (0.01)
n = 500 ; B_m = −0.3 ; B_x = 0 ; B_y = −0.3
CCPR (0.596)
UMPR (0.082)
UXPR (0.019)
AMPR (0.009)
AXPR (0.011)
UOV (0.002)
AOV (0)
uniform (0.01)
Pr(significance)
p−value threshold

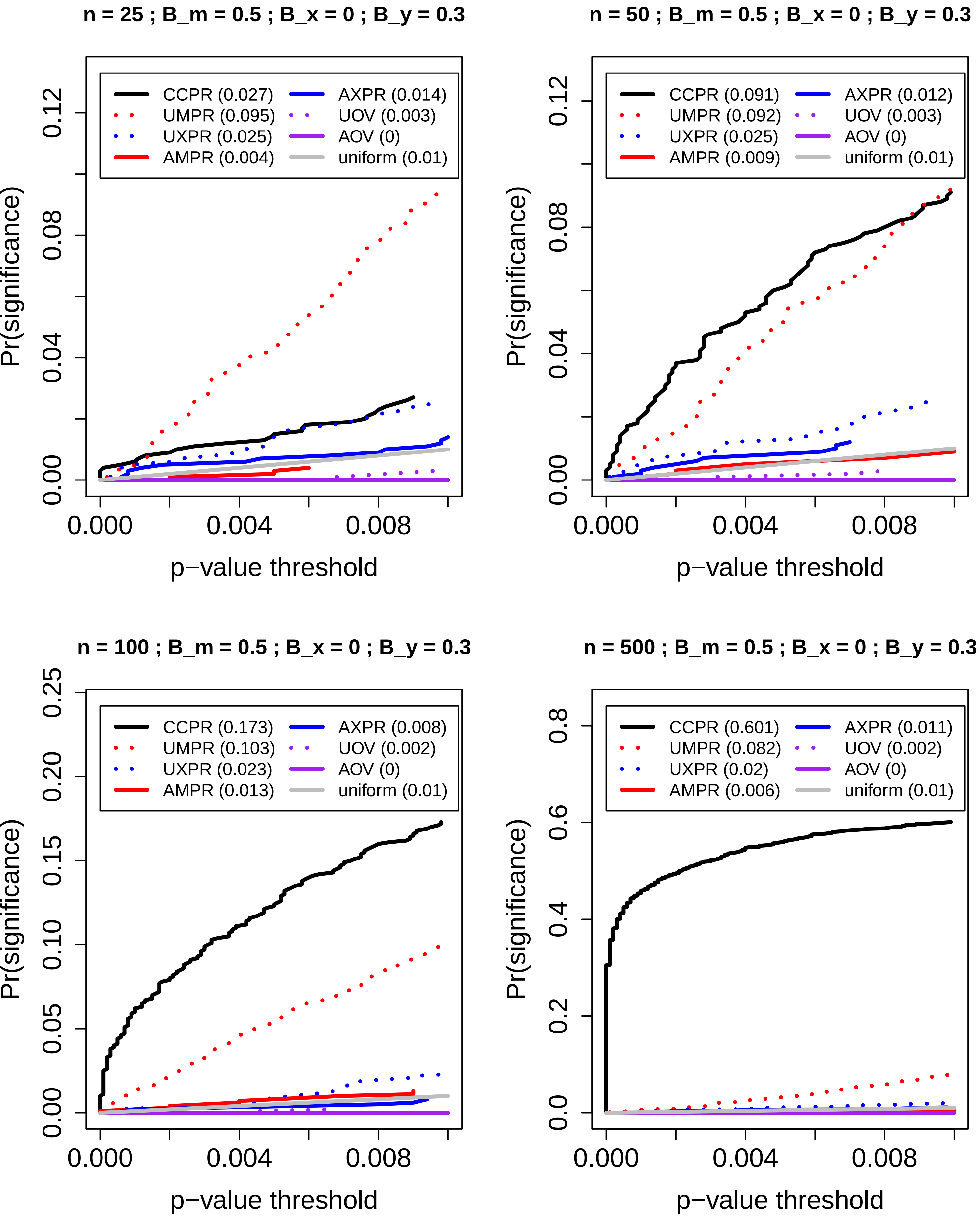

n = 25 ; B_m = 0.5 ; B_x = 0 ; B_y = 0.3
CCPR (0.027)
UMPR (0.095)
UXPR (0.025)
AMPR (0.004)
AXPR (0.014)
UOV (0.003)
AOV (0)
uniform (0.01)
Pr(significance)
p−value threshold
n = 50 ; B_m = 0.5 ; B_x = 0 ; B_y = 0.3
CCPR (0.091)
UMPR (0.092)
UXPR (0.025)
AMPR (0.009)
AXPR (0.012)
UOV (0.003)
AOV (0)
uniform (0.01)
Pr(significance)
p−value threshold
n = 100 ; B_m = 0.5 ; B_x = 0 ; B_y = 0.3
CCPR (0.173)
UMPR (0.103)
UXPR (0.023)
AMPR (0.013)
AXPR (0.008)
UOV (0.002)
AOV (0)
uniform (0.01)
Pr(significance)
p−value threshold
n = 500 ; B_m = 0.5 ; B_x = 0 ; B_y = 0.3
CCPR (0.601)
UMPR (0.082)
UXPR (0.02)
AMPR (0.006)
AXPR (0.011)
UOV (0.002)
AOV (0)
uniform (0.01)
Pr(significance)
p−value threshold

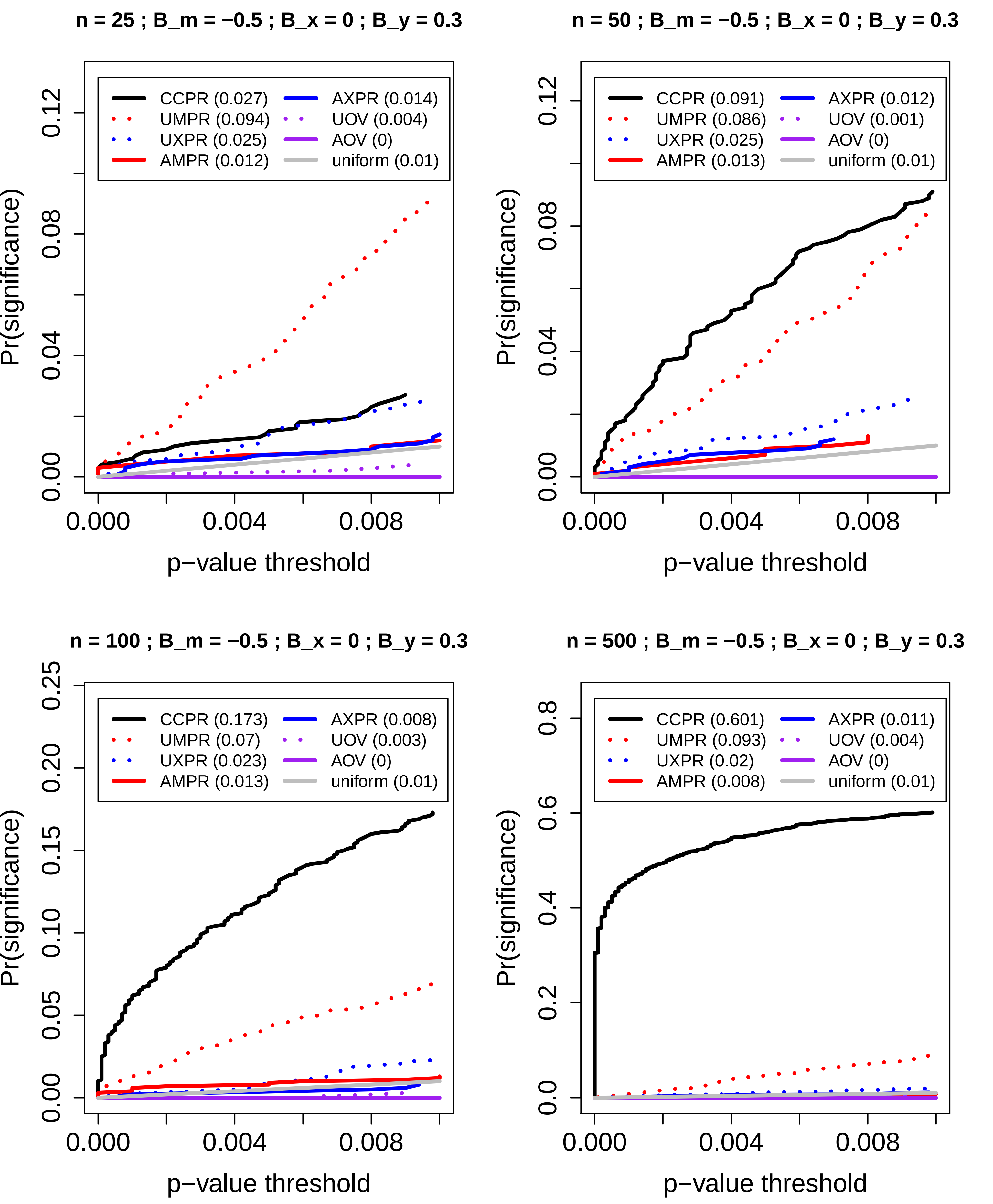

n = 25 ; B_m = −0.5 ; B_x = 0 ; B_y = 0.3
CCPR (0.027)
UMPR (0.094)
UXPR (0.025)
AMPR (0.012)
AXPR (0.014)
UOV (0.004)
AOV (0)
uniform (0.01)
n = 50 ; B_m = −0.5 ; B_x = 0 ; B_y = 0.3
CCPR (0.091)
UMPR (0.086)
UXPR (0.025)
AMPR (0.013)
AXPR (0.012)
UOV (0.001)
AOV (0)
uniform (0.01)
n = 100 ; B_m = −0.5 ; B_x = 0 ; B_y = 0.3
CCPR (0.173)
UMPR (0.07)
UXPR (0.023)
AMPR (0.013)
AXPR (0.008)
UOV (0.003)
AOV (0)
uniform (0.01)
n = 500 ; B_m = −0.5 ; B_x = 0 ; B_y = 0.3
CCPR (0.601)
UMPR (0.093)
UXPR (0.02)
AMPR (0.008)
AXPR (0.011)
UOV (0.004)
AOV (0)
uniform (0.01)
Pr(significance)
p−value threshold

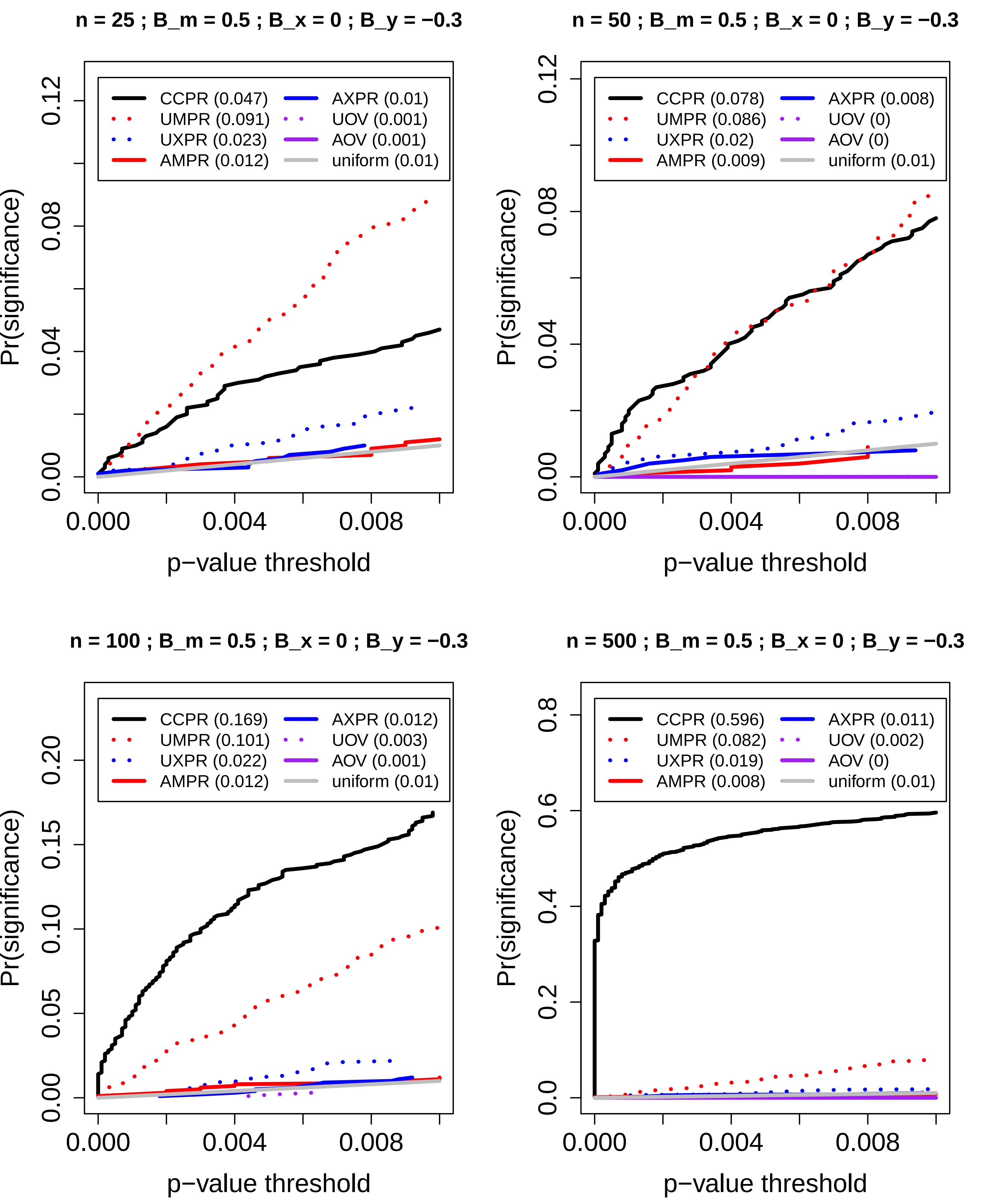
n = 25 ; B_m = 0.5 ; B_x = 0 ; B_y = −0.3
CCPR (0.047)
UMPR (0.091)
UXPR (0.023)
AMPR (0.012)
AXPR (0.01)
UOV (0.001)
AOV (0.001)
uniform (0.01)
n = 50 ; B_m = 0.5 ; B_x = 0 ; B_y = −0.3
CCPR (0.078)
UMPR (0.086)
UXPR (0.02)
AMPR (0.009)
AXPR (0.008)
UOV (0)
AOV (0)
uniform (0.01)
n = 100 ; B_m = 0.5 ; B_x = 0 ; B_y = −0.3
CCPR (0.169)
UMPR (0.101)
UXPR (0.022)
AMPR (0.012)
AXPR (0.012)
UOV (0.003)
AOV (0.001)
uniform (0.01)
n = 500 ; B_m = 0.5 ; B_x = 0 ; B_y = −0.3
CCPR (0.596)
UMPR (0.082)
UXPR (0.019)
AMPR (0.008)
AXPR (0.011)
UOV (0.002)
AOV (0)
uniform (0.01)
Pr(significance)
p−value threshold

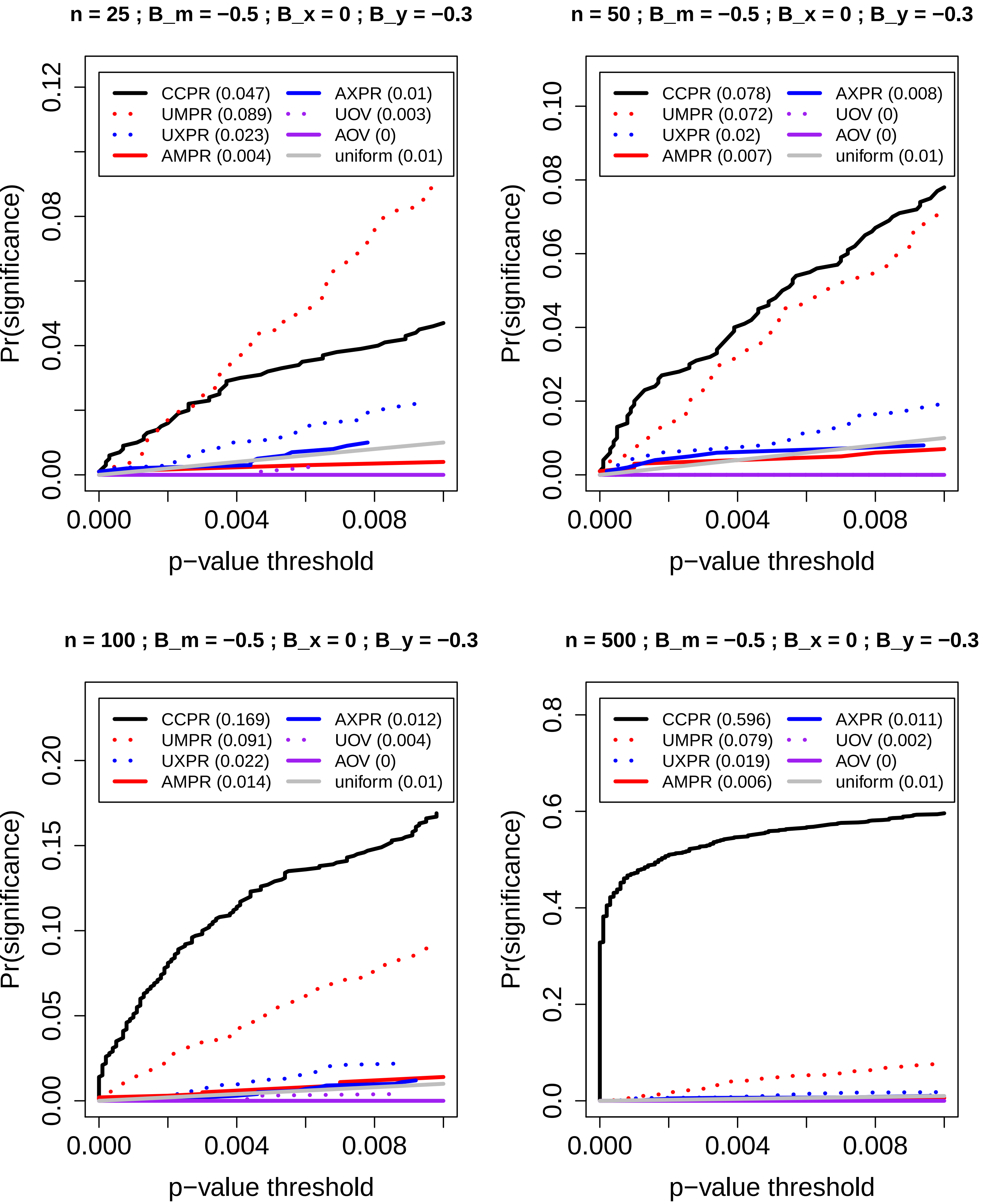

n = 25 ; B_m = −0.5 ; B_x = 0 ; B_y = −0.3
CCPR (0.047)
UMPR (0.089)
UXPR (0.023)
AMPR (0.004)
AXPR (0.01)
UOV (0.003)
AOV (0)
uniform (0.01)
n = 50 ; B_m = −0.5 ; B_x = 0 ; B_y = −0.3
CCPR (0.078)
UMPR (0.072)
UXPR (0.02)
AMPR (0.007)
AXPR (0.008)
UOV (0)
AOV (0)
uniform (0.01)
n = 100 ; B_m = −0.5 ; B_x = 0 ; B_y = −0.3
CCPR (0.169)
UMPR (0.091)
UXPR (0.022)
AMPR (0.014)
AXPR (0.012)
UOV (0.004)
AOV (0)
uniform (0.01)
n = 500 ; B_m = −0.5 ; B_x = 0 ; B_y = −0.3
CCPR (0.596)
UMPR (0.079)
UXPR (0.019)
AMPR (0.006)
AXPR (0.011)
UOV (0.002)
AOV (0)
uniform (0.01)
Pr(significance)
p−value threshold

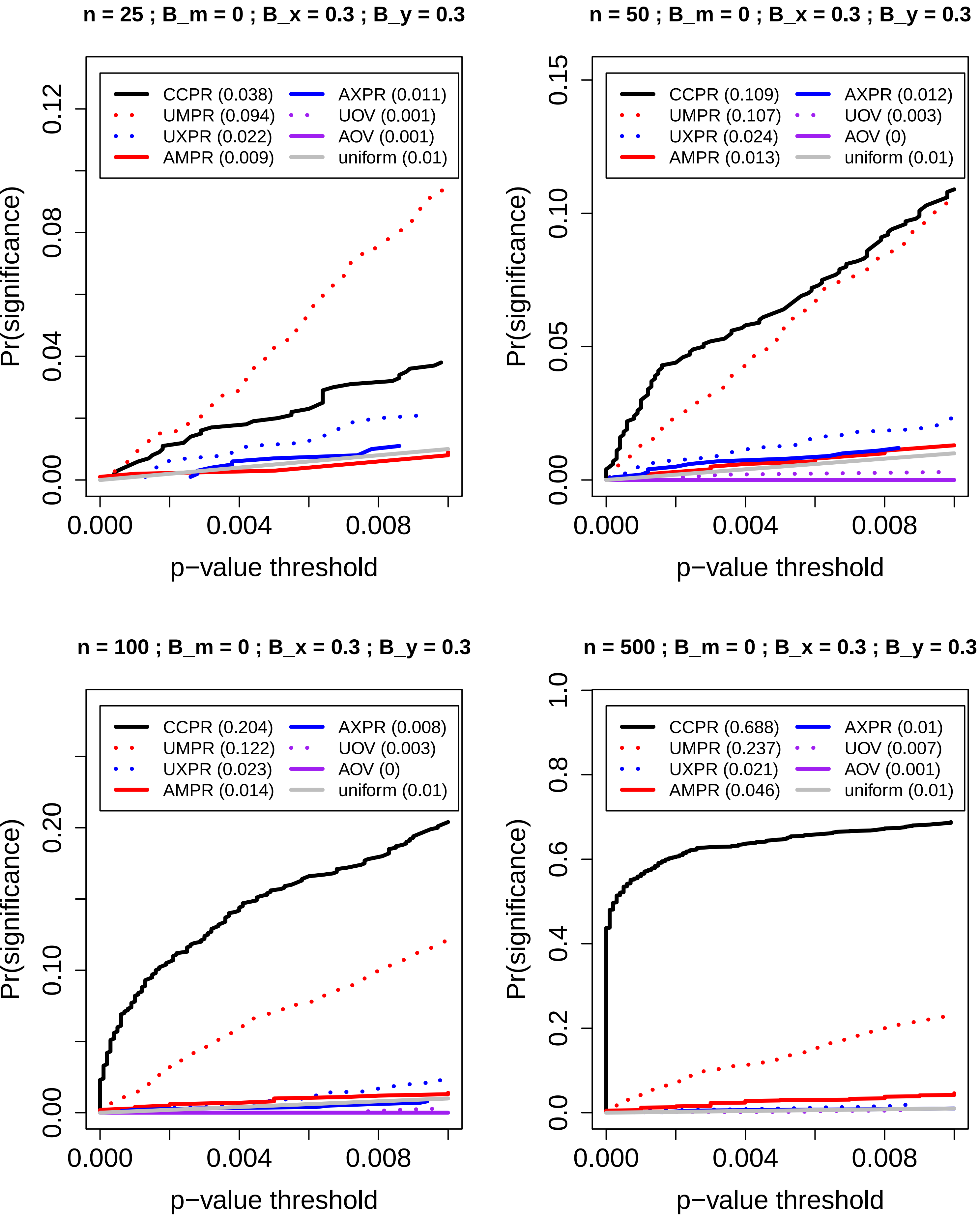

n = 25 ; B_m = 0 ; B_x = 0.3 ; B_y = 0.3
CCPR (0.038)
UMPR (0.094)
UXPR (0.022)
AMPR (0.009)
AXPR (0.011)
UOV (0.001)
AOV (0.001)
uniform (0.01)
n = 50 ; B_m = 0 ; B_x = 0.3 ; B_y = 0.3
CCPR (0.109)
UMPR (0.107)
UXPR (0.024)
AMPR (0.013)
AXPR (0.012)
UOV (0.003)
AOV (0)
uniform (0.01)
n = 100 ; B_m = 0 ; B_x = 0.3 ; B_y = 0.3
CCPR (0.204)
UMPR (0.122)
UXPR (0.023)
AMPR (0.014)
AXPR (0.008)
UOV (0.003)
AOV (0)
uniform (0.01)
n = 500 ; B_m = 0 ; B_x = 0.3 ; B_y = 0.3
CCPR (0.688)
UMPR (0.237)
UXPR (0.021)
AMPR (0.046)
AXPR (0.01)
UOV (0.007)
AOV (0.001)
uniform (0.01)
Pr(significance)
p−value threshold

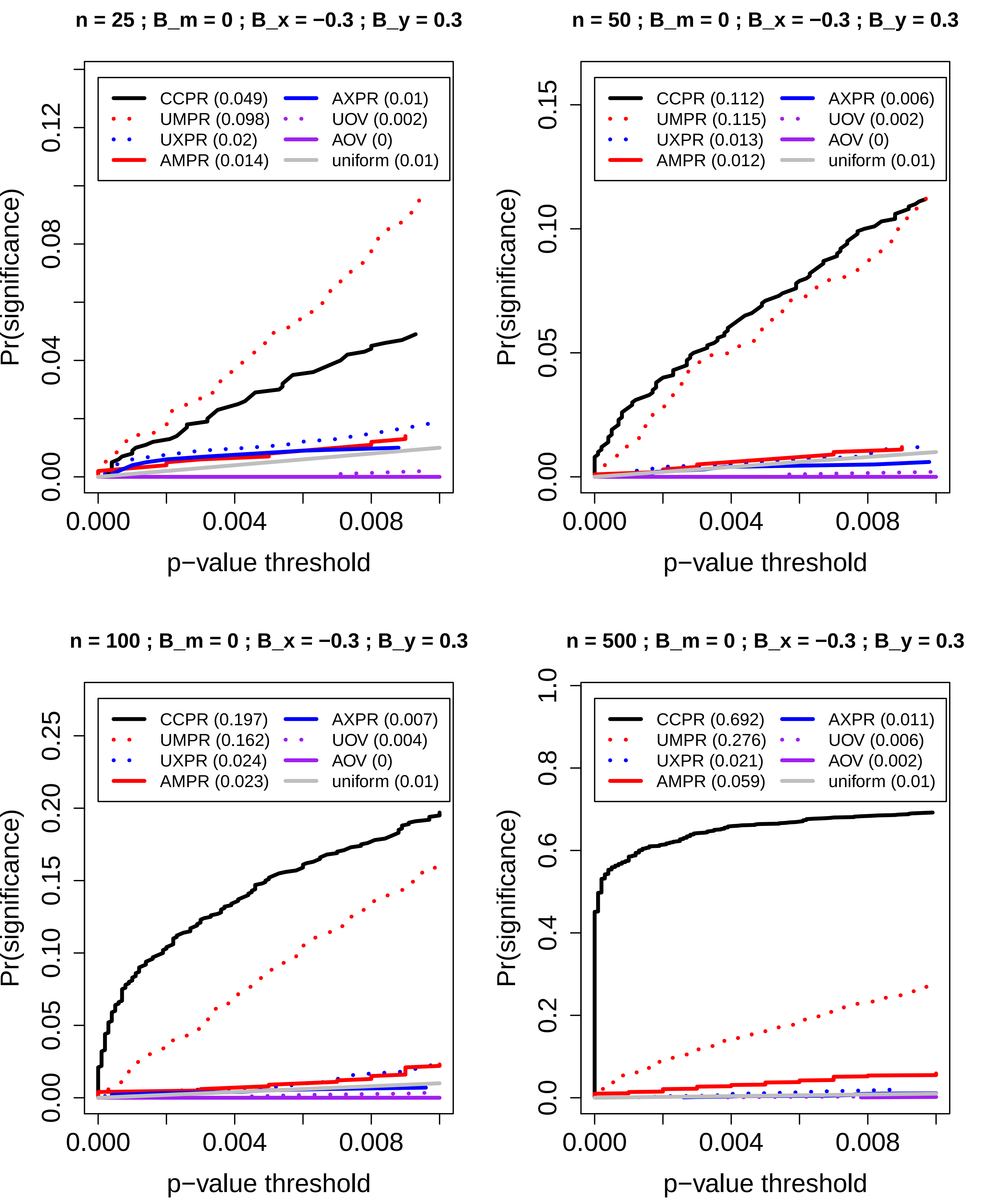

n = 25 ; B_m = 0 ; B_x = −0.3 ; B_y = 0.3
CCPR (0.049)
UMPR (0.098)
UXPR (0.02)
AMPR (0.014)
AXPR (0.01)
UOV (0.002)
AOV (0)
uniform (0.01)
Pr(significance)
p−value threshold
n = 50 ; B_m = 0 ; B_x = −0.3 ; B_y = 0.3
CCPR (0.112)
UMPR (0.115)
UXPR (0.013)
AMPR (0.012)
AXPR (0.006)
UOV (0.002)
AOV (0)
uniform (0.01)
Pr(significance)
p−value threshold
n = 100 ; B_m = 0 ; B_x = −0.3 ; B_y = 0.3
CCPR (0.197)
UMPR (0.162)
UXPR (0.024)
AMPR (0.023)
AXPR (0.007)
UOV (0.004)
AOV (0)
uniform (0.01)
Pr(significance)
p−value threshold
n = 500 ; B_m = 0 ; B_x = −0.3 ; B_y = 0.3
CCPR (0.692)
UMPR (0.276)
UXPR (0.021)
AMPR (0.059)
AXPR (0.011)
UOV (0.006)
AOV (0.002)
uniform (0.01)
Pr(significance)
p−value threshold

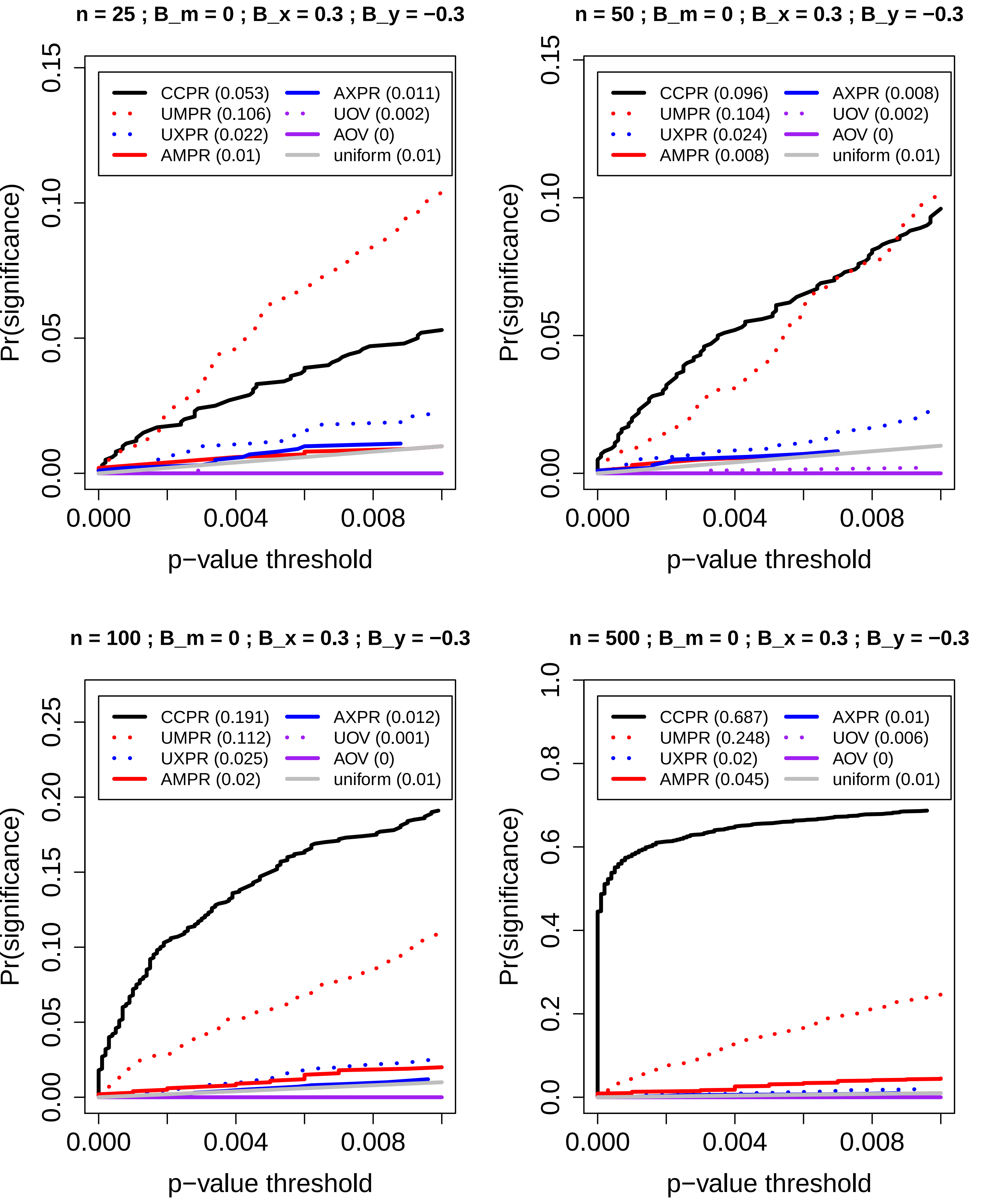
n = 25 ; B_m = 0 ; B_x = 0.3 ; B_y = −0.3
CCPR (0.053)
UMPR (0.106)
UXPR (0.022)
AMPR (0.01)
AXPR (0.011)
UOV (0.002)
AOV (0)
uniform (0.01)
n = 50 ; B_m = 0 ; B_x = 0.3 ; B_y = −0.3
CCPR (0.096)
UMPR (0.104)
UXPR (0.024)
AMPR (0.008)
AXPR (0.008)
UOV (0.002)
AOV (0)
uniform (0.01)
n = 100 ; B_m = 0 ; B_x = 0.3 ; B_y = −0.3
CCPR (0.191)
UMPR (0.112)
UXPR (0.025)
AMPR (0.02)
AXPR (0.012)
UOV (0.001)
AOV (0)
uniform (0.01)
n = 500 ; B_m = 0 ; B_x = 0.3 ; B_y = −0.3
CCPR (0.687)
UMPR (0.248)
UXPR (0.02)
AMPR (0.045)
AXPR (0.01)
UOV (0.006)
AOV (0)
uniform (0.01)
Pr(significance)
p−value threshold

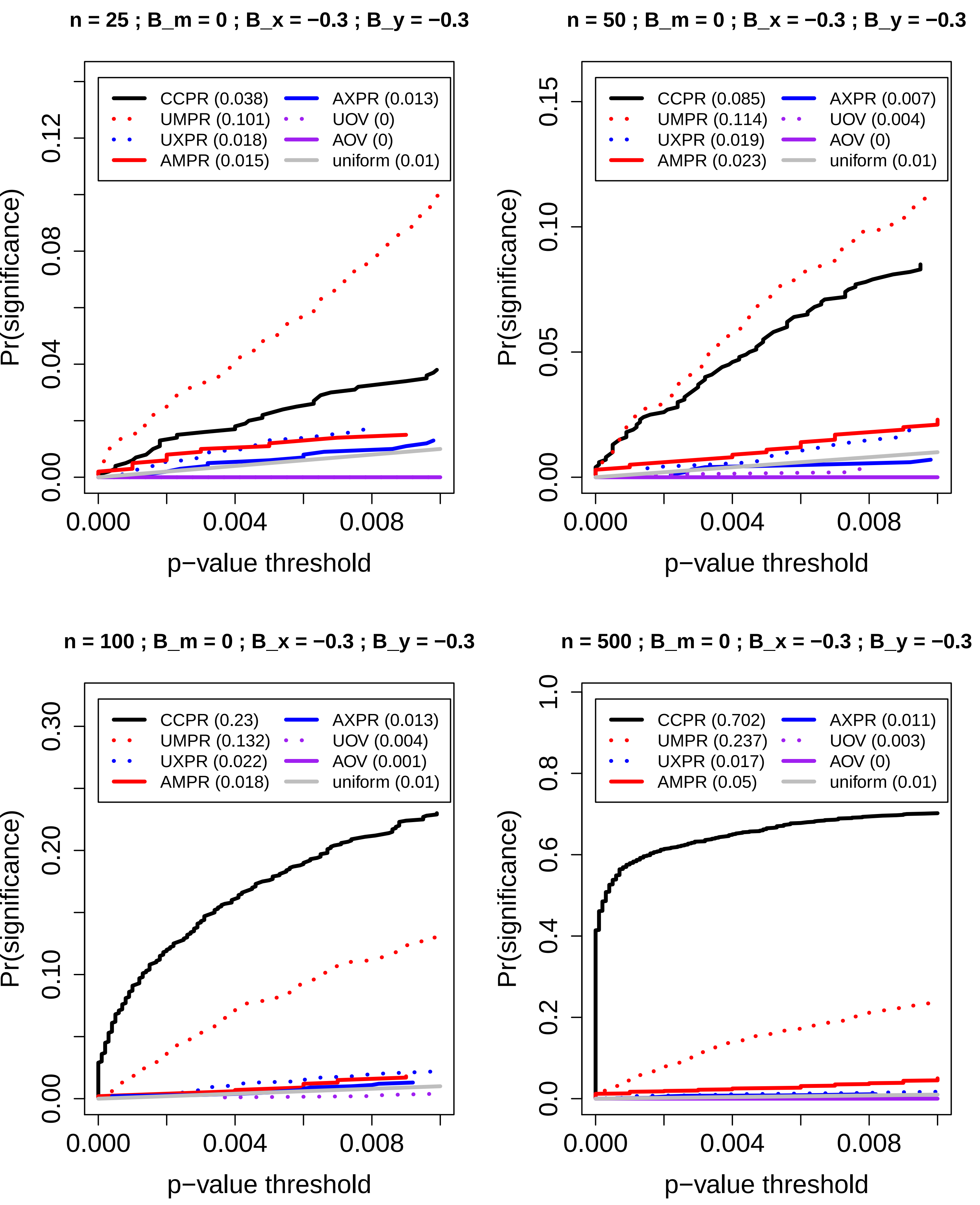

n = 25 ; B_m = 0 ; B_x = −0.3 ; B_y = −0.3
CCPR (0.038)
UMPR (0.101)
UXPR (0.018)
AMPR (0.015)
AXPR (0.013)
UOV (0)
AOV (0)
uniform (0.01)
Pr(significance)
0.00
0.04
0.08
0.12
0.000
0.004
0.008
p−value threshold
n = 50 ; B_m = 0 ; B_x = −0.3 ; B_y = −0.3
CCPR (0.085)
UMPR (0.114)
UXPR (0.019)
AMPR (0.023)
AXPR (0.007)
UOV (0.004)
AOV (0)
uniform (0.01)
Pr(significance)
0.00
0.05
0.10
0.15
0.000
0.004
0.008
p−value threshold
n = 100 ; B_m = 0 ; B_x = −0.3 ; B_y = −0.3
CCPR (0.23)
UMPR (0.132)
UXPR (0.022)
AMPR (0.018)
AXPR (0.013)
UOV (0.004)
AOV (0.001)
uniform (0.01)
Pr(significance)
0.00
0.10
0.20
0.30
0.000
0.004
0.008
p−value threshold
n = 500 ; B_m = 0 ; B_x = −0.3 ; B_y = −0.3
CCPR (0.702)
UMPR (0.237)
UXPR (0.017)
AMPR (0.05)
AXPR (0.011)
UOV (0.003)
AOV (0)
uniform (0.01)
Pr(significance)
0.0
0.2
0.4
0.6
0.8
1.0
0.000
0.004
0.008
p−value threshold

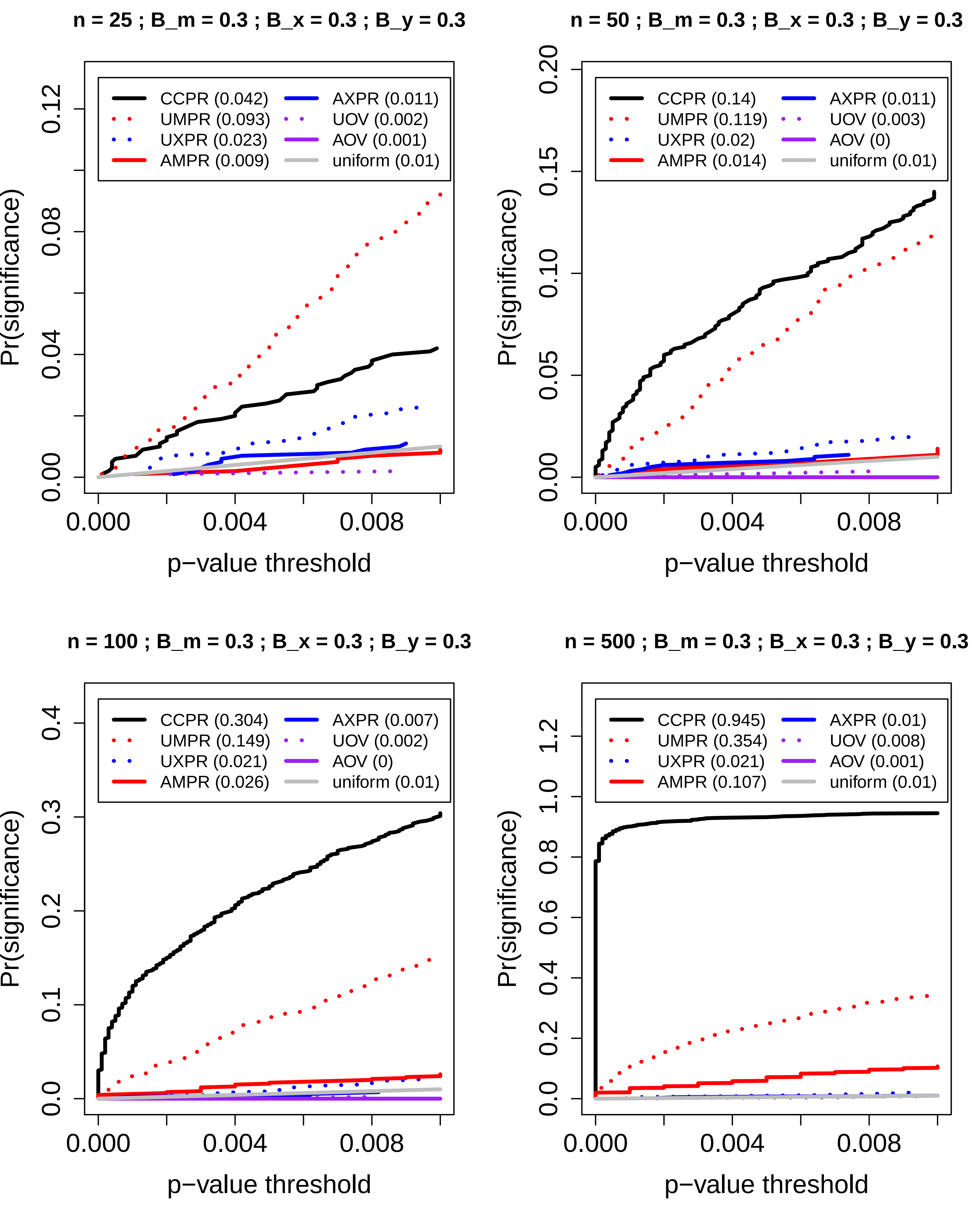

n = 25 ; B_m = 0.3 ; B_x = 0.3 ; B_y = 0.3
CCPR (0.042)
UMPR (0.093)
UXPR (0.023)
AMPR (0.009)
AXPR (0.011)
UOV (0.002)
AOV (0.001)
uniform (0.01)
Pr(significance)
p−value threshold
n = 50 ; B_m = 0.3 ; B_x = 0.3 ; B_y = 0.3
CCPR (0.14)
UMPR (0.119)
UXPR (0.02)
AMPR (0.014)
AXPR (0.011)
UOV (0.003)
AOV (0)
uniform (0.01)
Pr(significance)
p−value threshold
n = 100 ; B_m = 0.3 ; B_x = 0.3 ; B_y = 0.3
CCPR (0.304)
UMPR (0.149)
UXPR (0.021)
AMPR (0.026)
AXPR (0.007)
UOV (0.002)
AOV (0)
uniform (0.01)
Pr(significance)
p−value threshold
n = 500 ; B_m = 0.3 ; B_x = 0.3 ; B_y = 0.3
CCPR (0.945)
UMPR (0.354)
UXPR (0.021)
AMPR (0.107)
AXPR (0.01)
UOV (0.008)
AOV (0.001)
uniform (0.01)
Pr(significance)
p−value threshold

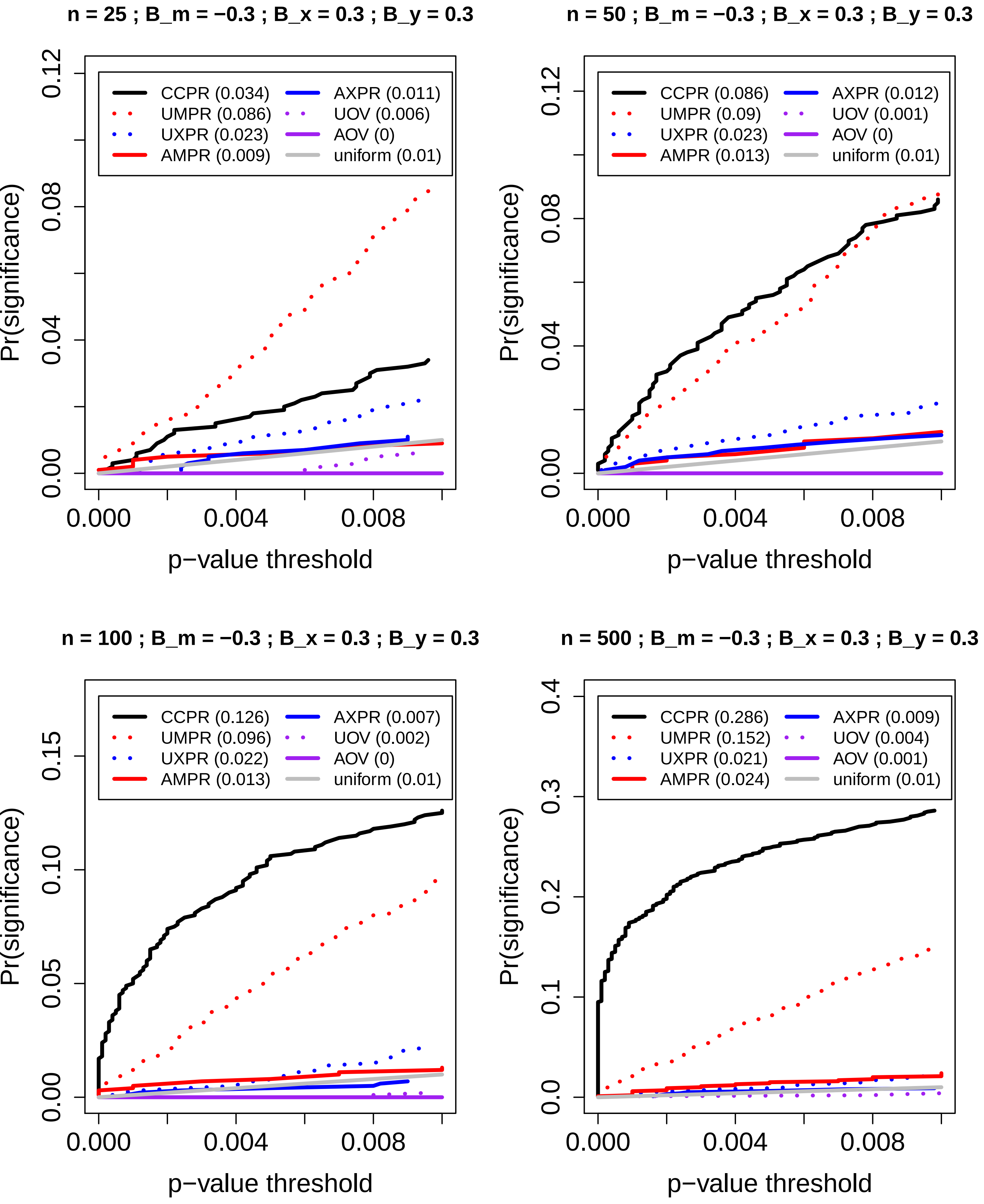

n = 25 ; B_m = −0.3 ; B_x = 0.3 ; B_y = 0.3
CCPR (0.034)
UMPR (0.086)
UXPR (0.023)
AMPR (0.009)
AXPR (0.011)
UOV (0.006)
AOV (0)
uniform (0.01)
Pr(significance)
p−value threshold
n = 50 ; B_m = −0.3 ; B_x = 0.3 ; B_y = 0.3
CCPR (0.086)
UMPR (0.09)
UXPR (0.023)
AMPR (0.013)
AXPR (0.012)
UOV (0.001)
AOV (0)
uniform (0.01)
Pr(significance)
p−value threshold
n = 100 ; B_m = −0.3 ; B_x = 0.3 ; B_y = 0.3
CCPR (0.126)
UMPR (0.096)
UXPR (0.022)
AMPR (0.013)
AXPR (0.007)
UOV (0.002)
AOV (0)
uniform (0.01)
Pr(significance)
p−value threshold
n = 500 ; B_m = −0.3 ; B_x = 0.3 ; B_y = 0.3
CCPR (0.286)
UMPR (0.152)
UXPR (0.021)
AMPR (0.024)
AXPR (0.009)
UOV (0.004)
AOV (0.001)
uniform (0.01)
Pr(significance)
p−value threshold

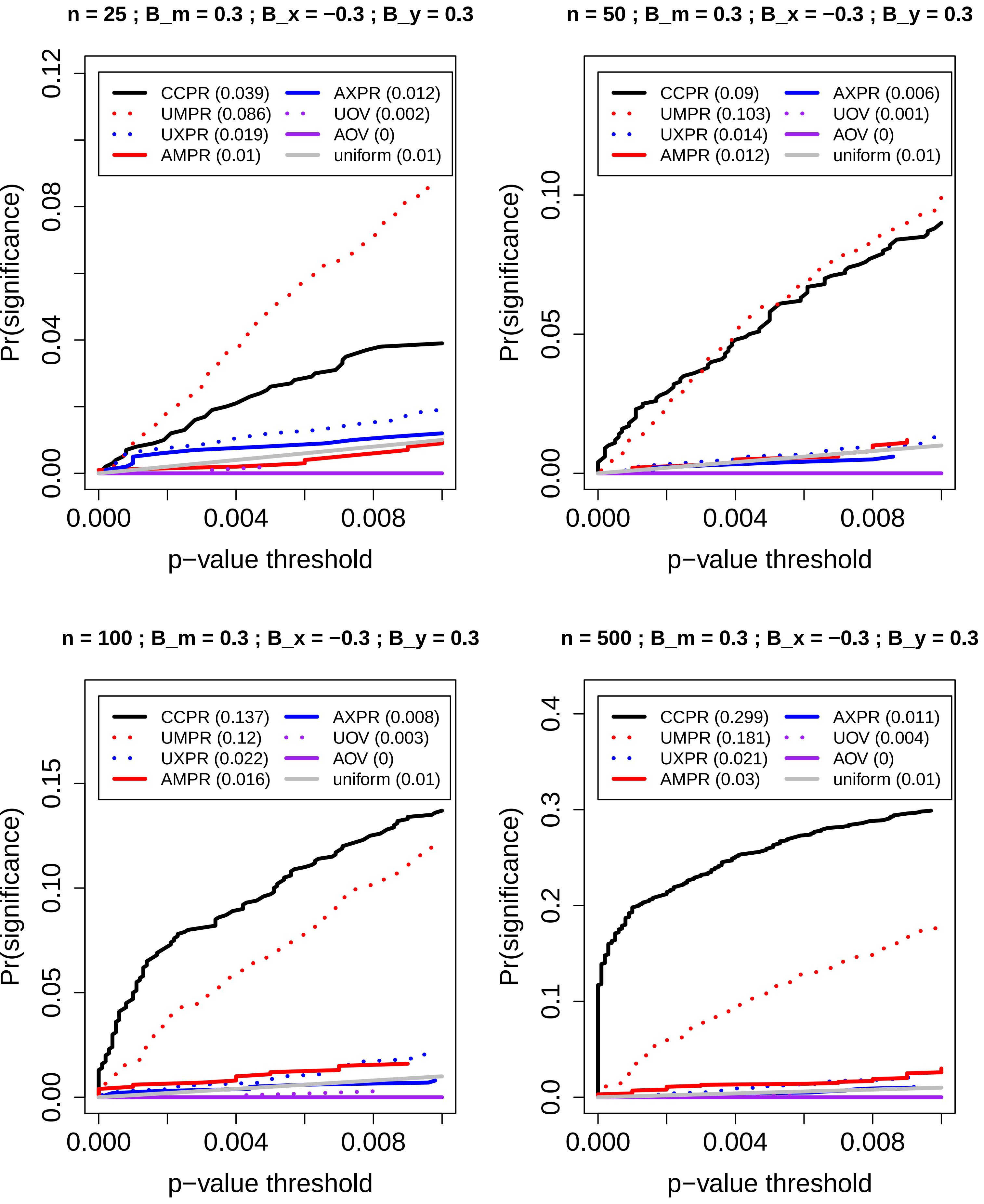
n = 25 ; B_m = 0.3 ; B_x = −0.3 ; B_y = 0.3
CCPR (0.039)
UMPR (0.086)
UXPR (0.019)
AMPR (0.01)
AXPR (0.012)
UOV (0.002)
AOV (0)
uniform (0.01)
Pr(significance)
0.00
0.04
0.08
0.12
0.000
0.004
0.008
p−value threshold
n = 50 ; B_m = 0.3 ; B_x = −0.3 ; B_y = 0.3
CCPR (0.09)
UMPR (0.103)
UXPR (0.014)
AMPR (0.012)
AXPR (0.006)
UOV (0.001)
AOV (0)
uniform (0.01)
Pr(significance)
0.00
0.05
0.10
0.000
0.004
0.008
p−value threshold
n = 100 ; B_m = 0.3 ; B_x = −0.3 ; B_y = 0.3
CCPR (0.137)
UMPR (0.12)
UXPR (0.022)
AMPR (0.016)
AXPR (0.008)
UOV (0.003)
AOV (0)
uniform (0.01)
Pr(significance)
0.00
0.05
0.10
0.15
0.000
0.004
0.008
p−value threshold
n = 500 ; B_m = 0.3 ; B_x = −0.3 ; B_y = 0.3
CCPR (0.299)
UMPR (0.181)
UXPR (0.021)
AMPR (0.03)
AXPR (0.011)
UOV (0.004)
AOV (0)
uniform (0.01)
Pr(significance)
0.0
0.1
0.2
0.3
0.4
0.000
0.004
0.008
p−value threshold

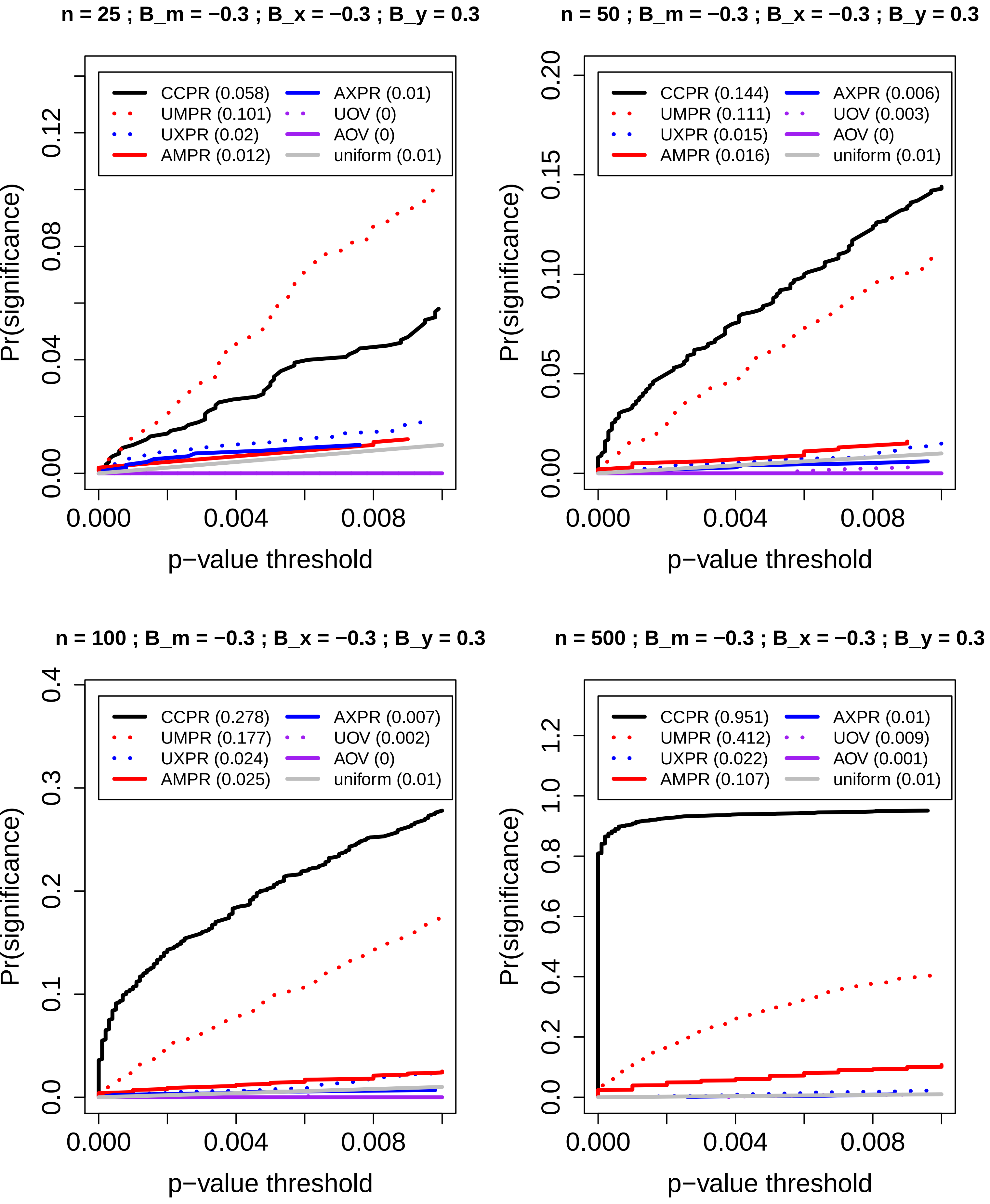

n = 25 ; B_m = −0.3 ; B_x = −0.3 ; B_y = 0.3
CCPR (0.058)
UMPR (0.101)
UXPR (0.02)
AMPR (0.012)
AXPR (0.01)
UOV (0)
AOV (0)
uniform (0.01)
Pr(significance)
0.00
0.04
0.08
0.12
0.000
0.004
0.008
p−value threshold
n = 50 ; B_m = −0.3 ; B_x = −0.3 ; B_y = 0.3
CCPR (0.144)
UMPR (0.111)
UXPR (0.015)
AMPR (0.016)
AXPR (0.006)
UOV (0.003)
AOV (0)
uniform (0.01)
Pr(significance)
0.00
0.05
0.10
0.15
0.20
0.000
0.004
0.008
p−value threshold
n = 100 ; B_m = −0.3 ; B_x = −0.3 ; B_y = 0.3
CCPR (0.278)
UMPR (0.177)
UXPR (0.024)
AMPR (0.025)
AXPR (0.007)
UOV (0.002)
AOV (0)
uniform (0.01)
Pr(significance)
0.0
0.1
0.2
0.3
0.4
0.000
0.004
0.008
p−value threshold
n = 500 ; B_m = −0.3 ; B_x = −0.3 ; B_y = 0.3
CCPR (0.951)
UMPR (0.412)
UXPR (0.022)
AMPR (0.107)
AXPR (0.01)
UOV (0.009)
AOV (0.001)
uniform (0.01)
Pr(significance)
0.0
0.2
0.4
0.6
0.8
1.0
1.2
0.000
0.004
0.008
p−value threshold

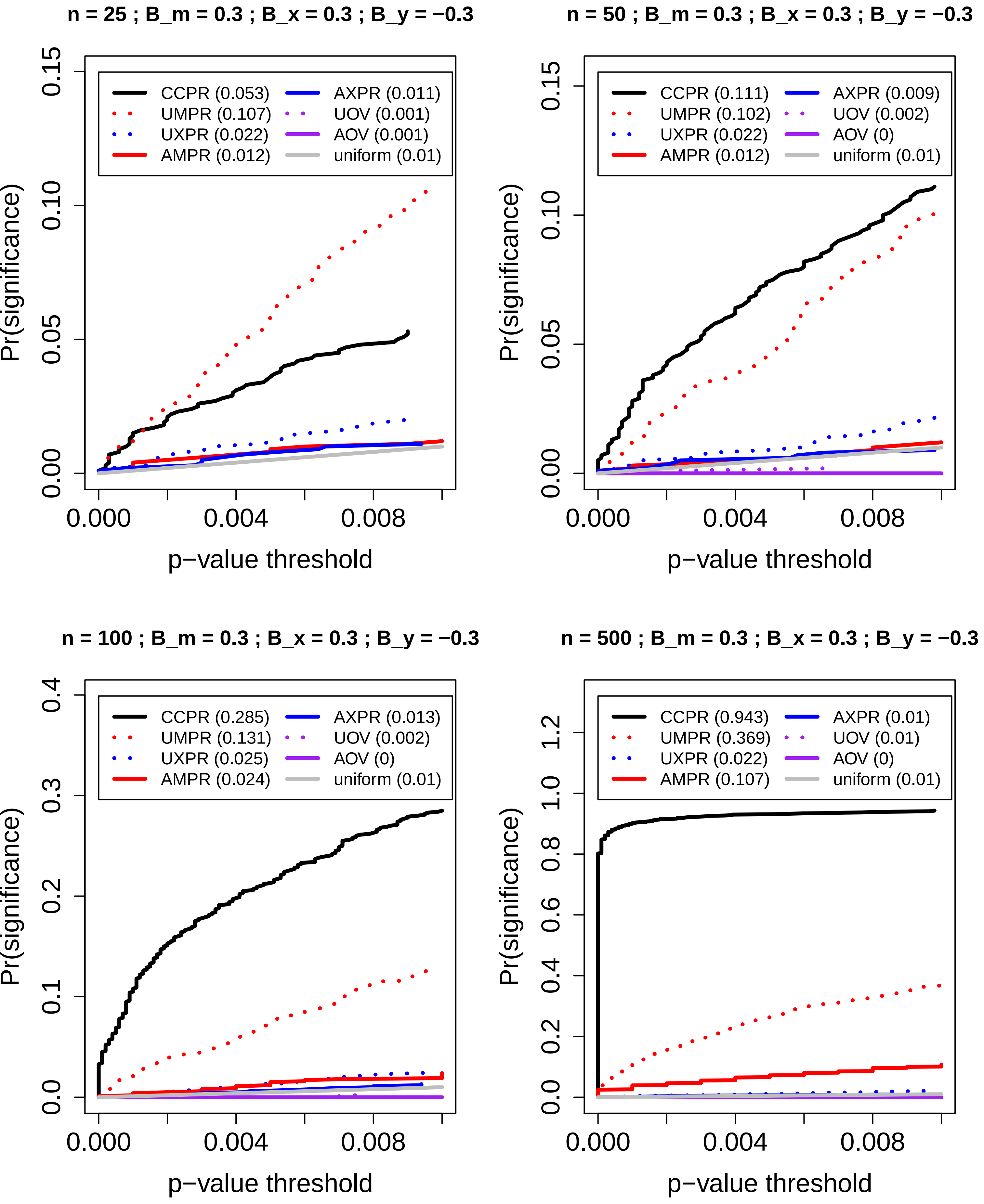
n = 25 ; B_m = 0.3 ; B_x = 0.3 ; B_y = −0.3
CCPR (0.053)
UMPR (0.107)
UXPR (0.022)
AMPR (0.012)
AXPR (0.011)
UOV (0.001)
AOV (0.001)
uniform (0.01)
n = 50 ; B_m = 0.3 ; B_x = 0.3 ; B_y = −0.3
CCPR (0.111)
UMPR (0.102)
UXPR (0.022)
AMPR (0.012)
AXPR (0.009)
UOV (0.002)
AOV (0)
uniform (0.01)
n = 100 ; B_m = 0.3 ; B_x = 0.3 ; B_y = −0.3
CCPR (0.285)
UMPR (0.131)
UXPR (0.025)
AMPR (0.024)
AXPR (0.013)
UOV (0.002)
AOV (0)
uniform (0.01)
n = 500 ; B_m = 0.3 ; B_x = 0.3 ; B_y = −0.3
CCPR (0.943)
UMPR (0.369)
UXPR (0.022)
AMPR (0.107)
AXPR (0.01)
UOV (0.01)
AOV (0)
uniform (0.01)
Pr(significance)
p−value threshold
0.000
0.004
0.008
0.00
0.05
0.10
0.15
0.0
0.1
0.2
0.3
0.4
0.6
0.8
1.0
1.2

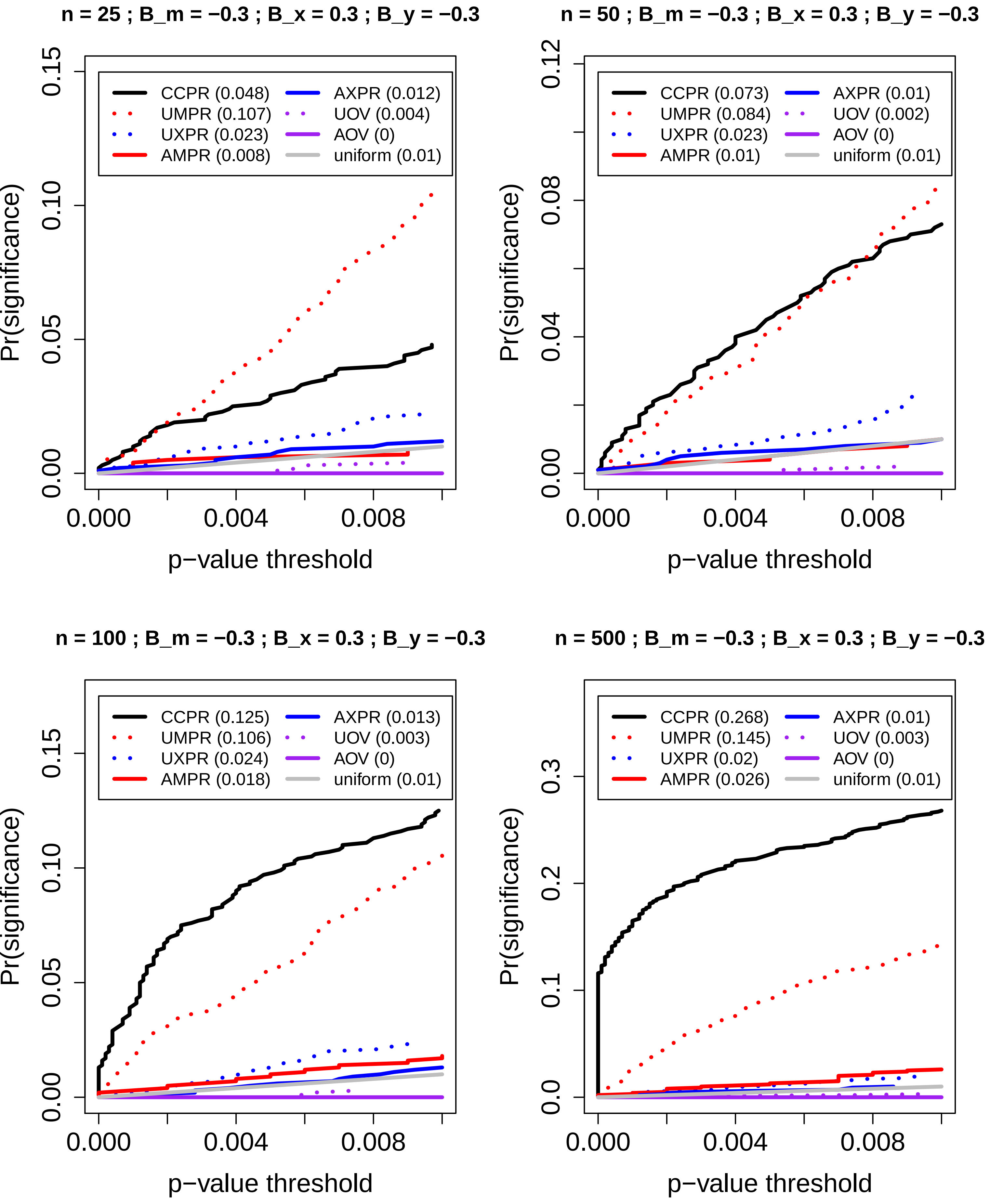

n = 25 ; B_m = −0.3 ; B_x = 0.3 ; B_y = −0.3
CCPR (0.048)
UMPR (0.107)
UXPR (0.023)
AMPR (0.008)
AXPR (0.012)
UOV (0.004)
AOV (0)
uniform (0.01)
Pr(significance)
p−value threshold
n = 50 ; B_m = −0.3 ; B_x = 0.3 ; B_y = −0.3
CCPR (0.073)
UMPR (0.084)
UXPR (0.023)
AMPR (0.01)
AXPR (0.01)
UOV (0.002)
AOV (0)
uniform (0.01)
Pr(significance)
p−value threshold
n = 100 ; B_m = −0.3 ; B_x = 0.3 ; B_y = −0.3
CCPR (0.125)
UMPR (0.106)
UXPR (0.024)
AMPR (0.018)
AXPR (0.013)
UOV (0.003)
AOV (0)
uniform (0.01)
Pr(significance)
p−value threshold
n = 500 ; B_m = −0.3 ; B_x = 0.3 ; B_y = −0.3
CCPR (0.268)
UMPR (0.145)
UXPR (0.02)
AMPR (0.026)
AXPR (0.01)
UOV (0.003)
AOV (0)
uniform (0.01)
Pr(significance)
p−value threshold

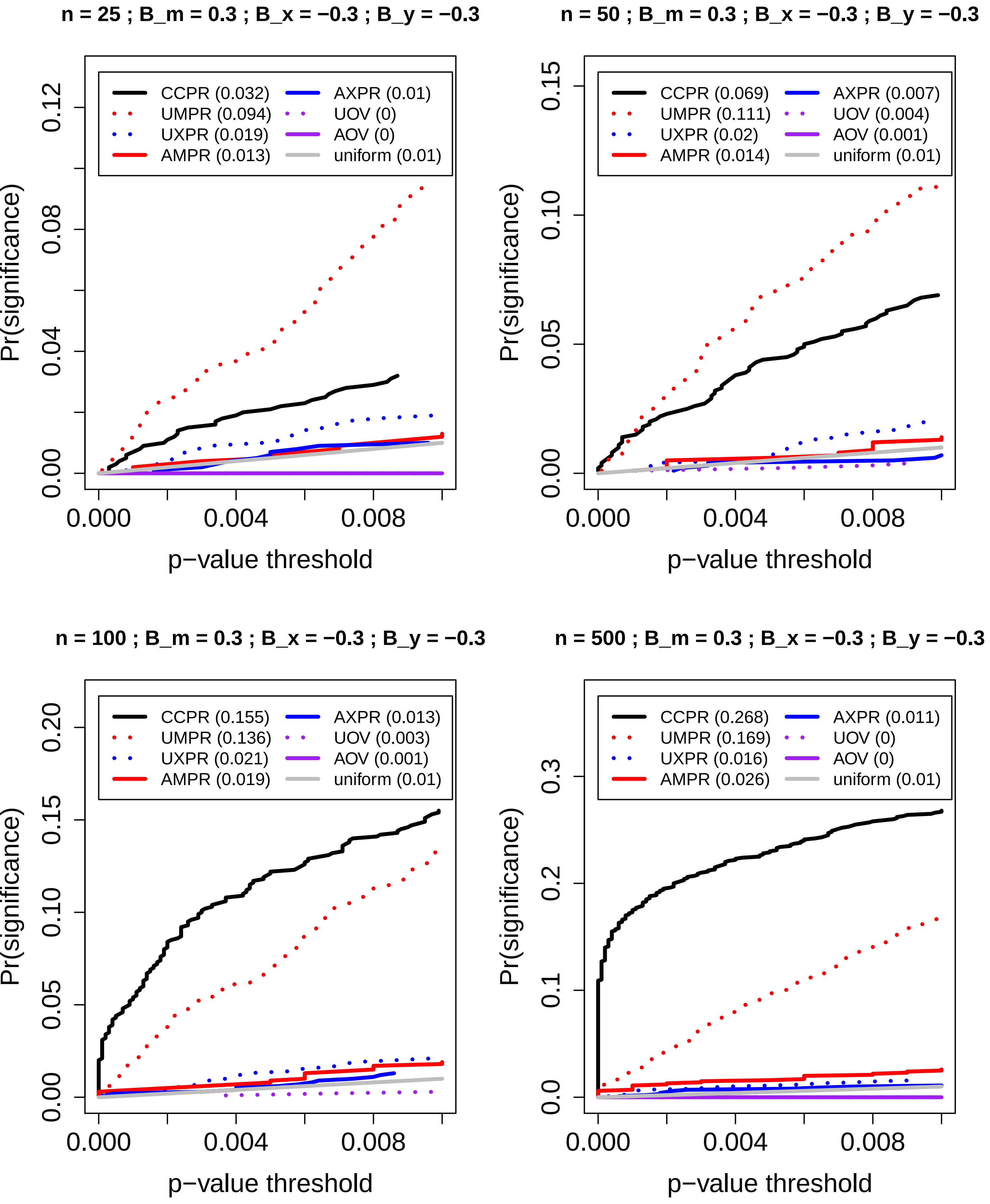
n = 25 ; B_m = 0.3 ; B_x = −0.3 ; B_y = −0.3
CCPR (0.032)
UMPR (0.094)
UXPR (0.019)
AMPR (0.013)
AXPR (0.01)
UOV (0)
AOV (0)
uniform (0.01)
Pr(significance)
p−value threshold
n = 50 ; B_m = 0.3 ; B_x = −0.3 ; B_y = −0.3
CCPR (0.069)
UMPR (0.111)
UXPR (0.02)
AMPR (0.014)
AXPR (0.007)
UOV (0.004)
AOV (0.001)
uniform (0.01)
Pr(significance)
p−value threshold
n = 100 ; B_m = 0.3 ; B_x = −0.3 ; B_y = −0.3
CCPR (0.155)
UMPR (0.136)
UXPR (0.021)
AMPR (0.019)
AXPR (0.013)
UOV (0.003)
AOV (0.001)
uniform (0.01)
Pr(significance)
p−value threshold
n = 500 ; B_m = 0.3 ; B_x = −0.3 ; B_y = −0.3
CCPR (0.268)
UMPR (0.169)
UXPR (0.016)
AMPR (0.026)
AXPR (0.011)
UOV (0)
AOV (0)
uniform (0.01)
Pr(significance)
p−value threshold

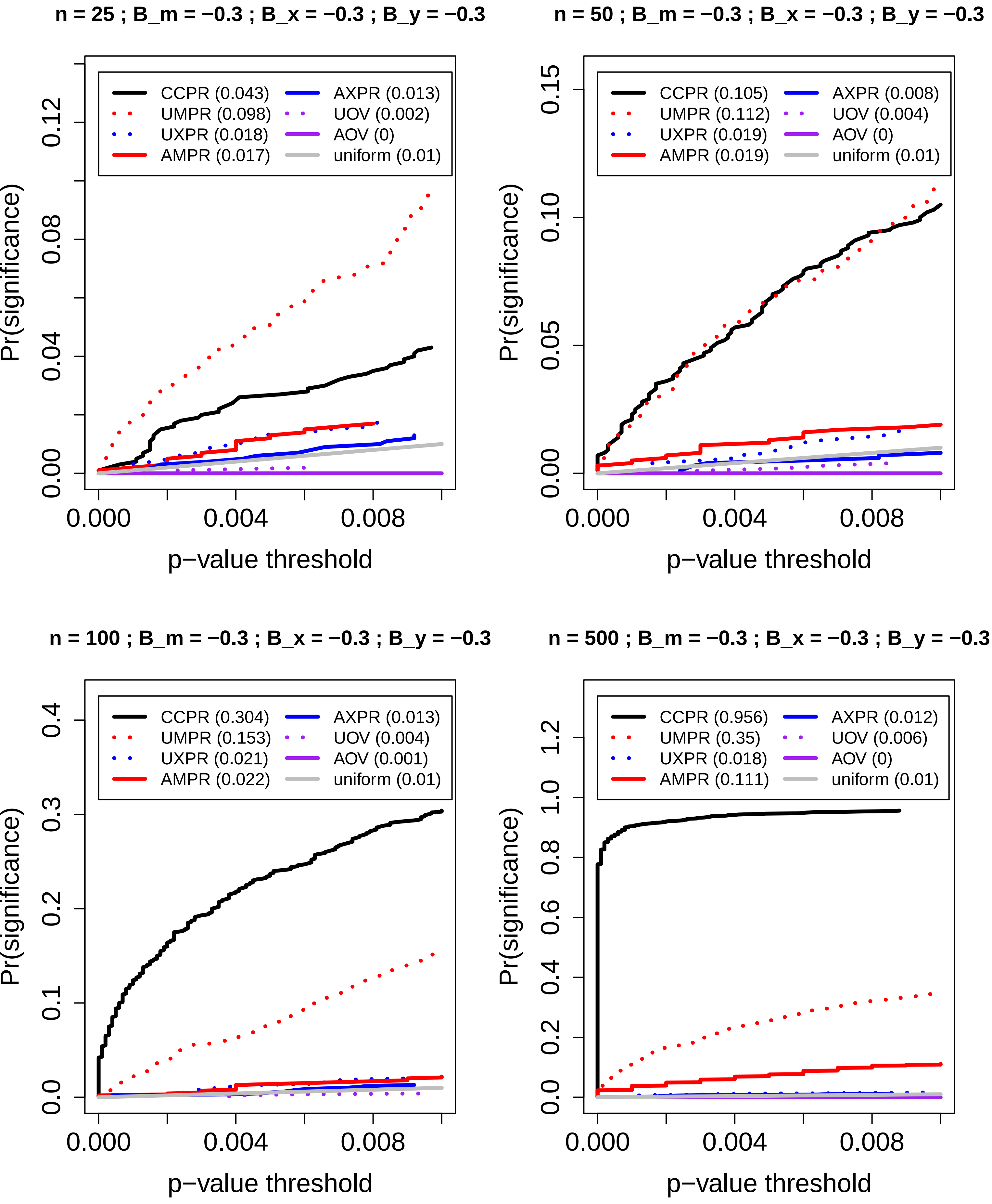
n = 25 ; B_m = −0.3 ; B_x = −0.3 ; B_y = −0.3
CCPR (0.043)
UMPR (0.098)
UXPR (0.018)
AMPR (0.017)
AXPR (0.013)
UOV (0.002)
AOV (0)
uniform (0.01)
Pr(significance)
p−value threshold
n = 50 ; B_m = −0.3 ; B_x = −0.3 ; B_y = −0.3
CCPR (0.105)
UMPR (0.112)
UXPR (0.019)
AMPR (0.019)
AXPR (0.008)
UOV (0.004)
AOV (0)
uniform (0.01)
Pr(significance)
p−value threshold
n = 100 ; B_m = −0.3 ; B_x = −0.3 ; B_y = −0.3
CCPR (0.304)
UMPR (0.153)
UXPR (0.021)
AMPR (0.022)
AXPR (0.013)
UOV (0.004)
AOV (0.001)
uniform (0.01)
Pr(significance)
p−value threshold
n = 500 ; B_m = −0.3 ; B_x = −0.3 ; B_y = −0.3
CCPR (0.956)
UMPR (0.35)
UXPR (0.018)
AMPR (0.111)
AXPR (0.012)
UOV (0.006)
AOV (0)
uniform (0.01)
Pr(significance)
p−value threshold

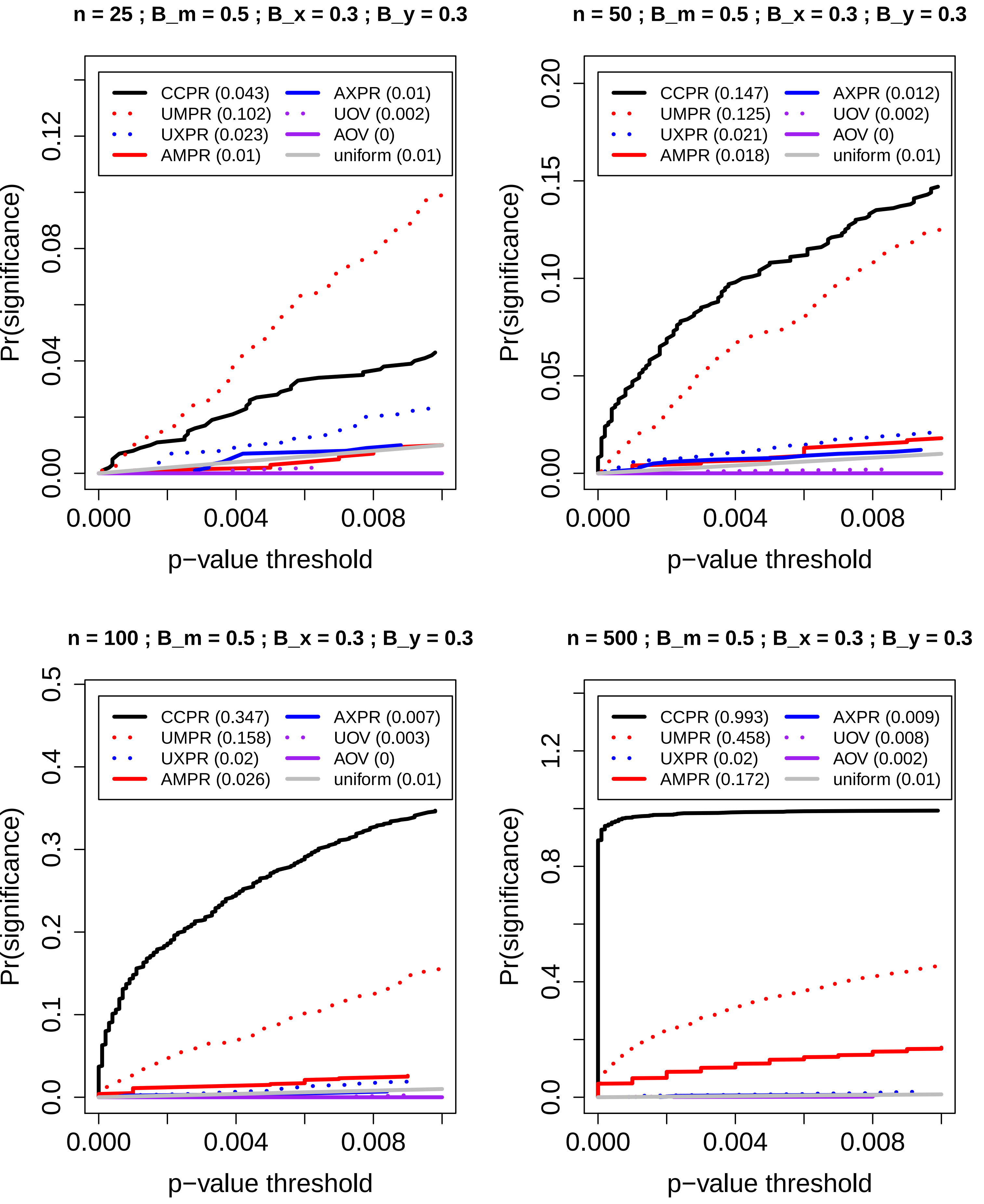

n = 25 ; B_m = 0.5 ; B_x = 0.3 ; B_y = 0.3
CCPR (0.043)
UMPR (0.102)
UXPR (0.023)
AMPR (0.01)
AXPR (0.01)
UOV (0.002)
AOV (0)
uniform (0.01)
n = 50 ; B_m = 0.5 ; B_x = 0.3 ; B_y = 0.3
CCPR (0.147)
UMPR (0.125)
UXPR (0.021)
AMPR (0.018)
AXPR (0.012)
UOV (0.002)
AOV (0)
uniform (0.01)
n = 100 ; B_m = 0.5 ; B_x = 0.3 ; B_y = 0.3
CCPR (0.347)
UMPR (0.158)
UXPR (0.02)
AMPR (0.026)
AXPR (0.007)
UOV (0.003)
AOV (0)
uniform (0.01)
n = 500 ; B_m = 0.5 ; B_x = 0.3 ; B_y = 0.3
CCPR (0.993)
UMPR (0.458)
UXPR (0.02)
AMPR (0.172)
AXPR (0.009)
UOV (0.008)
AOV (0.002)
uniform (0.01)
Pr(significance)
p−value threshold

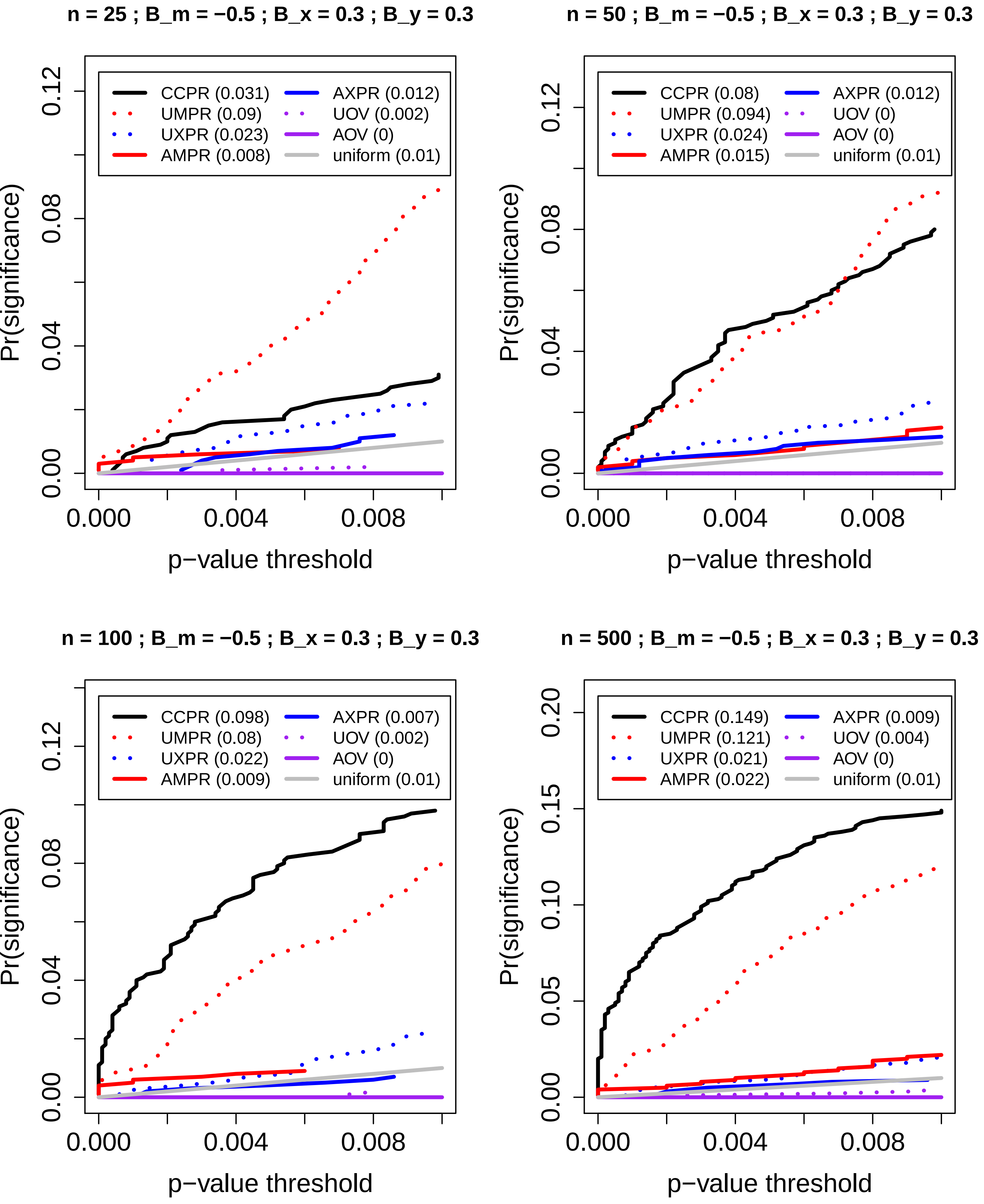

n = 25 ; B_m = −0.5 ; B_x = 0.3 ; B_y = 0.3
CCPR (0.031)
UMPR (0.09)
UXPR (0.023)
AMPR (0.008)
AXPR (0.012)
UOV (0.002)
AOV (0)
uniform (0.01)
n = 50 ; B_m = −0.5 ; B_x = 0.3 ; B_y = 0.3
CCPR (0.08)
UMPR (0.094)
UXPR (0.024)
AMPR (0.015)
AXPR (0.012)
UOV (0)
AOV (0)
uniform (0.01)
n = 100 ; B_m = −0.5 ; B_x = 0.3 ; B_y = 0.3
CCPR (0.098)
UMPR (0.08)
UXPR (0.022)
AMPR (0.009)
AXPR (0.007)
UOV (0.002)
AOV (0)
uniform (0.01)
n = 500 ; B_m = −0.5 ; B_x = 0.3 ; B_y = 0.3
CCPR (0.149)
UMPR (0.121)
UXPR (0.021)
AMPR (0.022)
AXPR (0.009)
UOV (0.004)
AOV (0)
uniform (0.01)
Pr(significance)
p−value threshold
0.00
0.04
0.08
0.12
0.05
0.10
0.15
0.20
0.000
0.004
0.008

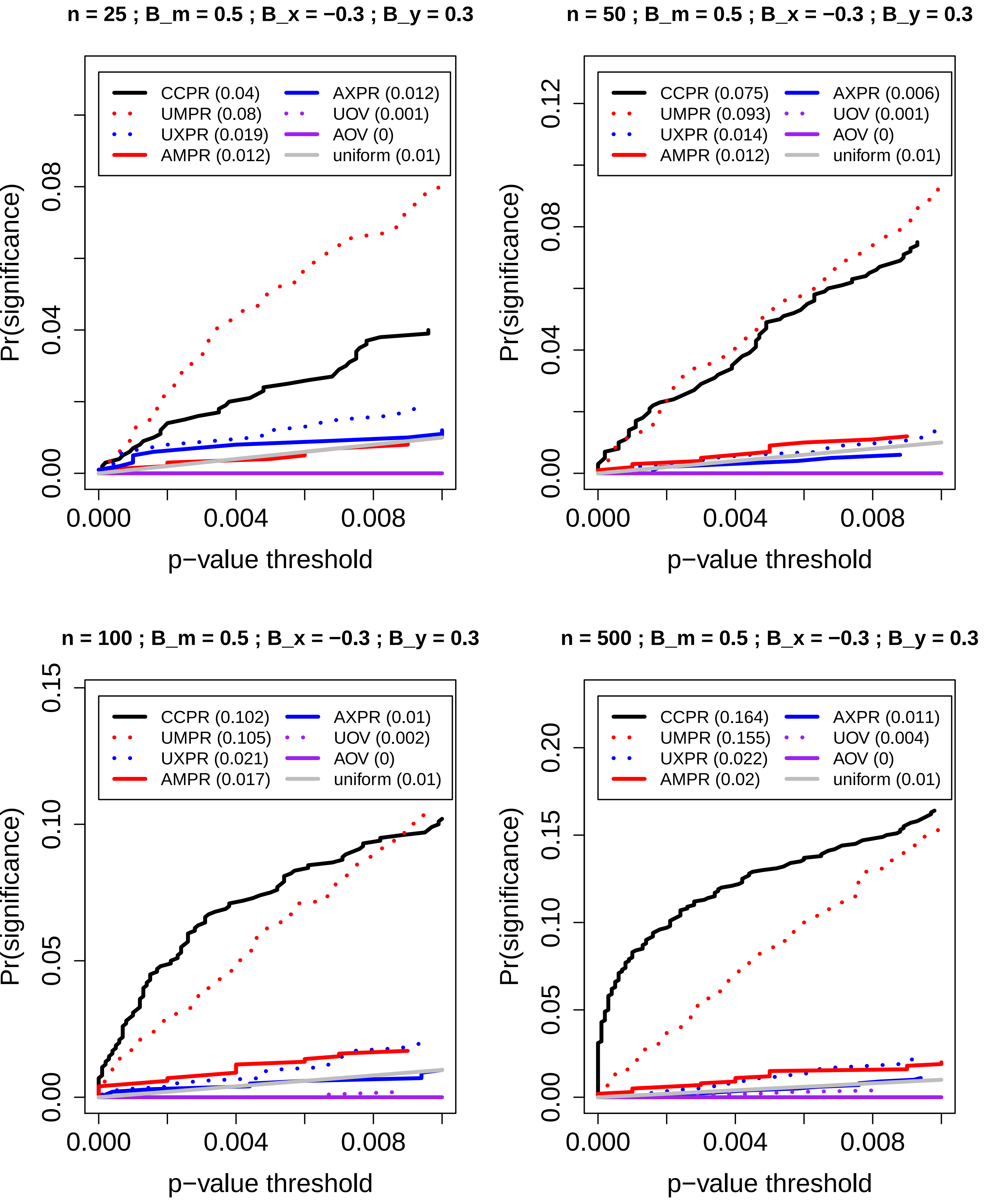

n = 25 ; B_m = 0.5 ; B_x = −0.3 ; B_y = 0.3
CCPR (0.04)
UMPR (0.08)
UXPR (0.019)
AMPR (0.012)
AXPR (0.012)
UOV (0.001)
AOV (0)
uniform (0.01)
n = 50 ; B_m = 0.5 ; B_x = −0.3 ; B_y = 0.3
CCPR (0.075)
UMPR (0.093)
UXPR (0.014)
AMPR (0.012)
AXPR (0.006)
UOV (0.001)
AOV (0)
uniform (0.01)
n = 100 ; B_m = 0.5 ; B_x = −0.3 ; B_y = 0.3
CCPR (0.102)
UMPR (0.105)
UXPR (0.021)
AMPR (0.017)
AXPR (0.01)
UOV (0.002)
AOV (0)
uniform (0.01)
n = 500 ; B_m = 0.5 ; B_x = −0.3 ; B_y = 0.3
CCPR (0.164)
UMPR (0.155)
UXPR (0.022)
AMPR (0.02)
AXPR (0.011)
UOV (0.004)
AOV (0)
uniform (0.01)
Pr(significance)
p−value threshold

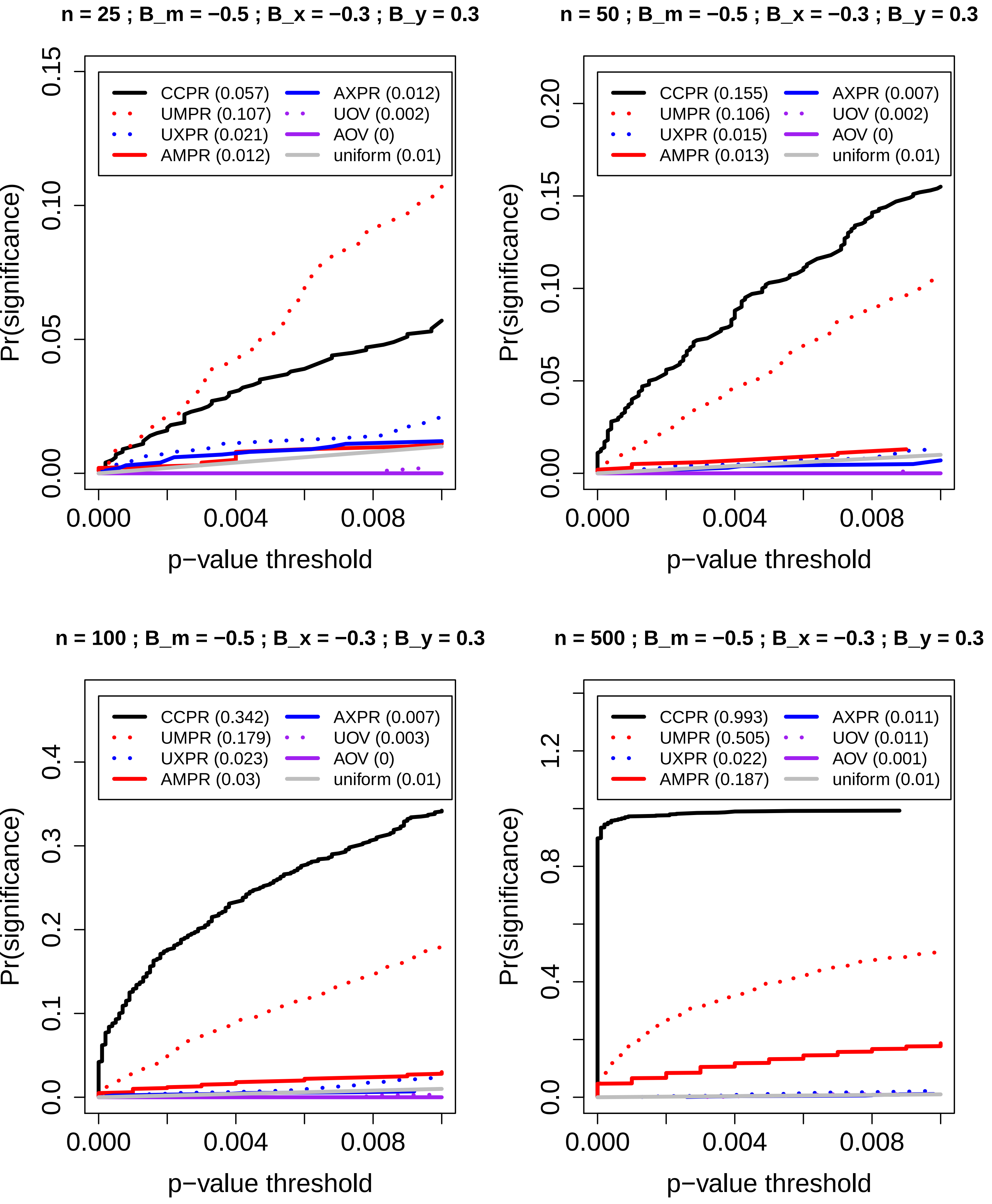

n = 25 ; B_m = −0.5 ; B_x = −0.3 ; B_y = 0.3
CCPR (0.057)
UMPR (0.107)
UXPR (0.021)
AMPR (0.012)
AXPR (0.012)
UOV (0.002)
AOV (0)
uniform (0.01)
Pr(significance)
p−value threshold
0.00
0.05
0.10
0.15
0.000
0.004
0.008
n = 50 ; B_m = −0.5 ; B_x = −0.3 ; B_y = 0.3
CCPR (0.155)
UMPR (0.106)
UXPR (0.015)
AMPR (0.013)
AXPR (0.007)
UOV (0.002)
AOV (0)
uniform (0.01)
Pr(significance)
p−value threshold
0.00
0.05
0.10
0.15
0.20
0.000
0.004
0.008
n = 100 ; B_m = −0.5 ; B_x = −0.3 ; B_y = 0.3
CCPR (0.342)
UMPR (0.179)
UXPR (0.023)
AMPR (0.03)
AXPR (0.007)
UOV (0.003)
AOV (0)
uniform (0.01)
Pr(significance)
p−value threshold
0.0
0.1
0.2
0.3
0.4
0.000
0.004
0.008
n = 500 ; B_m = −0.5 ; B_x = −0.3 ; B_y = 0.3
CCPR (0.993)
UMPR (0.505)
UXPR (0.022)
AMPR (0.187)
AXPR (0.011)
UOV (0.011)
AOV (0.001)
uniform (0.01)
Pr(significance)
p−value threshold
0.0
0.4
0.8
1.2
0.000
0.004
0.008

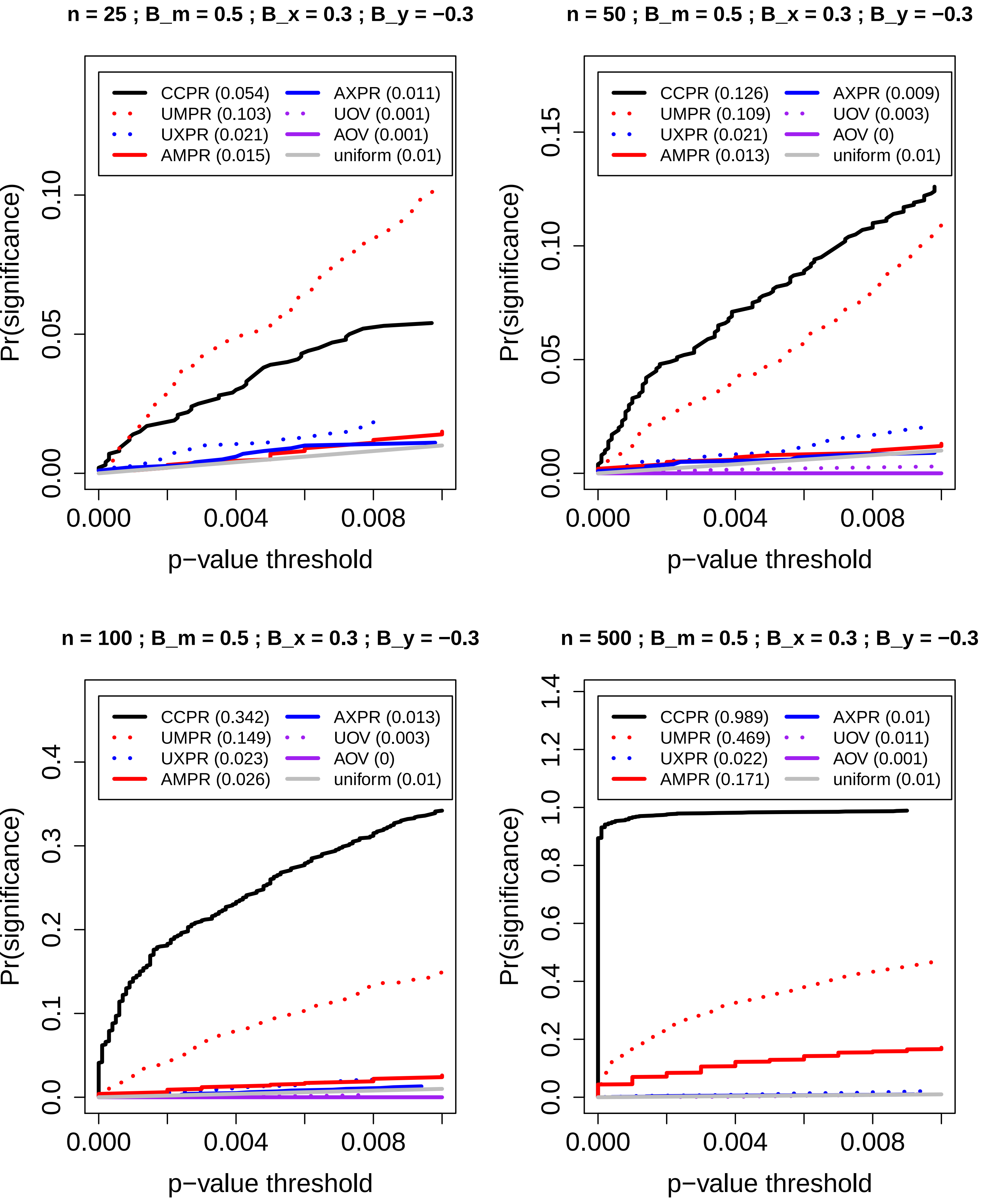

n = 25 ; B_m = 0.5 ; B_x = 0.3 ; B_y = −0.3
CCPR (0.054)
UMPR (0.103)
UXPR (0.021)
AMPR (0.015)
AXPR (0.011)
UOV (0.001)
AOV (0.001)
uniform (0.01)
Pr(significance)
0.00
0.05
0.10
0.000
0.004
0.008
p−value threshold
n = 50 ; B_m = 0.5 ; B_x = 0.3 ; B_y = −0.3
CCPR (0.126)
UMPR (0.109)
UXPR (0.021)
AMPR (0.013)
AXPR (0.009)
UOV (0.003)
AOV (0)
uniform (0.01)
Pr(significance)
0.00
0.05
0.10
0.15
0.000
0.004
0.008
p−value threshold
n = 100 ; B_m = 0.5 ; B_x = 0.3 ; B_y = −0.3
CCPR (0.342)
UMPR (0.149)
UXPR (0.023)
AMPR (0.026)
AXPR (0.013)
UOV (0.003)
AOV (0)
uniform (0.01)
Pr(significance)
0.0
0.1
0.2
0.3
0.4
0.000
0.004
0.008
p−value threshold
n = 500 ; B_m = 0.5 ; B_x = 0.3 ; B_y = −0.3
CCPR (0.989)
UMPR (0.469)
UXPR (0.022)
AMPR (0.171)
AXPR (0.01)
UOV (0.011)
AOV (0.001)
uniform (0.01)
Pr(significance)
0.0
0.2
0.4
0.6
0.8
1.0
1.2
1.4
0.000
0.004
0.008
p−value threshold

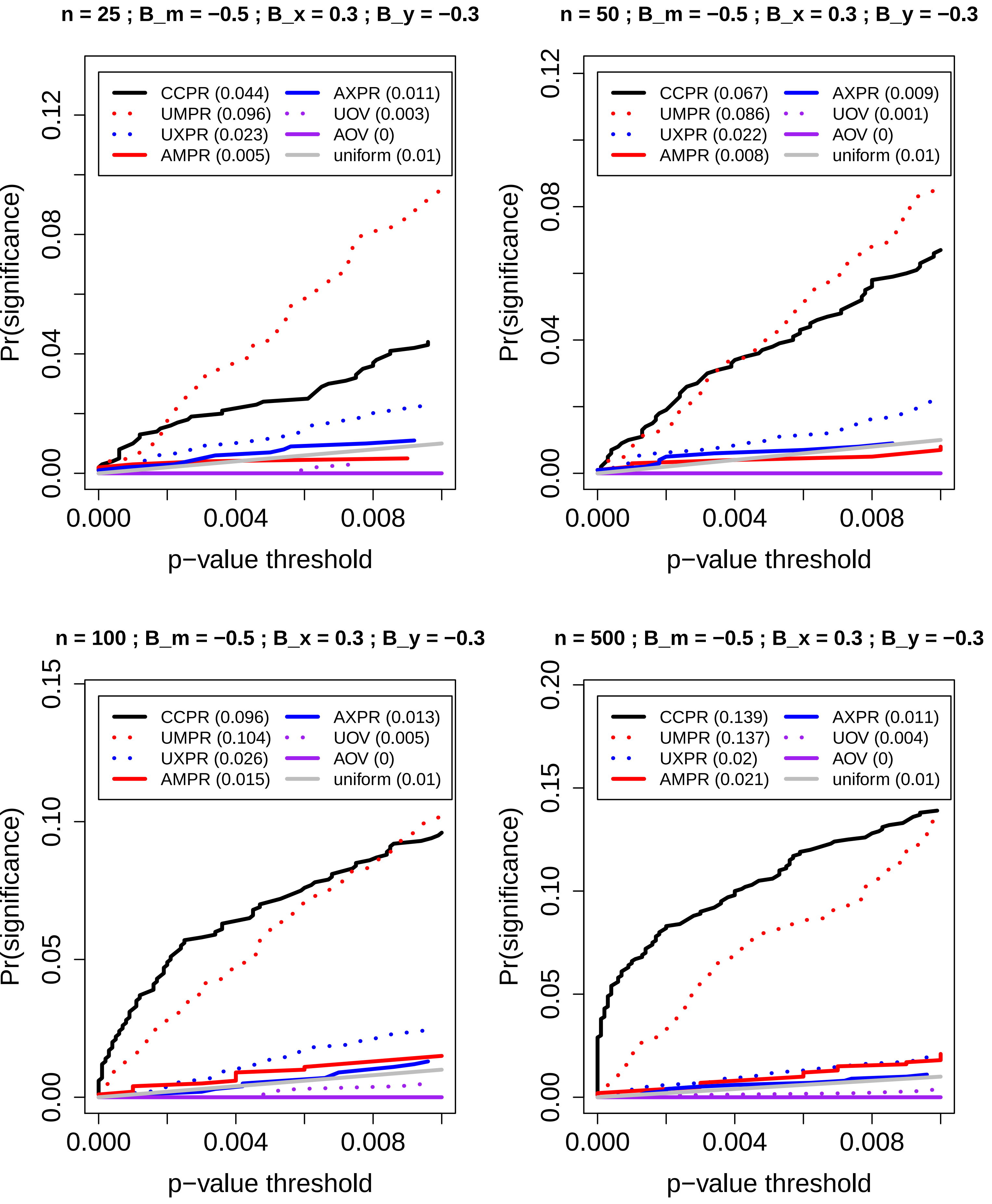

n = 25 ; B_m = −0.5 ; B_x = 0.3 ; B_y = −0.3
CCPR (0.044)
UMPR (0.096)
UXPR (0.023)
AMPR (0.005)
AXPR (0.011)
UOV (0.003)
AOV (0)
uniform (0.01)
n = 50 ; B_m = −0.5 ; B_x = 0.3 ; B_y = −0.3
CCPR (0.067)
UMPR (0.086)
UXPR (0.022)
AMPR (0.008)
AXPR (0.009)
UOV (0.001)
AOV (0)
uniform (0.01)
n = 100 ; B_m = −0.5 ; B_x = 0.3 ; B_y = −0.3
CCPR (0.096)
UMPR (0.104)
UXPR (0.026)
AMPR (0.015)
AXPR (0.013)
UOV (0.005)
AOV (0)
uniform (0.01)
n = 500 ; B_m = −0.5 ; B_x = 0.3 ; B_y = −0.3
CCPR (0.139)
UMPR (0.137)
UXPR (0.02)
AMPR (0.021)
AXPR (0.011)
UOV (0.004)
AOV (0)
uniform (0.01)
Pr(significance)
p−value threshold

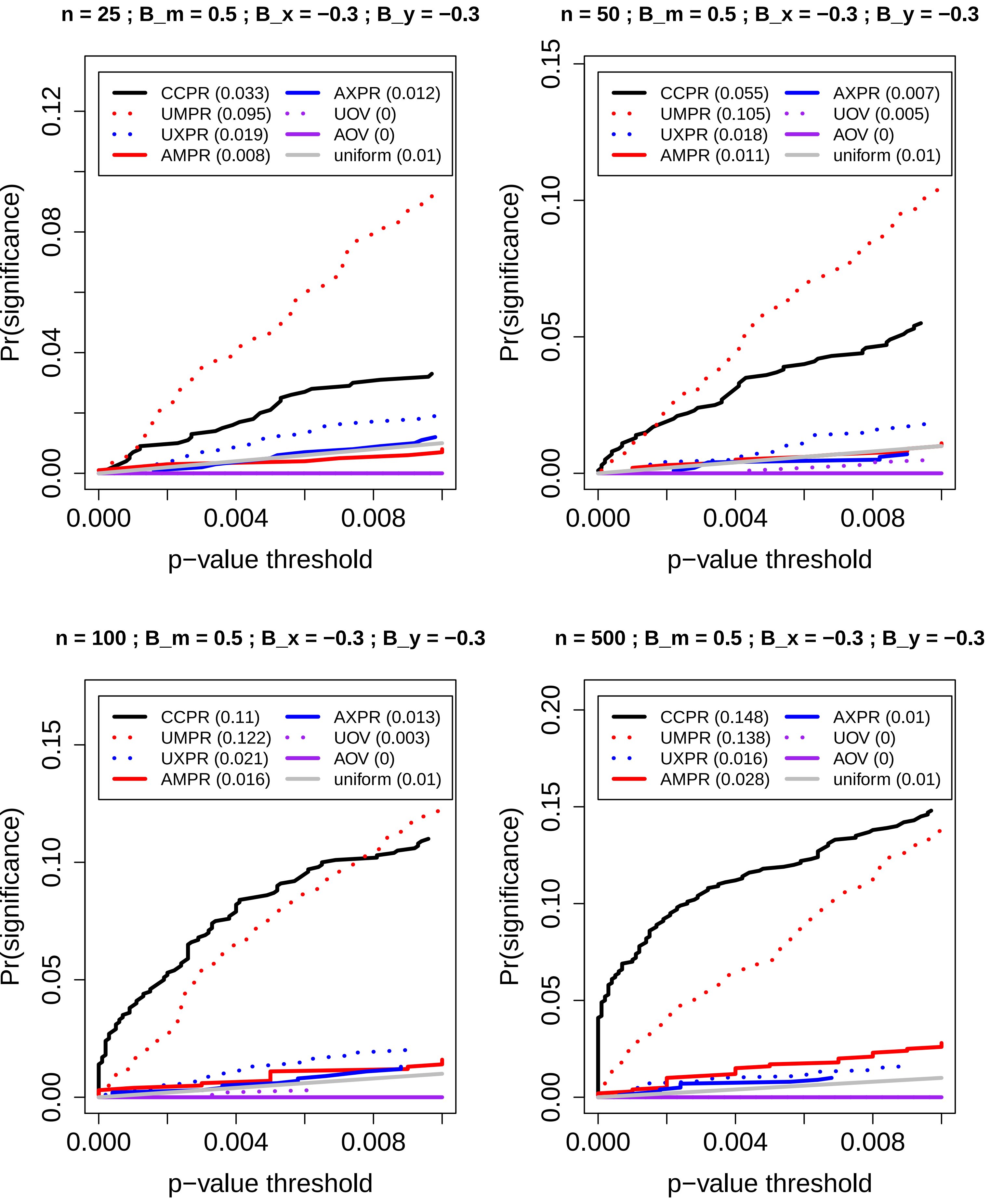

n = 25 ; B_m = 0.5 ; B_x = −0.3 ; B_y = −0.3
CCPR (0.033)
UMPR (0.095)
UXPR (0.019)
AMPR (0.008)
AXPR (0.012)
UOV (0)
AOV (0)
uniform (0.01)
Pr(significance)
p−value threshold
n = 50 ; B_m = 0.5 ; B_x = −0.3 ; B_y = −0.3
CCPR (0.055)
UMPR (0.105)
UXPR (0.018)
AMPR (0.011)
AXPR (0.007)
UOV (0.005)
AOV (0)
uniform (0.01)
n = 100 ; B_m = 0.5 ; B_x = −0.3 ; B_y = −0.3
CCPR (0.11)
UMPR (0.122)
UXPR (0.021)
AMPR (0.016)
AXPR (0.013)
UOV (0.003)
AOV (0)
uniform (0.01)
n = 500 ; B_m = 0.5 ; B_x = −0.3 ; B_y = −0.3
CCPR (0.148)
UMPR (0.138)
UXPR (0.016)
AMPR (0.028)
AXPR (0.01)
UOV (0)
AOV (0)
uniform (0.01)

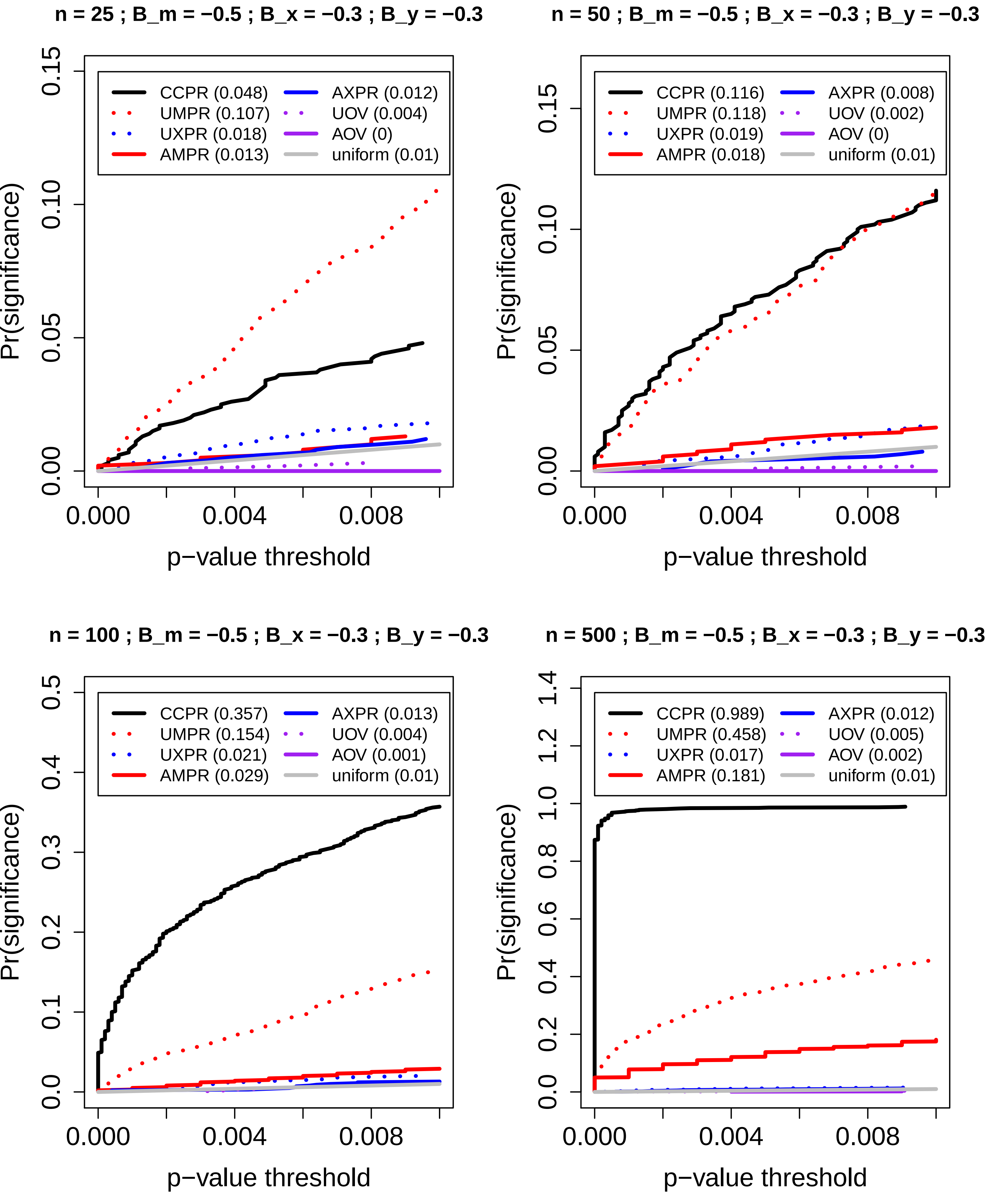

n = 25 ; B_m = −0.5 ; B_x = −0.3 ; B_y = −0.3
CCPR (0.048)
UMPR (0.107)
UXPR (0.018)
AMPR (0.013)
AXPR (0.012)
UOV (0.004)
AOV (0)
uniform (0.01)
n = 50 ; B_m = −0.5 ; B_x = −0.3 ; B_y = −0.3
CCPR (0.116)
UMPR (0.118)
UXPR (0.019)
AMPR (0.018)
AXPR (0.008)
UOV (0.002)
AOV (0)
uniform (0.01)
n = 100 ; B_m = −0.5 ; B_x = −0.3 ; B_y = −0.3
CCPR (0.357)
UMPR (0.154)
UXPR (0.021)
AMPR (0.029)
AXPR (0.013)
UOV (0.004)
AOV (0.001)
uniform (0.01)
n = 500 ; B_m = −0.5 ; B_x = −0.3 ; B_y = −0.3
CCPR (0.989)
UMPR (0.458)
UXPR (0.017)
AMPR (0.181)
AXPR (0.012)
UOV (0.005)
AOV (0.002)
uniform (0.01)
Pr(significance)
p−value threshold
0.000
0.004
0.008
0.00
0.05
0.10
0.15
0.0
0.1
0.2
0.3
0.4
0.5
0.6
0.8
1.0
1.2
1.4

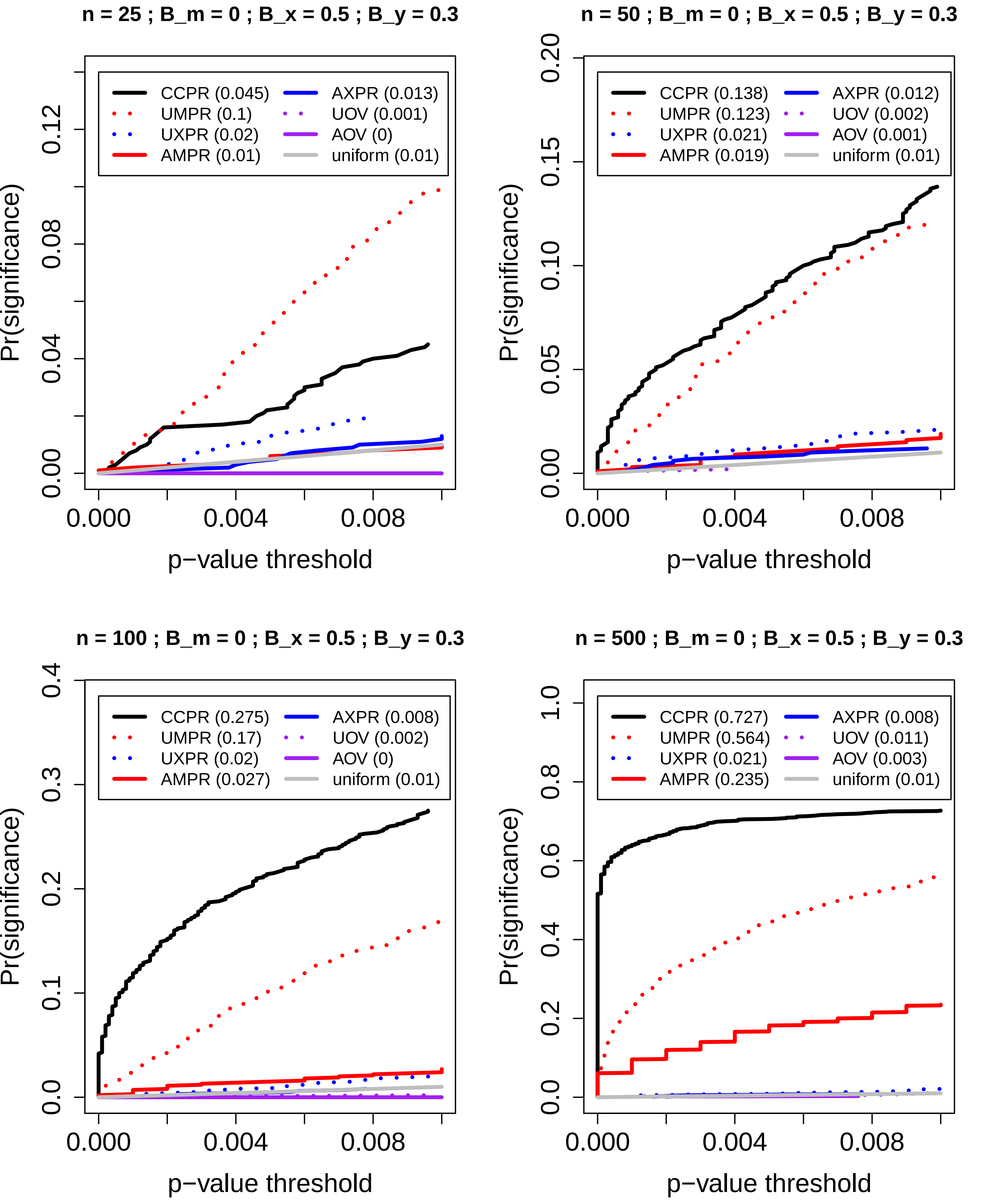

n = 25 ; B_m = 0 ; B_x = 0.5 ; B_y = 0.3
CCPR (0.045)
UMPR (0.1)
UXPR (0.02)
AMPR (0.01)
AXPR (0.013)
UOV (0.001)
AOV (0)
uniform (0.01)
n = 50 ; B_m = 0 ; B_x = 0.5 ; B_y = 0.3
CCPR (0.138)
UMPR (0.123)
UXPR (0.021)
AMPR (0.019)
AXPR (0.012)
UOV (0.002)
AOV (0.001)
uniform (0.01)
n = 100 ; B_m = 0 ; B_x = 0.5 ; B_y = 0.3
CCPR (0.275)
UMPR (0.17)
UXPR (0.02)
AMPR (0.027)
AXPR (0.008)
UOV (0.002)
AOV (0)
uniform (0.01)
n = 500 ; B_m = 0 ; B_x = 0.5 ; B_y = 0.3
CCPR (0.727)
UMPR (0.564)
UXPR (0.021)
AMPR (0.235)
AXPR (0.008)
UOV (0.011)
AOV (0.003)
uniform (0.01)
Pr(significance)
p−value threshold

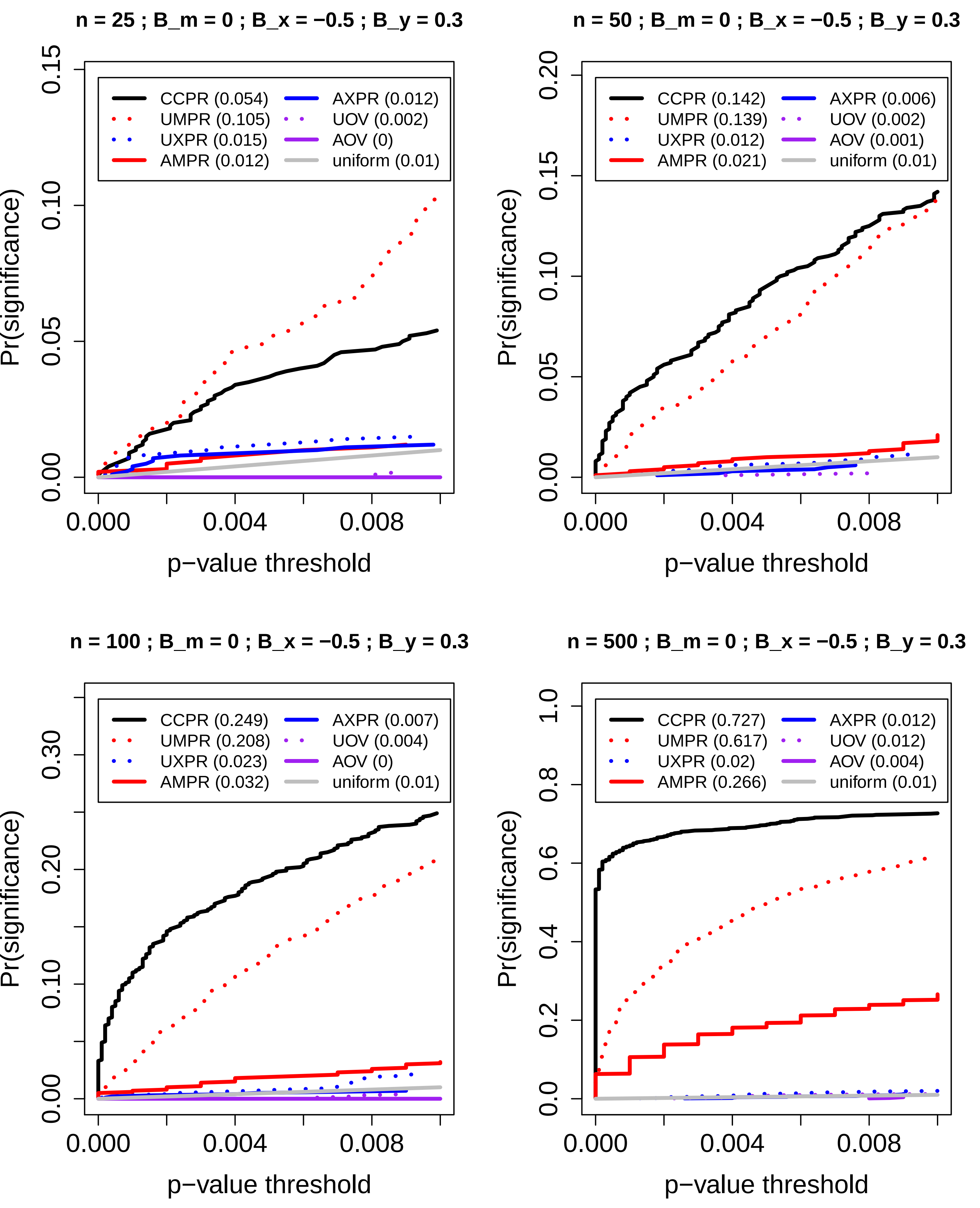
n = 25 ; B_m = 0 ; B_x = −0.5 ; B_y = 0.3
CCPR (0.054)
UMPR (0.105)
UXPR (0.015)
AMPR (0.012)
AXPR (0.012)
UOV (0.002)
AOV (0)
uniform (0.01)
n = 50 ; B_m = 0 ; B_x = −0.5 ; B_y = 0.3
CCPR (0.142)
UMPR (0.139)
UXPR (0.012)
AMPR (0.021)
AXPR (0.006)
UOV (0.002)
AOV (0.001)
uniform (0.01)
n = 100 ; B_m = 0 ; B_x = −0.5 ; B_y = 0.3
CCPR (0.249)
UMPR (0.208)
UXPR (0.023)
AMPR (0.032)
AXPR (0.007)
UOV (0.004)
AOV (0)
uniform (0.01)
n = 500 ; B_m = 0 ; B_x = −0.5 ; B_y = 0.3
CCPR (0.727)
UMPR (0.617)
UXPR (0.02)
AMPR (0.266)
AXPR (0.012)
UOV (0.012)
AOV (0.004)
uniform (0.01)
Pr(significance)
p−value threshold
0.000
0.004
0.008
0.00
0.05
0.10
0.15
0.20
0.30
0.0
0.2
0.4
0.6
0.8
1.0

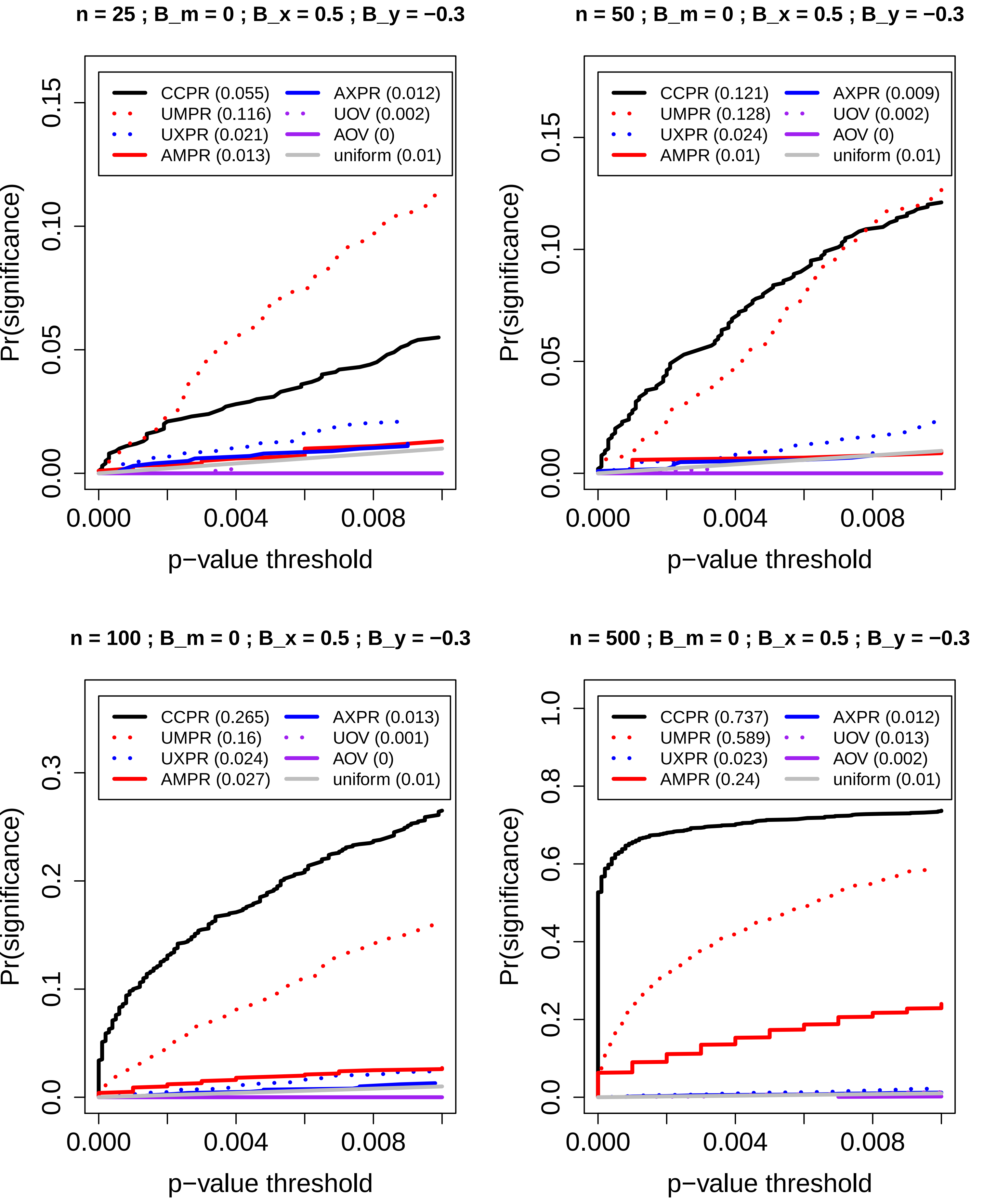
n = 25 ; B_m = 0 ; B_x = 0.5 ; B_y = −0.3
CCPR (0.055)
UMPR (0.116)
UXPR (0.021)
AMPR (0.013)
AXPR (0.012)
UOV (0.002)
AOV (0)
uniform (0.01)
n = 50 ; B_m = 0 ; B_x = 0.5 ; B_y = −0.3
CCPR (0.121)
UMPR (0.128)
UXPR (0.024)
AMPR (0.01)
AXPR (0.009)
UOV (0.002)
AOV (0)
uniform (0.01)
n = 100 ; B_m = 0 ; B_x = 0.5 ; B_y = −0.3
CCPR (0.265)
UMPR (0.16)
UXPR (0.024)
AMPR (0.027)
AXPR (0.013)
UOV (0.001)
AOV (0)
uniform (0.01)
n = 500 ; B_m = 0 ; B_x = 0.5 ; B_y = −0.3
CCPR (0.737)
UMPR (0.589)
UXPR (0.023)
AMPR (0.24)
AXPR (0.012)
UOV (0.013)
AOV (0.002)
uniform (0.01)
Pr(significance)
p−value threshold
0.000
0.004
0.008
0.00
0.05
0.10
0.15
0.0
0.1
0.2
0.3
0.2
0.4
0.6
0.8
1.0

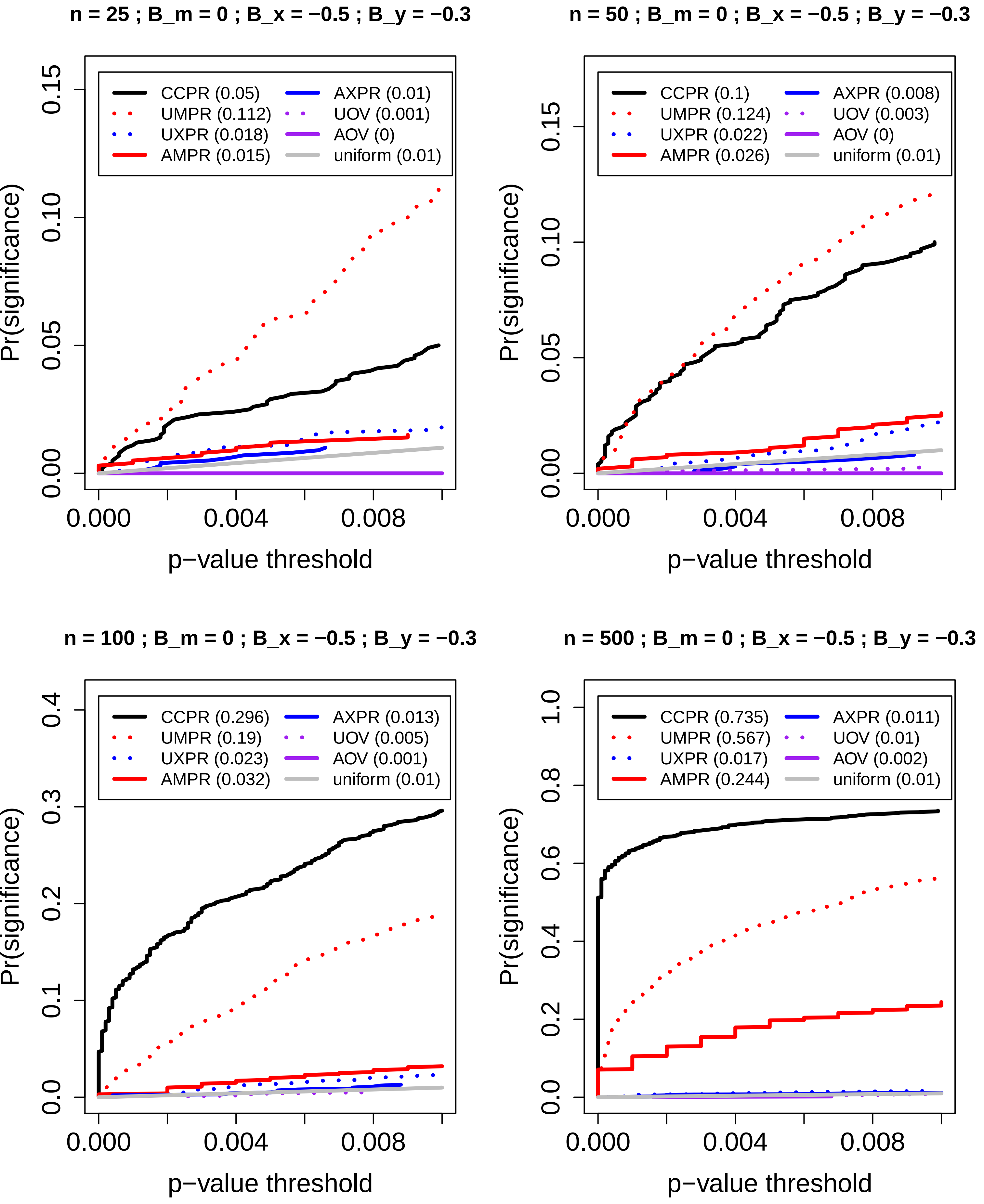
n = 25 ; B_m = 0 ; B_x = −0.5 ; B_y = −0.3
CCPR (0.05)
UMPR (0.112)
UXPR (0.018)
AMPR (0.015)
AXPR (0.01)
UOV (0.001)
AOV (0)
uniform (0.01)
n = 50 ; B_m = 0 ; B_x = −0.5 ; B_y = −0.3
CCPR (0.1)
UMPR (0.124)
UXPR (0.022)
AMPR (0.026)
AXPR (0.008)
UOV (0.003)
AOV (0)
uniform (0.01)
n = 100 ; B_m = 0 ; B_x = −0.5 ; B_y = −0.3
CCPR (0.296)
UMPR (0.19)
UXPR (0.023)
AMPR (0.032)
AXPR (0.013)
UOV (0.005)
AOV (0.001)
uniform (0.01)
n = 500 ; B_m = 0 ; B_x = −0.5 ; B_y = −0.3
CCPR (0.735)
UMPR (0.567)
UXPR (0.017)
AMPR (0.244)
AXPR (0.011)
UOV (0.01)
AOV (0.002)
uniform (0.01)
Pr(significance)
p−value threshold

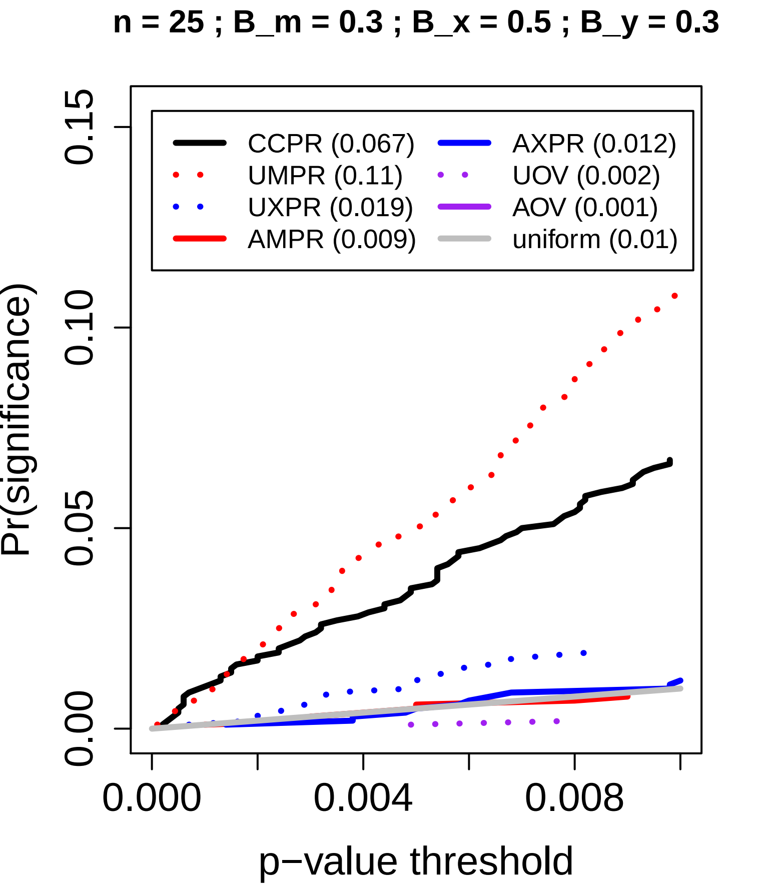

n = 25 ; B_m = 0.3 ; B_x = 0.5 ; B_y = 0.3
CCPR (0.067)
UMPR (0.11)
UXPR (0.019)
AMPR (0.009)
AXPR (0.012)
UOV (0.002)
AOV (0.001)
uniform (0.01)
Pr(significance)
0.00
0.05
0.10
0.15
0.000
0.004
0.008
p−value threshold

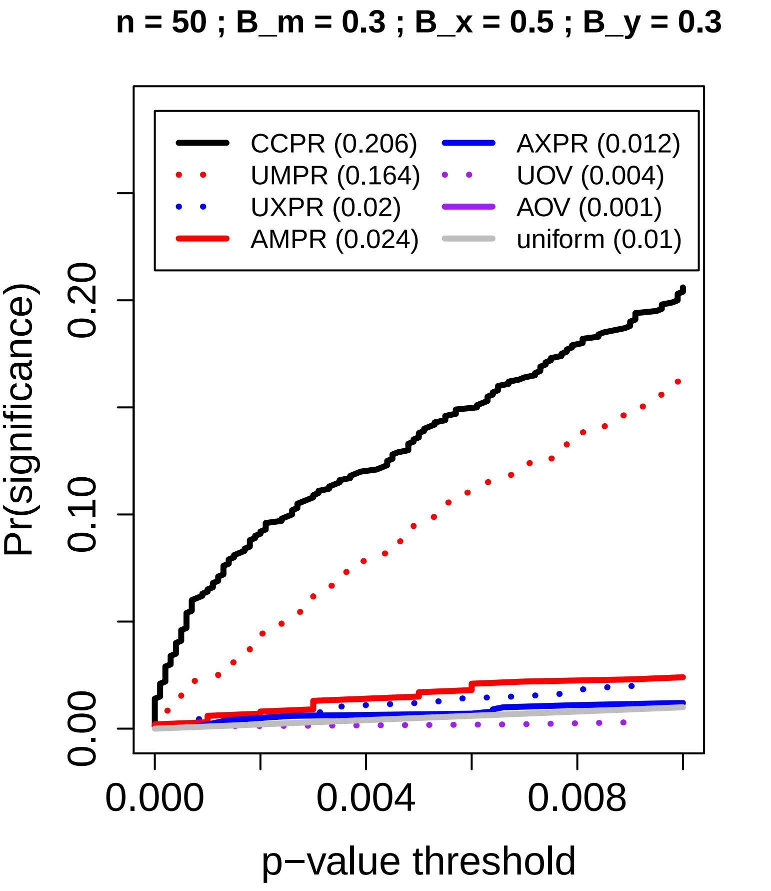

n = 50 ; B_m = 0.3 ; B_x = 0.5 ; B_y = 0.3
CCPR (0.206)
UMPR (0.164)
UXPR (0.02)
AMPR (0.024)
AXPR (0.012)
UOV (0.004)
AOV (0.001)
uniform (0.01)
Pr(significance)
0.00
0.10
0.20
0.000
0.004
0.008
p−value threshold

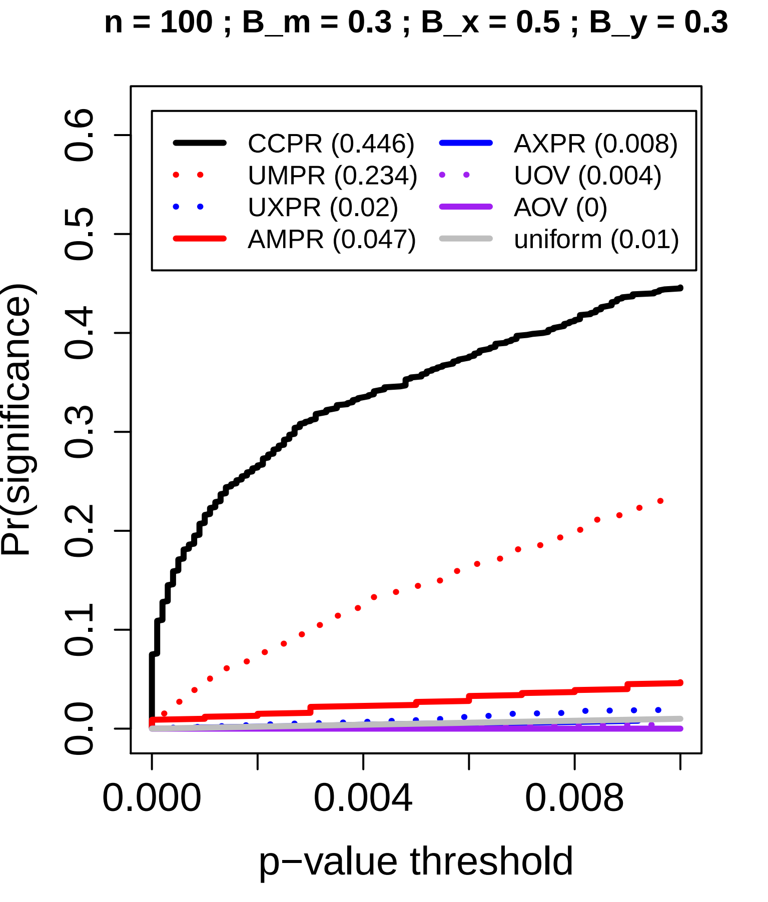

n = 100 ; B_m = 0.3 ; B_x = 0.5 ; B_y = 0.3
CCPR (0.446)
UMPR (0.234)
UXPR (0.02)
AMPR (0.047)
AXPR (0.008)
UOV (0.004)
AOV (0)
uniform (0.01)
Pr(significance)
0.0
0.1
0.2
0.3
0.4
0.5
0.6
0.000
0.004
0.008
p−value threshold

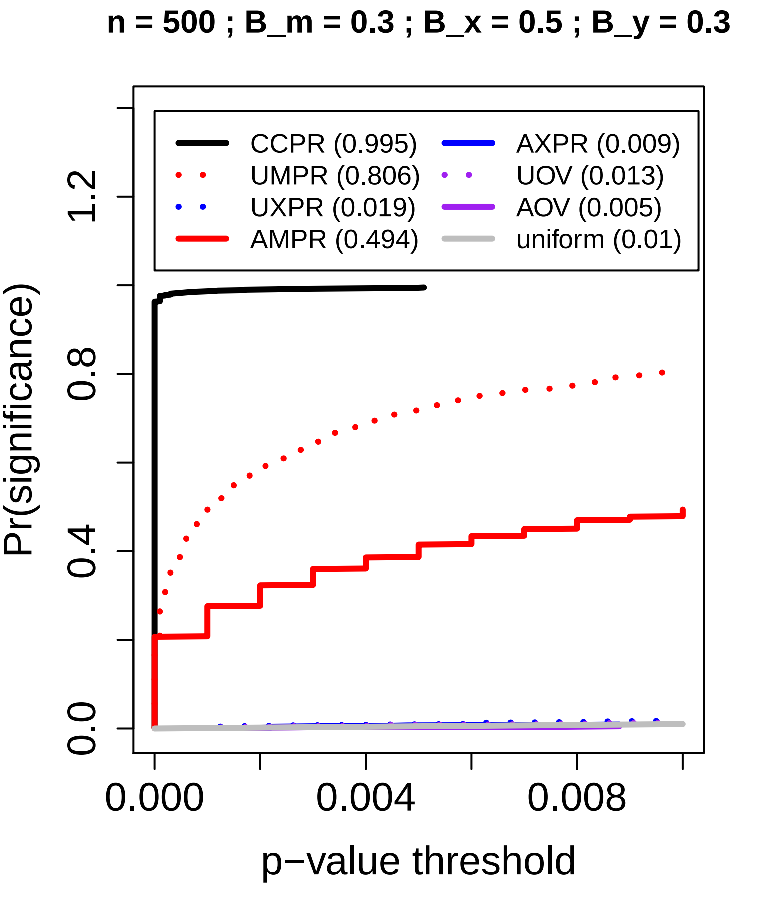

n = 500 ; B_m = 0.3 ; B_x = 0.5 ; B_y = 0.3
CCPR (0.995)
UMPR (0.806)
UXPR (0.019)
AMPR (0.494)
AXPR (0.009)
UOV (0.013)
AOV (0.005)
uniform (0.01)
Pr(significance)
0.0
0.4
0.8
1.2
0.000
0.004
0.008
p−value threshold

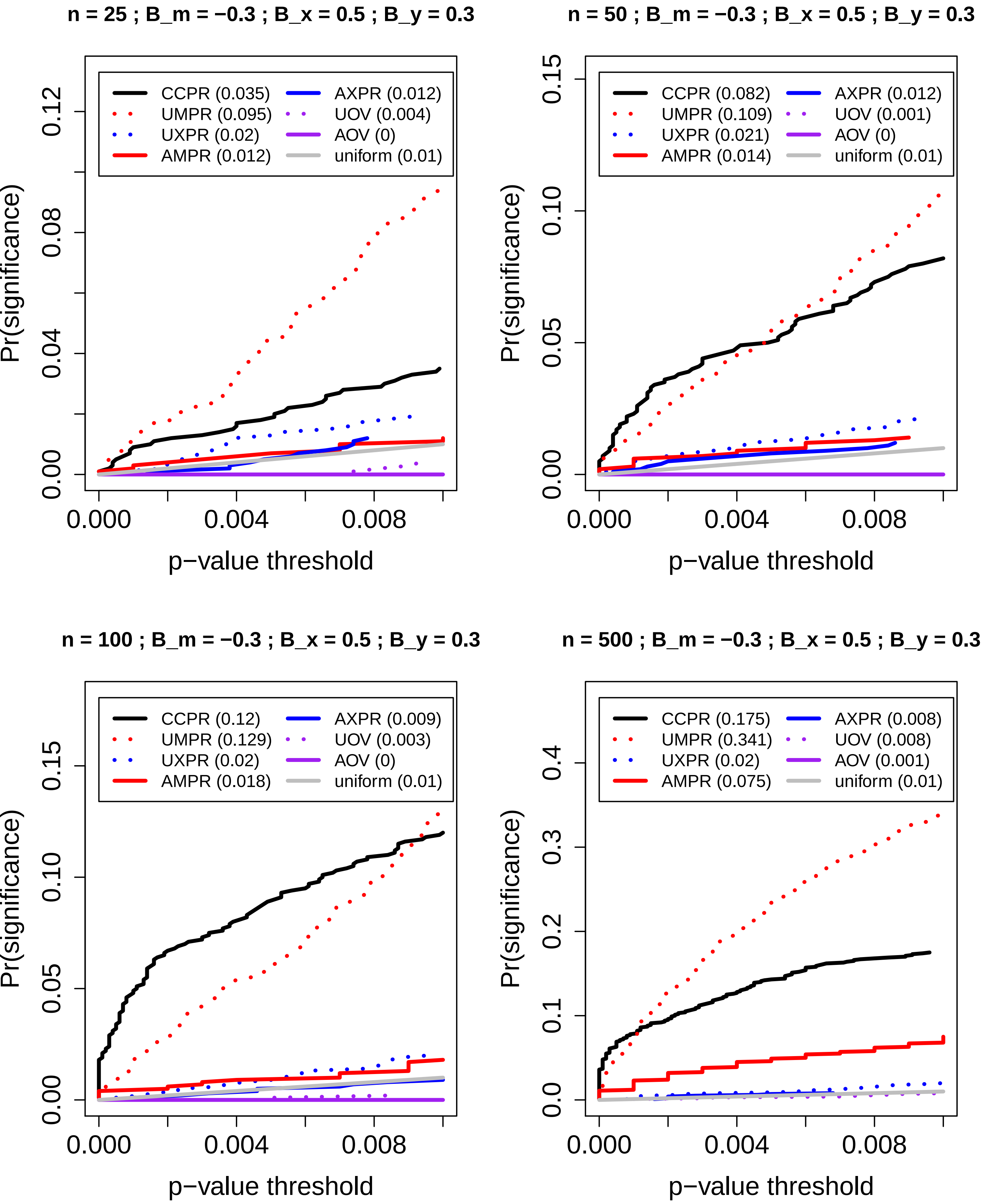

n = 25 ; B_m = −0.3 ; B_x = 0.5 ; B_y = 0.3
CCPR (0.035)
UMPR (0.095)
UXPR (0.02)
AMPR (0.012)
AXPR (0.012)
UOV (0.004)
AOV (0)
uniform (0.01)
n = 50 ; B_m = −0.3 ; B_x = 0.5 ; B_y = 0.3
CCPR (0.082)
UMPR (0.109)
UXPR (0.021)
AMPR (0.014)
AXPR (0.012)
UOV (0.001)
AOV (0)
uniform (0.01)
n = 100 ; B_m = −0.3 ; B_x = 0.5 ; B_y = 0.3
CCPR (0.12)
UMPR (0.129)
UXPR (0.02)
AMPR (0.018)
AXPR (0.009)
UOV (0.003)
AOV (0)
uniform (0.01)
n = 500 ; B_m = −0.3 ; B_x = 0.5 ; B_y = 0.3
CCPR (0.175)
UMPR (0.341)
UXPR (0.02)
AMPR (0.075)
AXPR (0.008)
UOV (0.008)
AOV (0.001)
uniform (0.01)
Pr(significance)
p−value threshold

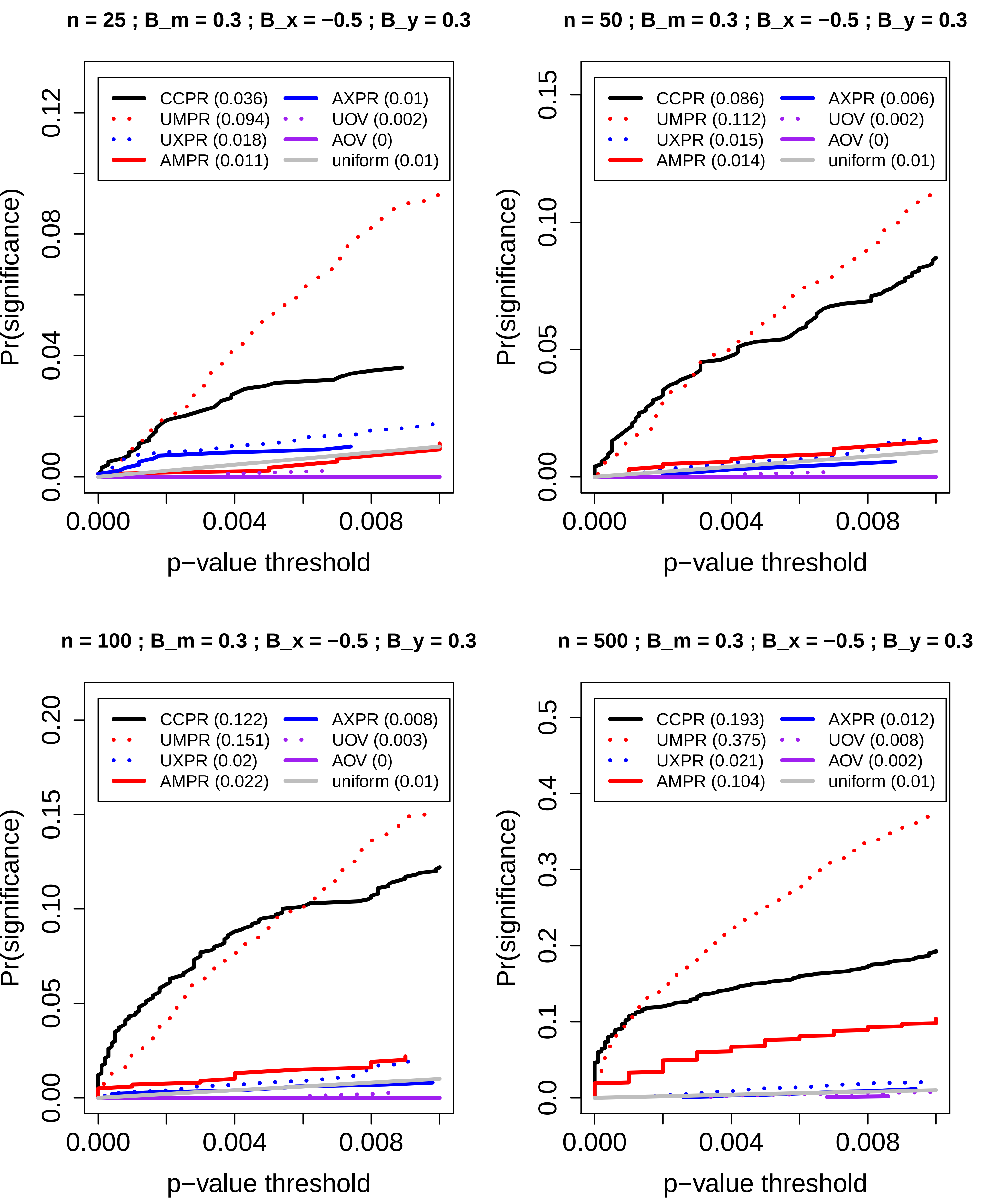
n = 25 ; B_m = 0.3 ; B_x = −0.5 ; B_y = 0.3
CCPR (0.036)
UMPR (0.094)
UXPR (0.018)
AMPR (0.011)
AXPR (0.01)
UOV (0.002)
AOV (0)
uniform (0.01)
Pr(significance)
0.00
0.04
0.08
0.12
0.000
0.004
0.008
p−value threshold
n = 50 ; B_m = 0.3 ; B_x = −0.5 ; B_y = 0.3
CCPR (0.086)
UMPR (0.112)
UXPR (0.015)
AMPR (0.014)
AXPR (0.006)
UOV (0.002)
AOV (0)
uniform (0.01)
Pr(significance)
0.00
0.05
0.10
0.15
0.000
0.004
0.008
p−value threshold
n = 100 ; B_m = 0.3 ; B_x = −0.5 ; B_y = 0.3
CCPR (0.122)
UMPR (0.151)
UXPR (0.02)
AMPR (0.022)
AXPR (0.008)
UOV (0.003)
AOV (0)
uniform (0.01)
Pr(significance)
0.00
0.05
0.10
0.15
0.20
0.000
0.004
0.008
p−value threshold
n = 500 ; B_m = 0.3 ; B_x = −0.5 ; B_y = 0.3
CCPR (0.193)
UMPR (0.375)
UXPR (0.021)
AMPR (0.104)
AXPR (0.012)
UOV (0.008)
AOV (0.002)
uniform (0.01)
Pr(significance)
0.0
0.1
0.2
0.3
0.4
0.5
0.000
0.004
0.008
p−value threshold

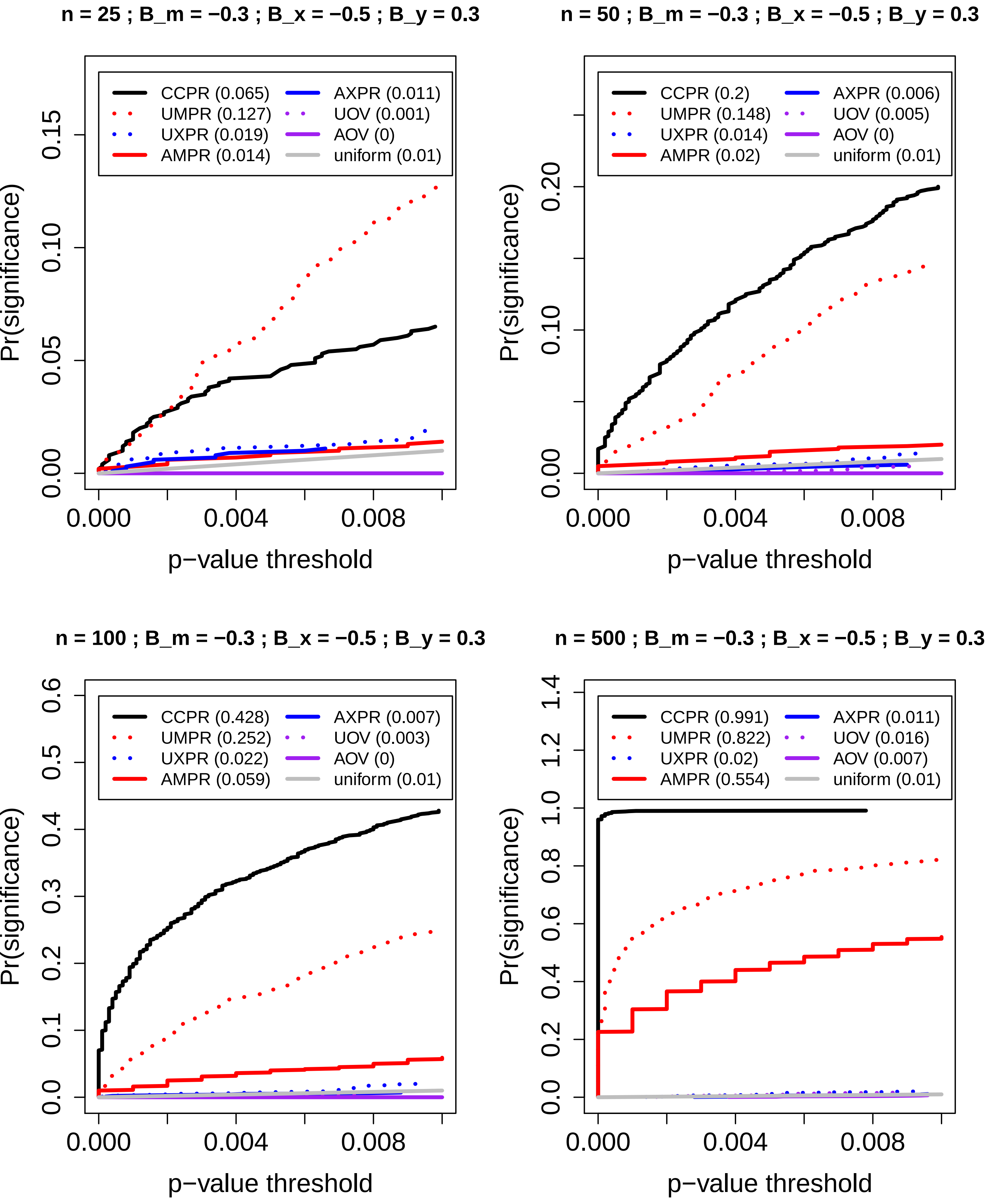

n = 25 ; B_m = −0.3 ; B_x = −0.5 ; B_y = 0.3
CCPR (0.065)
UMPR (0.127)
UXPR (0.019)
AMPR (0.014)
AXPR (0.011)
UOV (0.001)
AOV (0)
uniform (0.01)
Pr(significance)
p−value threshold
n = 50 ; B_m = −0.3 ; B_x = −0.5 ; B_y = 0.3
CCPR (0.2)
UMPR (0.148)
UXPR (0.014)
AMPR (0.02)
AXPR (0.006)
UOV (0.005)
AOV (0)
uniform (0.01)
Pr(significance)
p−value threshold
n = 100 ; B_m = −0.3 ; B_x = −0.5 ; B_y = 0.3
CCPR (0.428)
UMPR (0.252)
UXPR (0.022)
AMPR (0.059)
AXPR (0.007)
UOV (0.003)
AOV (0)
uniform (0.01)
Pr(significance)
p−value threshold
n = 500 ; B_m = −0.3 ; B_x = −0.5 ; B_y = 0.3
CCPR (0.991)
UMPR (0.822)
UXPR (0.02)
AMPR (0.554)
AXPR (0.011)
UOV (0.016)
AOV (0.007)
uniform (0.01)
Pr(significance)
p−value threshold

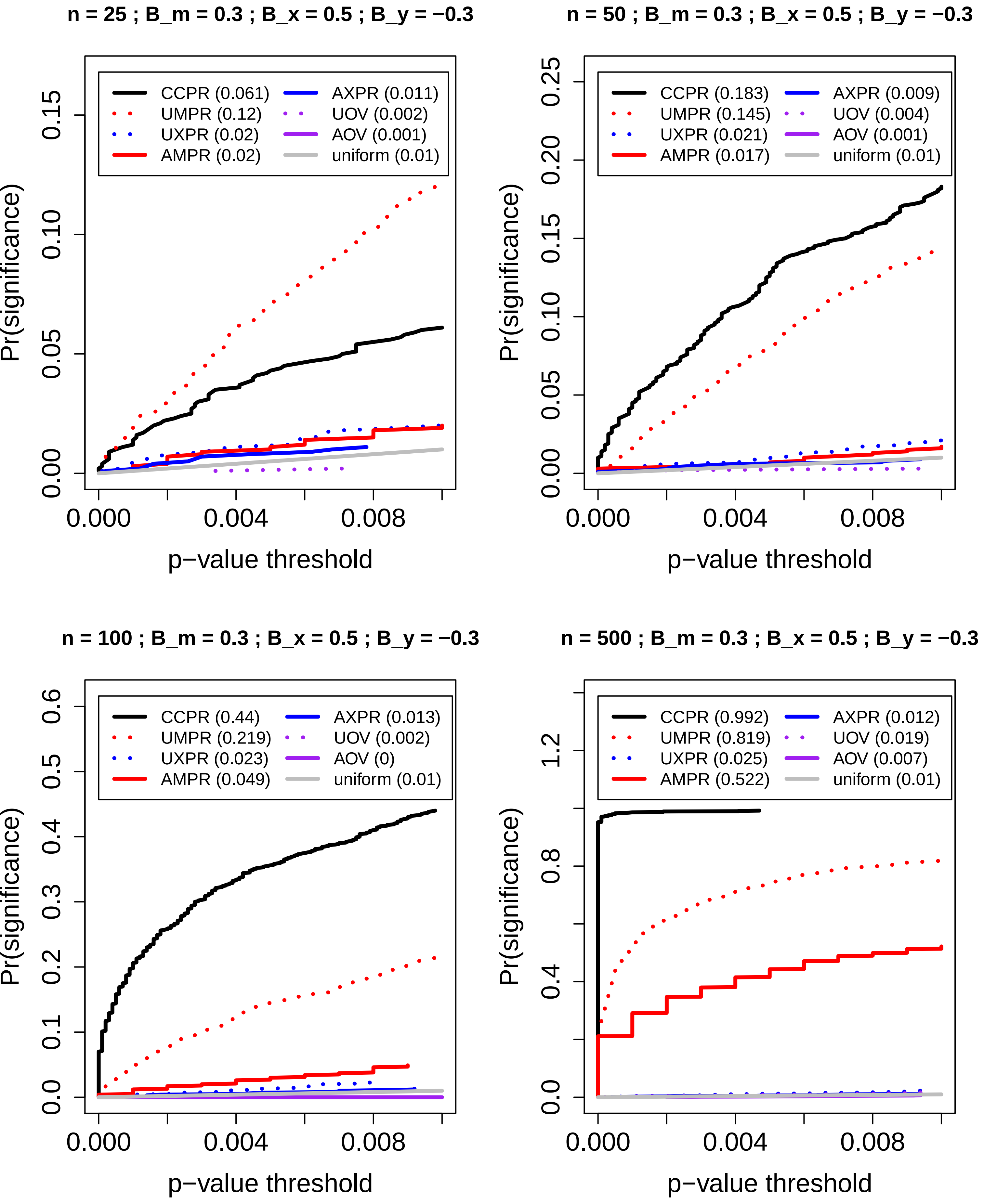

n = 25 ; B_m = 0.3 ; B_x = 0.5 ; B_y = −0.3
CCPR (0.061)
UMPR (0.12)
UXPR (0.02)
AMPR (0.02)
AXPR (0.011)
UOV (0.002)
AOV (0.001)
uniform (0.01)
Pr(significance)
p−value threshold
n = 50 ; B_m = 0.3 ; B_x = 0.5 ; B_y = −0.3
CCPR (0.183)
UMPR (0.145)
UXPR (0.021)
AMPR (0.017)
AXPR (0.009)
UOV (0.004)
AOV (0.001)
uniform (0.01)
Pr(significance)
p−value threshold
n = 100 ; B_m = 0.3 ; B_x = 0.5 ; B_y = −0.3
CCPR (0.44)
UMPR (0.219)
UXPR (0.023)
AMPR (0.049)
AXPR (0.013)
UOV (0.002)
AOV (0)
uniform (0.01)
Pr(significance)
p−value threshold
n = 500 ; B_m = 0.3 ; B_x = 0.5 ; B_y = −0.3
CCPR (0.992)
UMPR (0.819)
UXPR (0.025)
AMPR (0.522)
AXPR (0.012)
UOV (0.019)
AOV (0.007)
uniform (0.01)
Pr(significance)
p−value threshold

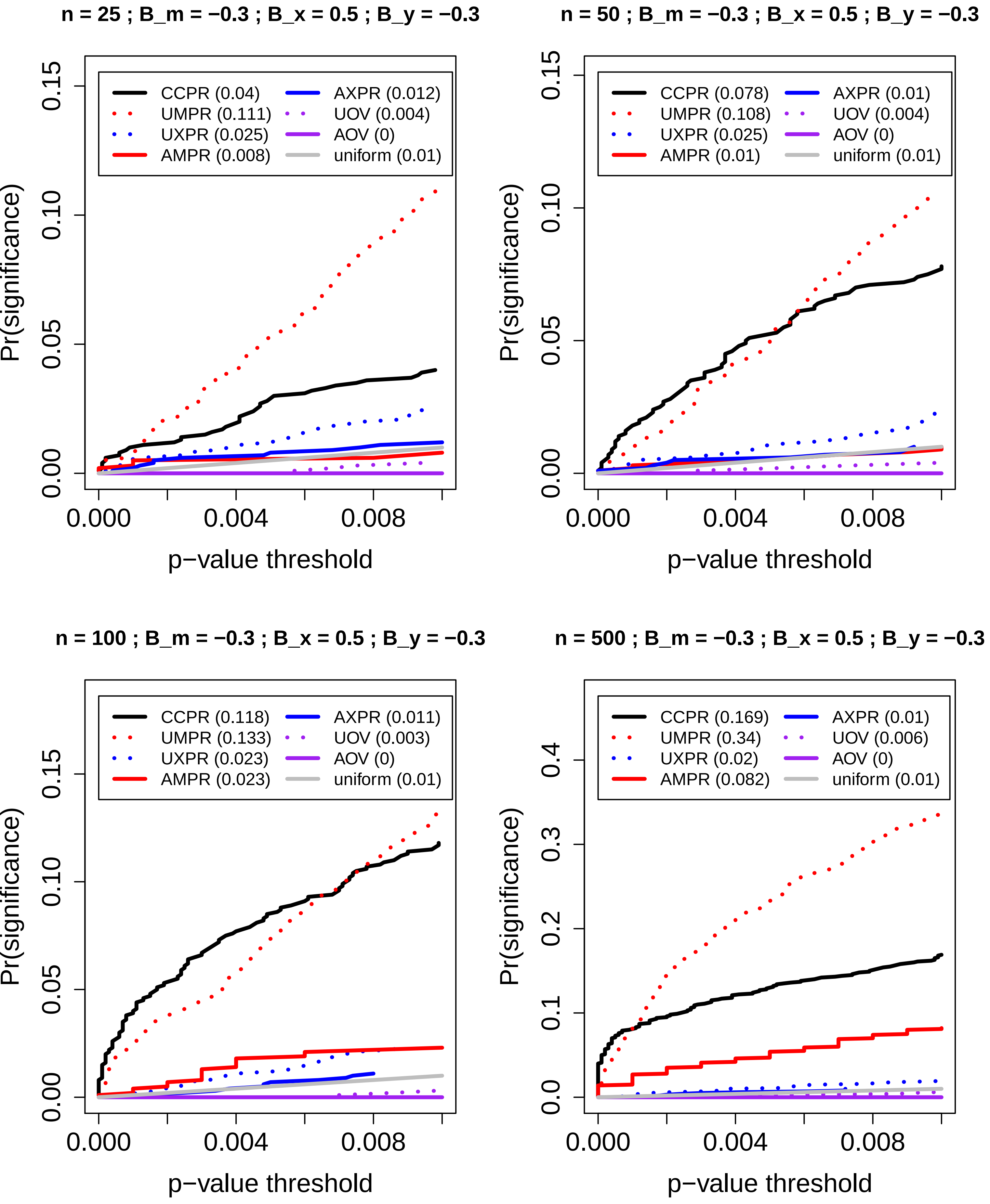
n = 25 ; B_m = −0.3 ; B_x = 0.5 ; B_y = −0.3
CCPR (0.04)
UMPR (0.111)
UXPR (0.025)
AMPR (0.008)
AXPR (0.012)
UOV (0.004)
AOV (0)
uniform (0.01)
Pr(significance)
p−value threshold
n = 50 ; B_m = −0.3 ; B_x = 0.5 ; B_y = −0.3
CCPR (0.078)
UMPR (0.108)
UXPR (0.025)
AMPR (0.01)
AXPR (0.01)
UOV (0.004)
AOV (0)
uniform (0.01)
Pr(significance)
p−value threshold
n = 100 ; B_m = −0.3 ; B_x = 0.5 ; B_y = −0.3
CCPR (0.118)
UMPR (0.133)
UXPR (0.023)
AMPR (0.023)
AXPR (0.011)
UOV (0.003)
AOV (0)
uniform (0.01)
Pr(significance)
p−value threshold
n = 500 ; B_m = −0.3 ; B_x = 0.5 ; B_y = −0.3
CCPR (0.169)
UMPR (0.34)
UXPR (0.02)
AMPR (0.082)
AXPR (0.01)
UOV (0.006)
AOV (0)
uniform (0.01)
Pr(significance)
p−value threshold

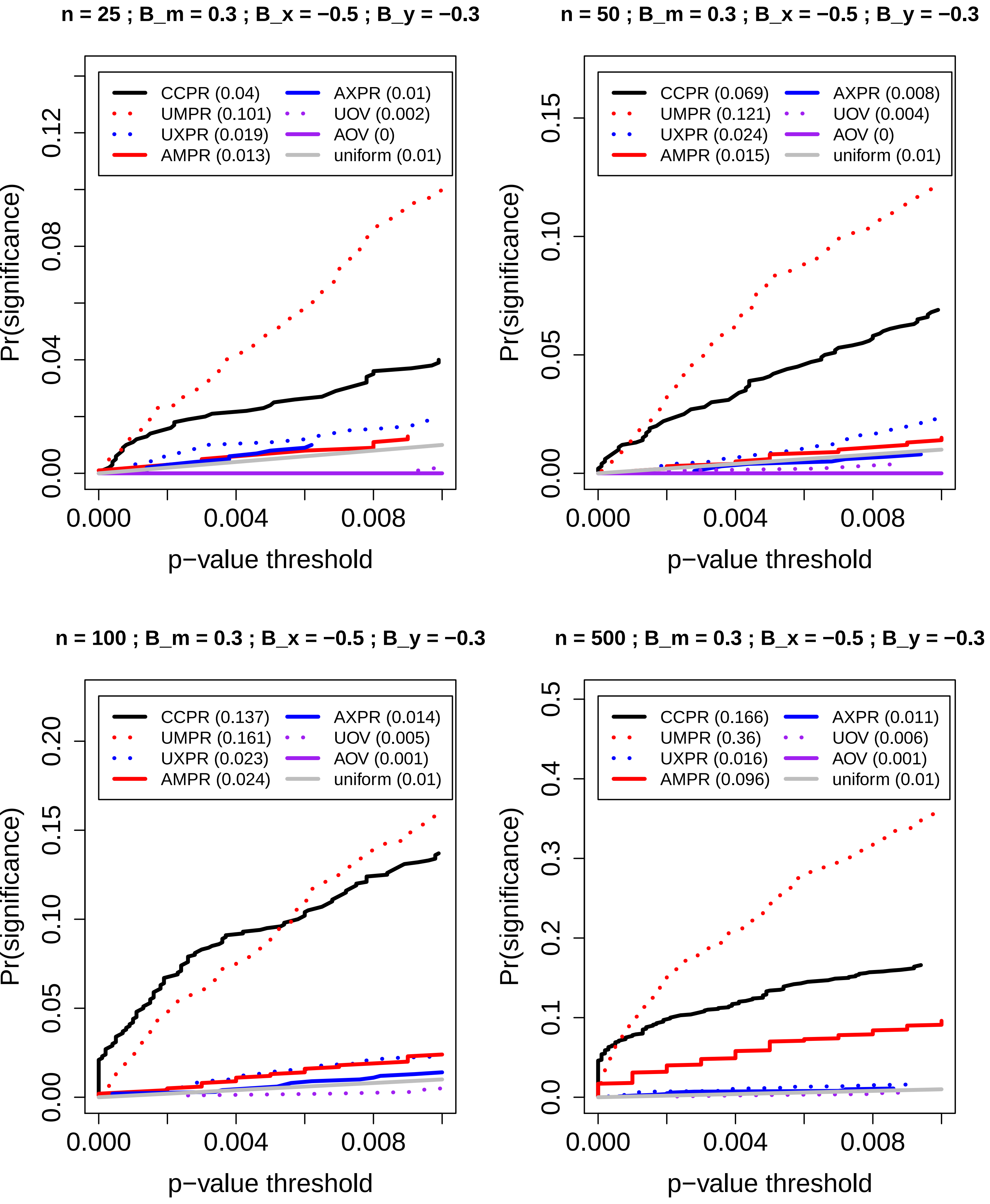
n = 25 ; B_m = 0.3 ; B_x = −0.5 ; B_y = −0.3
CCPR (0.04)
UMPR (0.101)
UXPR (0.019)
AMPR (0.013)
AXPR (0.01)
UOV (0.002)
AOV (0)
uniform (0.01)
n = 50 ; B_m = 0.3 ; B_x = −0.5 ; B_y = −0.3
CCPR (0.069)
UMPR (0.121)
UXPR (0.024)
AMPR (0.015)
AXPR (0.008)
UOV (0.004)
AOV (0)
uniform (0.01)
n = 100 ; B_m = 0.3 ; B_x = −0.5 ; B_y = −0.3
CCPR (0.137)
UMPR (0.161)
UXPR (0.023)
AMPR (0.024)
AXPR (0.014)
UOV (0.005)
AOV (0.001)
uniform (0.01)
n = 500 ; B_m = 0.3 ; B_x = −0.5 ; B_y = −0.3
CCPR (0.166)
UMPR (0.36)
UXPR (0.016)
AMPR (0.096)
AXPR (0.011)
UOV (0.006)
AOV (0.001)
uniform (0.01)
Pr(significance)
p−value threshold
0.000
0.004
0.008

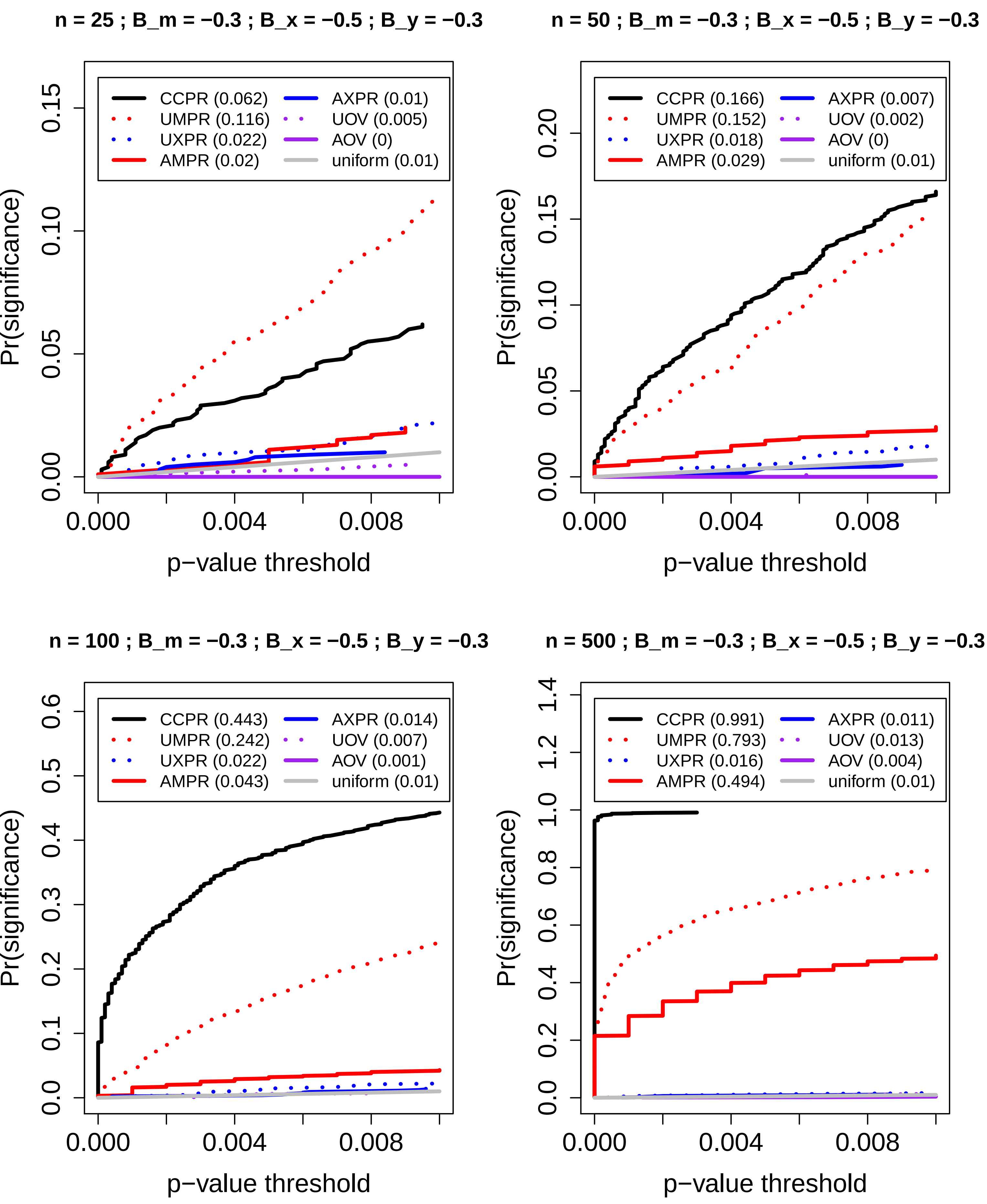

n = 25 ; B_m = −0.3 ; B_x = −0.5 ; B_y = −0.3
CCPR (0.062)
UMPR (0.116)
UXPR (0.022)
AMPR (0.02)
AXPR (0.01)
UOV (0.005)
AOV (0)
uniform (0.01)
n = 50 ; B_m = −0.3 ; B_x = −0.5 ; B_y = −0.3
CCPR (0.166)
UMPR (0.152)
UXPR (0.018)
AMPR (0.029)
AXPR (0.007)
UOV (0.002)
AOV (0)
uniform (0.01)
n = 100 ; B_m = −0.3 ; B_x = −0.5 ; B_y = −0.3
CCPR (0.443)
UMPR (0.242)
UXPR (0.022)
AMPR (0.043)
AXPR (0.014)
UOV (0.007)
AOV (0.001)
uniform (0.01)
n = 500 ; B_m = −0.3 ; B_x = −0.5 ; B_y = −0.3
CCPR (0.991)
UMPR (0.793)
UXPR (0.016)
AMPR (0.494)
AXPR (0.011)
UOV (0.013)
AOV (0.004)
uniform (0.01)
Pr(significance)
p−value threshold

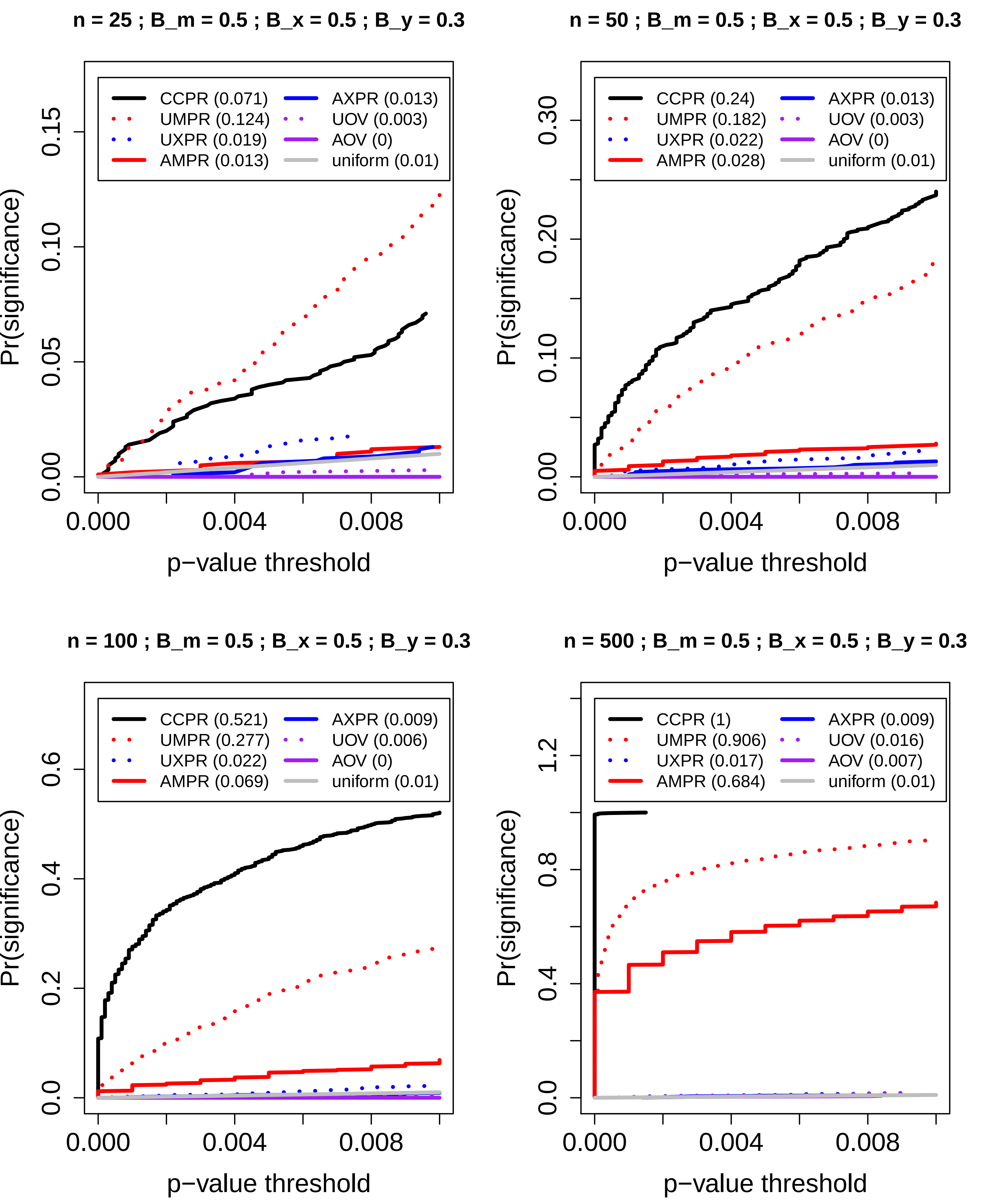

n = 25 ; B_m = 0.5 ; B_x = 0.5 ; B_y = 0.3
CCPR (0.071)
UMPR (0.124)
UXPR (0.019)
AMPR (0.013)
AXPR (0.013)
UOV (0.003)
AOV (0)
uniform (0.01)
n = 50 ; B_m = 0.5 ; B_x = 0.5 ; B_y = 0.3
CCPR (0.24)
UMPR (0.182)
UXPR (0.022)
AMPR (0.028)
AXPR (0.013)
UOV (0.003)
AOV (0)
uniform (0.01)
n = 100 ; B_m = 0.5 ; B_x = 0.5 ; B_y = 0.3
CCPR (0.521)
UMPR (0.277)
UXPR (0.022)
AMPR (0.069)
AXPR (0.009)
UOV (0.006)
AOV (0)
uniform (0.01)
n = 500 ; B_m = 0.5 ; B_x = 0.5 ; B_y = 0.3
CCPR (1)
UMPR (0.906)
UXPR (0.017)
AMPR (0.684)
AXPR (0.009)
UOV (0.016)
AOV (0.007)
uniform (0.01)
Pr(significance)
p−value threshold

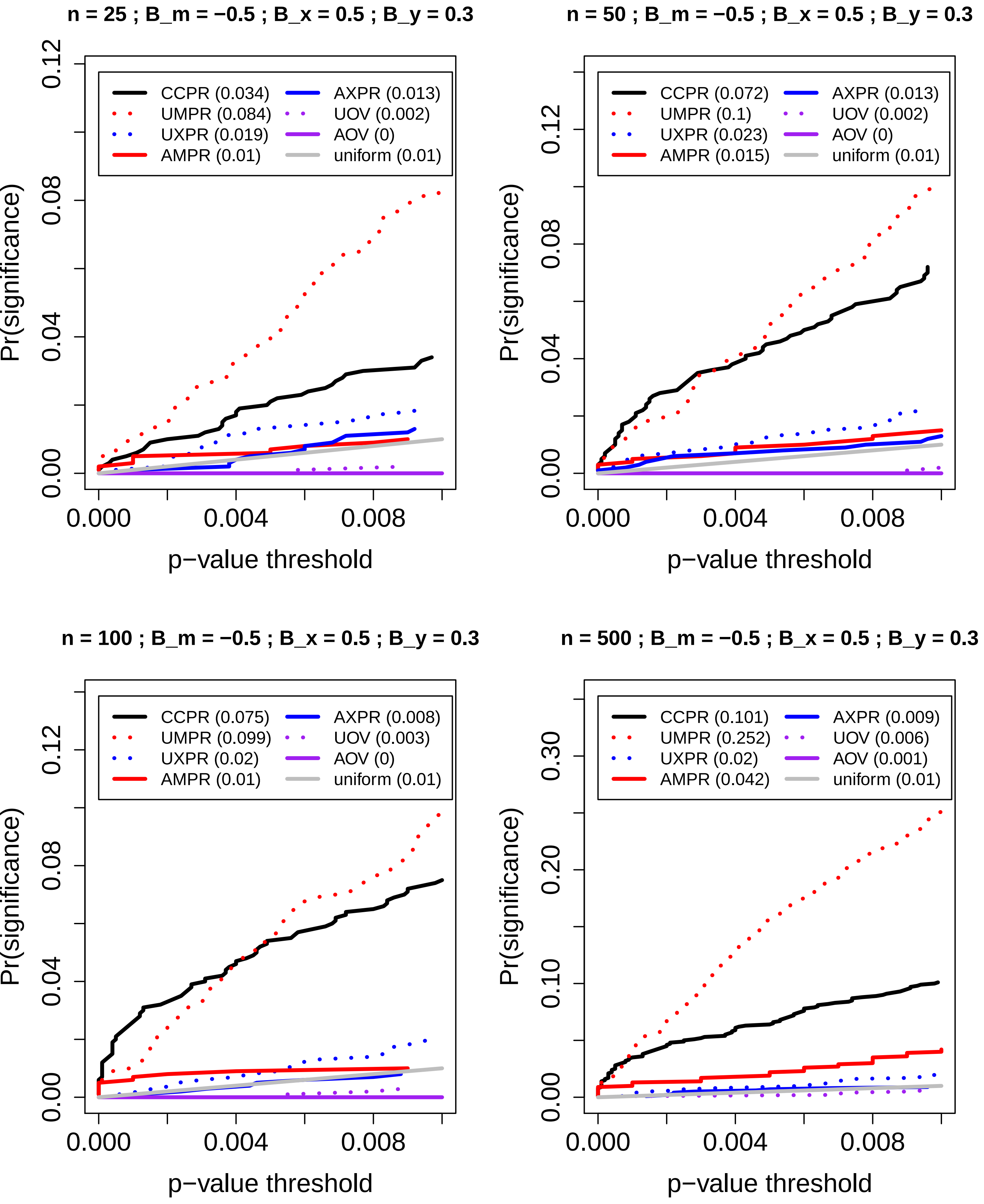
n = 25 ; B_m = −0.5 ; B_x = 0.5 ; B_y = 0.3
CCPR (0.034)
UMPR (0.084)
UXPR (0.019)
AMPR (0.01)
AXPR (0.013)
UOV (0.002)
AOV (0)
uniform (0.01)
Pr(significance)
p−value threshold
n = 50 ; B_m = −0.5 ; B_x = 0.5 ; B_y = 0.3
CCPR (0.072)
UMPR (0.1)
UXPR (0.023)
AMPR (0.015)
AXPR (0.013)
UOV (0.002)
AOV (0)
uniform (0.01)
Pr(significance)
p−value threshold
n = 100 ; B_m = −0.5 ; B_x = 0.5 ; B_y = 0.3
CCPR (0.075)
UMPR (0.099)
UXPR (0.02)
AMPR (0.01)
AXPR (0.008)
UOV (0.003)
AOV (0)
uniform (0.01)
Pr(significance)
p−value threshold
n = 500 ; B_m = −0.5 ; B_x = 0.5 ; B_y = 0.3
CCPR (0.101)
UMPR (0.252)
UXPR (0.02)
AMPR (0.042)
AXPR (0.009)
UOV (0.006)
AOV (0.001)
uniform (0.01)
Pr(significance)
p−value threshold

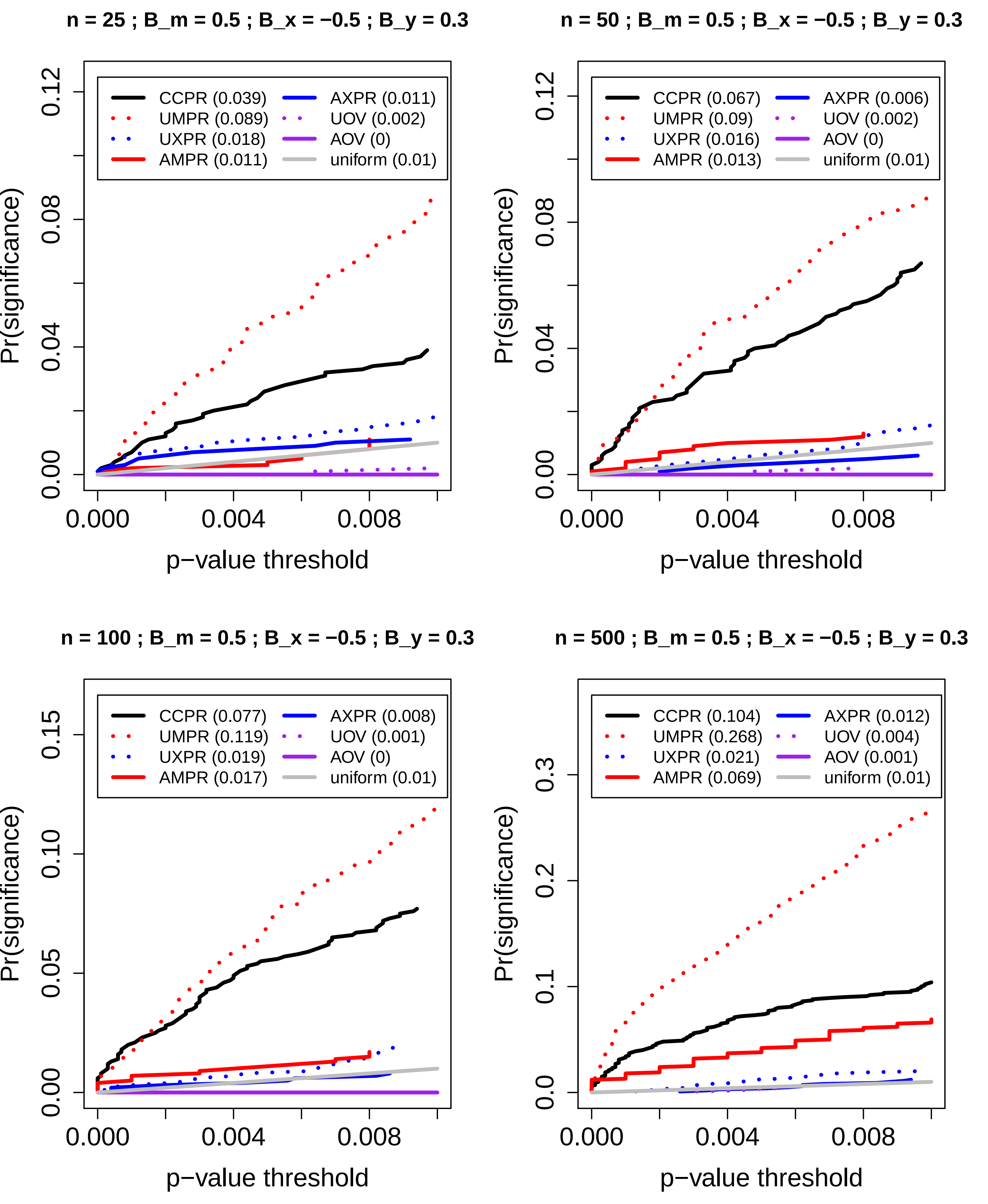
n = 25 ; B_m = 0.5 ; B_x = −0.5 ; B_y = 0.3
CCPR (0.039)
UMPR (0.089)
UXPR (0.018)
AMPR (0.011)
AXPR (0.011)
UOV (0.002)
AOV (0)
uniform (0.01)
Pr(significance)
p−value threshold
n = 50 ; B_m = 0.5 ; B_x = −0.5 ; B_y = 0.3
CCPR (0.067)
UMPR (0.09)
UXPR (0.016)
AMPR (0.013)
AXPR (0.006)
UOV (0.002)
AOV (0)
uniform (0.01)
Pr(significance)
p−value threshold
n = 100 ; B_m = 0.5 ; B_x = −0.5 ; B_y = 0.3
CCPR (0.077)
UMPR (0.119)
UXPR (0.019)
AMPR (0.017)
AXPR (0.008)
UOV (0.001)
AOV (0)
uniform (0.01)
Pr(significance)
p−value threshold
n = 500 ; B_m = 0.5 ; B_x = −0.5 ; B_y = 0.3
CCPR (0.104)
UMPR (0.268)
UXPR (0.021)
AMPR (0.069)
AXPR (0.012)
UOV (0.004)
AOV (0.001)
uniform (0.01)
Pr(significance)
p−value threshold

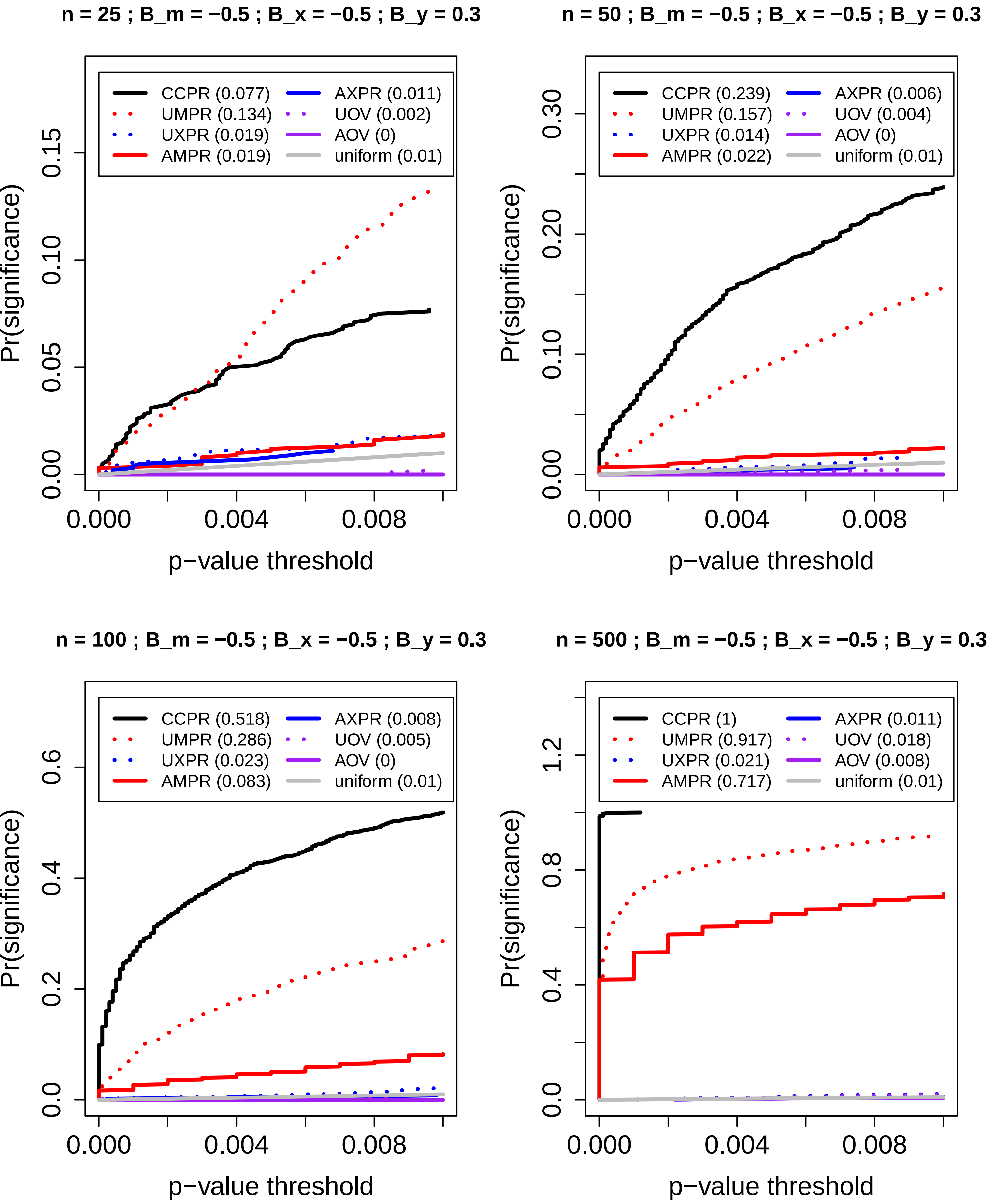
n = 25 ; B_m = −0.5 ; B_x = −0.5 ; B_y = 0.3
CCPR (0.077)
UMPR (0.134)
UXPR (0.019)
AMPR (0.019)
AXPR (0.011)
UOV (0.002)
AOV (0)
uniform (0.01)
n = 50 ; B_m = −0.5 ; B_x = −0.5 ; B_y = 0.3
CCPR (0.239)
UMPR (0.157)
UXPR (0.014)
AMPR (0.022)
AXPR (0.006)
UOV (0.004)
AOV (0)
uniform (0.01)
n = 100 ; B_m = −0.5 ; B_x = −0.5 ; B_y = 0.3
CCPR (0.518)
UMPR (0.286)
UXPR (0.023)
AMPR (0.083)
AXPR (0.008)
UOV (0.005)
AOV (0)
uniform (0.01)
n = 500 ; B_m = −0.5 ; B_x = −0.5 ; B_y = 0.3
CCPR (1)
UMPR (0.917)
UXPR (0.021)
AMPR (0.717)
AXPR (0.011)
UOV (0.018)
AOV (0.008)
uniform (0.01)
Pr(significance)
p−value threshold
0.000
0.004
0.008

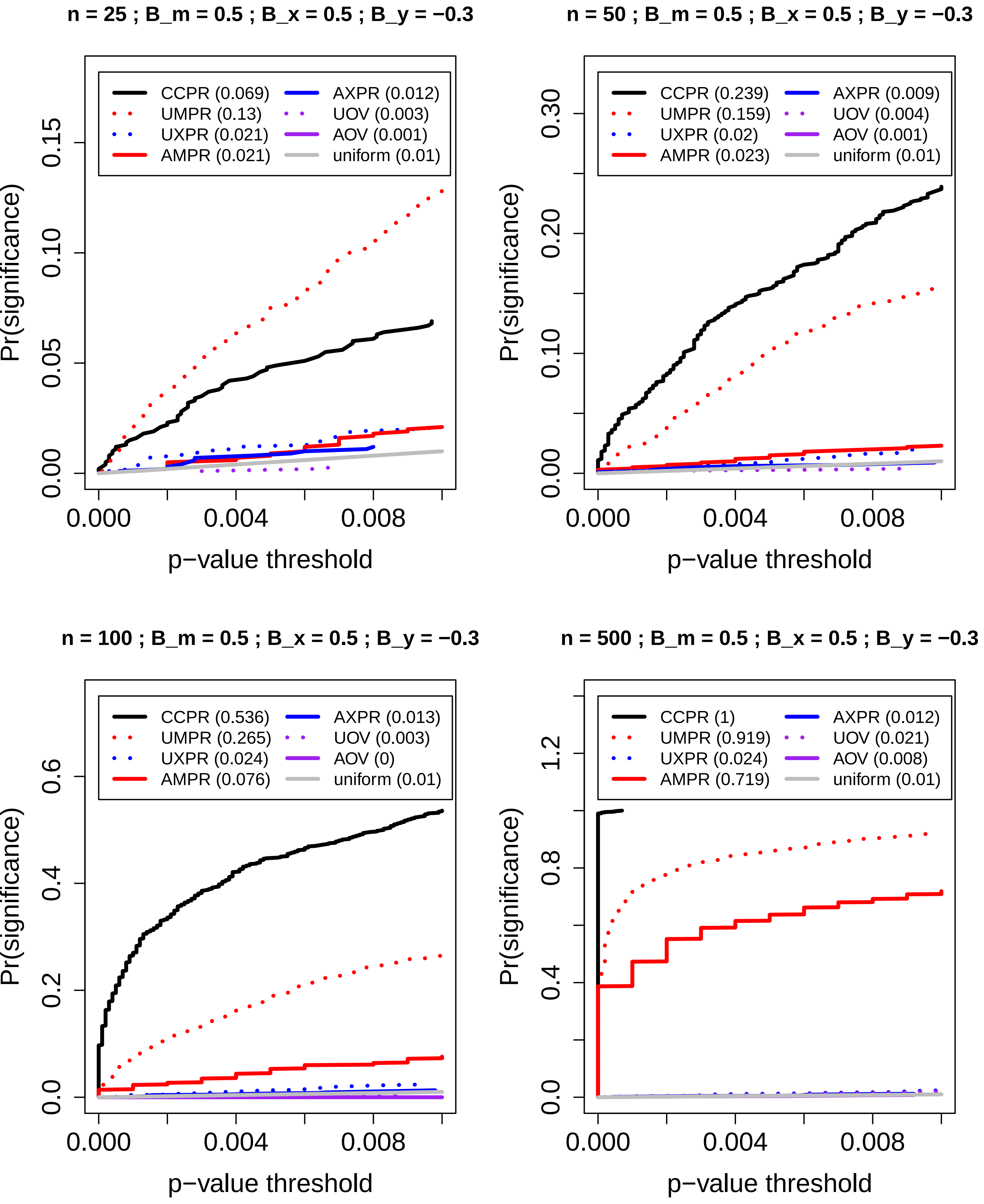

n = 25 ; B_m = 0.5 ; B_x = 0.5 ; B_y = −0.3
CCPR (0.069)
UMPR (0.13)
UXPR (0.021)
AMPR (0.021)
AXPR (0.012)
UOV (0.003)
AOV (0.001)
uniform (0.01)
Pr(significance)
0.00
0.05
0.10
0.15
0.000
0.004
0.008
p−value threshold
n = 50 ; B_m = 0.5 ; B_x = 0.5 ; B_y = −0.3
CCPR (0.239)
UMPR (0.159)
UXPR (0.02)
AMPR (0.023)
AXPR (0.009)
UOV (0.004)
AOV (0.001)
uniform (0.01)
Pr(significance)
0.00
0.10
0.20
0.30
0.000
0.004
0.008
p−value threshold
n = 100 ; B_m = 0.5 ; B_x = 0.5 ; B_y = −0.3
CCPR (0.536)
UMPR (0.265)
UXPR (0.024)
AMPR (0.076)
AXPR (0.013)
UOV (0.003)
AOV (0)
uniform (0.01)
Pr(significance)
0.0
0.2
0.4
0.6
0.000
0.004
0.008
p−value threshold
n = 500 ; B_m = 0.5 ; B_x = 0.5 ; B_y = −0.3
CCPR (1)
UMPR (0.919)
UXPR (0.024)
AMPR (0.719)
AXPR (0.012)
UOV (0.021)
AOV (0.008)
uniform (0.01)
Pr(significance)
0.0
0.4
0.8
1.2
0.000
0.004
0.008
p−value threshold

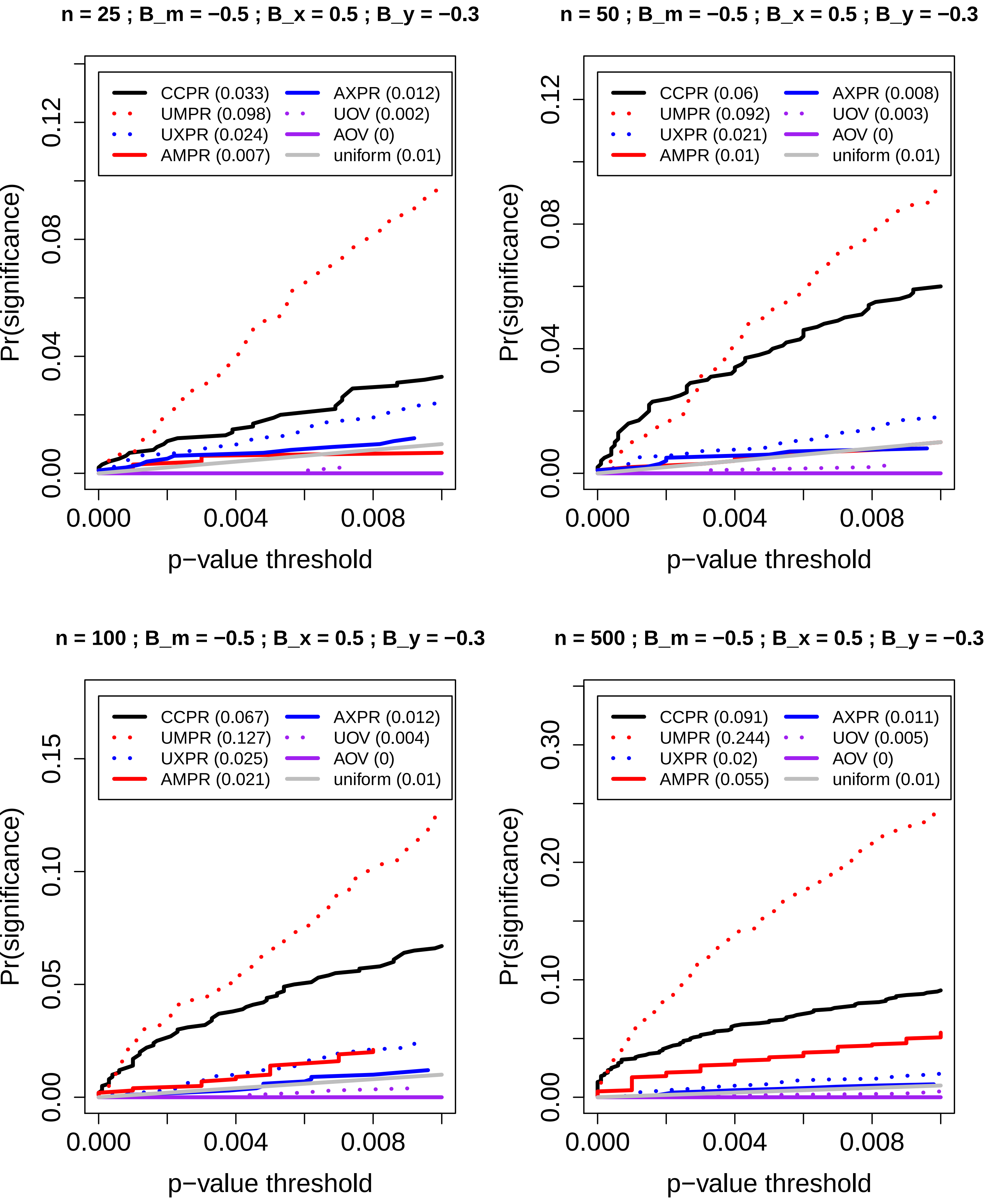

n = 25 ; B_m = −0.5 ; B_x = 0.5 ; B_y = −0.3
CCPR (0.033)
UMPR (0.098)
UXPR (0.024)
AMPR (0.007)
AXPR (0.012)
UOV (0.002)
AOV (0)
uniform (0.01)
Pr(significance)
p−value threshold
n = 50 ; B_m = −0.5 ; B_x = 0.5 ; B_y = −0.3
CCPR (0.06)
UMPR (0.092)
UXPR (0.021)
AMPR (0.01)
AXPR (0.008)
UOV (0.003)
AOV (0)
uniform (0.01)
Pr(significance)
p−value threshold
n = 100 ; B_m = −0.5 ; B_x = 0.5 ; B_y = −0.3
CCPR (0.067)
UMPR (0.127)
UXPR (0.025)
AMPR (0.021)
AXPR (0.012)
UOV (0.004)
AOV (0)
uniform (0.01)
Pr(significance)
p−value threshold
n = 500 ; B_m = −0.5 ; B_x = 0.5 ; B_y = −0.3
CCPR (0.091)
UMPR (0.244)
UXPR (0.02)
AMPR (0.055)
AXPR (0.011)
UOV (0.005)
AOV (0)
uniform (0.01)
Pr(significance)
p−value threshold

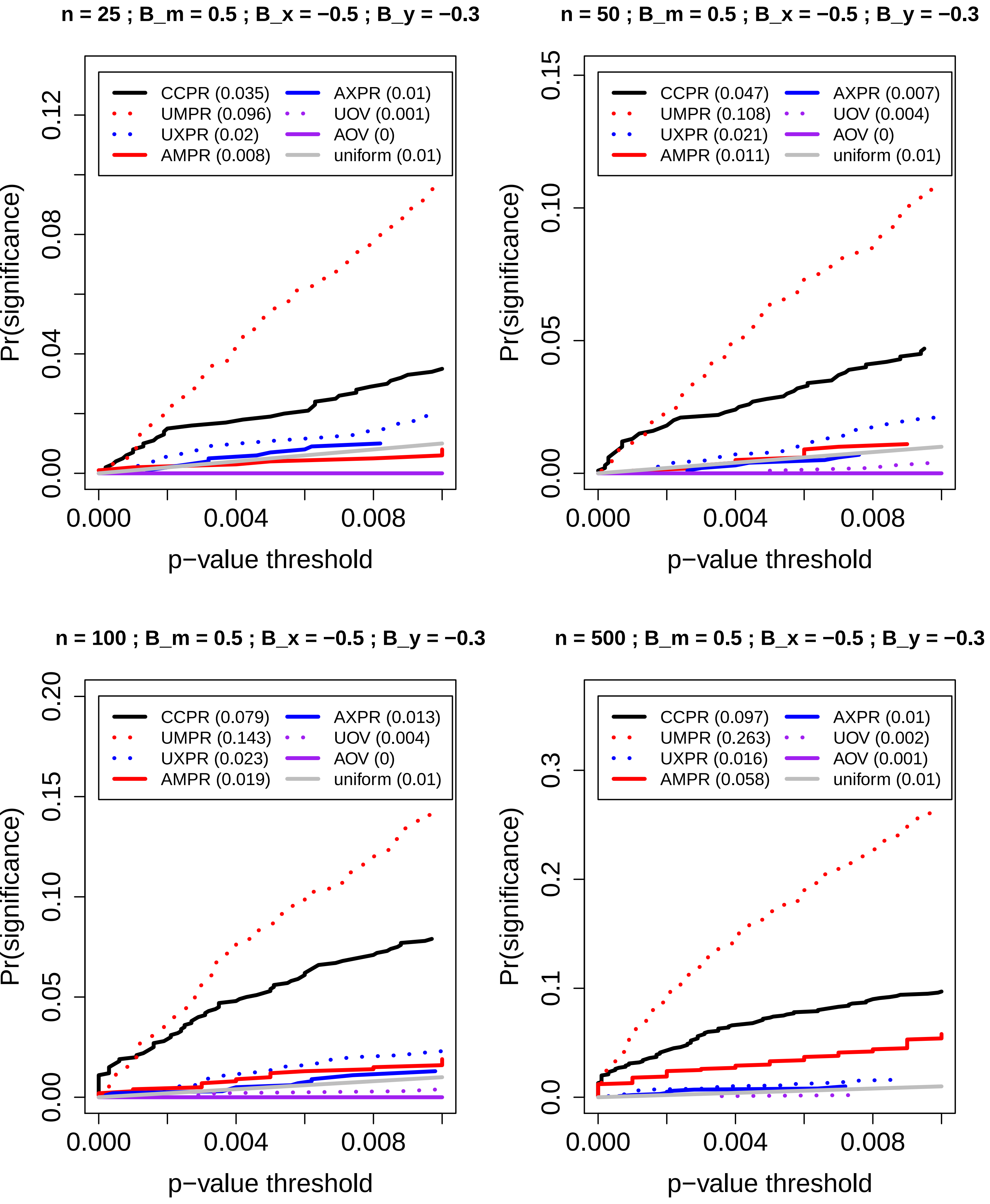
n = 25 ; B_m = 0.5 ; B_x = −0.5 ; B_y = −0.3
CCPR (0.035)
UMPR (0.096)
UXPR (0.02)
AMPR (0.008)
AXPR (0.01)
UOV (0.001)
AOV (0)
uniform (0.01)
Pr(significance)
0.00
0.04
0.08
0.12
0.000
0.004
0.008
p−value threshold
n = 50 ; B_m = 0.5 ; B_x = −0.5 ; B_y = −0.3
CCPR (0.047)
UMPR (0.108)
UXPR (0.021)
AMPR (0.011)
AXPR (0.007)
UOV (0.004)
AOV (0)
uniform (0.01)
Pr(significance)
0.00
0.05
0.10
0.15
0.000
0.004
0.008
p−value threshold
n = 100 ; B_m = 0.5 ; B_x = −0.5 ; B_y = −0.3
CCPR (0.079)
UMPR (0.143)
UXPR (0.023)
AMPR (0.019)
AXPR (0.013)
UOV (0.004)
AOV (0)
uniform (0.01)
Pr(significance)
0.00
0.05
0.10
0.15
0.20
0.000
0.004
0.008
p−value threshold
n = 500 ; B_m = 0.5 ; B_x = −0.5 ; B_y = −0.3
CCPR (0.097)
UMPR (0.263)
UXPR (0.016)
AMPR (0.058)
AXPR (0.01)
UOV (0.002)
AOV (0.001)
uniform (0.01)
Pr(significance)
0.0
0.1
0.2
0.3
0.000
0.004
0.008
p−value threshold

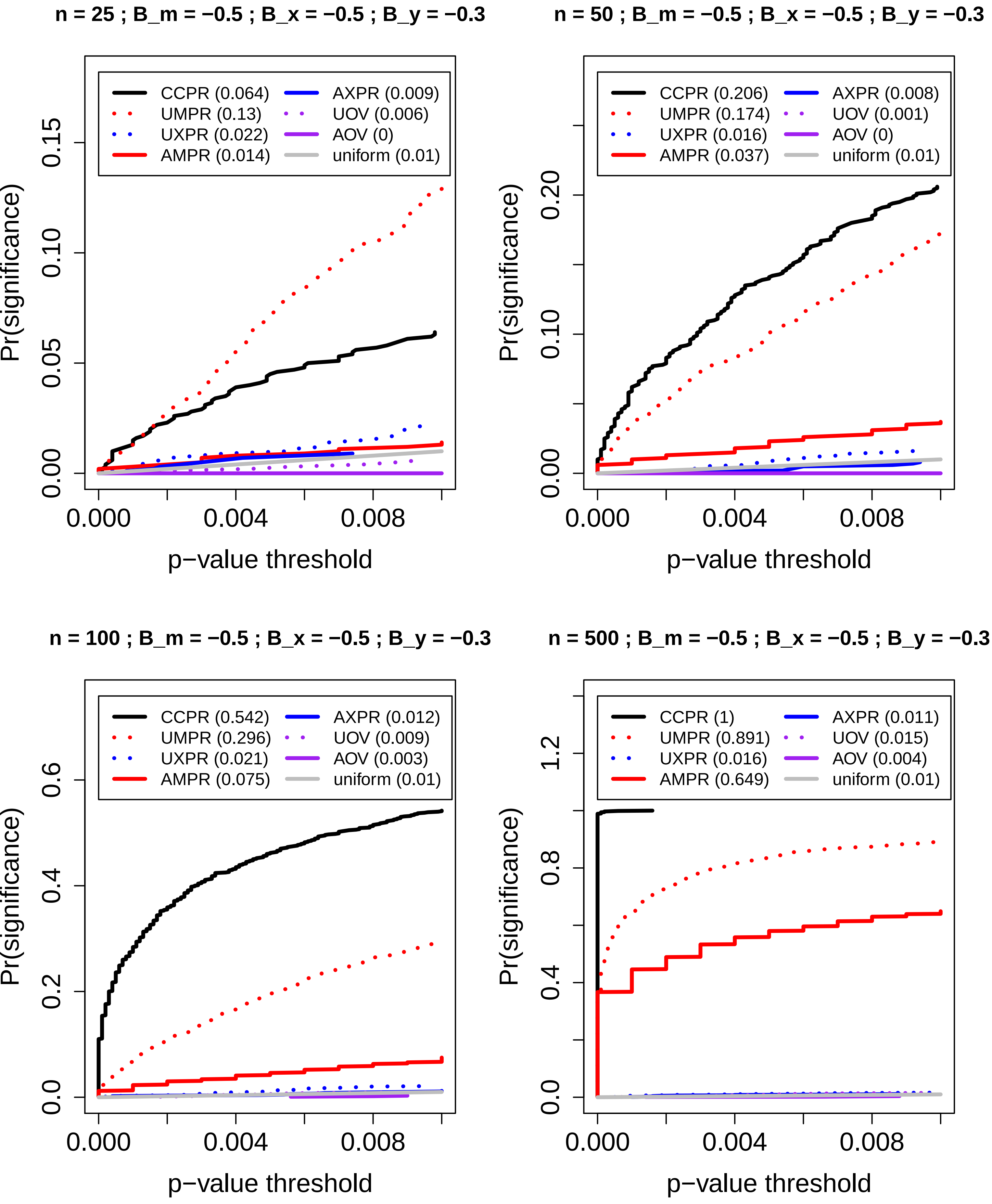

n = 25 ; B_m = −0.5 ; B_x = −0.5 ; B_y = −0.3
CCPR (0.064)
UMPR (0.13)
UXPR (0.022)
AMPR (0.014)
AXPR (0.009)
UOV (0.006)
AOV (0)
uniform (0.01)
n = 50 ; B_m = −0.5 ; B_x = −0.5 ; B_y = −0.3
CCPR (0.206)
UMPR (0.174)
UXPR (0.016)
AMPR (0.037)
AXPR (0.008)
UOV (0.001)
AOV (0)
uniform (0.01)
n = 100 ; B_m = −0.5 ; B_x = −0.5 ; B_y = −0.3
CCPR (0.542)
UMPR (0.296)
UXPR (0.021)
AMPR (0.075)
AXPR (0.012)
UOV (0.009)
AOV (0.003)
uniform (0.01)
n = 500 ; B_m = −0.5 ; B_x = −0.5 ; B_y = −0.3
CCPR (1)
UMPR (0.891)
UXPR (0.016)
AMPR (0.649)
AXPR (0.011)
UOV (0.015)
AOV (0.004)
uniform (0.01)
Pr(significance)
p−value threshold

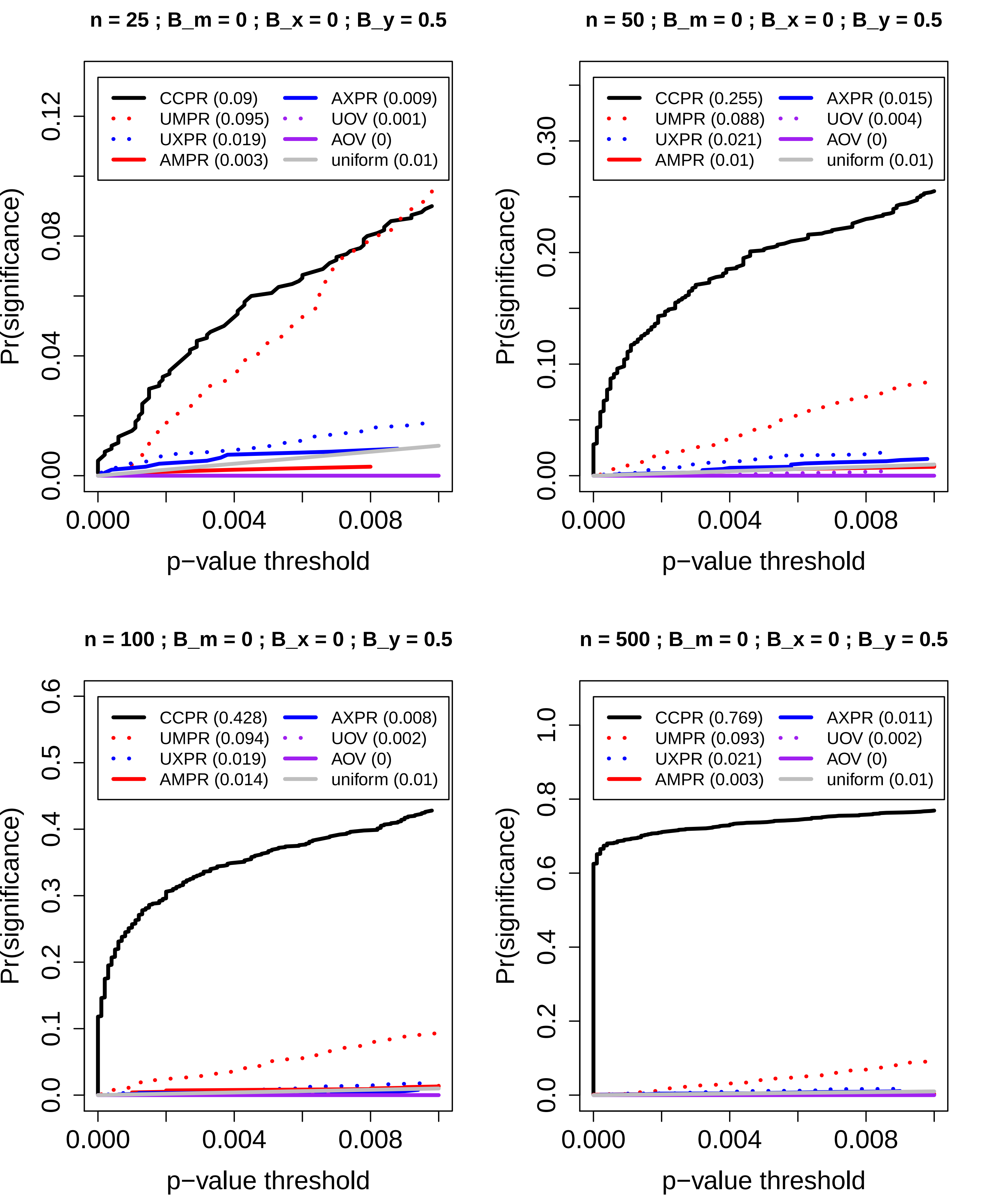
n = 25 ; B_m = 0 ; B_x = 0 ; B_y = 0.5
CCPR (0.09)
UMPR (0.095)
UXPR (0.019)
AMPR (0.003)
AXPR (0.009)
UOV (0.001)
AOV (0)
uniform (0.01)
Pr(significance)
0.00
0.04
0.08
0.12
0.000
0.004
0.008
p−value threshold
n = 50 ; B_m = 0 ; B_x = 0 ; B_y = 0.5
CCPR (0.255)
UMPR (0.088)
UXPR (0.021)
AMPR (0.01)
AXPR (0.015)
UOV (0.004)
AOV (0)
uniform (0.01)
Pr(significance)
0.00
0.10
0.20
0.30
0.000
0.004
0.008
p−value threshold
n = 100 ; B_m = 0 ; B_x = 0 ; B_y = 0.5
CCPR (0.428)
UMPR (0.094)
UXPR (0.019)
AMPR (0.014)
AXPR (0.008)
UOV (0.002)
AOV (0)
uniform (0.01)
Pr(significance)
0.0
0.1
0.2
0.3
0.4
0.5
0.6
0.000
0.004
0.008
p−value threshold
n = 500 ; B_m = 0 ; B_x = 0 ; B_y = 0.5
CCPR (0.769)
UMPR (0.093)
UXPR (0.021)
AMPR (0.003)
AXPR (0.011)
UOV (0.002)
AOV (0)
uniform (0.01)
Pr(significance)
0.0
0.2
0.4
0.6
0.8
1.0
0.000
0.004
0.008
p−value threshold

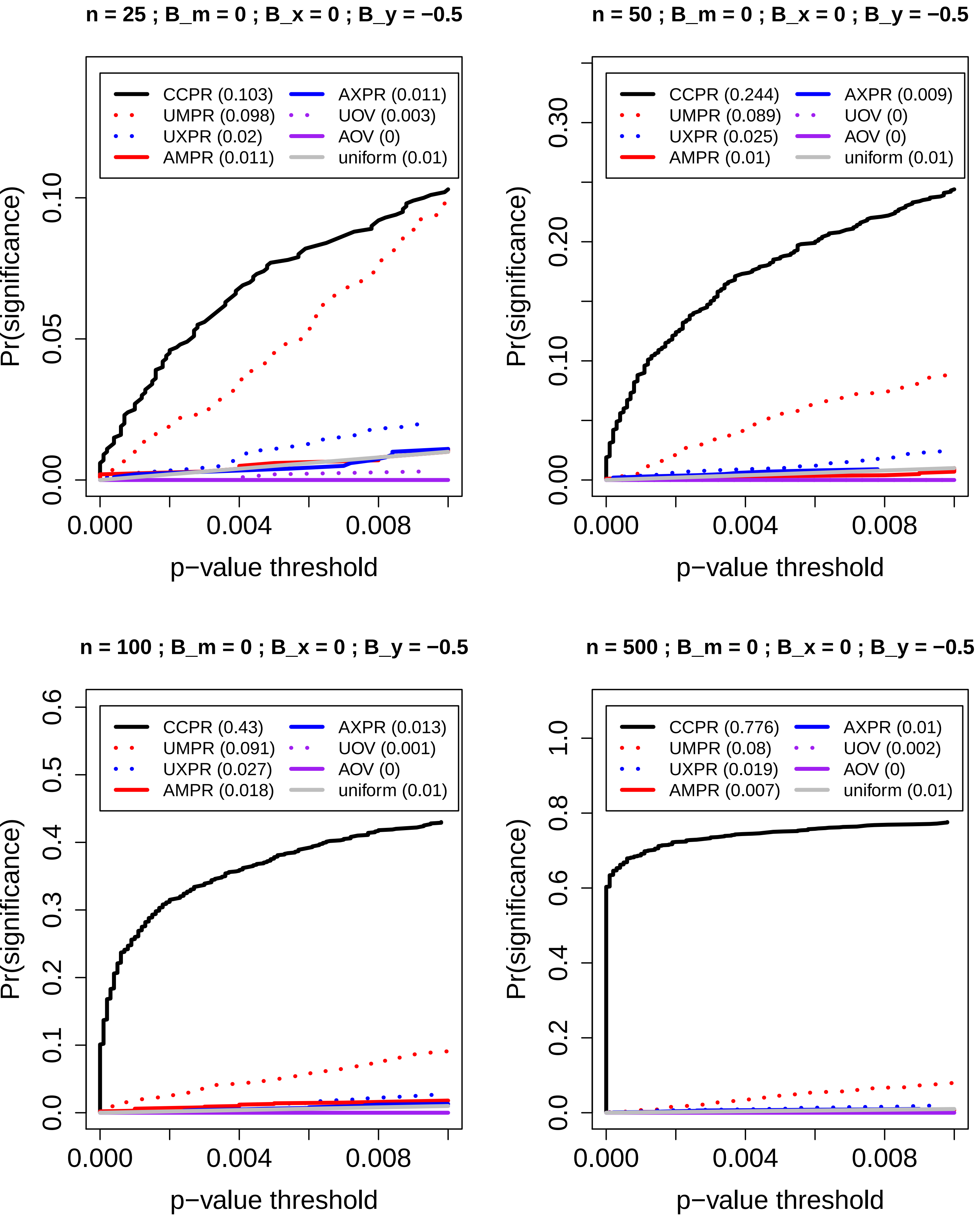
n = 25 ; B_m = 0 ; B_x = 0 ; B_y = −0.5
CCPR (0.103)
UMPR (0.098)
UXPR (0.02)
AMPR (0.011)
AXPR (0.011)
UOV (0.003)
AOV (0)
uniform (0.01)
Pr(significance)
p−value threshold
n = 50 ; B_m = 0 ; B_x = 0 ; B_y = −0.5
CCPR (0.244)
UMPR (0.089)
UXPR (0.025)
AMPR (0.01)
AXPR (0.009)
UOV (0)
AOV (0)
uniform (0.01)
n = 100 ; B_m = 0 ; B_x = 0 ; B_y = −0.5
CCPR (0.43)
UMPR (0.091)
UXPR (0.027)
AMPR (0.018)
AXPR (0.013)
UOV (0.001)
AOV (0)
uniform (0.01)
n = 500 ; B_m = 0 ; B_x = 0 ; B_y = −0.5
CCPR (0.776)
UMPR (0.08)
UXPR (0.019)
AMPR (0.007)
AXPR (0.01)
UOV (0.002)
AOV (0)
uniform (0.01)

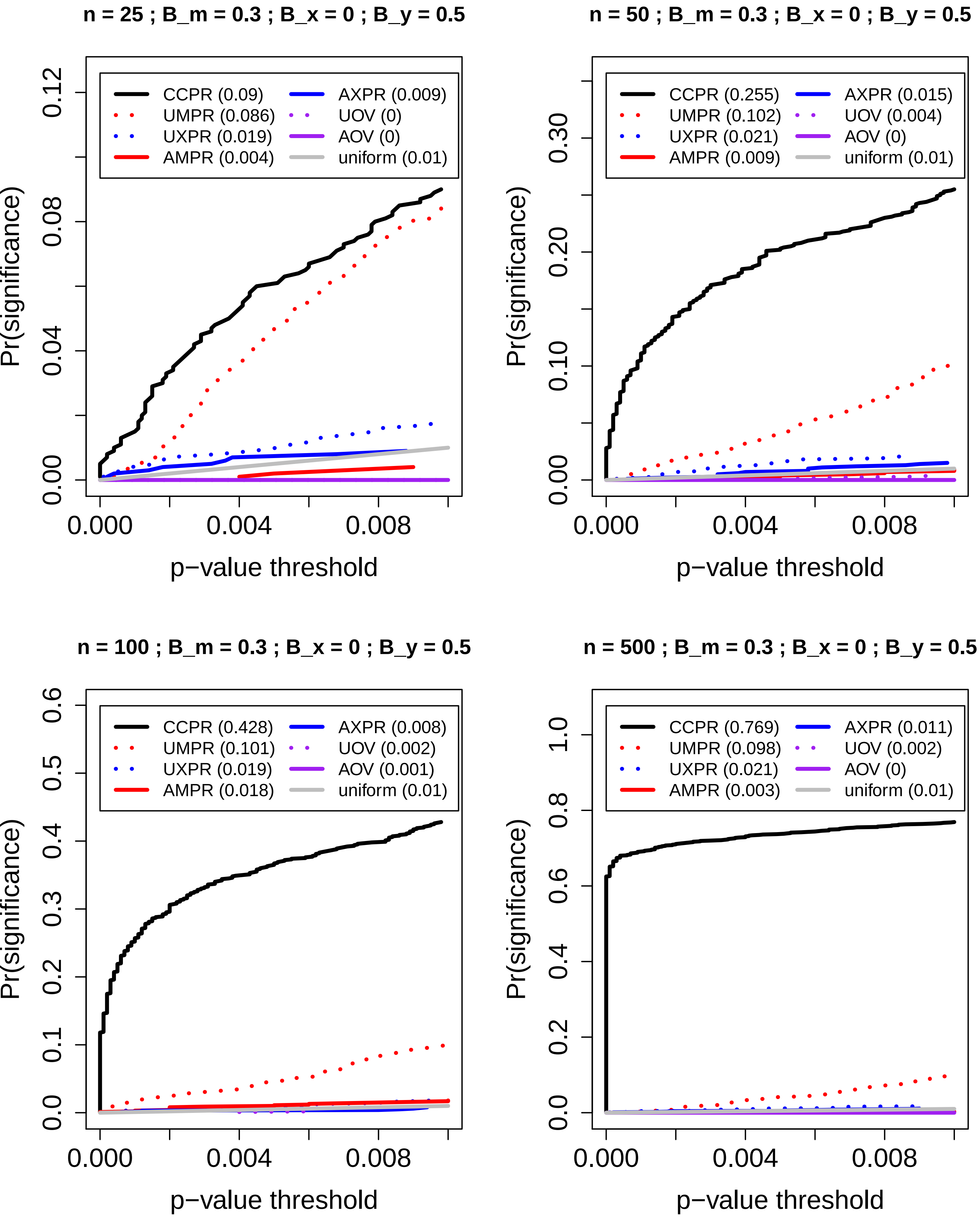

n = 25 ; B_m = 0.3 ; B_x = 0 ; B_y = 0.5
CCPR (0.09)
UMPR (0.086)
UXPR (0.019)
AMPR (0.004)
AXPR (0.009)
UOV (0)
AOV (0)
uniform (0.01)
Pr(significance)
p−value threshold
n = 50 ; B_m = 0.3 ; B_x = 0 ; B_y = 0.5
CCPR (0.255)
UMPR (0.102)
UXPR (0.021)
AMPR (0.009)
AXPR (0.015)
UOV (0.004)
AOV (0)
uniform (0.01)
Pr(significance)
p−value threshold
n = 100 ; B_m = 0.3 ; B_x = 0 ; B_y = 0.5
CCPR (0.428)
UMPR (0.101)
UXPR (0.019)
AMPR (0.018)
AXPR (0.008)
UOV (0.002)
AOV (0.001)
uniform (0.01)
Pr(significance)
p−value threshold
n = 500 ; B_m = 0.3 ; B_x = 0 ; B_y = 0.5
CCPR (0.769)
UMPR (0.098)
UXPR (0.021)
AMPR (0.003)
AXPR (0.011)
UOV (0.002)
AOV (0)
uniform (0.01)
Pr(significance)
p−value threshold

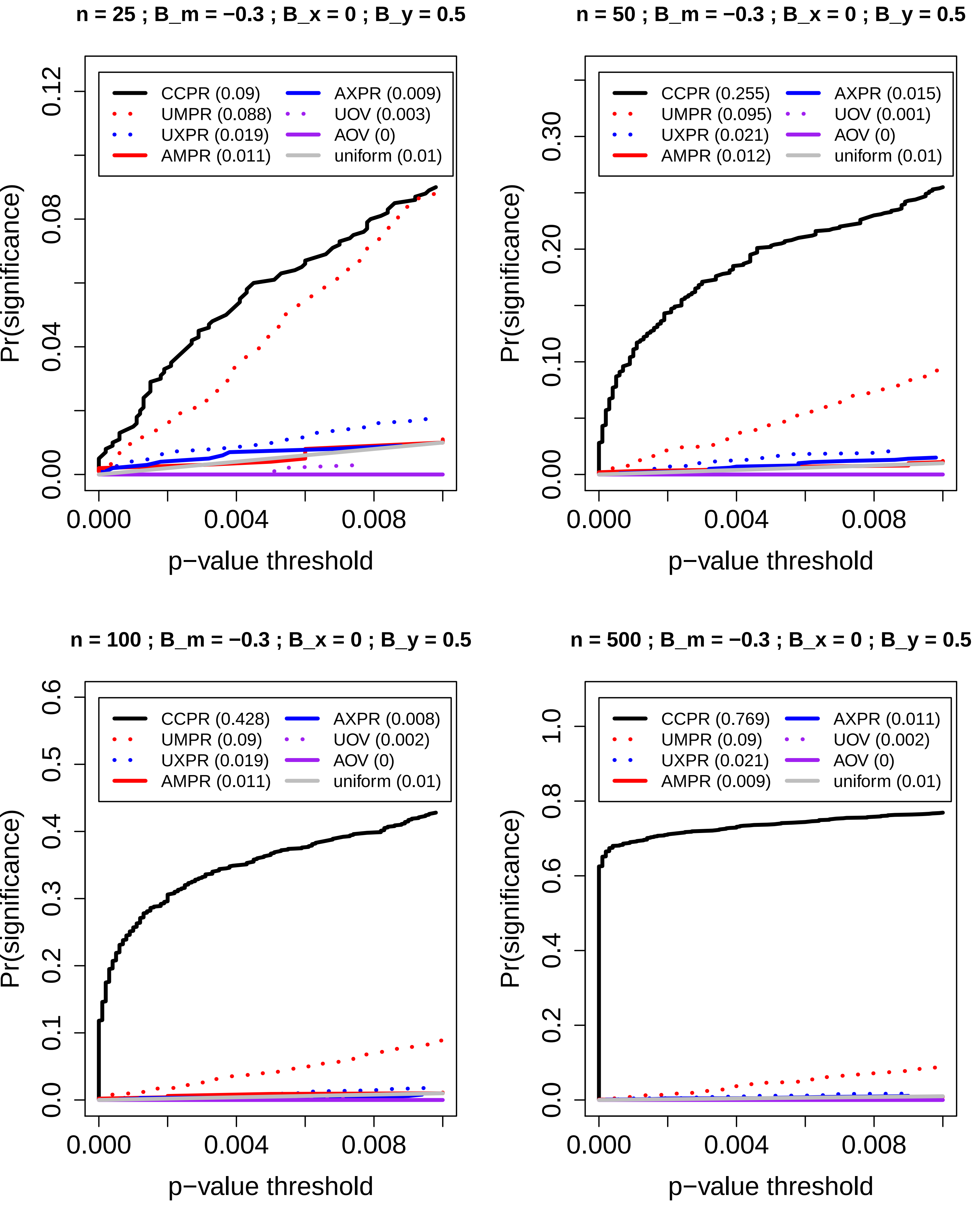

n = 25 ; B_m = −0.3 ; B_x = 0 ; B_y = 0.5
CCPR (0.09)
UMPR (0.088)
UXPR (0.019)
AMPR (0.011)
AXPR (0.009)
UOV (0.003)
AOV (0)
uniform (0.01)
Pr(significance)
p−value threshold
n = 50 ; B_m = −0.3 ; B_x = 0 ; B_y = 0.5
CCPR (0.255)
UMPR (0.095)
UXPR (0.021)
AMPR (0.012)
AXPR (0.015)
UOV (0.001)
AOV (0)
uniform (0.01)
Pr(significance)
p−value threshold
n = 100 ; B_m = −0.3 ; B_x = 0 ; B_y = 0.5
CCPR (0.428)
UMPR (0.09)
UXPR (0.019)
AMPR (0.011)
AXPR (0.008)
UOV (0.002)
AOV (0)
uniform (0.01)
Pr(significance)
p−value threshold
n = 500 ; B_m = −0.3 ; B_x = 0 ; B_y = 0.5
CCPR (0.769)
UMPR (0.09)
UXPR (0.021)
AMPR (0.009)
AXPR (0.011)
UOV (0.002)
AOV (0)
uniform (0.01)
Pr(significance)
p−value threshold

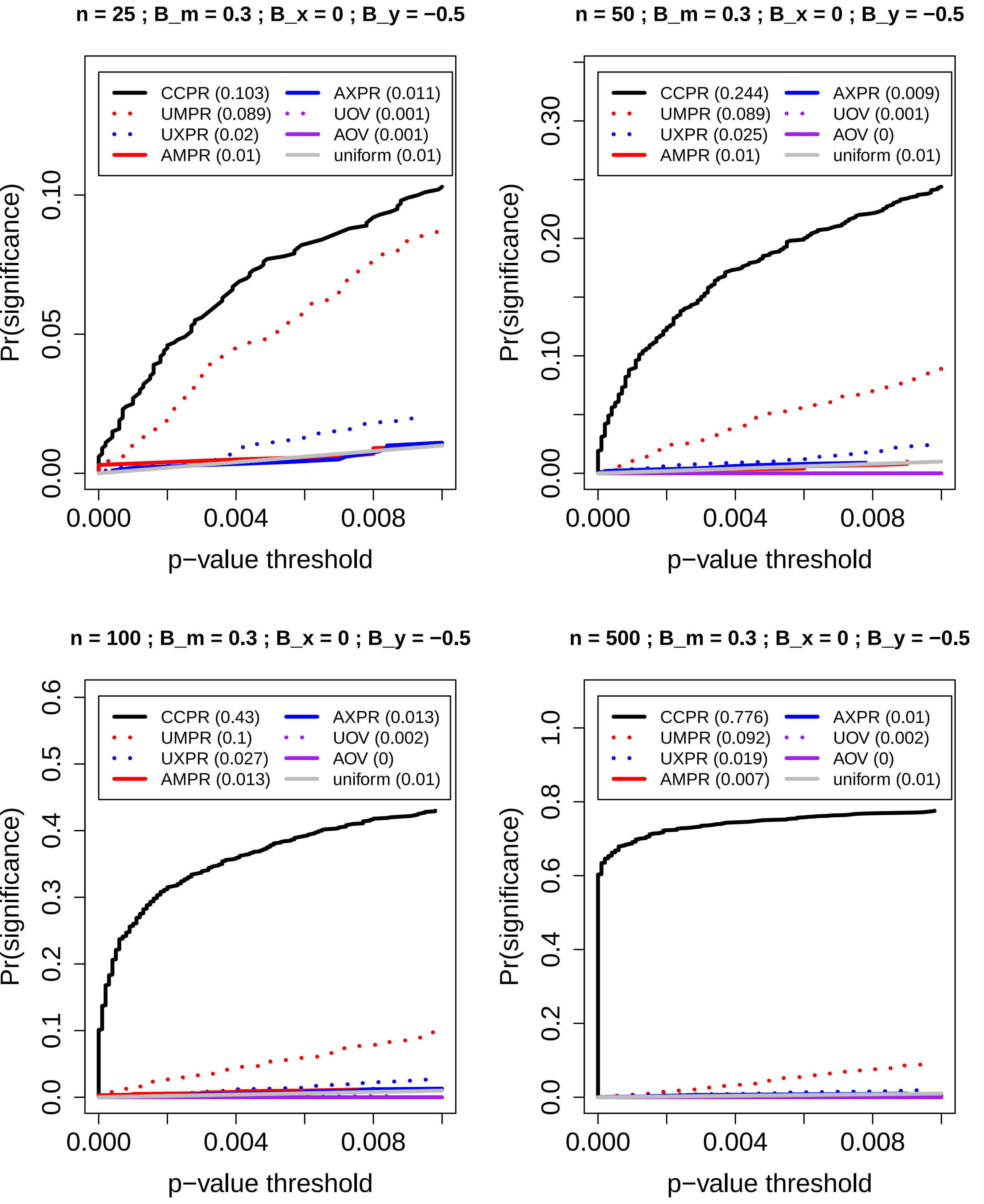
n = 25 ; B_m = 0.3 ; B_x = 0 ; B_y = −0.5
CCPR (0.103)
UMPR (0.089)
UXPR (0.02)
AMPR (0.01)
AXPR (0.011)
UOV (0.001)
AOV (0.001)
uniform (0.01)
Pr(significance)
p−value threshold
n = 50 ; B_m = 0.3 ; B_x = 0 ; B_y = −0.5
CCPR (0.244)
UMPR (0.089)
UXPR (0.025)
AMPR (0.01)
AXPR (0.009)
UOV (0.001)
AOV (0)
uniform (0.01)
Pr(significance)
p−value threshold
n = 100 ; B_m = 0.3 ; B_x = 0 ; B_y = −0.5
CCPR (0.43)
UMPR (0.1)
UXPR (0.027)
AMPR (0.013)
AXPR (0.013)
UOV (0.002)
AOV (0)
uniform (0.01)
Pr(significance)
p−value threshold
n = 500 ; B_m = 0.3 ; B_x = 0 ; B_y = −0.5
CCPR (0.776)
UMPR (0.092)
UXPR (0.019)
AMPR (0.007)
AXPR (0.01)
UOV (0.002)
AOV (0)
uniform (0.01)
Pr(significance)
p−value threshold

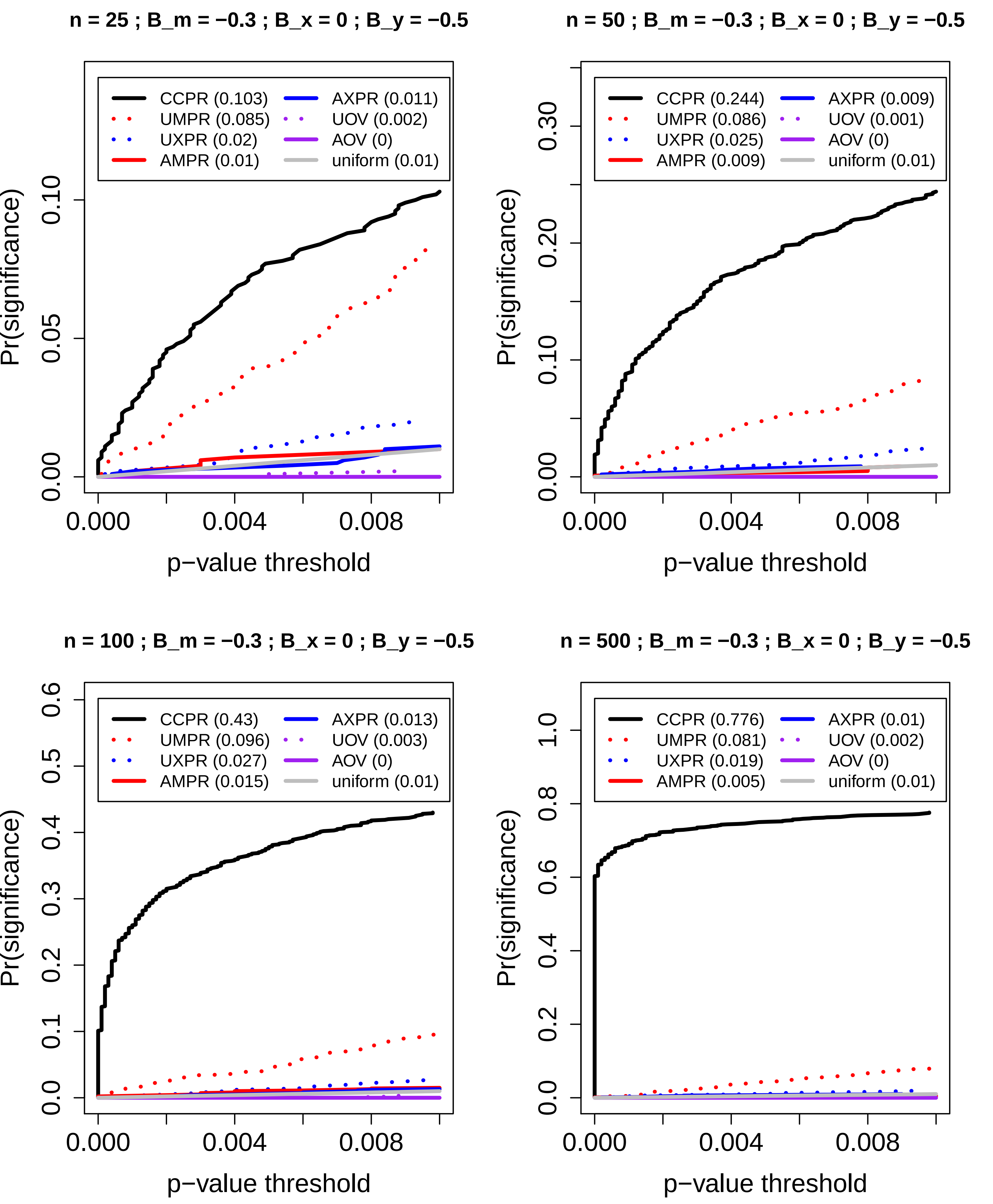
n = 25 ; B_m = −0.3 ; B_x = 0 ; B_y = −0.5
CCPR (0.103)
UMPR (0.085)
UXPR (0.02)
AMPR (0.01)
AXPR (0.011)
UOV (0.002)
AOV (0)
uniform (0.01)
n = 50 ; B_m = −0.3 ; B_x = 0 ; B_y = −0.5
CCPR (0.244)
UMPR (0.086)
UXPR (0.025)
AMPR (0.009)
AXPR (0.009)
UOV (0.001)
AOV (0)
uniform (0.01)
n = 100 ; B_m = −0.3 ; B_x = 0 ; B_y = −0.5
CCPR (0.43)
UMPR (0.096)
UXPR (0.027)
AMPR (0.015)
AXPR (0.013)
UOV (0.003)
AOV (0)
uniform (0.01)
n = 500 ; B_m = −0.3 ; B_x = 0 ; B_y = −0.5
CCPR (0.776)
UMPR (0.081)
UXPR (0.019)
AMPR (0.005)
AXPR (0.01)
UOV (0.002)
AOV (0)
uniform (0.01)
Pr(significance)
p−value threshold
0.000
0.004
0.008

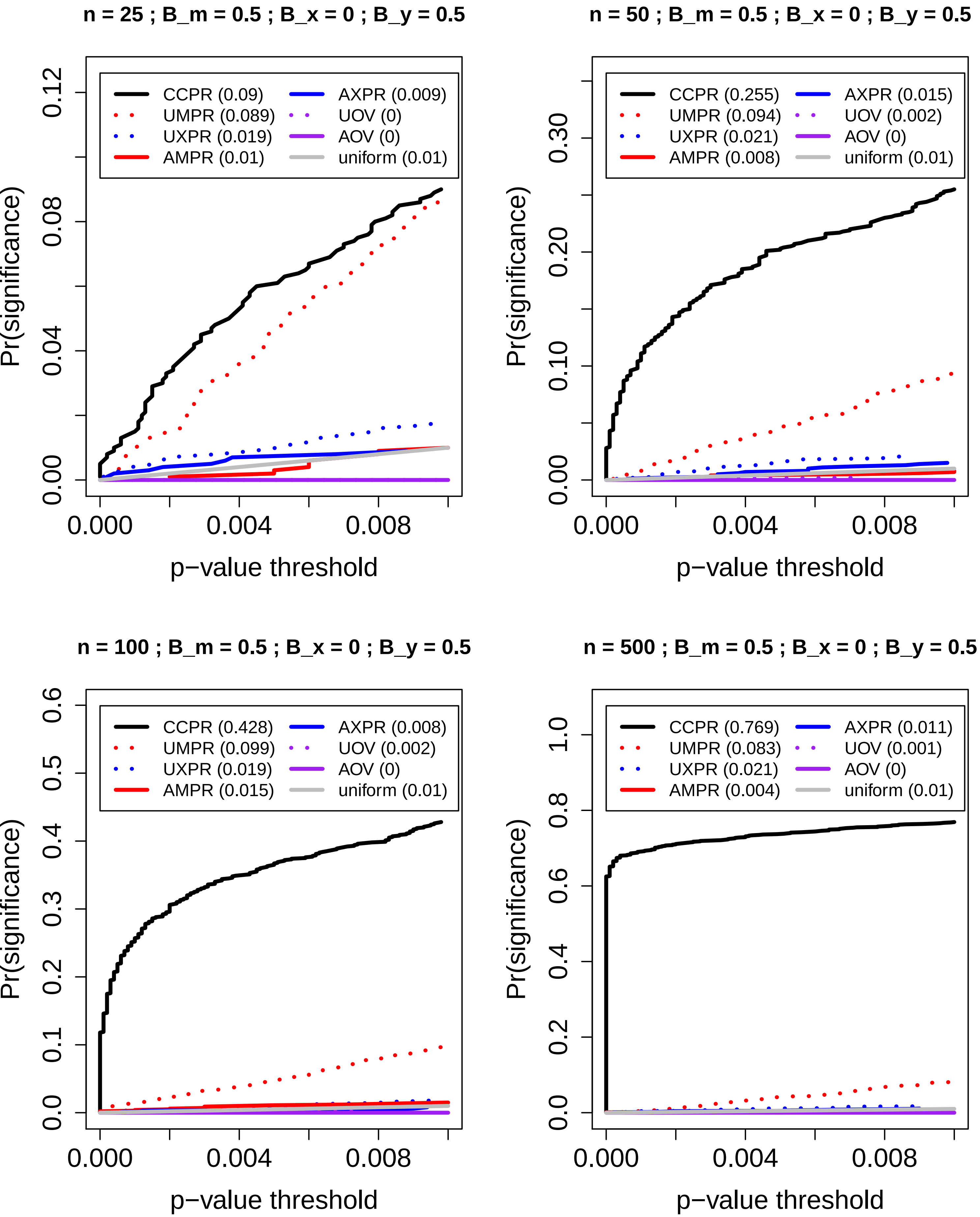

n = 25 ; B_m = 0.5 ; B_x = 0 ; B_y = 0.5
CCPR (0.09)
UMPR (0.089)
UXPR (0.019)
AMPR (0.01)
AXPR (0.009)
UOV (0)
AOV (0)
uniform (0.01)
n = 50 ; B_m = 0.5 ; B_x = 0 ; B_y = 0.5
CCPR (0.255)
UMPR (0.094)
UXPR (0.021)
AMPR (0.008)
AXPR (0.015)
UOV (0.002)
AOV (0)
uniform (0.01)
n = 100 ; B_m = 0.5 ; B_x = 0 ; B_y = 0.5
CCPR (0.428)
UMPR (0.099)
UXPR (0.019)
AMPR (0.015)
AXPR (0.008)
UOV (0.002)
AOV (0)
uniform (0.01)
n = 500 ; B_m = 0.5 ; B_x = 0 ; B_y = 0.5
CCPR (0.769)
UMPR (0.083)
UXPR (0.021)
AMPR (0.004)
AXPR (0.011)
UOV (0.001)
AOV (0)
uniform (0.01)
Pr(significance)
p−value threshold

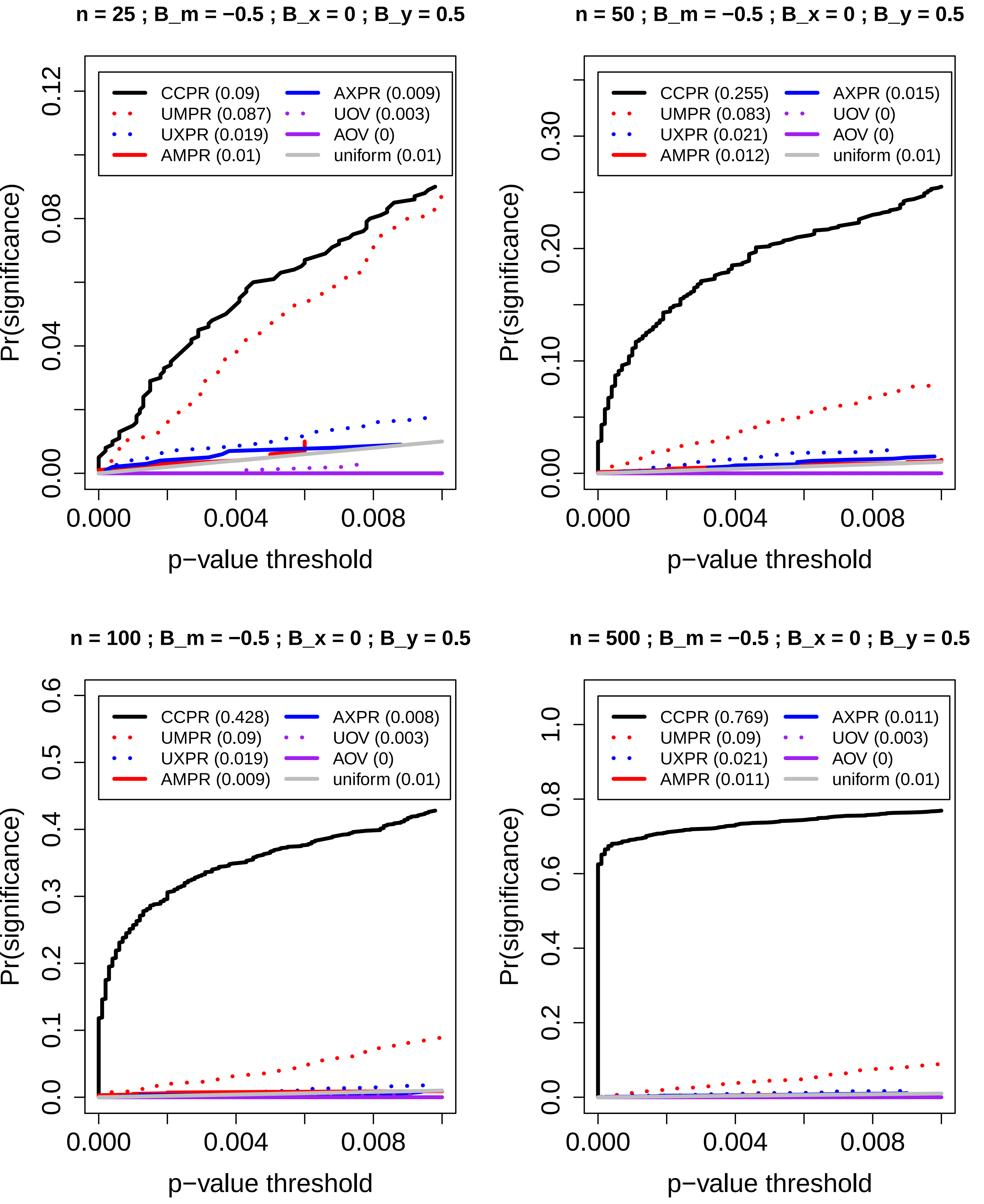
n = 25 ; B_m = −0.5 ; B_x = 0 ; B_y = 0.5
CCPR (0.09)
UMPR (0.087)
UXPR (0.019)
AMPR (0.01)
AXPR (0.009)
UOV (0.003)
AOV (0)
uniform (0.01)
n = 50 ; B_m = −0.5 ; B_x = 0 ; B_y = 0.5
CCPR (0.255)
UMPR (0.083)
UXPR (0.021)
AMPR (0.012)
AXPR (0.015)
UOV (0)
AOV (0)
uniform (0.01)
n = 100 ; B_m = −0.5 ; B_x = 0 ; B_y = 0.5
CCPR (0.428)
UMPR (0.09)
UXPR (0.019)
AMPR (0.009)
AXPR (0.008)
UOV (0.003)
AOV (0)
uniform (0.01)
n = 500 ; B_m = −0.5 ; B_x = 0 ; B_y = 0.5
CCPR (0.769)
UMPR (0.09)
UXPR (0.021)
AMPR (0.011)
AXPR (0.011)
UOV (0.003)
AOV (0)
uniform (0.01)
Pr(significance)
p−value threshold
0.000
0.004
0.008
0.00
0.04
0.08
0.12
0.10
0.20
0.30
0.0
0.1
0.2
0.3
0.4
0.5
0.6
0.8
1.0

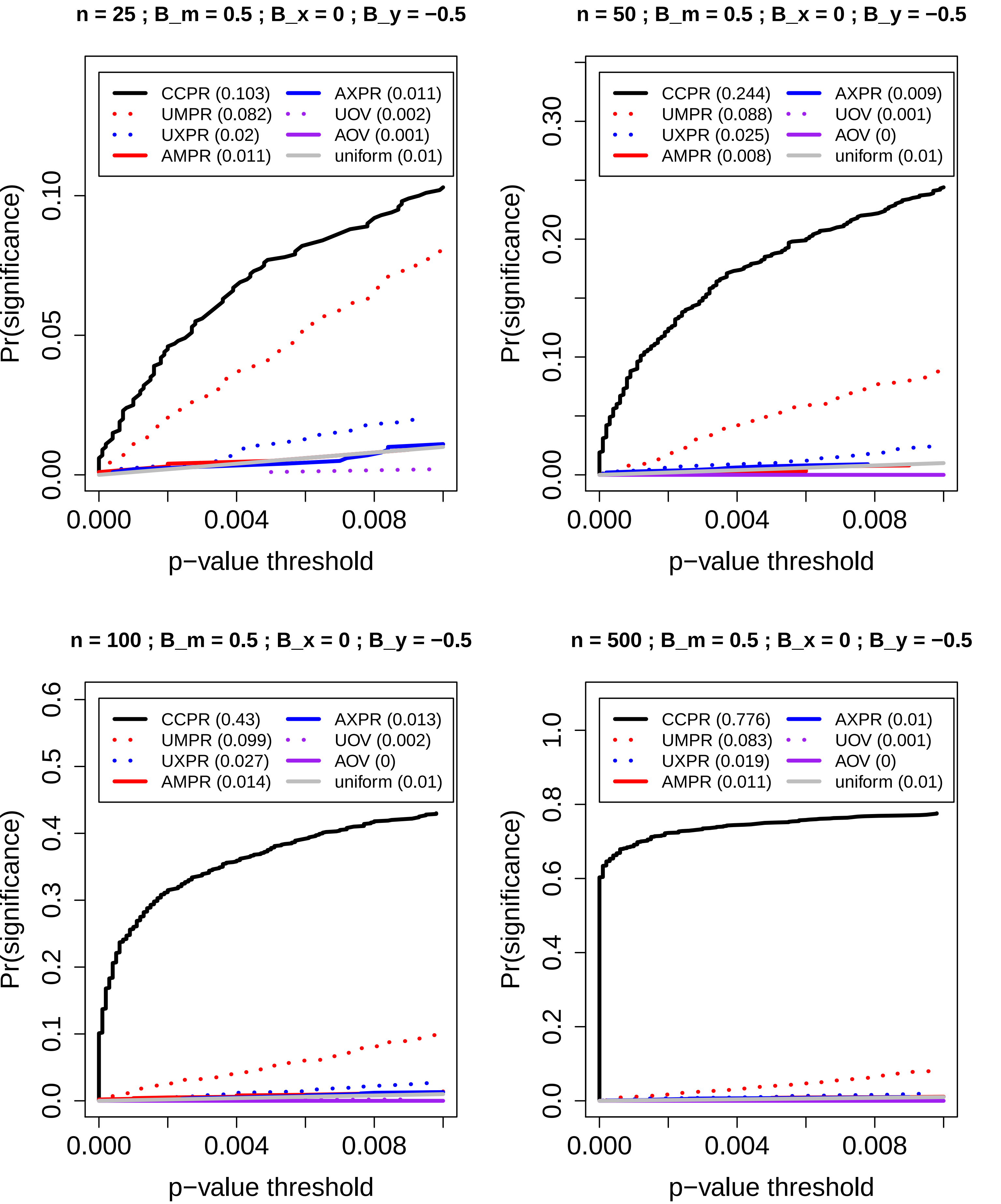
n = 25 ; B_m = 0.5 ; B_x = 0 ; B_y = −0.5
CCPR (0.103)
UMPR (0.082)
UXPR (0.02)
AMPR (0.011)
AXPR (0.011)
UOV (0.002)
AOV (0.001)
uniform (0.01)
Pr(significance)
p−value threshold
n = 50 ; B_m = 0.5 ; B_x = 0 ; B_y = −0.5
CCPR (0.244)
UMPR (0.088)
UXPR (0.025)
AMPR (0.008)
AXPR (0.009)
UOV (0.001)
AOV (0)
uniform (0.01)
Pr(significance)
p−value threshold
n = 100 ; B_m = 0.5 ; B_x = 0 ; B_y = −0.5
CCPR (0.43)
UMPR (0.099)
UXPR (0.027)
AMPR (0.014)
AXPR (0.013)
UOV (0.002)
AOV (0)
uniform (0.01)
Pr(significance)
p−value threshold
n = 500 ; B_m = 0.5 ; B_x = 0 ; B_y = −0.5
CCPR (0.776)
UMPR (0.083)
UXPR (0.019)
AMPR (0.011)
AXPR (0.01)
UOV (0.001)
AOV (0)
uniform (0.01)
Pr(significance)
p−value threshold

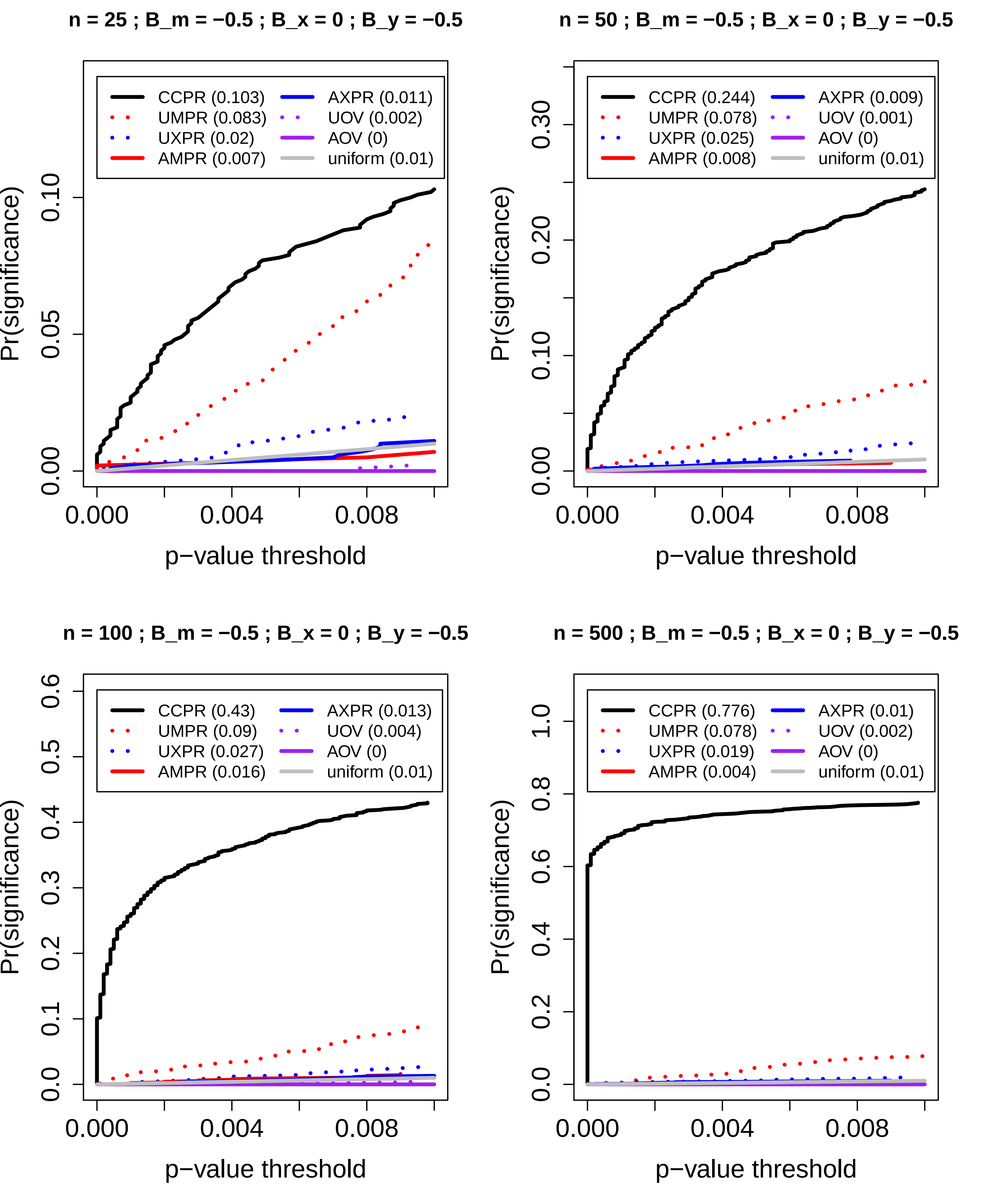

n = 25 ; B_m = −0.5 ; B_x = 0 ; B_y = −0.5
CCPR (0.103)
UMPR (0.083)
UXPR (0.02)
AMPR (0.007)
AXPR (0.011)
UOV (0.002)
AOV (0)
uniform (0.01)
Pr(significance)
p−value threshold
n = 50 ; B_m = −0.5 ; B_x = 0 ; B_y = −0.5
CCPR (0.244)
UMPR (0.078)
UXPR (0.025)
AMPR (0.008)
AXPR (0.009)
UOV (0.001)
AOV (0)
uniform (0.01)
Pr(significance)
p−value threshold
n = 100 ; B_m = −0.5 ; B_x = 0 ; B_y = −0.5
CCPR (0.43)
UMPR (0.09)
UXPR (0.027)
AMPR (0.016)
AXPR (0.013)
UOV (0.004)
AOV (0)
uniform (0.01)
Pr(significance)
p−value threshold
n = 500 ; B_m = −0.5 ; B_x = 0 ; B_y = −0.5
CCPR (0.776)
UMPR (0.078)
UXPR (0.019)
AMPR (0.004)
AXPR (0.01)
UOV (0.002)
AOV (0)
uniform (0.01)
Pr(significance)
p−value threshold

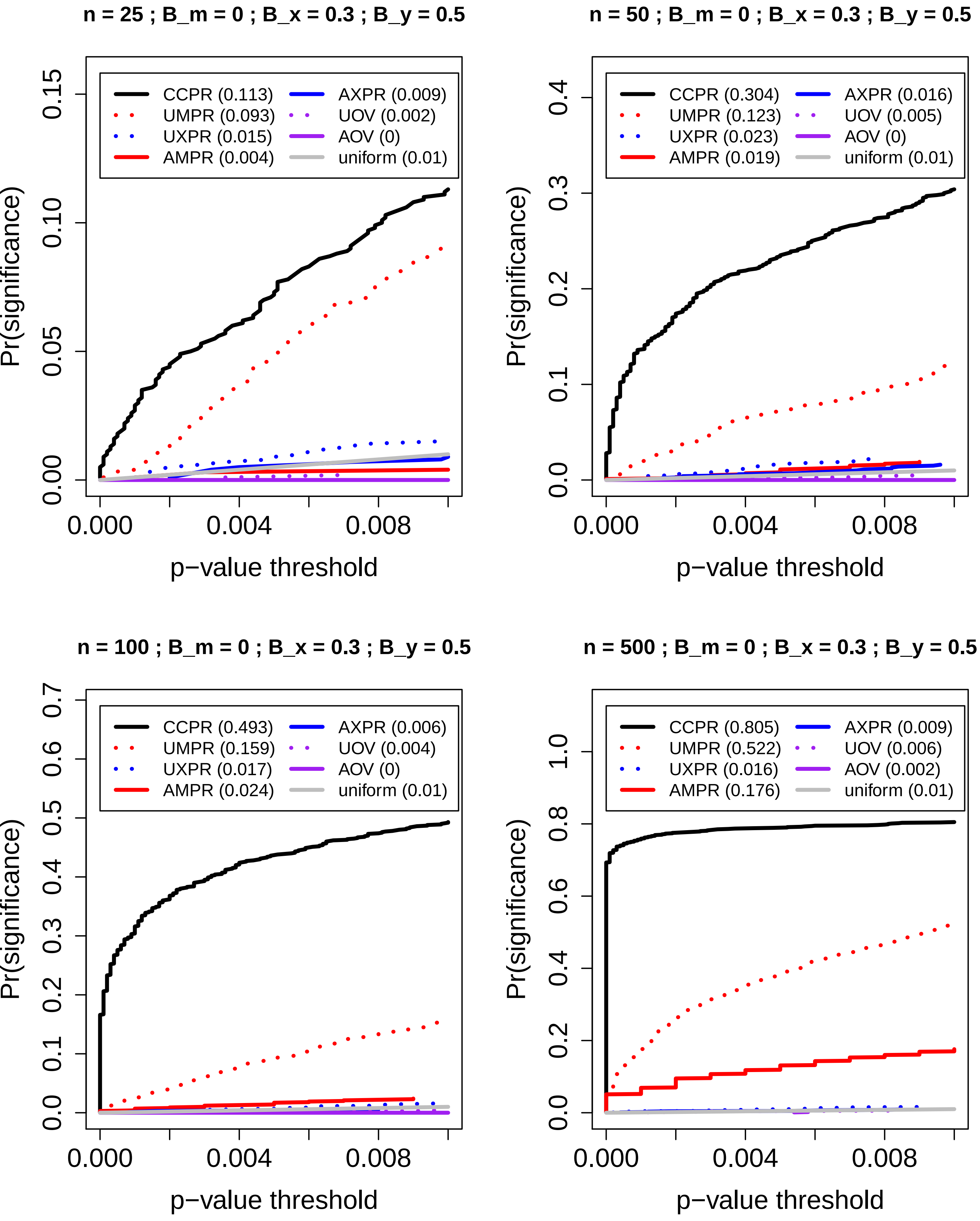

n = 25 ; B_m = 0 ; B_x = 0.3 ; B_y = 0.5
CCPR (0.113)
UMPR (0.093)
UXPR (0.015)
AMPR (0.004)
AXPR (0.009)
UOV (0.002)
AOV (0)
uniform (0.01)
n = 50 ; B_m = 0 ; B_x = 0.3 ; B_y = 0.5
CCPR (0.304)
UMPR (0.123)
UXPR (0.023)
AMPR (0.019)
AXPR (0.016)
UOV (0.005)
AOV (0)
uniform (0.01)
n = 100 ; B_m = 0 ; B_x = 0.3 ; B_y = 0.5
CCPR (0.493)
UMPR (0.159)
UXPR (0.017)
AMPR (0.024)
AXPR (0.006)
UOV (0.004)
AOV (0)
uniform (0.01)
n = 500 ; B_m = 0 ; B_x = 0.3 ; B_y = 0.5
CCPR (0.805)
UMPR (0.522)
UXPR (0.016)
AMPR (0.176)
AXPR (0.009)
UOV (0.006)
AOV (0.002)
uniform (0.01)
Pr(significance)
p−value threshold
0.000
0.004
0.008

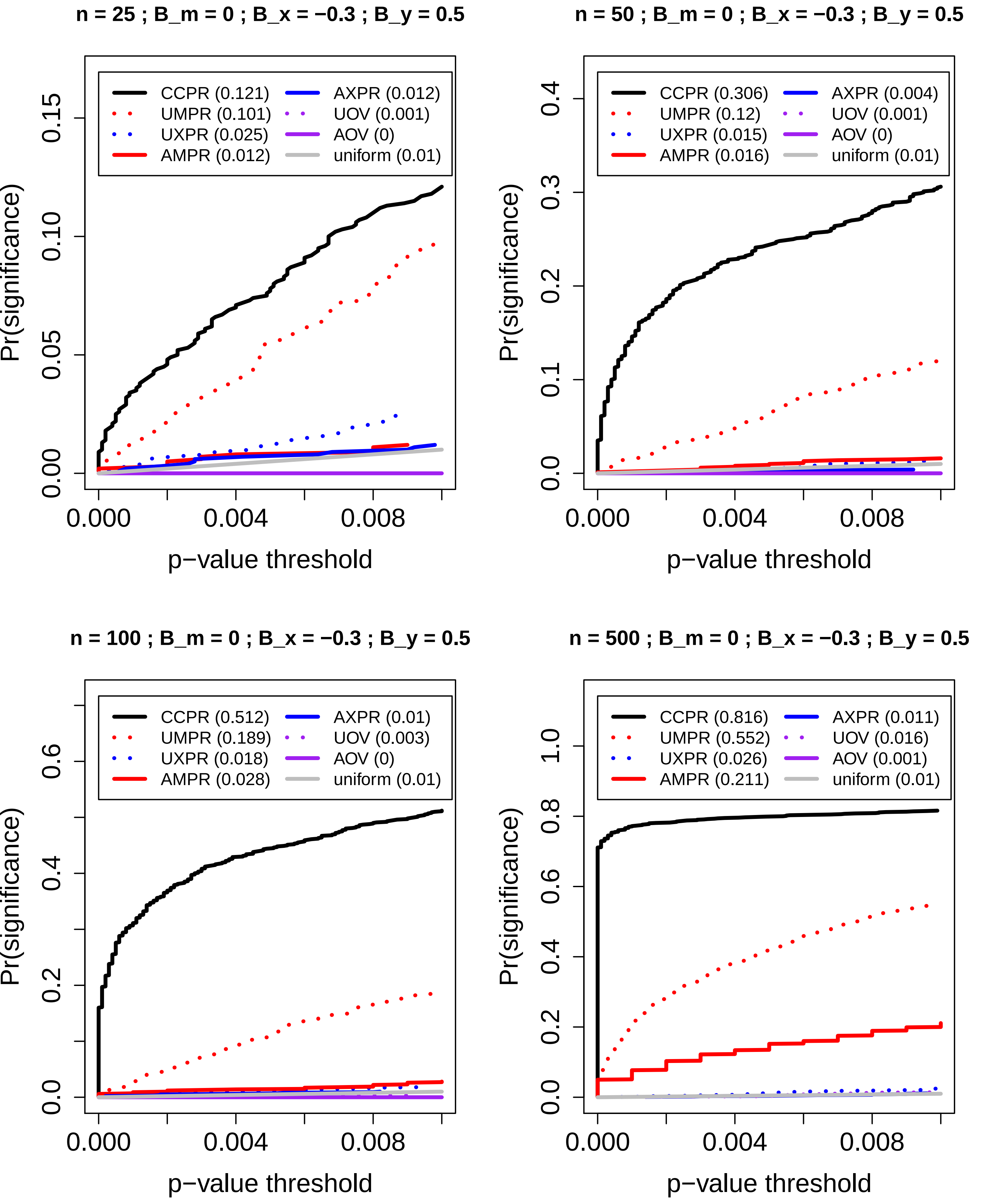
n = 25 ; B_m = 0 ; B_x = −0.3 ; B_y = 0.5
CCPR (0.121)
UMPR (0.101)
UXPR (0.025)
AMPR (0.012)
AXPR (0.012)
UOV (0.001)
AOV (0)
uniform (0.01)
n = 50 ; B_m = 0 ; B_x = −0.3 ; B_y = 0.5
CCPR (0.306)
UMPR (0.12)
UXPR (0.015)
AMPR (0.016)
AXPR (0.004)
UOV (0.001)
AOV (0)
uniform (0.01)
n = 100 ; B_m = 0 ; B_x = −0.3 ; B_y = 0.5
CCPR (0.512)
UMPR (0.189)
UXPR (0.018)
AMPR (0.028)
AXPR (0.01)
UOV (0.003)
AOV (0)
uniform (0.01)
n = 500 ; B_m = 0 ; B_x = −0.3 ; B_y = 0.5
CCPR (0.816)
UMPR (0.552)
UXPR (0.026)
AMPR (0.211)
AXPR (0.011)
UOV (0.016)
AOV (0.001)
uniform (0.01)
Pr(significance)
p−value threshold

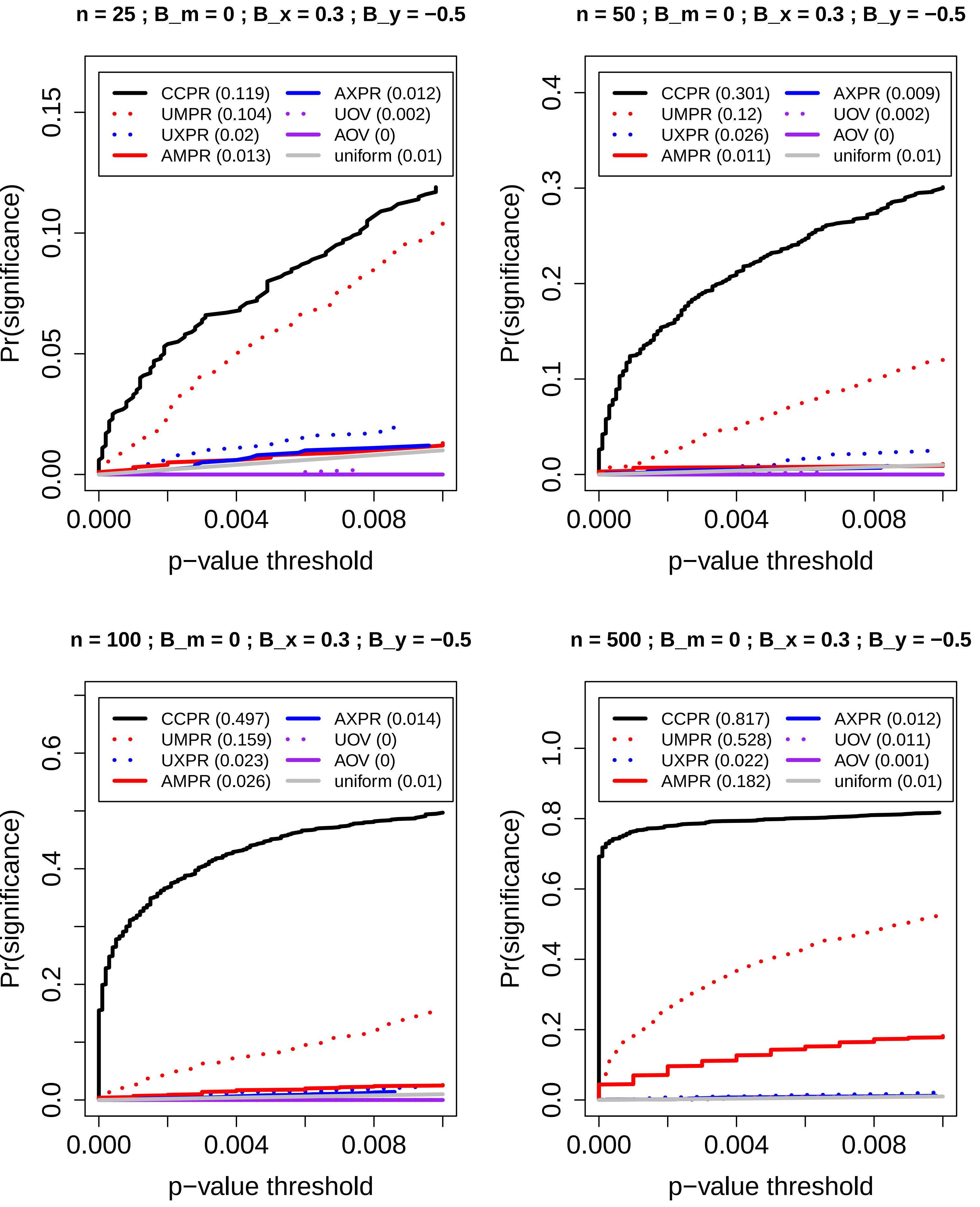
n = 25 ; B_m = 0 ; B_x = 0.3 ; B_y = −0.5
CCPR (0.119)
UMPR (0.104)
UXPR (0.02)
AMPR (0.013)
AXPR (0.012)
UOV (0.002)
AOV (0)
uniform (0.01)
Pr(significance)
p−value threshold
n = 50 ; B_m = 0 ; B_x = 0.3 ; B_y = −0.5
CCPR (0.301)
UMPR (0.12)
UXPR (0.026)
AMPR (0.011)
AXPR (0.009)
UOV (0.002)
AOV (0)
uniform (0.01)
Pr(significance)
p−value threshold
n = 100 ; B_m = 0 ; B_x = 0.3 ; B_y = −0.5
CCPR (0.497)
UMPR (0.159)
UXPR (0.023)
AMPR (0.026)
AXPR (0.014)
UOV (0)
AOV (0)
uniform (0.01)
Pr(significance)
p−value threshold
n = 500 ; B_m = 0 ; B_x = 0.3 ; B_y = −0.5
CCPR (0.817)
UMPR (0.528)
UXPR (0.022)
AMPR (0.182)
AXPR (0.012)
UOV (0.011)
AOV (0.001)
uniform (0.01)
Pr(significance)
p−value threshold

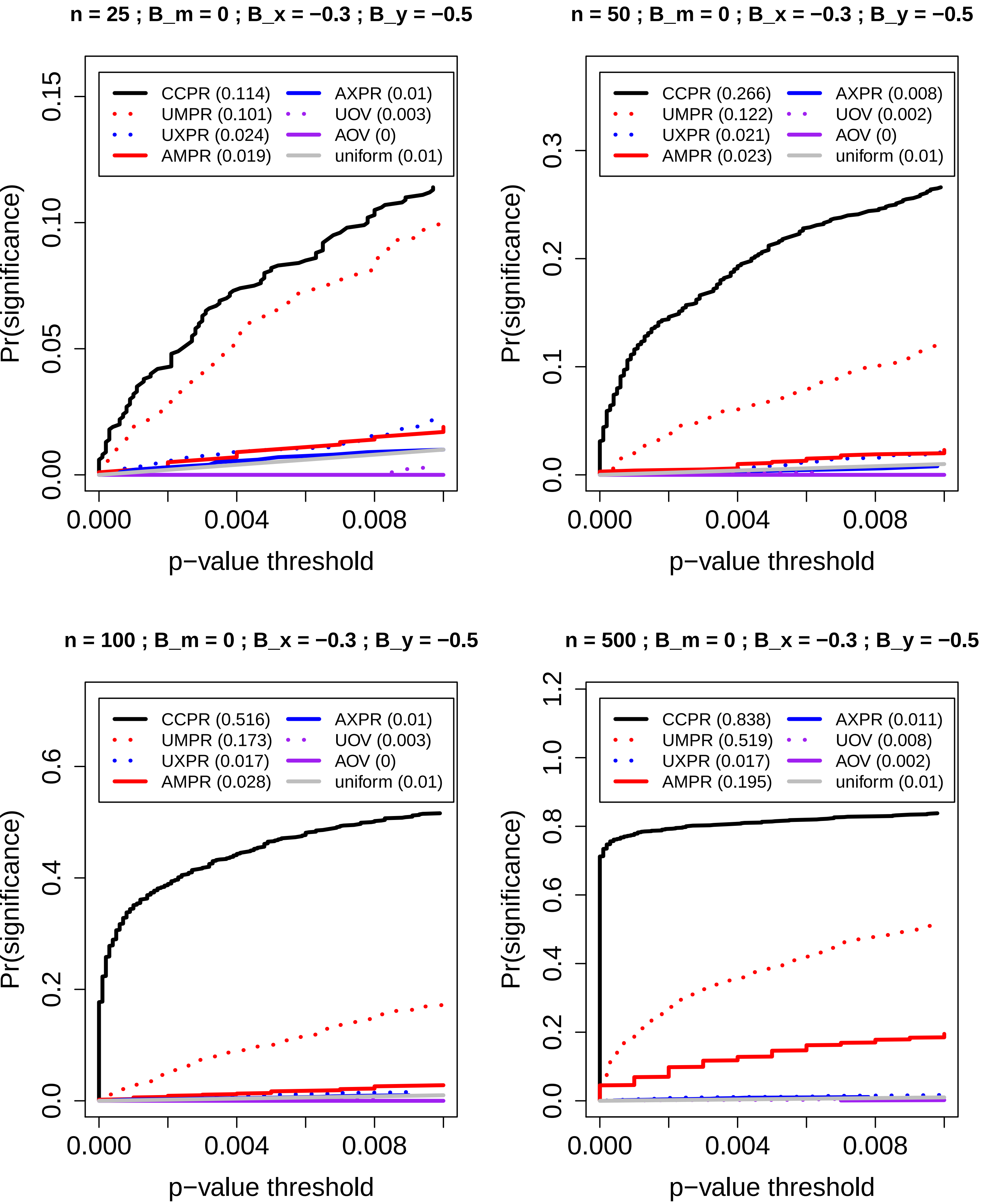
n = 25 ; B_m = 0 ; B_x = −0.3 ; B_y = −0.5
CCPR (0.114)
UMPR (0.101)
UXPR (0.024)
AMPR (0.019)
AXPR (0.01)
UOV (0.003)
AOV (0)
uniform (0.01)
Pr(significance)
p−value threshold
n = 50 ; B_m = 0 ; B_x = −0.3 ; B_y = −0.5
CCPR (0.266)
UMPR (0.122)
UXPR (0.021)
AMPR (0.023)
AXPR (0.008)
UOV (0.002)
AOV (0)
uniform (0.01)
Pr(significance)
p−value threshold
n = 100 ; B_m = 0 ; B_x = −0.3 ; B_y = −0.5
CCPR (0.516)
UMPR (0.173)
UXPR (0.017)
AMPR (0.028)
AXPR (0.01)
UOV (0.003)
AOV (0)
uniform (0.01)
Pr(significance)
p−value threshold
n = 500 ; B_m = 0 ; B_x = −0.3 ; B_y = −0.5
CCPR (0.838)
UMPR (0.519)
UXPR (0.017)
AMPR (0.195)
AXPR (0.011)
UOV (0.008)
AOV (0.002)
uniform (0.01)
Pr(significance)
p−value threshold

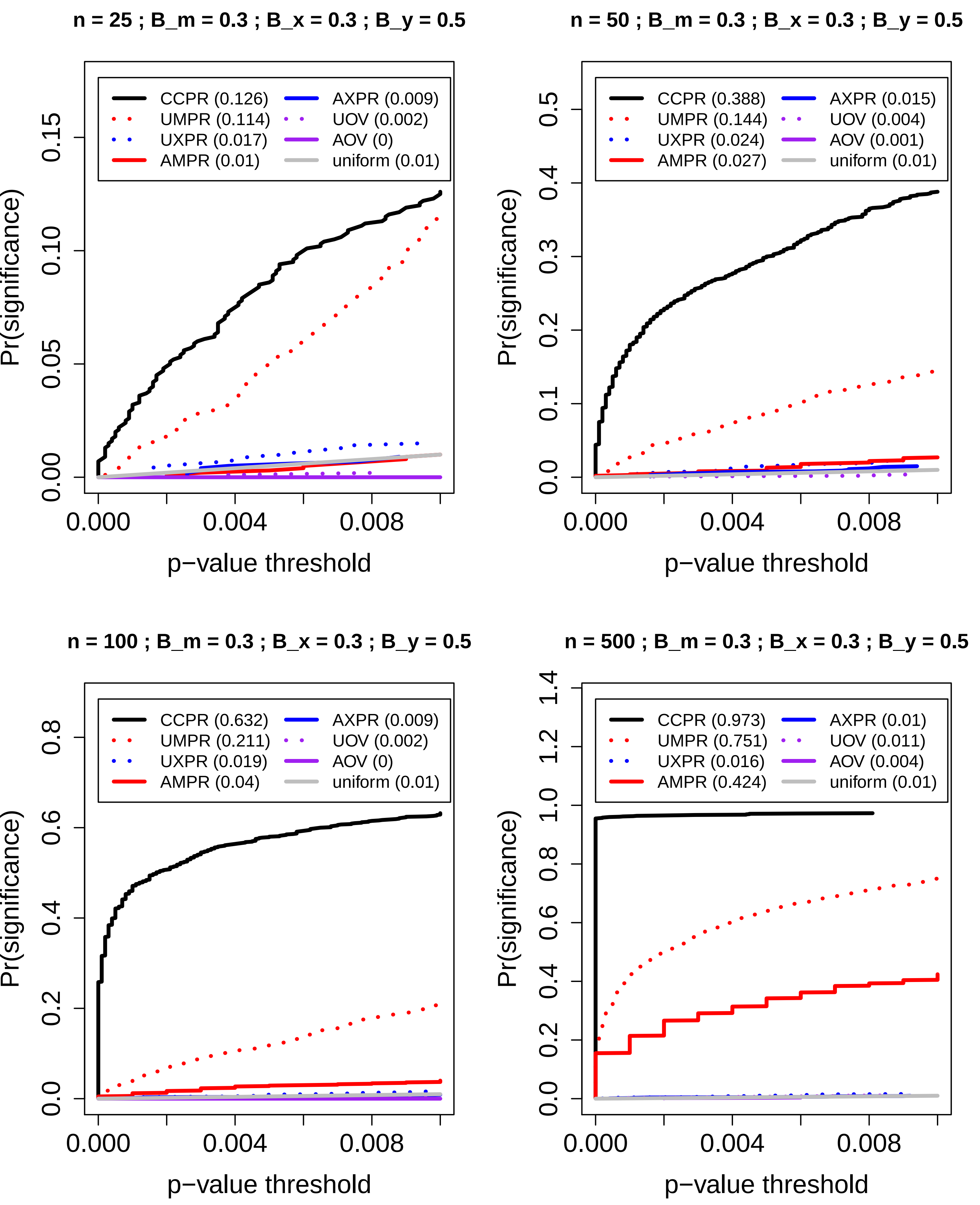

n = 25 ; B_m = 0.3 ; B_x = 0.3 ; B_y = 0.5
CCPR (0.126)
UMPR (0.114)
UXPR (0.017)
AMPR (0.01)
AXPR (0.009)
UOV (0.002)
AOV (0)
uniform (0.01)
Pr(significance)
p−value threshold
n = 50 ; B_m = 0.3 ; B_x = 0.3 ; B_y = 0.5
CCPR (0.388)
UMPR (0.144)
UXPR (0.024)
AMPR (0.027)
AXPR (0.015)
UOV (0.004)
AOV (0.001)
uniform (0.01)
Pr(significance)
p−value threshold
n = 100 ; B_m = 0.3 ; B_x = 0.3 ; B_y = 0.5
CCPR (0.632)
UMPR (0.211)
UXPR (0.019)
AMPR (0.04)
AXPR (0.009)
UOV (0.002)
AOV (0)
uniform (0.01)
Pr(significance)
p−value threshold
n = 500 ; B_m = 0.3 ; B_x = 0.3 ; B_y = 0.5
CCPR (0.973)
UMPR (0.751)
UXPR (0.016)
AMPR (0.424)
AXPR (0.01)
UOV (0.011)
AOV (0.004)
uniform (0.01)
Pr(significance)
p−value threshold

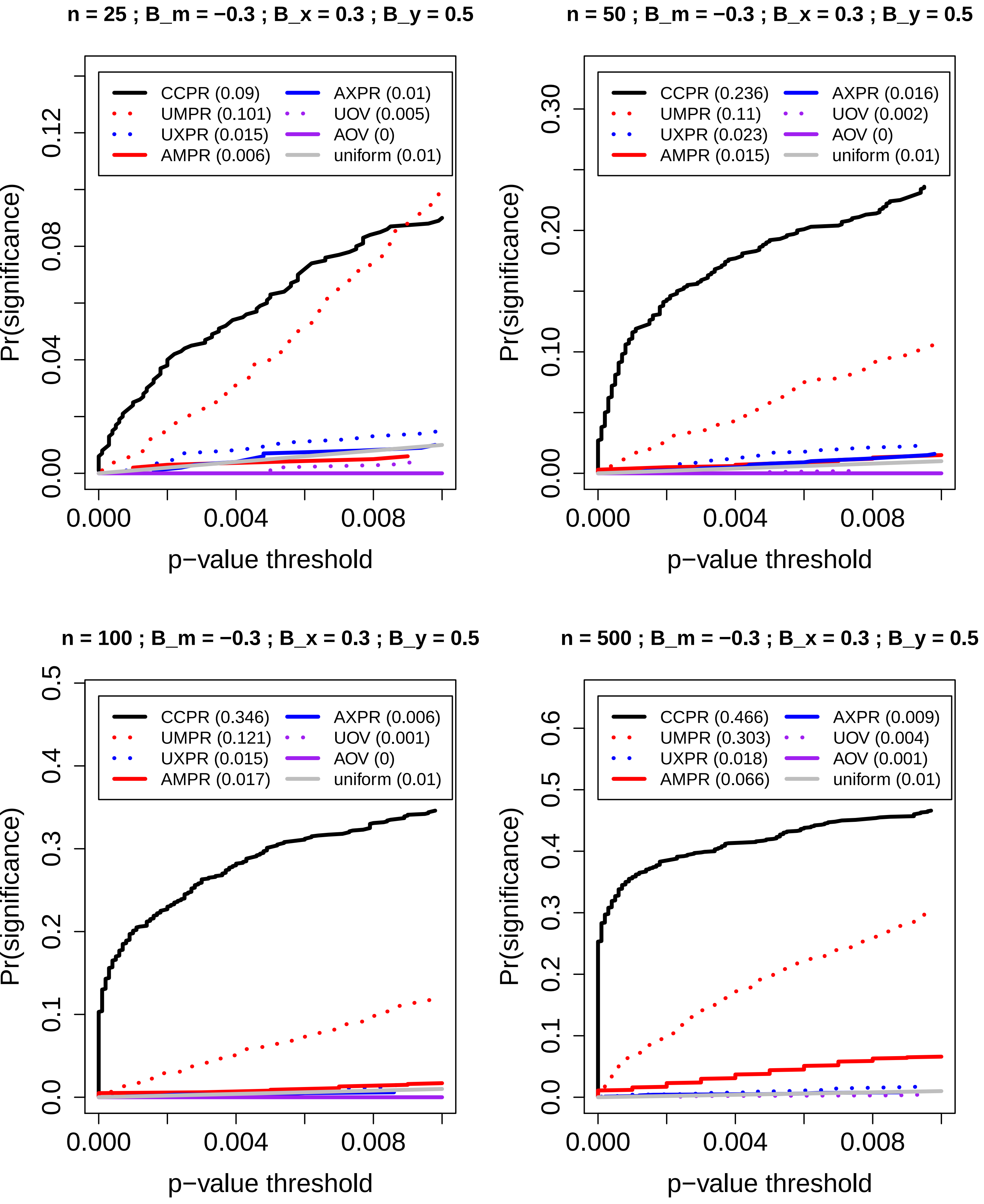

n = 25 ; B_m = −0.3 ; B_x = 0.3 ; B_y = 0.5
CCPR (0.09)
UMPR (0.101)
UXPR (0.015)
AMPR (0.006)
AXPR (0.01)
UOV (0.005)
AOV (0)
uniform (0.01)
Pr(significance)
p−value threshold
n = 50 ; B_m = −0.3 ; B_x = 0.3 ; B_y = 0.5
CCPR (0.236)
UMPR (0.11)
UXPR (0.023)
AMPR (0.015)
AXPR (0.016)
UOV (0.002)
AOV (0)
uniform (0.01)
Pr(significance)
p−value threshold
n = 100 ; B_m = −0.3 ; B_x = 0.3 ; B_y = 0.5
CCPR (0.346)
UMPR (0.121)
UXPR (0.015)
AMPR (0.017)
AXPR (0.006)
UOV (0.001)
AOV (0)
uniform (0.01)
Pr(significance)
p−value threshold
n = 500 ; B_m = −0.3 ; B_x = 0.3 ; B_y = 0.5
CCPR (0.466)
UMPR (0.303)
UXPR (0.018)
AMPR (0.066)
AXPR (0.009)
UOV (0.004)
AOV (0.001)
uniform (0.01)
Pr(significance)
p−value threshold

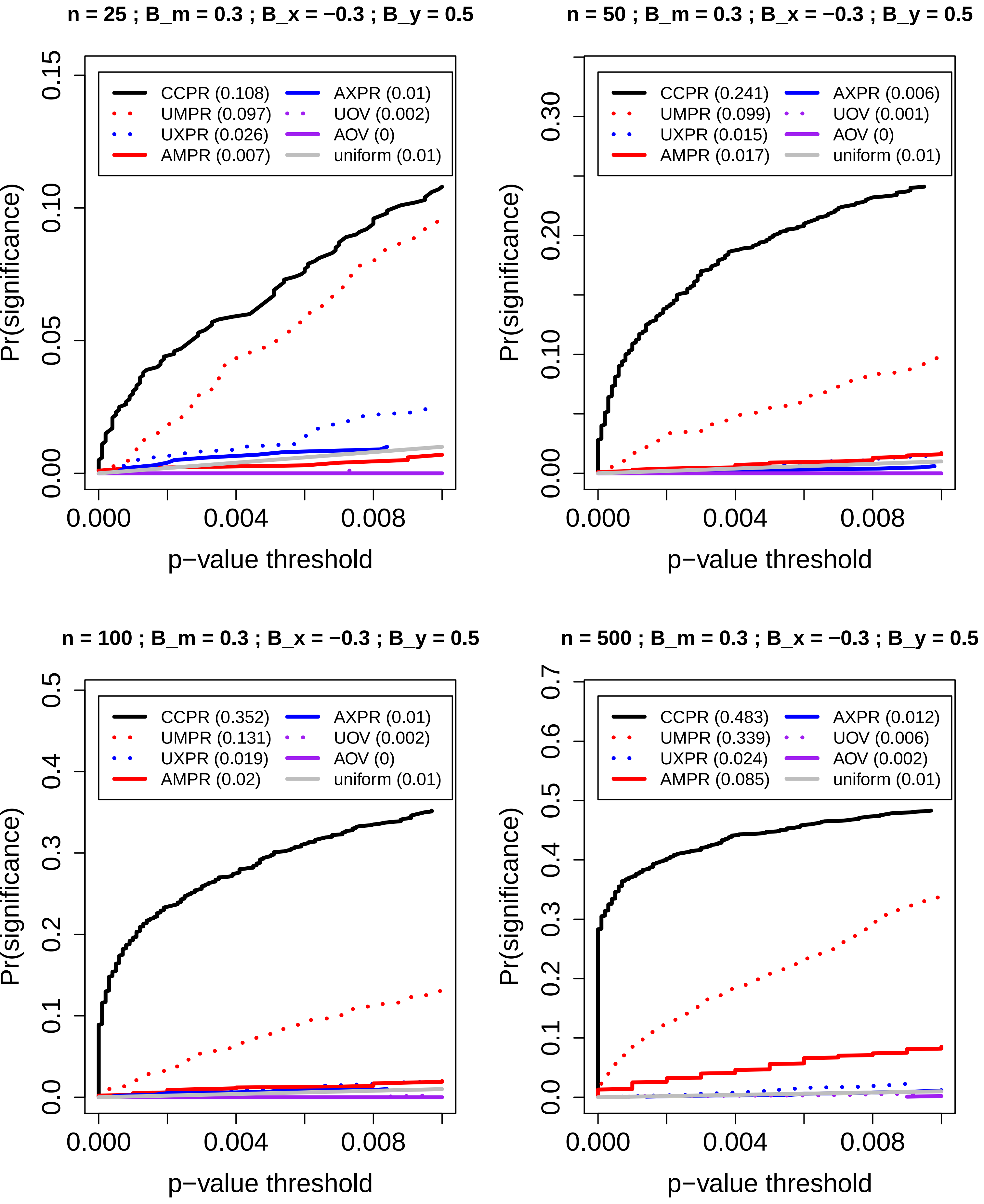

n = 25 ; B_m = 0.3 ; B_x = −0.3 ; B_y = 0.5
CCPR (0.108)
UMPR (0.097)
UXPR (0.026)
AMPR (0.007)
AXPR (0.01)
UOV (0.002)
AOV (0)
uniform (0.01)
n = 50 ; B_m = 0.3 ; B_x = −0.3 ; B_y = 0.5
CCPR (0.241)
UMPR (0.099)
UXPR (0.015)
AMPR (0.017)
AXPR (0.006)
UOV (0.001)
AOV (0)
uniform (0.01)
n = 100 ; B_m = 0.3 ; B_x = −0.3 ; B_y = 0.5
CCPR (0.352)
UMPR (0.131)
UXPR (0.019)
AMPR (0.02)
AXPR (0.01)
UOV (0.002)
AOV (0)
uniform (0.01)
n = 500 ; B_m = 0.3 ; B_x = −0.3 ; B_y = 0.5
CCPR (0.483)
UMPR (0.339)
UXPR (0.024)
AMPR (0.085)
AXPR (0.012)
UOV (0.006)
AOV (0.002)
uniform (0.01)
Pr(significance)
p−value threshold

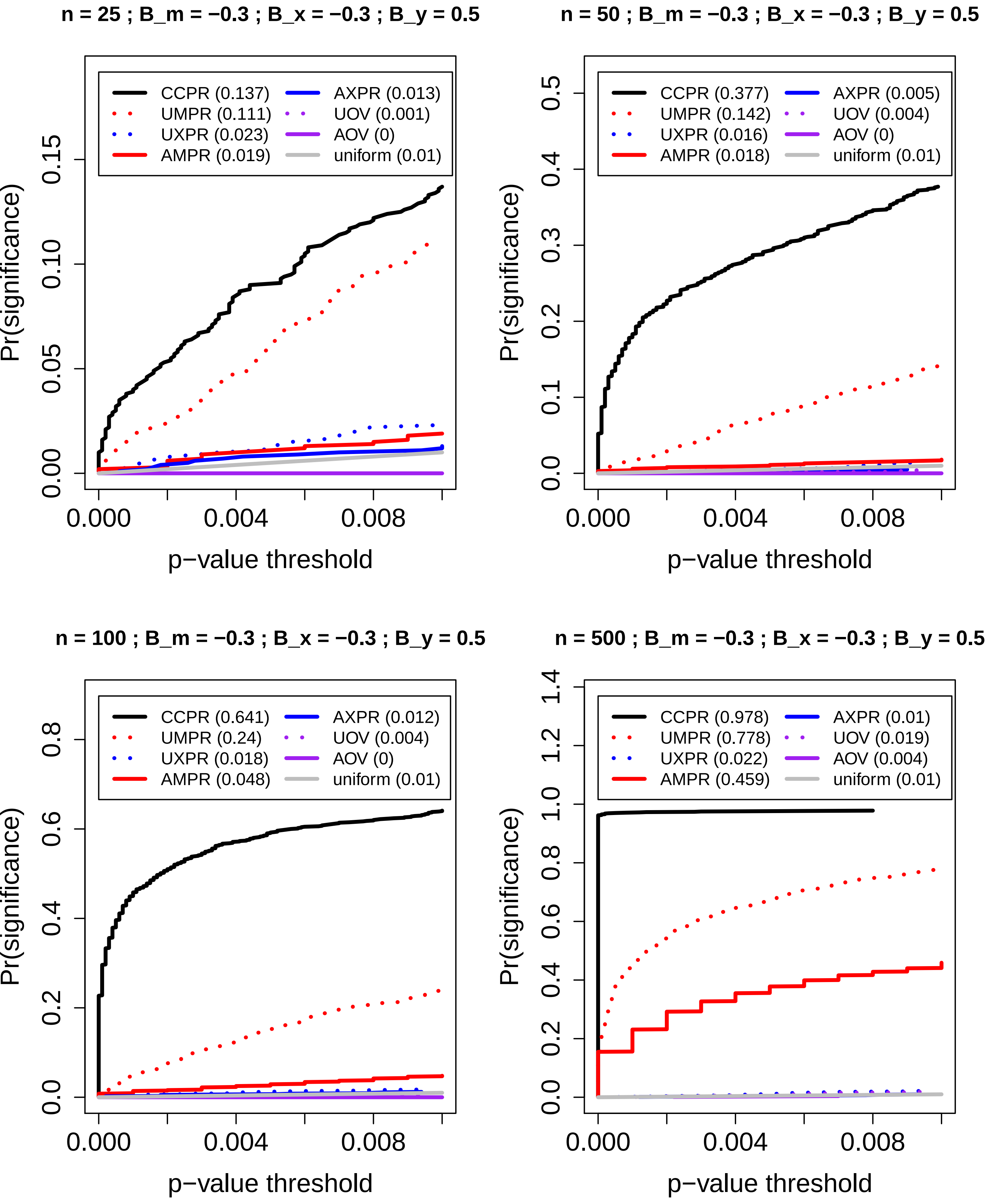

n = 25 ; B_m = −0.3 ; B_x = −0.3 ; B_y = 0.5
CCPR (0.137)
UMPR (0.111)
UXPR (0.023)
AMPR (0.019)
AXPR (0.013)
UOV (0.001)
AOV (0)
uniform (0.01)
Pr(significance)
p−value threshold
n = 50 ; B_m = −0.3 ; B_x = −0.3 ; B_y = 0.5
CCPR (0.377)
UMPR (0.142)
UXPR (0.016)
AMPR (0.018)
AXPR (0.005)
UOV (0.004)
AOV (0)
uniform (0.01)
Pr(significance)
p−value threshold
n = 100 ; B_m = −0.3 ; B_x = −0.3 ; B_y = 0.5
CCPR (0.641)
UMPR (0.24)
UXPR (0.018)
AMPR (0.048)
AXPR (0.012)
UOV (0.004)
AOV (0)
uniform (0.01)
Pr(significance)
p−value threshold
n = 500 ; B_m = −0.3 ; B_x = −0.3 ; B_y = 0.5
CCPR (0.978)
UMPR (0.778)
UXPR (0.022)
AMPR (0.459)
AXPR (0.01)
UOV (0.019)
AOV (0.004)
uniform (0.01)
Pr(significance)
p−value threshold

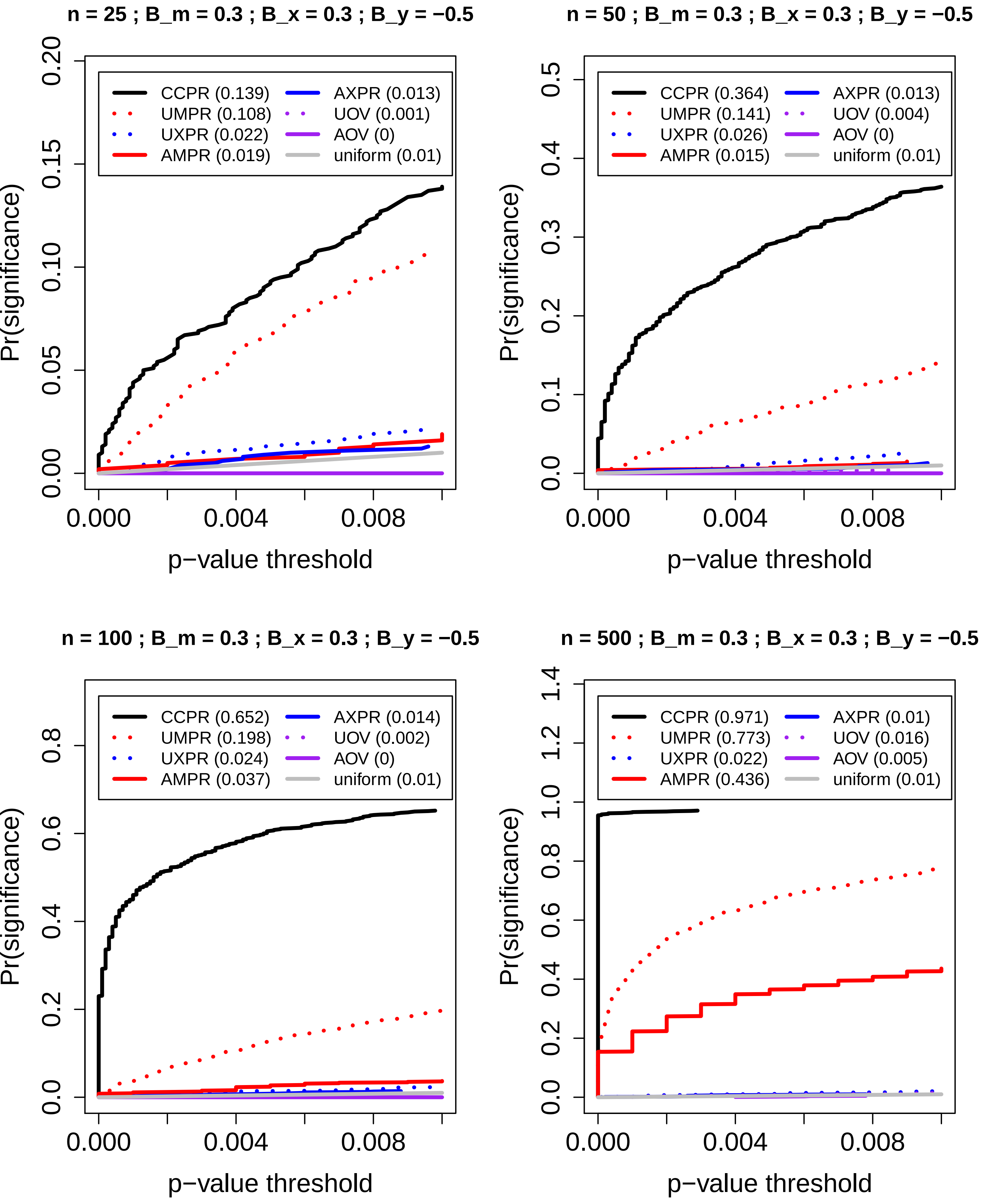
n = 25 ; B_m = 0.3 ; B_x = 0.3 ; B_y = −0.5
CCPR (0.139)
UMPR (0.108)
UXPR (0.022)
AMPR (0.019)
AXPR (0.013)
UOV (0.001)
AOV (0)
uniform (0.01)
n = 50 ; B_m = 0.3 ; B_x = 0.3 ; B_y = −0.5
CCPR (0.364)
UMPR (0.141)
UXPR (0.026)
AMPR (0.015)
AXPR (0.013)
UOV (0.004)
AOV (0)
uniform (0.01)
n = 100 ; B_m = 0.3 ; B_x = 0.3 ; B_y = −0.5
CCPR (0.652)
UMPR (0.198)
UXPR (0.024)
AMPR (0.037)
AXPR (0.014)
UOV (0.002)
AOV (0)
uniform (0.01)
n = 500 ; B_m = 0.3 ; B_x = 0.3 ; B_y = −0.5
CCPR (0.971)
UMPR (0.773)
UXPR (0.022)
AMPR (0.436)
AXPR (0.01)
UOV (0.016)
AOV (0.005)
uniform (0.01)
Pr(significance)
p−value threshold

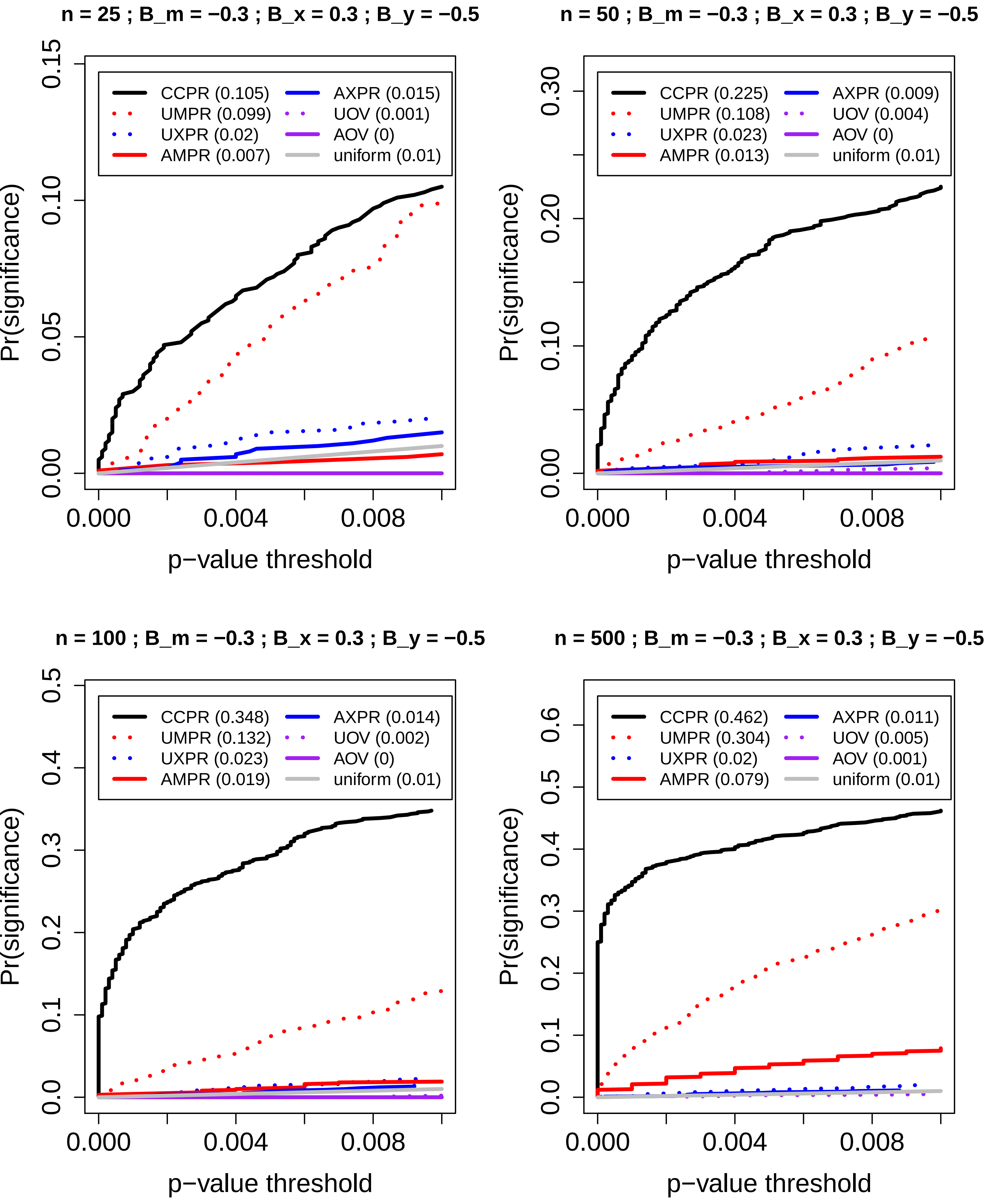
n = 25 ; B_m = −0.3 ; B_x = 0.3 ; B_y = −0.5
CCPR (0.105)
UMPR (0.099)
UXPR (0.02)
AMPR (0.007)
AXPR (0.015)
UOV (0.001)
AOV (0)
uniform (0.01)
Pr(significance)
p−value threshold
n = 50 ; B_m = −0.3 ; B_x = 0.3 ; B_y = −0.5
CCPR (0.225)
UMPR (0.108)
UXPR (0.023)
AMPR (0.013)
AXPR (0.009)
UOV (0.004)
AOV (0)
uniform (0.01)
Pr(significance)
p−value threshold
n = 100 ; B_m = −0.3 ; B_x = 0.3 ; B_y = −0.5
CCPR (0.348)
UMPR (0.132)
UXPR (0.023)
AMPR (0.019)
AXPR (0.014)
UOV (0.002)
AOV (0)
uniform (0.01)
Pr(significance)
p−value threshold
n = 500 ; B_m = −0.3 ; B_x = 0.3 ; B_y = −0.5
CCPR (0.462)
UMPR (0.304)
UXPR (0.02)
AMPR (0.079)
AXPR (0.011)
UOV (0.005)
AOV (0.001)
uniform (0.01)
Pr(significance)
p−value threshold

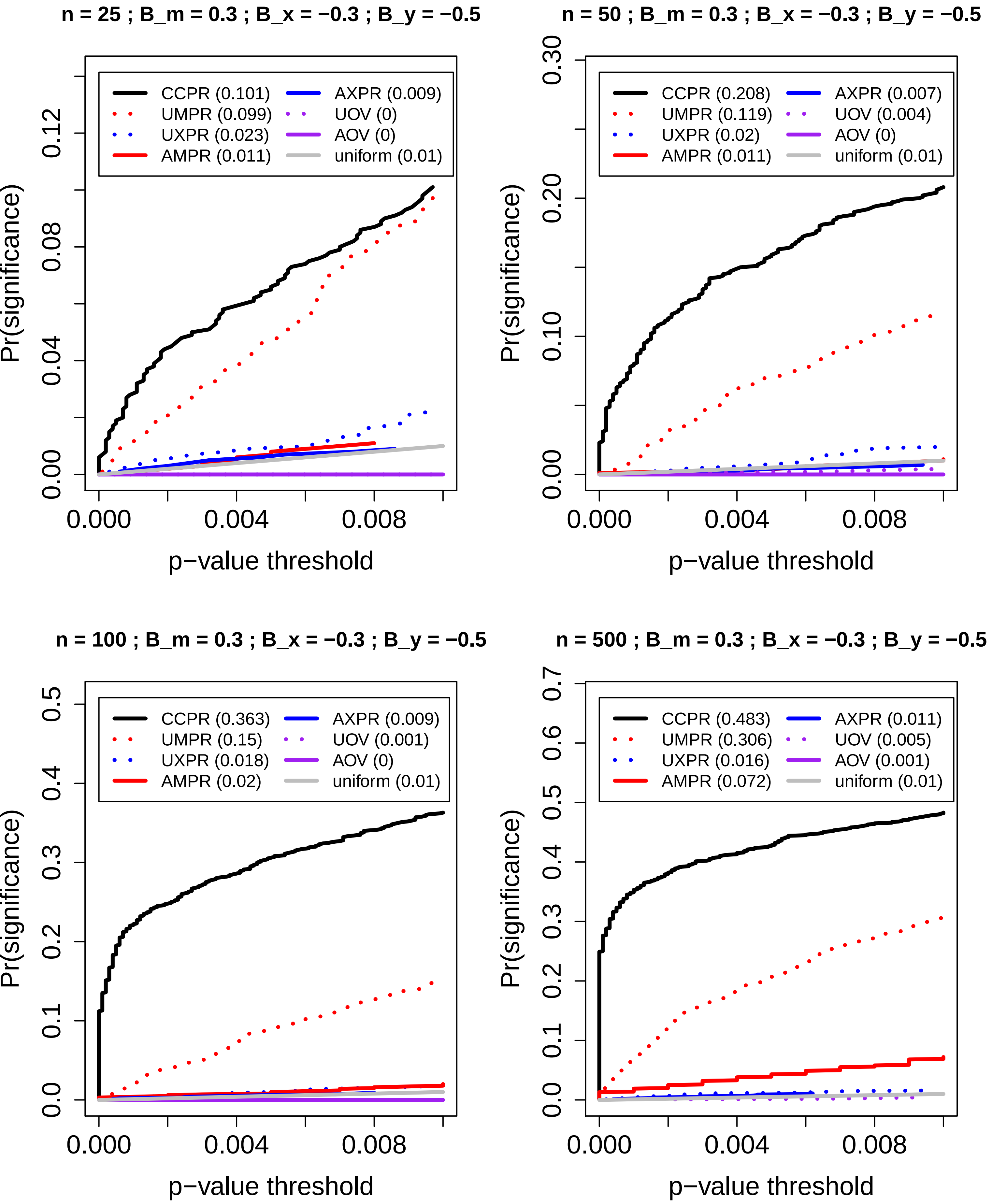
n = 25 ; B_m = 0.3 ; B_x = −0.3 ; B_y = −0.5
CCPR (0.101)
UMPR (0.099)
UXPR (0.023)
AMPR (0.011)
AXPR (0.009)
UOV (0)
AOV (0)
uniform (0.01)
Pr(significance)
0.00
0.04
0.08
0.12
0.000
0.004
0.008
p−value threshold
n = 50 ; B_m = 0.3 ; B_x = −0.3 ; B_y = −0.5
CCPR (0.208)
UMPR (0.119)
UXPR (0.02)
AMPR (0.011)
AXPR (0.007)
UOV (0.004)
AOV (0)
uniform (0.01)
Pr(significance)
0.00
0.10
0.20
0.30
0.000
0.004
0.008
p−value threshold
n = 100 ; B_m = 0.3 ; B_x = −0.3 ; B_y = −0.5
CCPR (0.363)
UMPR (0.15)
UXPR (0.018)
AMPR (0.02)
AXPR (0.009)
UOV (0.001)
AOV (0)
uniform (0.01)
Pr(significance)
0.0
0.1
0.2
0.3
0.4
0.5
0.000
0.004
0.008
p−value threshold
n = 500 ; B_m = 0.3 ; B_x = −0.3 ; B_y = −0.5
CCPR (0.483)
UMPR (0.306)
UXPR (0.016)
AMPR (0.072)
AXPR (0.011)
UOV (0.005)
AOV (0.001)
uniform (0.01)
Pr(significance)
0.0
0.1
0.2
0.3
0.4
0.5
0.6
0.7
0.000
0.004
0.008
p−value threshold

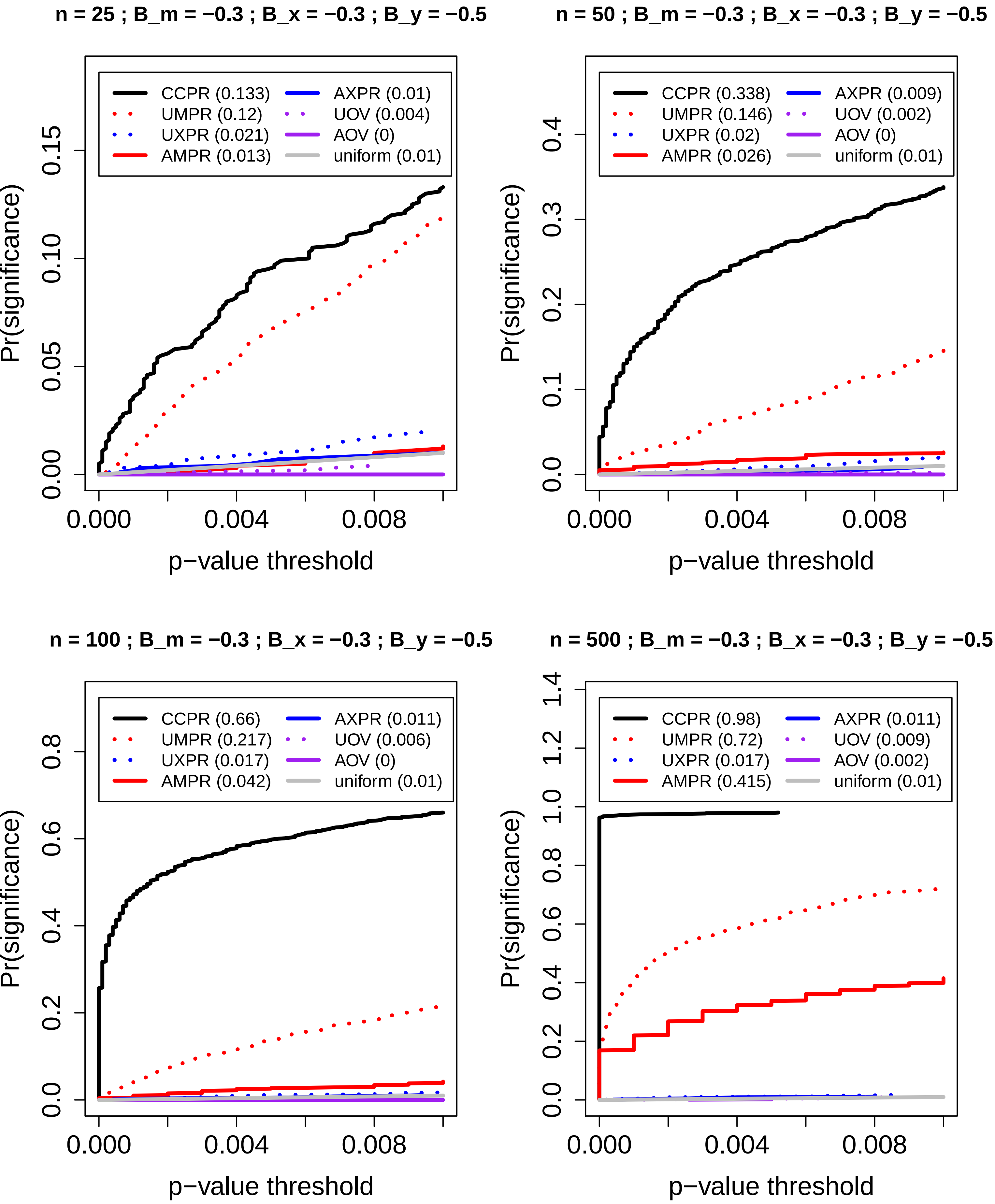
n = 25 ; B_m = −0.3 ; B_x = −0.3 ; B_y = −0.5
CCPR (0.133)
UMPR (0.12)
UXPR (0.021)
AMPR (0.013)
AXPR (0.01)
UOV (0.004)
AOV (0)
uniform (0.01)
Pr(significance)
p−value threshold
n = 50 ; B_m = −0.3 ; B_x = −0.3 ; B_y = −0.5
CCPR (0.338)
UMPR (0.146)
UXPR (0.02)
AMPR (0.026)
AXPR (0.009)
UOV (0.002)
AOV (0)
uniform (0.01)
Pr(significance)
p−value threshold
n = 100 ; B_m = −0.3 ; B_x = −0.3 ; B_y = −0.5
CCPR (0.66)
UMPR (0.217)
UXPR (0.017)
AMPR (0.042)
AXPR (0.011)
UOV (0.006)
AOV (0)
uniform (0.01)
Pr(significance)
p−value threshold
n = 500 ; B_m = −0.3 ; B_x = −0.3 ; B_y = −0.5
CCPR (0.98)
UMPR (0.72)
UXPR (0.017)
AMPR (0.415)
AXPR (0.011)
UOV (0.009)
AOV (0.002)
uniform (0.01)
Pr(significance)
p−value threshold

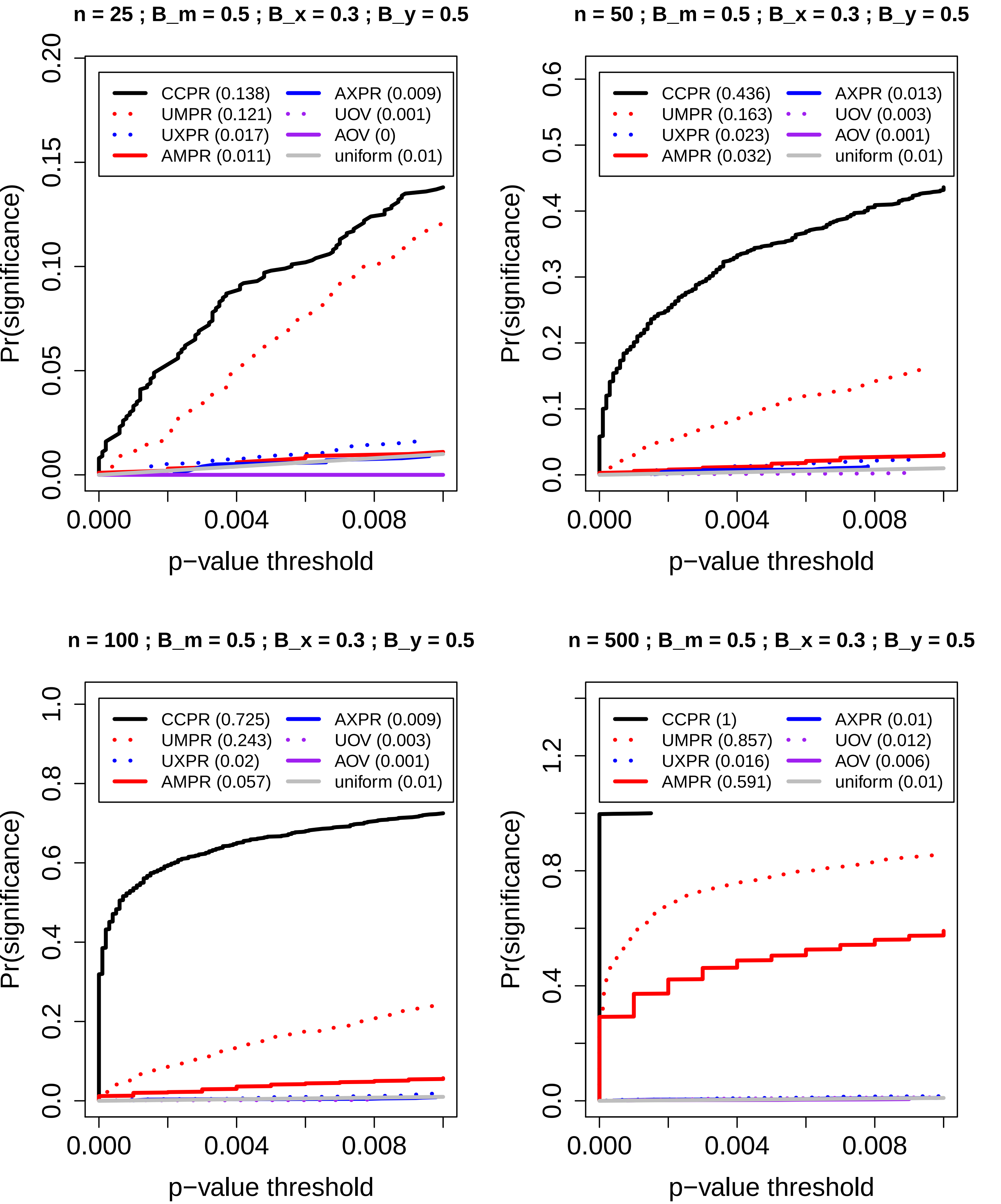

n = 25 ; B_m = 0.5 ; B_x = 0.3 ; B_y = 0.5
CCPR (0.138)
UMPR (0.121)
UXPR (0.017)
AMPR (0.011)
AXPR (0.009)
UOV (0.001)
AOV (0)
uniform (0.01)
Pr(significance)
p−value threshold
n = 50 ; B_m = 0.5 ; B_x = 0.3 ; B_y = 0.5
CCPR (0.436)
UMPR (0.163)
UXPR (0.023)
AMPR (0.032)
AXPR (0.013)
UOV (0.003)
AOV (0.001)
uniform (0.01)
Pr(significance)
p−value threshold
n = 100 ; B_m = 0.5 ; B_x = 0.3 ; B_y = 0.5
CCPR (0.725)
UMPR (0.243)
UXPR (0.02)
AMPR (0.057)
AXPR (0.009)
UOV (0.003)
AOV (0.001)
uniform (0.01)
Pr(significance)
p−value threshold
n = 500 ; B_m = 0.5 ; B_x = 0.3 ; B_y = 0.5
CCPR (1)
UMPR (0.857)
UXPR (0.016)
AMPR (0.591)
AXPR (0.01)
UOV (0.012)
AOV (0.006)
uniform (0.01)
Pr(significance)
p−value threshold

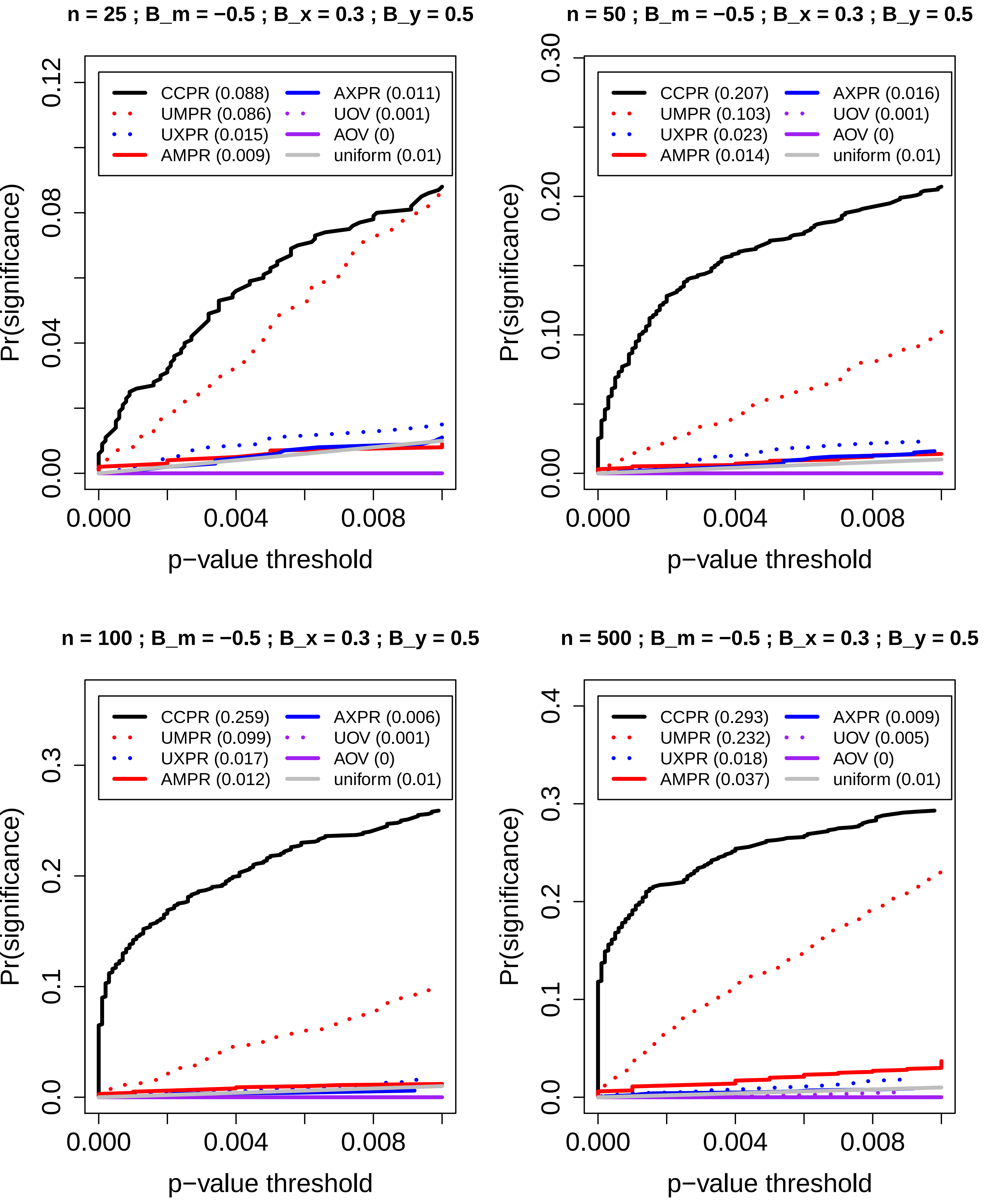

n = 25 ; B_m = −0.5 ; B_x = 0.3 ; B_y = 0.5
CCPR (0.088)
UMPR (0.086)
UXPR (0.015)
AMPR (0.009)
AXPR (0.011)
UOV (0.001)
AOV (0)
uniform (0.01)
Pr(significance)
p−value threshold
0.00
0.04
0.08
0.12
0.000
0.004
0.008
n = 50 ; B_m = −0.5 ; B_x = 0.3 ; B_y = 0.5
CCPR (0.207)
UMPR (0.103)
UXPR (0.023)
AMPR (0.014)
AXPR (0.016)
UOV (0.001)
AOV (0)
uniform (0.01)
Pr(significance)
p−value threshold
0.00
0.10
0.20
0.30
0.000
0.004
0.008
n = 100 ; B_m = −0.5 ; B_x = 0.3 ; B_y = 0.5
CCPR (0.259)
UMPR (0.099)
UXPR (0.017)
AMPR (0.012)
AXPR (0.006)
UOV (0.001)
AOV (0)
uniform (0.01)
Pr(significance)
p−value threshold
0.0
0.1
0.2
0.3
0.000
0.004
0.008
n = 500 ; B_m = −0.5 ; B_x = 0.3 ; B_y = 0.5
CCPR (0.293)
UMPR (0.232)
UXPR (0.018)
AMPR (0.037)
AXPR (0.009)
UOV (0.005)
AOV (0)
uniform (0.01)
Pr(significance)
p−value threshold
0.0
0.1
0.2
0.3
0.4
0.000
0.004
0.008

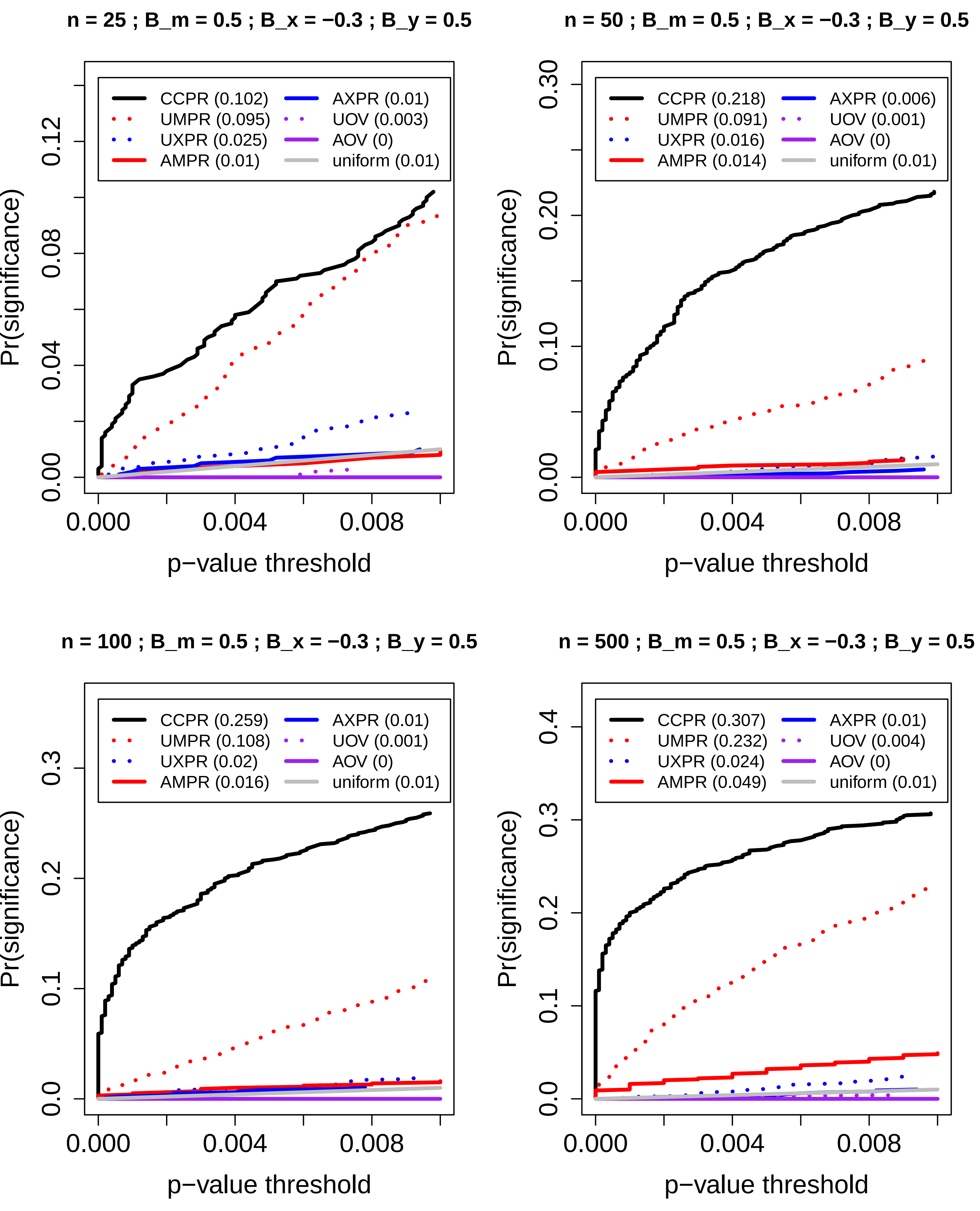

n = 25 ; B_m = 0.5 ; B_x = −0.3 ; B_y = 0.5
CCPR (0.102)
UMPR (0.095)
UXPR (0.025)
AMPR (0.01)
AXPR (0.01)
UOV (0.003)
AOV (0)
uniform (0.01)
Pr(significance)
p−value threshold
n = 50 ; B_m = 0.5 ; B_x = −0.3 ; B_y = 0.5
CCPR (0.218)
UMPR (0.091)
UXPR (0.016)
AMPR (0.014)
AXPR (0.006)
UOV (0.001)
AOV (0)
uniform (0.01)
Pr(significance)
p−value threshold
n = 100 ; B_m = 0.5 ; B_x = −0.3 ; B_y = 0.5
CCPR (0.259)
UMPR (0.108)
UXPR (0.02)
AMPR (0.016)
AXPR (0.01)
UOV (0.001)
AOV (0)
uniform (0.01)
Pr(significance)
p−value threshold
n = 500 ; B_m = 0.5 ; B_x = −0.3 ; B_y = 0.5
CCPR (0.307)
UMPR (0.232)
UXPR (0.024)
AMPR (0.049)
AXPR (0.01)
UOV (0.004)
AOV (0)
uniform (0.01)
Pr(significance)
p−value threshold

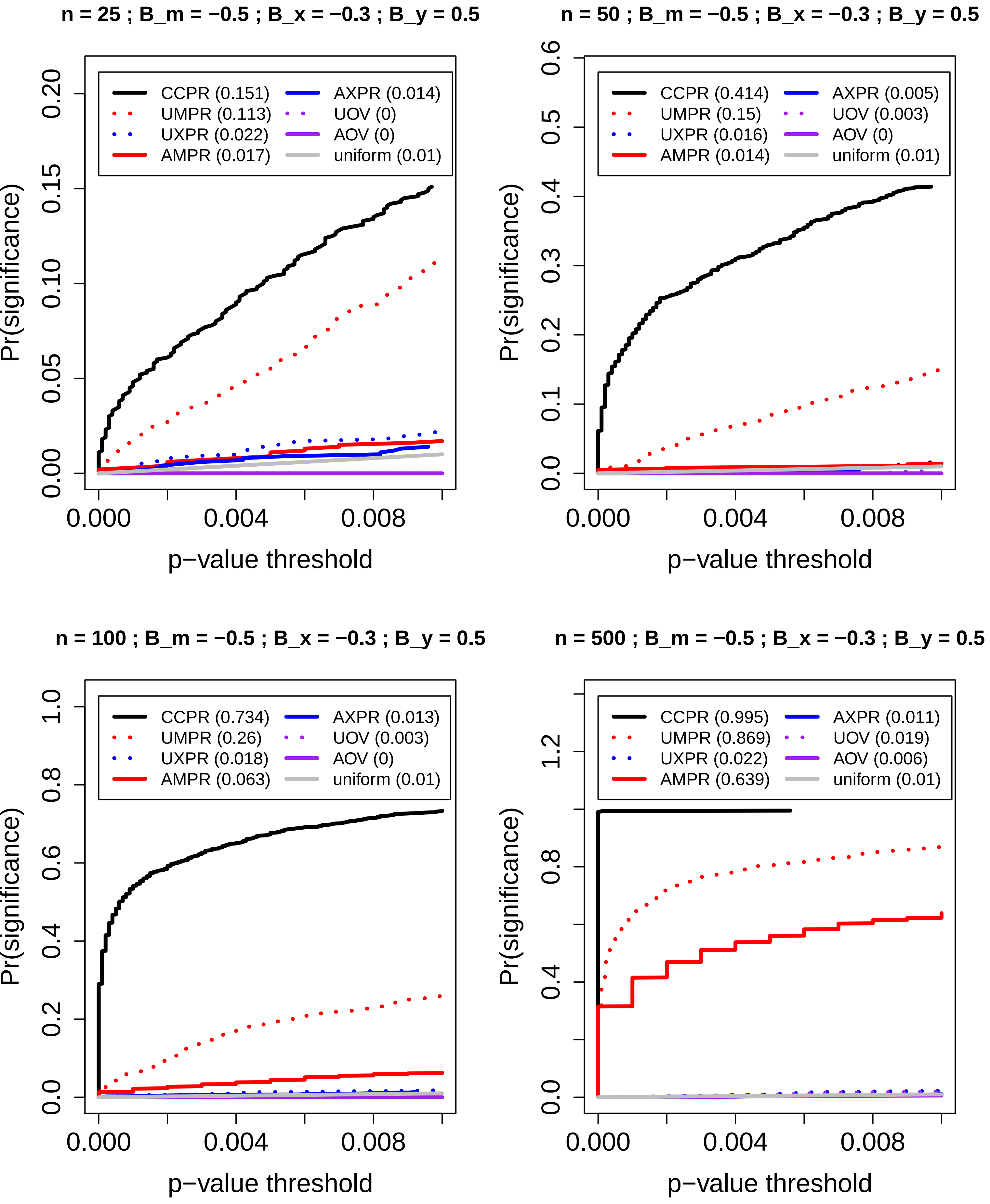

n = 25 ; B_m = −0.5 ; B_x = −0.3 ; B_y = 0.5
CCPR (0.151)
UMPR (0.113)
UXPR (0.022)
AMPR (0.017)
AXPR (0.014)
UOV (0)
AOV (0)
uniform (0.01)
n = 50 ; B_m = −0.5 ; B_x = −0.3 ; B_y = 0.5
CCPR (0.414)
UMPR (0.15)
UXPR (0.016)
AMPR (0.014)
AXPR (0.005)
UOV (0.003)
AOV (0)
uniform (0.01)
n = 100 ; B_m = −0.5 ; B_x = −0.3 ; B_y = 0.5
CCPR (0.734)
UMPR (0.26)
UXPR (0.018)
AMPR (0.063)
AXPR (0.013)
UOV (0.003)
AOV (0)
uniform (0.01)
n = 500 ; B_m = −0.5 ; B_x = −0.3 ; B_y = 0.5
CCPR (0.995)
UMPR (0.869)
UXPR (0.022)
AMPR (0.639)
AXPR (0.011)
UOV (0.019)
AOV (0.006)
uniform (0.01)
Pr(significance)
p−value threshold
0.000
0.004
0.008

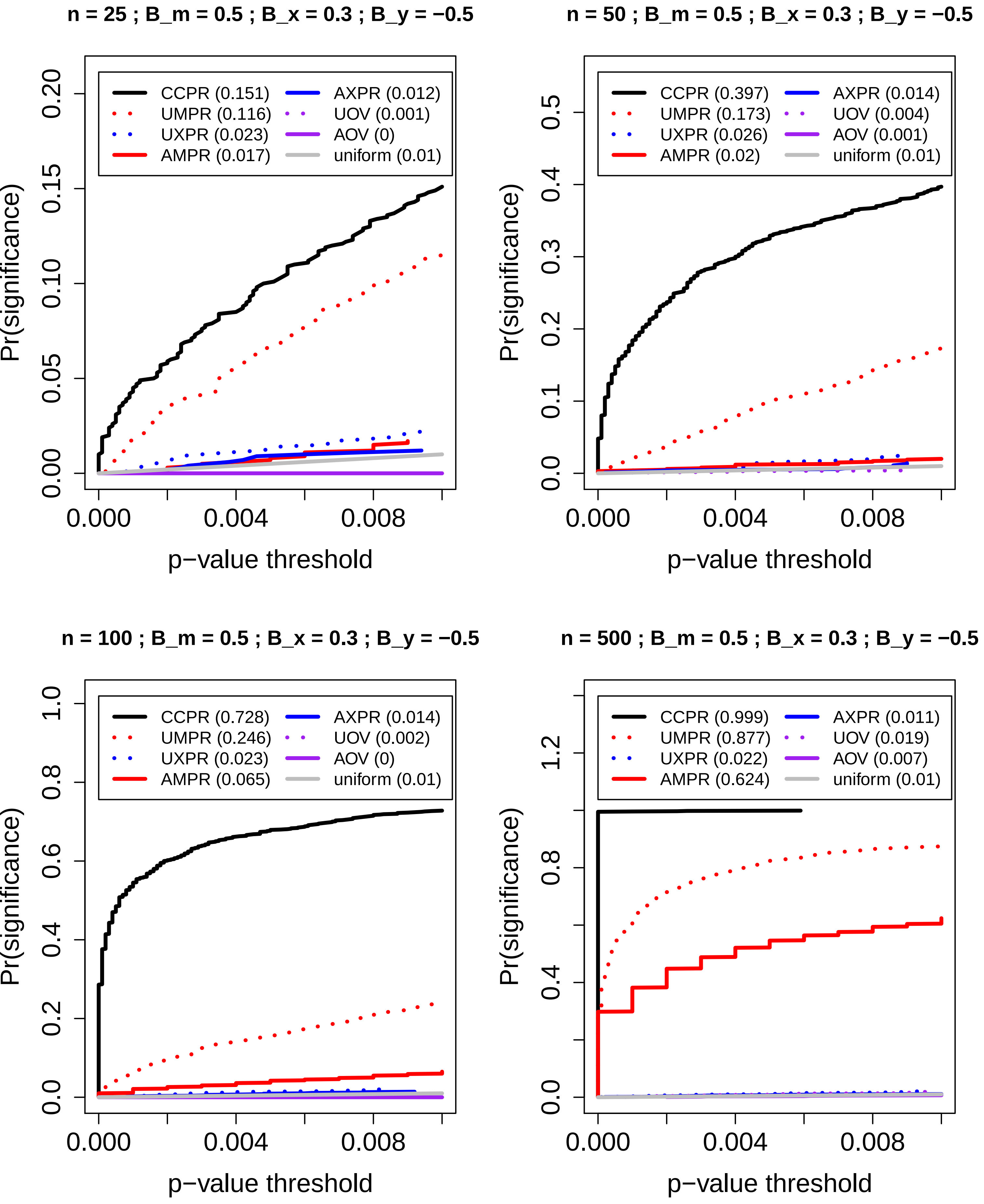

n = 25 ; B_m = 0.5 ; B_x = 0.3 ; B_y = −0.5
CCPR (0.151)
UMPR (0.116)
UXPR (0.023)
AMPR (0.017)
AXPR (0.012)
UOV (0.001)
AOV (0)
uniform (0.01)
n = 50 ; B_m = 0.5 ; B_x = 0.3 ; B_y = −0.5
CCPR (0.397)
UMPR (0.173)
UXPR (0.026)
AMPR (0.02)
AXPR (0.014)
UOV (0.004)
AOV (0.001)
uniform (0.01)
n = 100 ; B_m = 0.5 ; B_x = 0.3 ; B_y = −0.5
CCPR (0.728)
UMPR (0.246)
UXPR (0.023)
AMPR (0.065)
AXPR (0.014)
UOV (0.002)
AOV (0)
uniform (0.01)
n = 500 ; B_m = 0.5 ; B_x = 0.3 ; B_y = −0.5
CCPR (0.999)
UMPR (0.877)
UXPR (0.022)
AMPR (0.624)
AXPR (0.011)
UOV (0.019)
AOV (0.007)
uniform (0.01)
Pr(significance)
p−value threshold

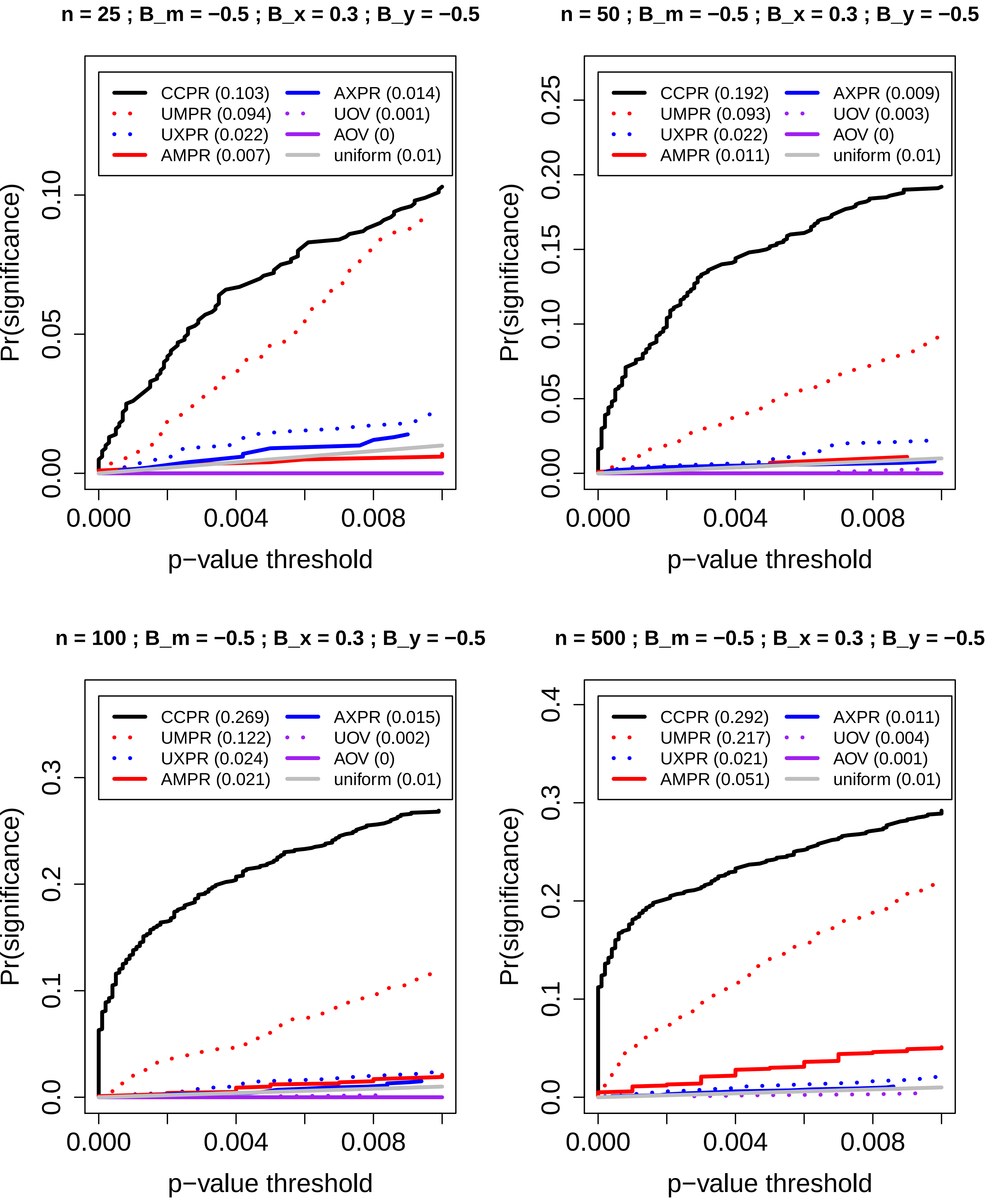

n = 25 ; B_m = −0.5 ; B_x = 0.3 ; B_y = −0.5
CCPR (0.103)
UMPR (0.094)
UXPR (0.022)
AMPR (0.007)
AXPR (0.014)
UOV (0.001)
AOV (0)
uniform (0.01)
Pr(significance)
0.00
0.05
0.10
0.000
0.004
0.008
p−value threshold
n = 50 ; B_m = −0.5 ; B_x = 0.3 ; B_y = −0.5
CCPR (0.192)
UMPR (0.093)
UXPR (0.022)
AMPR (0.011)
AXPR (0.009)
UOV (0.003)
AOV (0)
uniform (0.01)
Pr(significance)
0.00
0.05
0.10
0.15
0.20
0.25
0.000
0.004
0.008
p−value threshold
n = 100 ; B_m = −0.5 ; B_x = 0.3 ; B_y = −0.5
CCPR (0.269)
UMPR (0.122)
UXPR (0.024)
AMPR (0.021)
AXPR (0.015)
UOV (0.002)
AOV (0)
uniform (0.01)
Pr(significance)
0.0
0.1
0.2
0.3
0.000
0.004
0.008
p−value threshold
n = 500 ; B_m = −0.5 ; B_x = 0.3 ; B_y = −0.5
CCPR (0.292)
UMPR (0.217)
UXPR (0.021)
AMPR (0.051)
AXPR (0.011)
UOV (0.004)
AOV (0.001)
uniform (0.01)
Pr(significance)
0.0
0.1
0.2
0.3
0.4
0.000
0.004
0.008
p−value threshold

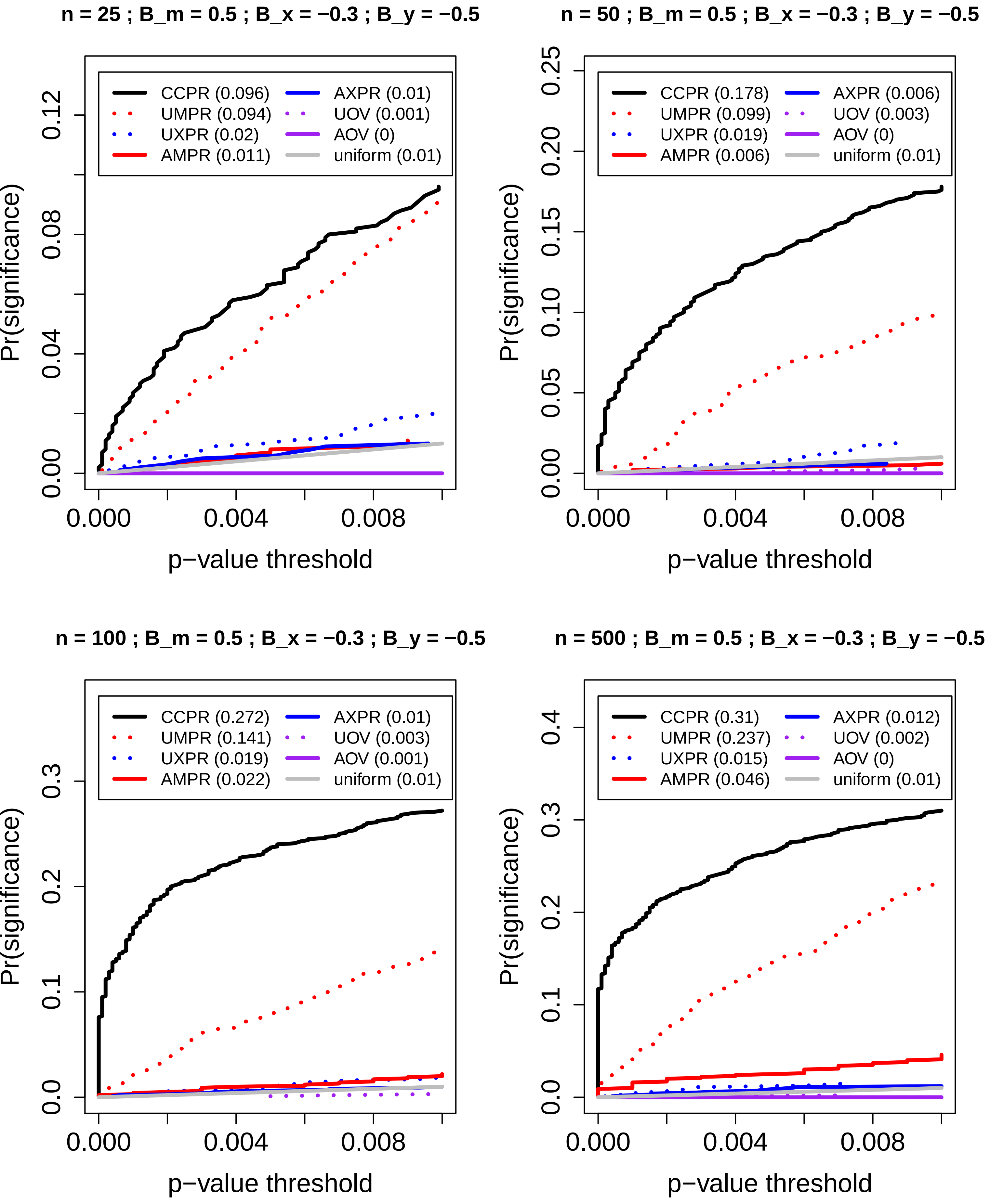
n = 25 ; B_m = 0.5 ; B_x = −0.3 ; B_y = −0.5
CCPR (0.096)
UMPR (0.094)
UXPR (0.02)
AMPR (0.011)
AXPR (0.01)
UOV (0.001)
AOV (0)
uniform (0.01)
Pr(significance)
p−value threshold
n = 50 ; B_m = 0.5 ; B_x = −0.3 ; B_y = −0.5
CCPR (0.178)
UMPR (0.099)
UXPR (0.019)
AMPR (0.006)
AXPR (0.006)
UOV (0.003)
AOV (0)
uniform (0.01)
Pr(significance)
p−value threshold
n = 100 ; B_m = 0.5 ; B_x = −0.3 ; B_y = −0.5
CCPR (0.272)
UMPR (0.141)
UXPR (0.019)
AMPR (0.022)
AXPR (0.01)
UOV (0.003)
AOV (0.001)
uniform (0.01)
Pr(significance)
p−value threshold
n = 500 ; B_m = 0.5 ; B_x = −0.3 ; B_y = −0.5
CCPR (0.31)
UMPR (0.237)
UXPR (0.015)
AMPR (0.046)
AXPR (0.012)
UOV (0.002)
AOV (0)
uniform (0.01)
Pr(significance)
p−value threshold

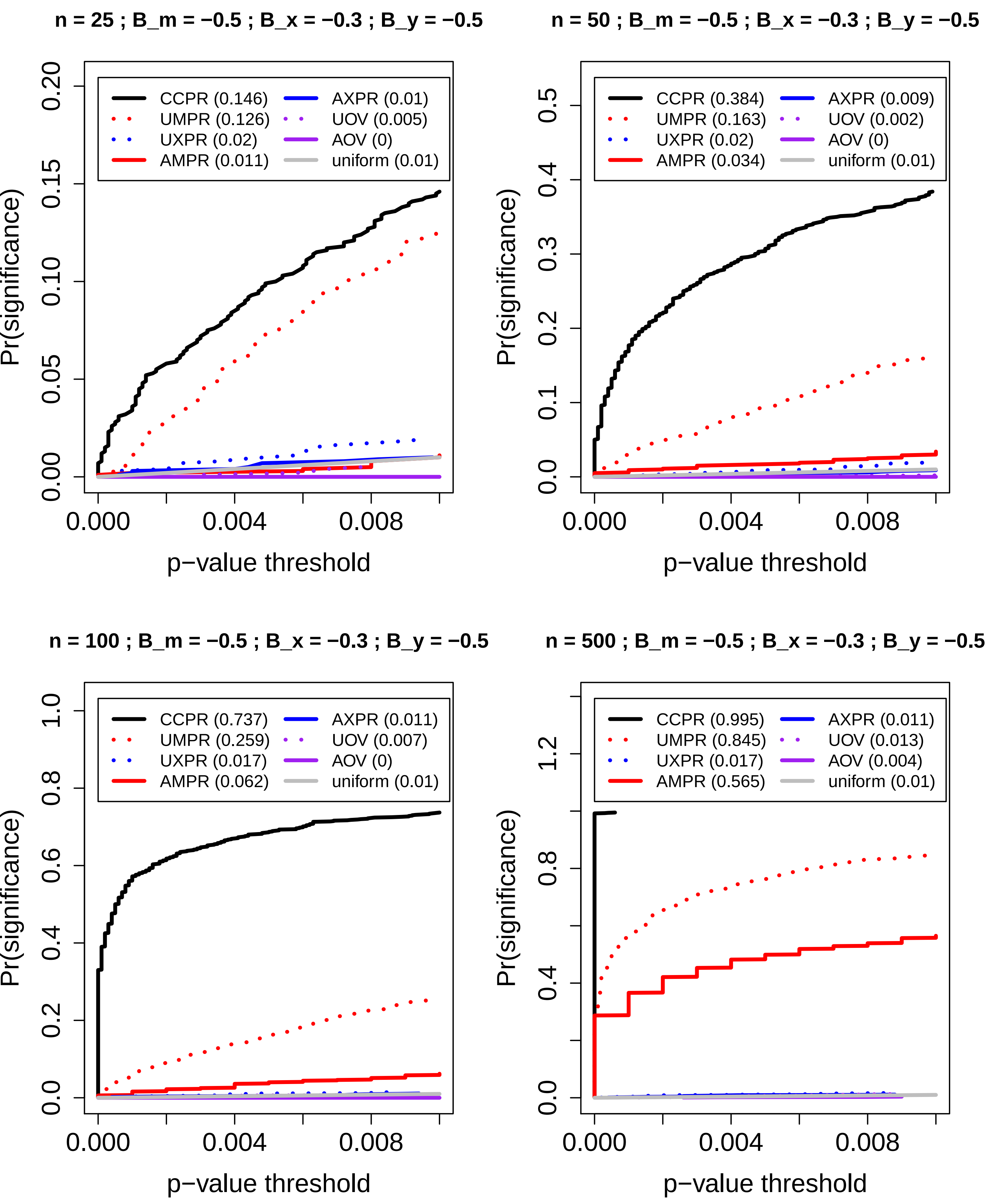
n = 25 ; B_m = −0.5 ; B_x = −0.3 ; B_y = −0.5
CCPR (0.146)
UMPR (0.126)
UXPR (0.02)
AMPR (0.011)
AXPR (0.01)
UOV (0.005)
AOV (0)
uniform (0.01)
Pr(significance)
p−value threshold
n = 50 ; B_m = −0.5 ; B_x = −0.3 ; B_y = −0.5
CCPR (0.384)
UMPR (0.163)
UXPR (0.02)
AMPR (0.034)
AXPR (0.009)
UOV (0.002)
AOV (0)
uniform (0.01)
Pr(significance)
p−value threshold
n = 100 ; B_m = −0.5 ; B_x = −0.3 ; B_y = −0.5
CCPR (0.737)
UMPR (0.259)
UXPR (0.017)
AMPR (0.062)
AXPR (0.011)
UOV (0.007)
AOV (0)
uniform (0.01)
Pr(significance)
p−value threshold
n = 500 ; B_m = −0.5 ; B_x = −0.3 ; B_y = −0.5
CCPR (0.995)
UMPR (0.845)
UXPR (0.017)
AMPR (0.565)
AXPR (0.011)
UOV (0.013)
AOV (0.004)
uniform (0.01)
Pr(significance)
p−value threshold

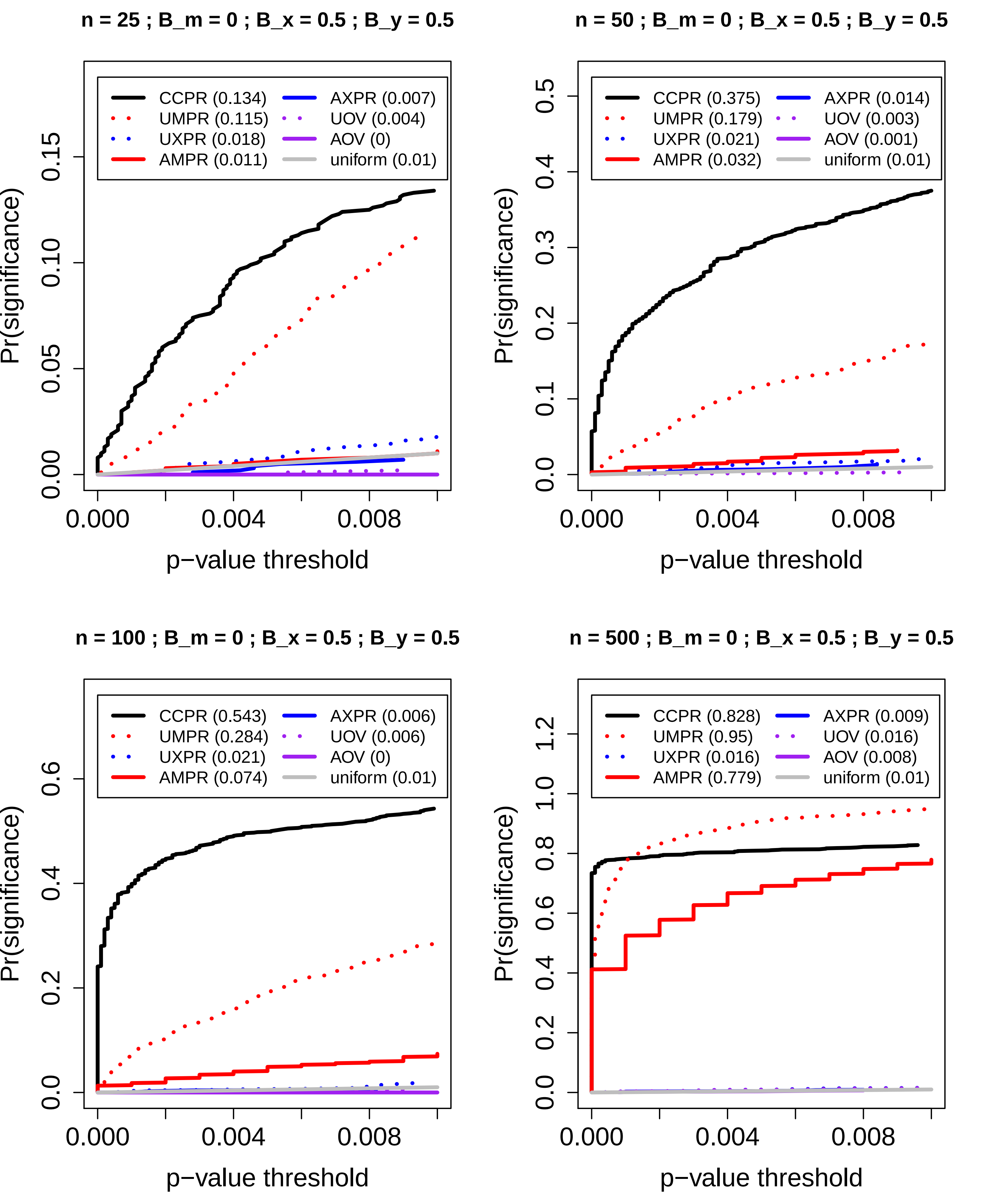

n = 25 ; B_m = 0 ; B_x = 0.5 ; B_y = 0.5
CCPR (0.134)
UMPR (0.115)
UXPR (0.018)
AMPR (0.011)
AXPR (0.007)
UOV (0.004)
AOV (0)
uniform (0.01)
Pr(significance)
p−value threshold
n = 50 ; B_m = 0 ; B_x = 0.5 ; B_y = 0.5
CCPR (0.375)
UMPR (0.179)
UXPR (0.021)
AMPR (0.032)
AXPR (0.014)
UOV (0.003)
AOV (0.001)
uniform (0.01)
Pr(significance)
p−value threshold
n = 100 ; B_m = 0 ; B_x = 0.5 ; B_y = 0.5
CCPR (0.543)
UMPR (0.284)
UXPR (0.021)
AMPR (0.074)
AXPR (0.006)
UOV (0.006)
AOV (0)
uniform (0.01)
Pr(significance)
p−value threshold
n = 500 ; B_m = 0 ; B_x = 0.5 ; B_y = 0.5
CCPR (0.828)
UMPR (0.95)
UXPR (0.016)
AMPR (0.779)
AXPR (0.009)
UOV (0.016)
AOV (0.008)
uniform (0.01)
Pr(significance)
p−value threshold

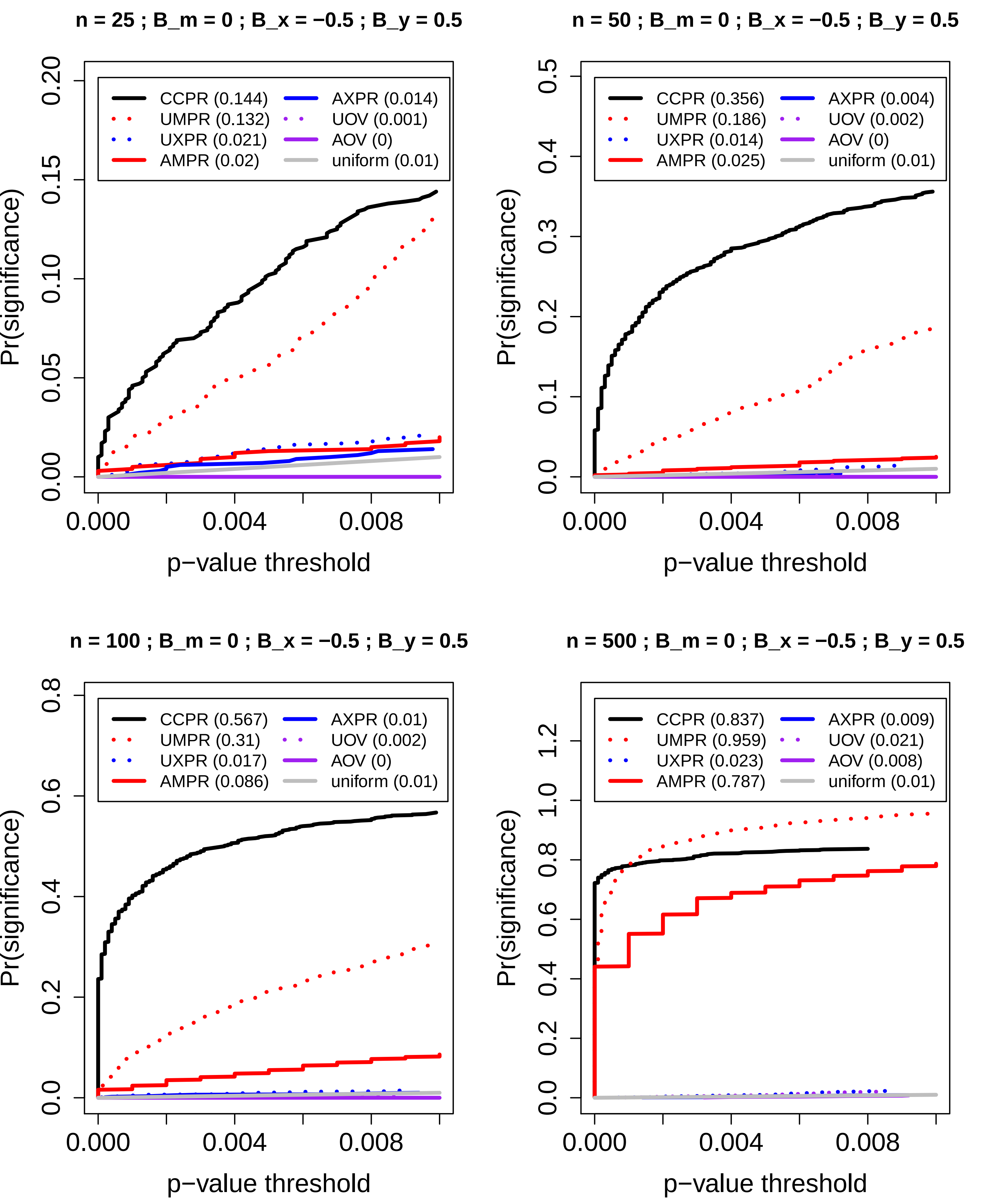
n = 25 ; B_m = 0 ; B_x = −0.5 ; B_y = 0.5
CCPR (0.144)
UMPR (0.132)
UXPR (0.021)
AMPR (0.02)
AXPR (0.014)
UOV (0.001)
AOV (0)
uniform (0.01)
n = 50 ; B_m = 0 ; B_x = −0.5 ; B_y = 0.5
CCPR (0.356)
UMPR (0.186)
UXPR (0.014)
AMPR (0.025)
AXPR (0.004)
UOV (0.002)
AOV (0)
uniform (0.01)
n = 100 ; B_m = 0 ; B_x = −0.5 ; B_y = 0.5
CCPR (0.567)
UMPR (0.31)
UXPR (0.017)
AMPR (0.086)
AXPR (0.01)
UOV (0.002)
AOV (0)
uniform (0.01)
n = 500 ; B_m = 0 ; B_x = −0.5 ; B_y = 0.5
CCPR (0.837)
UMPR (0.959)
UXPR (0.023)
AMPR (0.787)
AXPR (0.009)
UOV (0.021)
AOV (0.008)
uniform (0.01)
Pr(significance)
p−value threshold

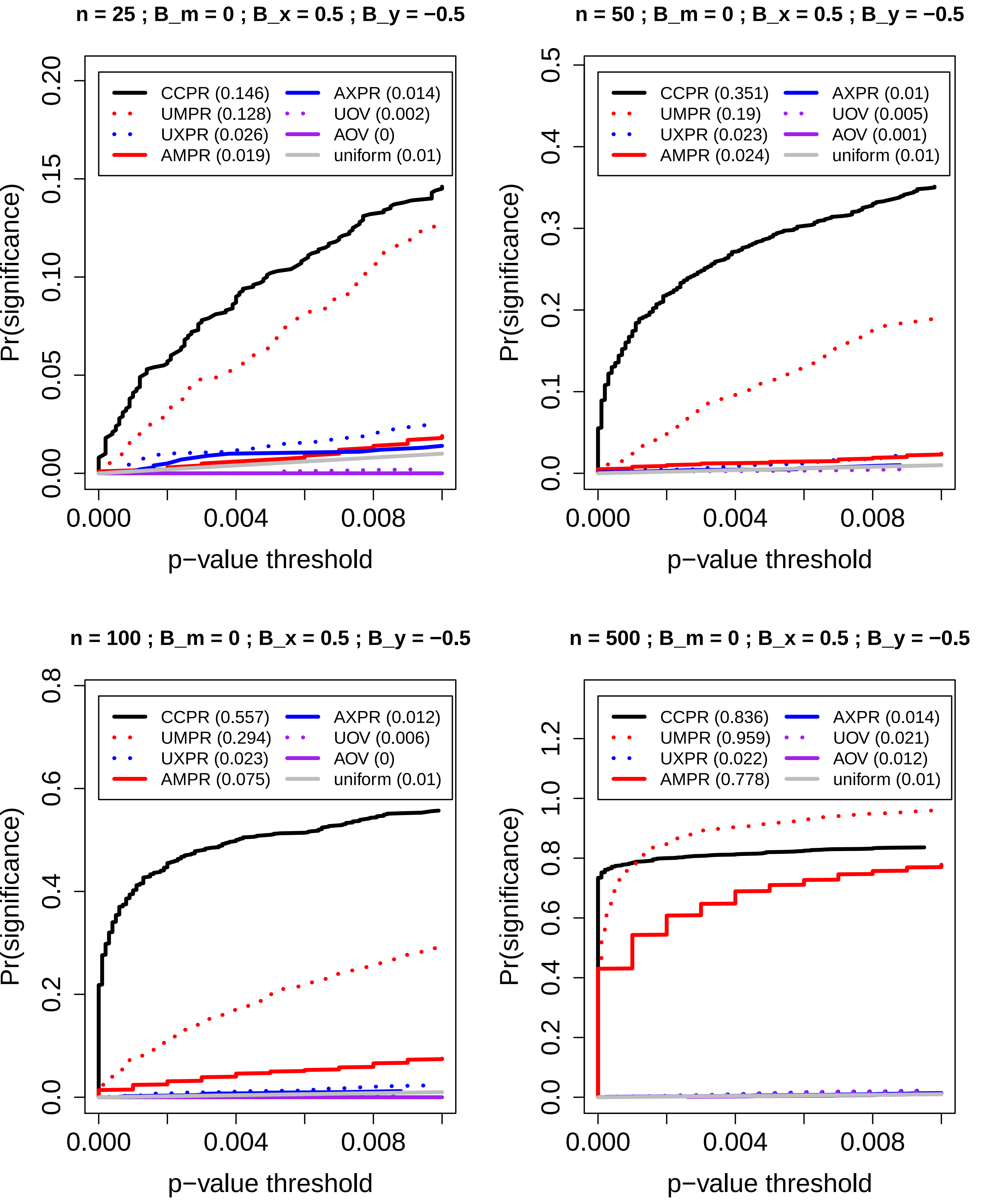

n = 25 ; B_m = 0 ; B_x = 0.5 ; B_y = −0.5
CCPR (0.146)
UMPR (0.128)
UXPR (0.026)
AMPR (0.019)
AXPR (0.014)
UOV (0.002)
AOV (0)
uniform (0.01)
Pr(significance)
0.00
0.05
0.10
0.15
0.20
0.000
0.004
0.008
p−value threshold
n = 50 ; B_m = 0 ; B_x = 0.5 ; B_y = −0.5
CCPR (0.351)
UMPR (0.19)
UXPR (0.023)
AMPR (0.024)
AXPR (0.01)
UOV (0.005)
AOV (0.001)
uniform (0.01)
Pr(significance)
0.0
0.1
0.2
0.3
0.4
0.5
0.000
0.004
0.008
p−value threshold
n = 100 ; B_m = 0 ; B_x = 0.5 ; B_y = −0.5
CCPR (0.557)
UMPR (0.294)
UXPR (0.023)
AMPR (0.075)
AXPR (0.012)
UOV (0.006)
AOV (0)
uniform (0.01)
Pr(significance)
0.0
0.2
0.4
0.6
0.8
0.000
0.004
0.008
p−value threshold
n = 500 ; B_m = 0 ; B_x = 0.5 ; B_y = −0.5
CCPR (0.836)
UMPR (0.959)
UXPR (0.022)
AMPR (0.778)
AXPR (0.014)
UOV (0.021)
AOV (0.012)
uniform (0.01)
Pr(significance)
0.0
0.2
0.4
0.6
0.8
1.0
1.2
0.000
0.004
0.008
p−value threshold

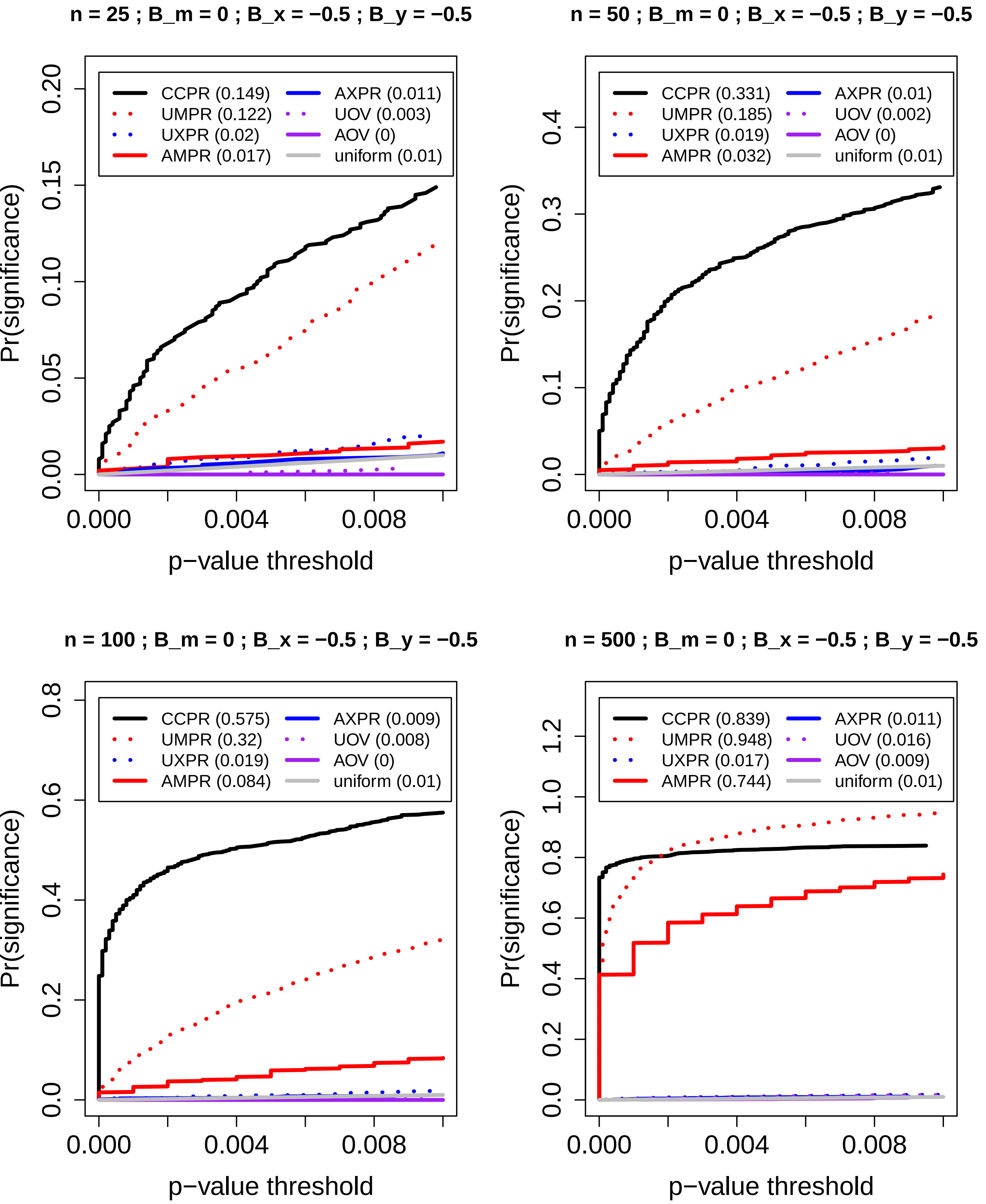

n = 25 ; B_m = 0 ; B_x = −0.5 ; B_y = −0.5
CCPR (0.149)
UMPR (0.122)
UXPR (0.02)
AMPR (0.017)
AXPR (0.011)
UOV (0.003)
AOV (0)
uniform (0.01)
Pr(significance)
p−value threshold
n = 50 ; B_m = 0 ; B_x = −0.5 ; B_y = −0.5
CCPR (0.331)
UMPR (0.185)
UXPR (0.019)
AMPR (0.032)
AXPR (0.01)
UOV (0.002)
AOV (0)
uniform (0.01)
Pr(significance)
p−value threshold
n = 100 ; B_m = 0 ; B_x = −0.5 ; B_y = −0.5
CCPR (0.575)
UMPR (0.32)
UXPR (0.019)
AMPR (0.084)
AXPR (0.009)
UOV (0.008)
AOV (0)
uniform (0.01)
Pr(significance)
p−value threshold
n = 500 ; B_m = 0 ; B_x = −0.5 ; B_y = −0.5
CCPR (0.839)
UMPR (0.948)
UXPR (0.017)
AMPR (0.744)
AXPR (0.011)
UOV (0.016)
AOV (0.009)
uniform (0.01)
Pr(significance)
p−value threshold

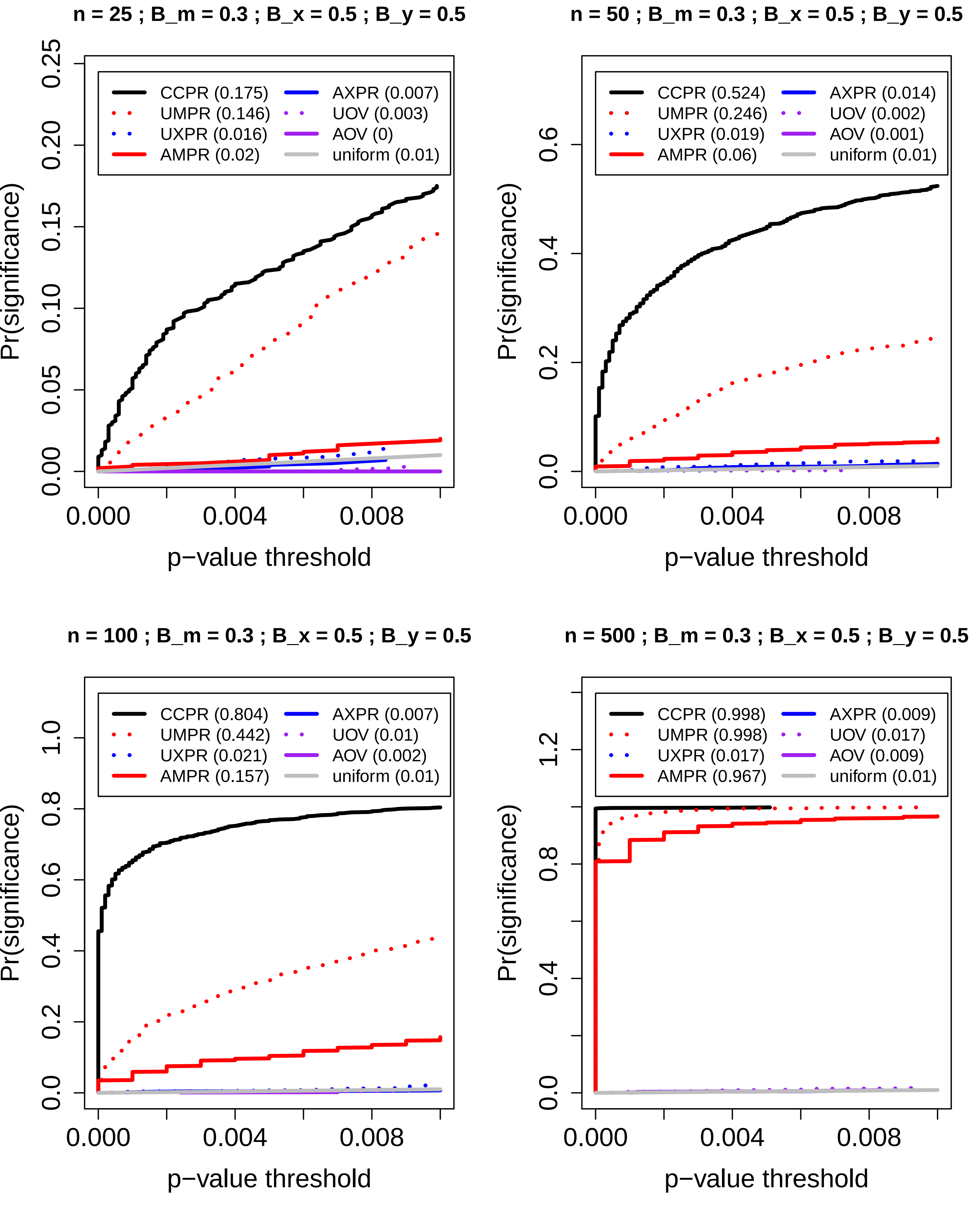
n = 25 ; B_m = 0.3 ; B_x = 0.5 ; B_y = 0.5
CCPR (0.175)
UMPR (0.146)
UXPR (0.016)
AMPR (0.02)
AXPR (0.007)
UOV (0.003)
AOV (0)
uniform (0.01)
Pr(significance)
0.00
0.05
0.10
0.15
0.20
0.25
0.000
0.004
0.008
p−value threshold
n = 50 ; B_m = 0.3 ; B_x = 0.5 ; B_y = 0.5
CCPR (0.524)
UMPR (0.246)
UXPR (0.019)
AMPR (0.06)
AXPR (0.014)
UOV (0.002)
AOV (0.001)
uniform (0.01)
Pr(significance)
0.0
0.2
0.4
0.6
0.000
0.004
0.008
p−value threshold
n = 100 ; B_m = 0.3 ; B_x = 0.5 ; B_y = 0.5
CCPR (0.804)
UMPR (0.442)
UXPR (0.021)
AMPR (0.157)
AXPR (0.007)
UOV (0.01)
AOV (0.002)
uniform (0.01)
Pr(significance)
0.0
0.2
0.4
0.6
0.8
1.0
0.000
0.004
0.008
p−value threshold
n = 500 ; B_m = 0.3 ; B_x = 0.5 ; B_y = 0.5
CCPR (0.998)
UMPR (0.998)
UXPR (0.017)
AMPR (0.967)
AXPR (0.009)
UOV (0.017)
AOV (0.009)
uniform (0.01)
Pr(significance)
0.0
0.4
0.8
1.2
0.000
0.004
0.008
p−value threshold

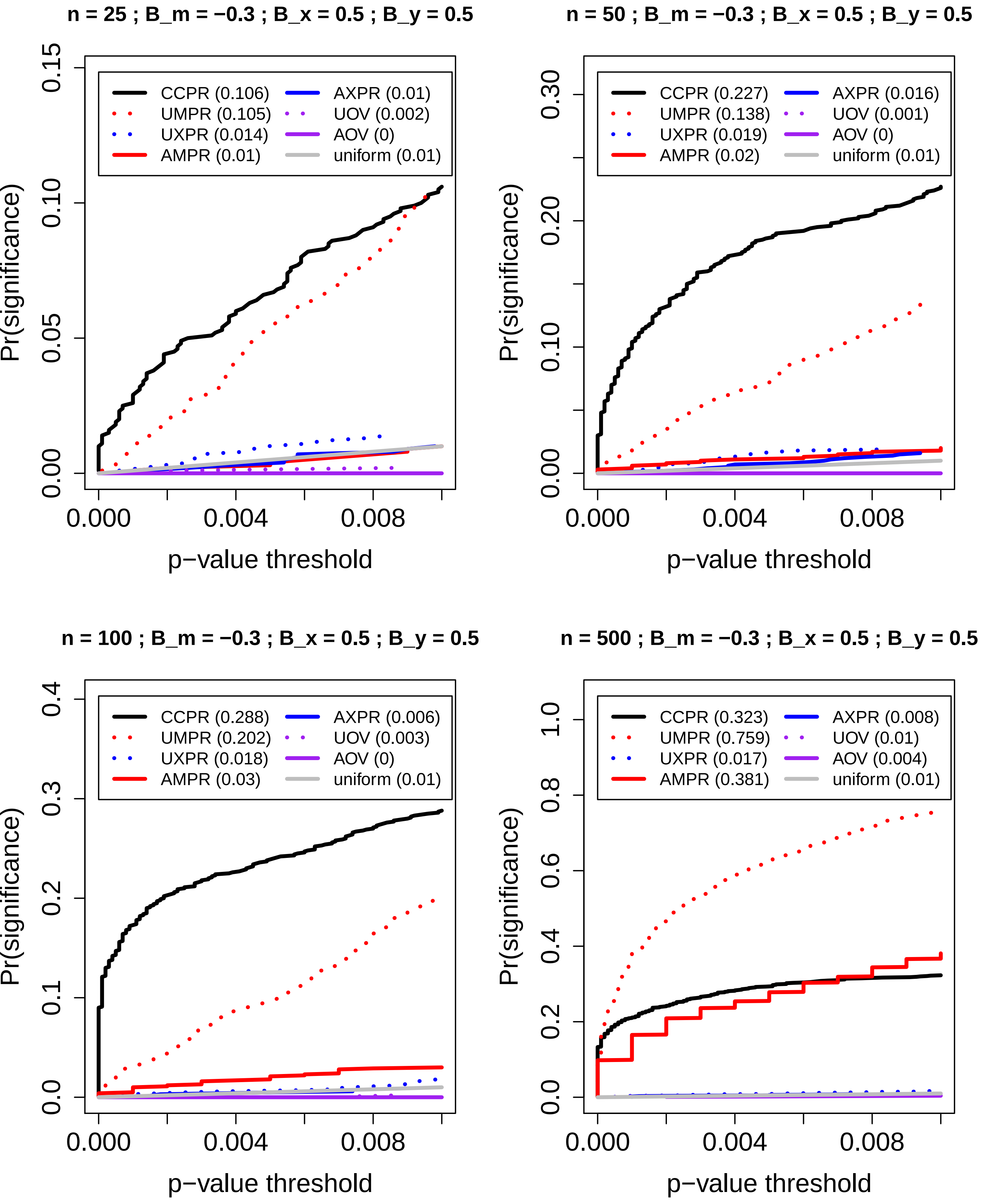

n = 25 ; B_m = −0.3 ; B_x = 0.5 ; B_y = 0.5
CCPR (0.106)
UMPR (0.105)
UXPR (0.014)
AMPR (0.01)
AXPR (0.01)
UOV (0.002)
AOV (0)
uniform (0.01)
Pr(significance)
0.00
0.05
0.10
0.15
0.000
0.004
0.008
p−value threshold
n = 50 ; B_m = −0.3 ; B_x = 0.5 ; B_y = 0.5
CCPR (0.227)
UMPR (0.138)
UXPR (0.019)
AMPR (0.02)
AXPR (0.016)
UOV (0.001)
AOV (0)
uniform (0.01)
Pr(significance)
0.00
0.10
0.20
0.30
0.000
0.004
0.008
p−value threshold
n = 100 ; B_m = −0.3 ; B_x = 0.5 ; B_y = 0.5
CCPR (0.288)
UMPR (0.202)
UXPR (0.018)
AMPR (0.03)
AXPR (0.006)
UOV (0.003)
AOV (0)
uniform (0.01)
Pr(significance)
0.0
0.1
0.2
0.3
0.4
0.000
0.004
0.008
p−value threshold
n = 500 ; B_m = −0.3 ; B_x = 0.5 ; B_y = 0.5
CCPR (0.323)
UMPR (0.759)
UXPR (0.017)
AMPR (0.381)
AXPR (0.008)
UOV (0.01)
AOV (0.004)
uniform (0.01)
Pr(significance)
0.0
0.2
0.4
0.6
0.8
1.0
0.000
0.004
0.008
p−value threshold

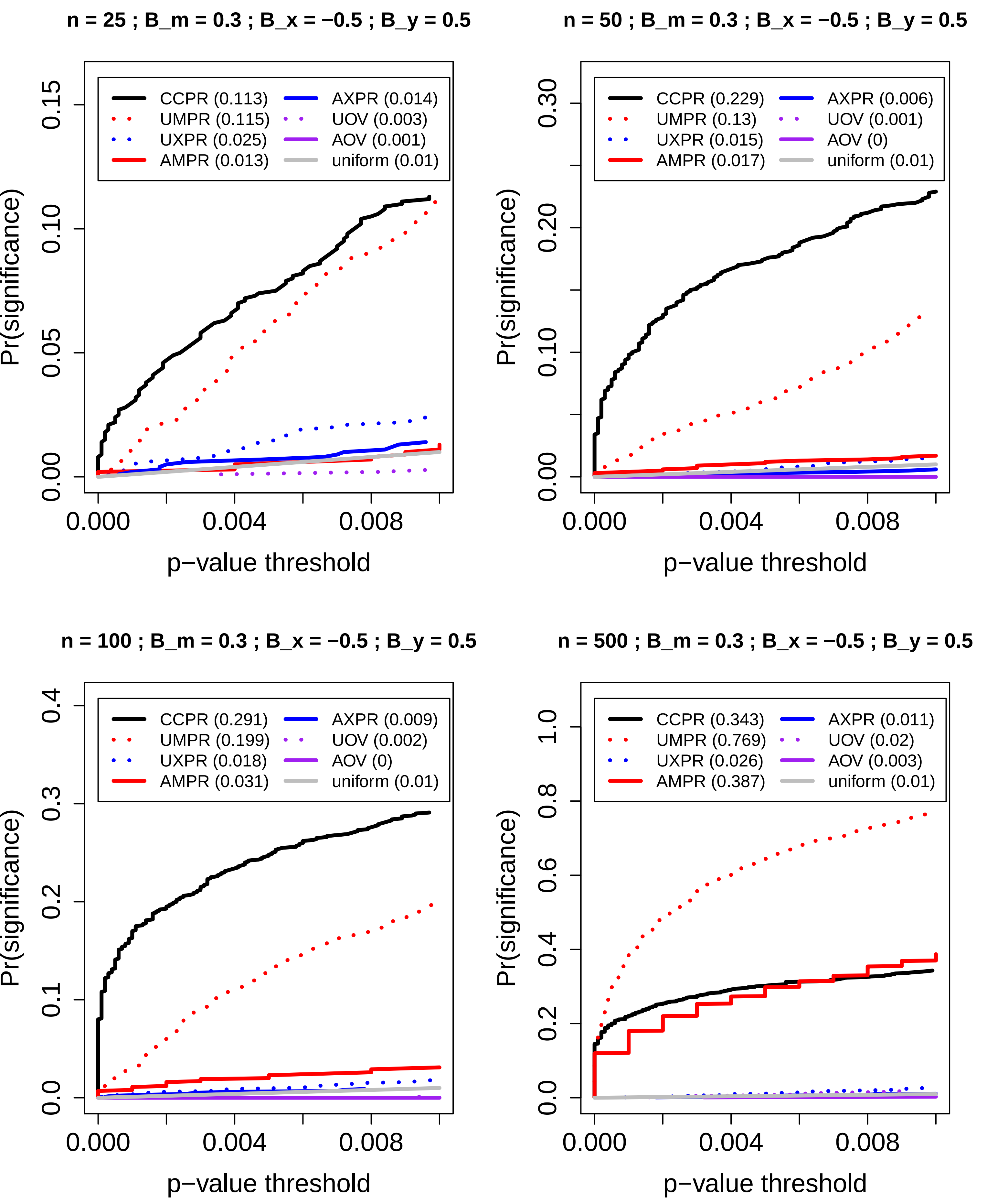

n = 25 ; B_m = 0.3 ; B_x = −0.5 ; B_y = 0.5
CCPR (0.113)
UMPR (0.115)
UXPR (0.025)
AMPR (0.013)
AXPR (0.014)
UOV (0.003)
AOV (0.001)
uniform (0.01)
Pr(significance)
p−value threshold
n = 50 ; B_m = 0.3 ; B_x = −0.5 ; B_y = 0.5
CCPR (0.229)
UMPR (0.13)
UXPR (0.015)
AMPR (0.017)
AXPR (0.006)
UOV (0.001)
AOV (0)
uniform (0.01)
Pr(significance)
p−value threshold
n = 100 ; B_m = 0.3 ; B_x = −0.5 ; B_y = 0.5
CCPR (0.291)
UMPR (0.199)
UXPR (0.018)
AMPR (0.031)
AXPR (0.009)
UOV (0.002)
AOV (0)
uniform (0.01)
Pr(significance)
p−value threshold
n = 500 ; B_m = 0.3 ; B_x = −0.5 ; B_y = 0.5
CCPR (0.343)
UMPR (0.769)
UXPR (0.026)
AMPR (0.387)
AXPR (0.011)
UOV (0.02)
AOV (0.003)
uniform (0.01)
Pr(significance)
p−value threshold

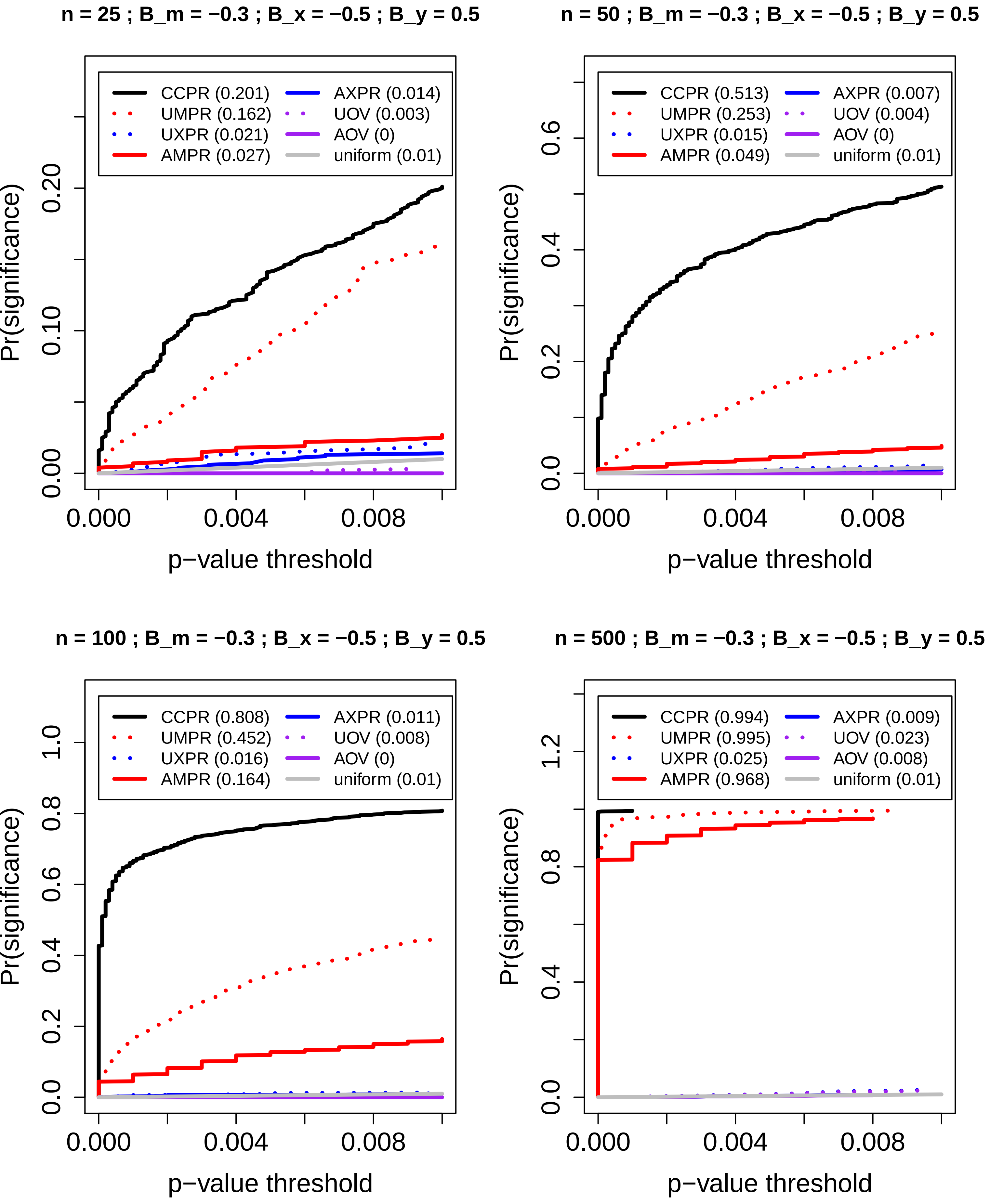
n = 25 ; B_m = −0.3 ; B_x = −0.5 ; B_y = 0.5
CCPR (0.201)
UMPR (0.162)
UXPR (0.021)
AMPR (0.027)
AXPR (0.014)
UOV (0.003)
AOV (0)
uniform (0.01)
Pr(significance)
0.00
0.10
0.20
0.000
0.004
0.008
p−value threshold
n = 50 ; B_m = −0.3 ; B_x = −0.5 ; B_y = 0.5
CCPR (0.513)
UMPR (0.253)
UXPR (0.015)
AMPR (0.049)
AXPR (0.007)
UOV (0.004)
AOV (0)
uniform (0.01)
Pr(significance)
0.0
0.2
0.4
0.6
0.000
0.004
0.008
p−value threshold
n = 100 ; B_m = −0.3 ; B_x = −0.5 ; B_y = 0.5
CCPR (0.808)
UMPR (0.452)
UXPR (0.016)
AMPR (0.164)
AXPR (0.011)
UOV (0.008)
AOV (0)
uniform (0.01)
Pr(significance)
0.0
0.2
0.4
0.6
0.8
1.0
0.000
0.004
0.008
p−value threshold
n = 500 ; B_m = −0.3 ; B_x = −0.5 ; B_y = 0.5
CCPR (0.994)
UMPR (0.995)
UXPR (0.025)
AMPR (0.968)
AXPR (0.009)
UOV (0.023)
AOV (0.008)
uniform (0.01)
Pr(significance)
0.0
0.4
0.8
1.2
0.000
0.004
0.008
p−value threshold

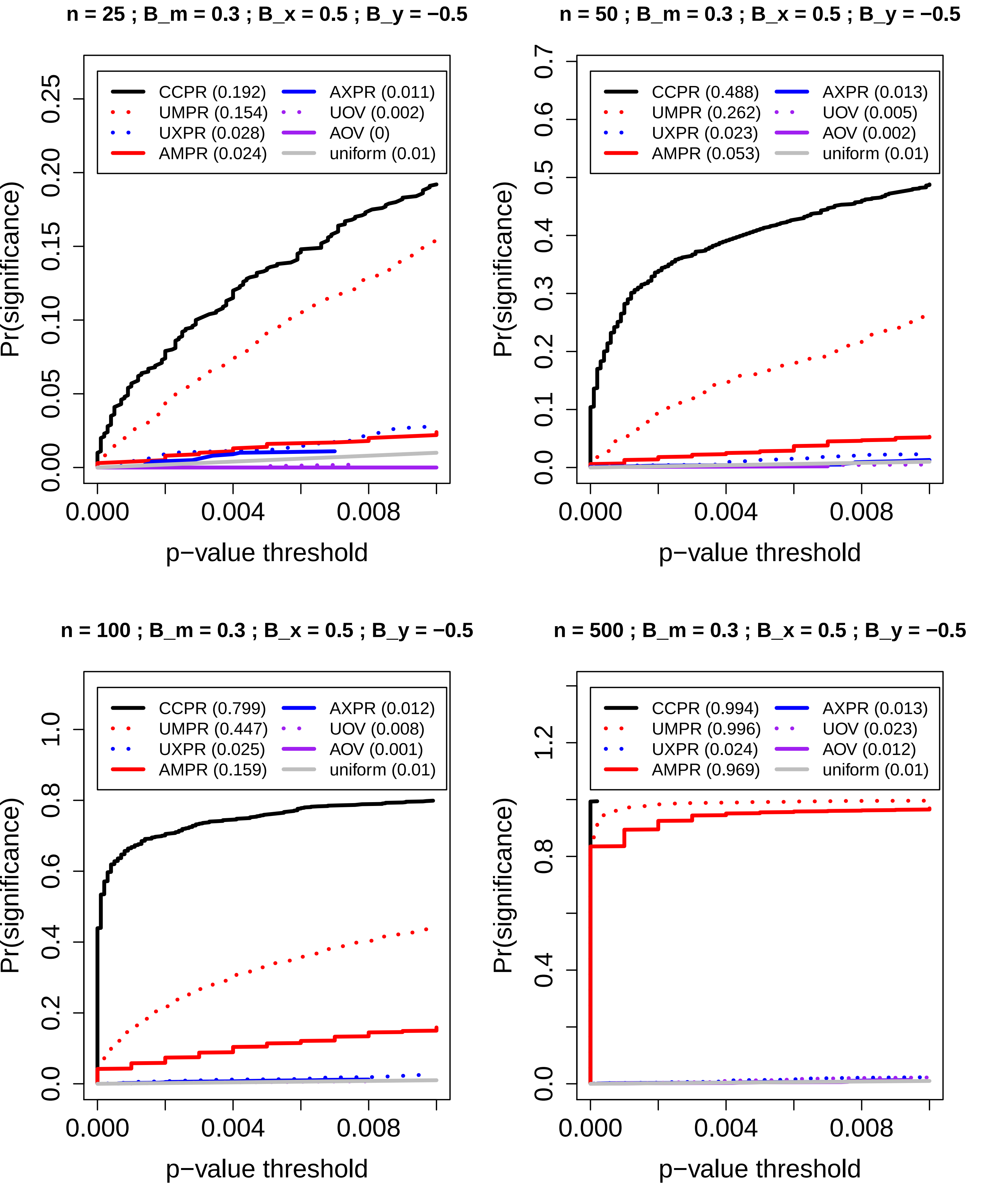

n = 25 ; B_m = 0.3 ; B_x = 0.5 ; B_y = −0.5
CCPR (0.192)
UMPR (0.154)
UXPR (0.028)
AMPR (0.024)
AXPR (0.011)
UOV (0.002)
AOV (0)
uniform (0.01)
Pr(significance)
p−value threshold
n = 50 ; B_m = 0.3 ; B_x = 0.5 ; B_y = −0.5
CCPR (0.488)
UMPR (0.262)
UXPR (0.023)
AMPR (0.053)
AXPR (0.013)
UOV (0.005)
AOV (0.002)
uniform (0.01)
Pr(significance)
p−value threshold
n = 100 ; B_m = 0.3 ; B_x = 0.5 ; B_y = −0.5
CCPR (0.799)
UMPR (0.447)
UXPR (0.025)
AMPR (0.159)
AXPR (0.012)
UOV (0.008)
AOV (0.001)
uniform (0.01)
Pr(significance)
p−value threshold
n = 500 ; B_m = 0.3 ; B_x = 0.5 ; B_y = −0.5
CCPR (0.994)
UMPR (0.996)
UXPR (0.024)
AMPR (0.969)
AXPR (0.013)
UOV (0.023)
AOV (0.012)
uniform (0.01)
Pr(significance)
p−value threshold

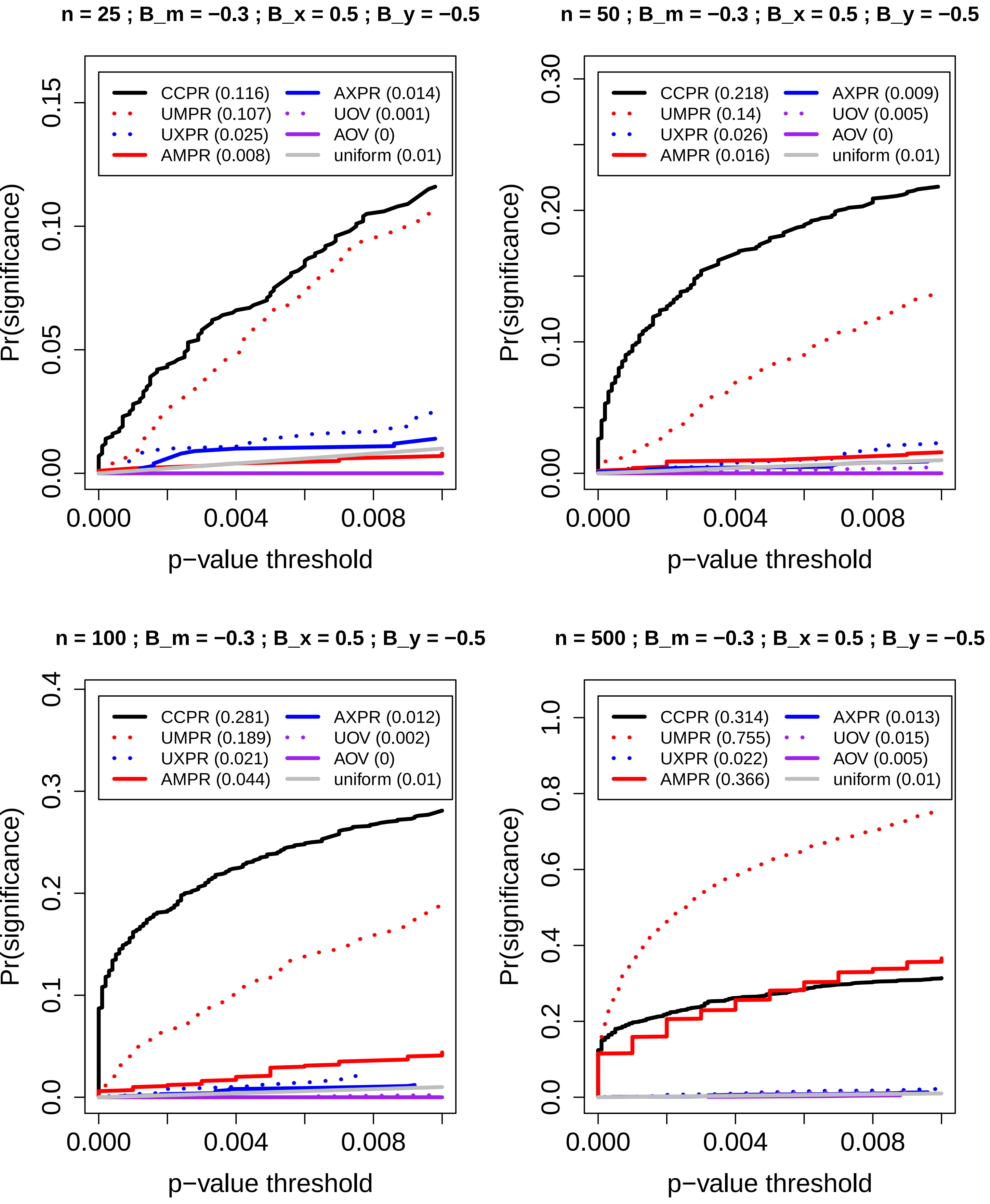

n = 25 ; B_m = −0.3 ; B_x = 0.5 ; B_y = −0.5
CCPR (0.116)
UMPR (0.107)
UXPR (0.025)
AMPR (0.008)
AXPR (0.014)
UOV (0.001)
AOV (0)
uniform (0.01)
Pr(significance)
p−value threshold
n = 50 ; B_m = −0.3 ; B_x = 0.5 ; B_y = −0.5
CCPR (0.218)
UMPR (0.14)
UXPR (0.026)
AMPR (0.016)
AXPR (0.009)
UOV (0.005)
AOV (0)
uniform (0.01)
Pr(significance)
p−value threshold
n = 100 ; B_m = −0.3 ; B_x = 0.5 ; B_y = −0.5
CCPR (0.281)
UMPR (0.189)
UXPR (0.021)
AMPR (0.044)
AXPR (0.012)
UOV (0.002)
AOV (0)
uniform (0.01)
Pr(significance)
p−value threshold
n = 500 ; B_m = −0.3 ; B_x = 0.5 ; B_y = −0.5
CCPR (0.314)
UMPR (0.755)
UXPR (0.022)
AMPR (0.366)
AXPR (0.013)
UOV (0.015)
AOV (0.005)
uniform (0.01)
Pr(significance)
p−value threshold

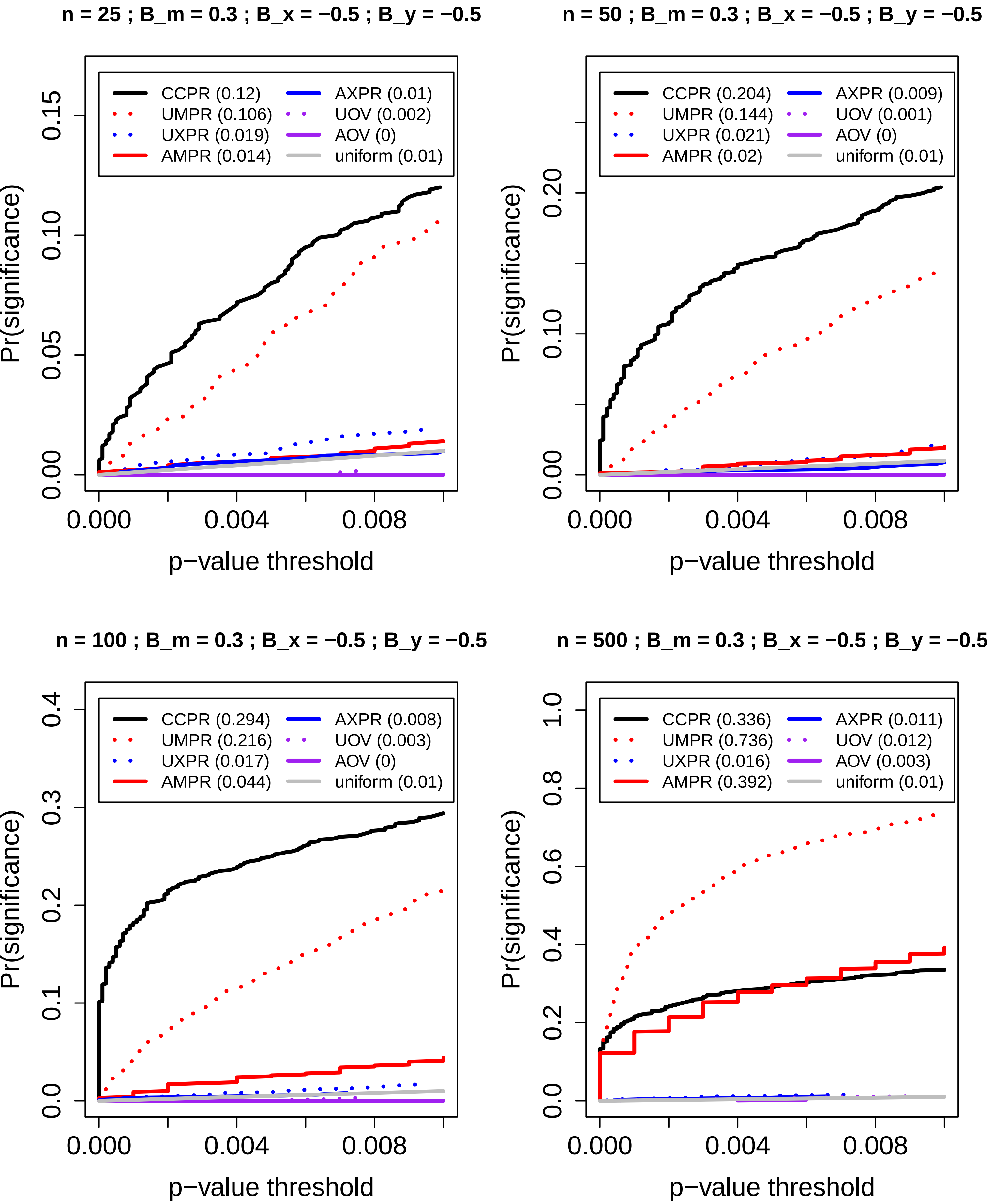

n = 25 ; B_m = 0.3 ; B_x = −0.5 ; B_y = −0.5
CCPR (0.12)
UMPR (0.106)
UXPR (0.019)
AMPR (0.014)
AXPR (0.01)
UOV (0.002)
AOV (0)
uniform (0.01)
n = 50 ; B_m = 0.3 ; B_x = −0.5 ; B_y = −0.5
CCPR (0.204)
UMPR (0.144)
UXPR (0.021)
AMPR (0.02)
AXPR (0.009)
UOV (0.001)
AOV (0)
uniform (0.01)
n = 100 ; B_m = 0.3 ; B_x = −0.5 ; B_y = −0.5
CCPR (0.294)
UMPR (0.216)
UXPR (0.017)
AMPR (0.044)
AXPR (0.008)
UOV (0.003)
AOV (0)
uniform (0.01)
n = 500 ; B_m = 0.3 ; B_x = −0.5 ; B_y = −0.5
CCPR (0.336)
UMPR (0.736)
UXPR (0.016)
AMPR (0.392)
AXPR (0.011)
UOV (0.012)
AOV (0.003)
uniform (0.01)
Pr(significance)
p−value threshold

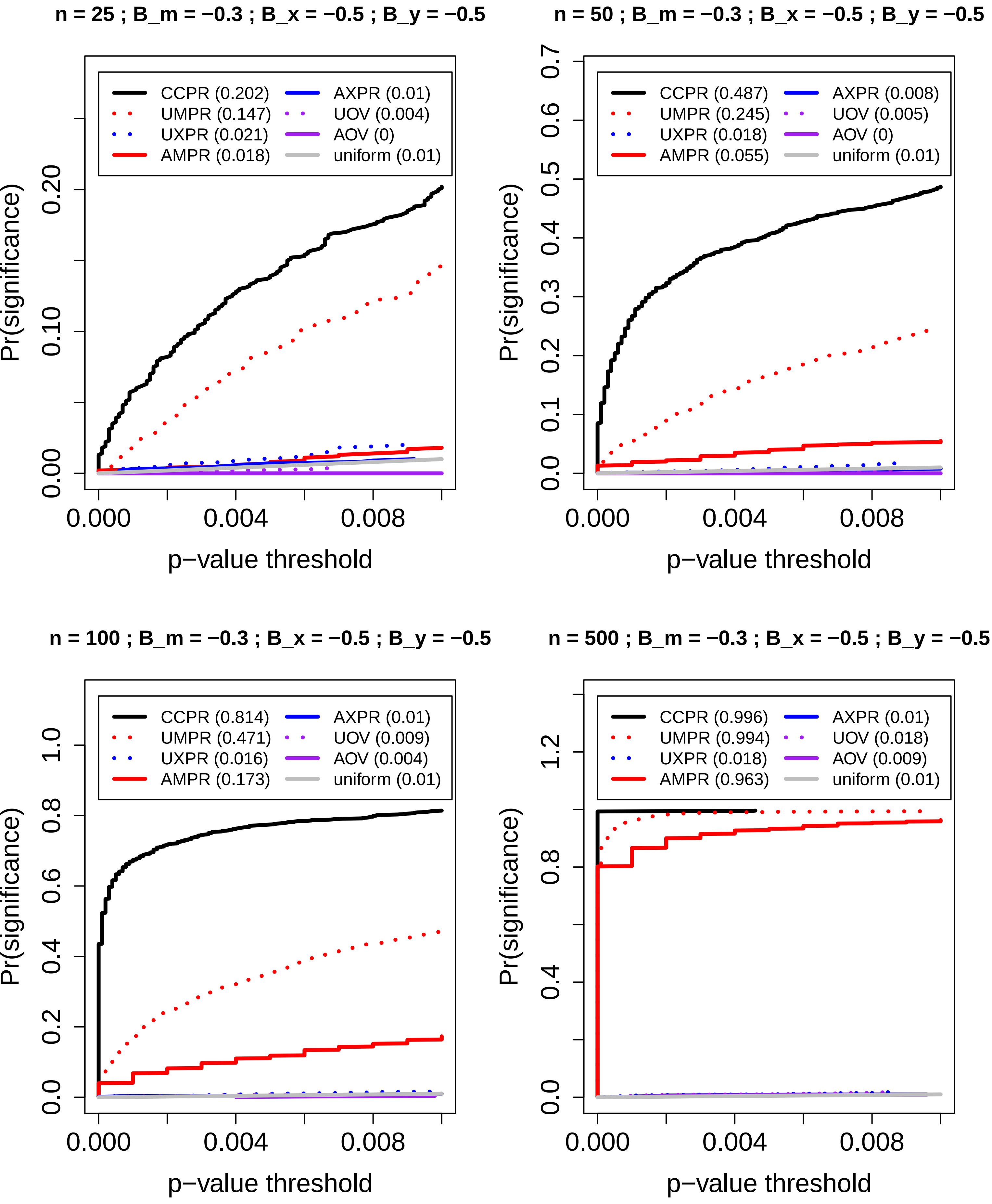

n = 25 ; B_m = −0.3 ; B_x = −0.5 ; B_y = −0.5
CCPR (0.202)
UMPR (0.147)
UXPR (0.021)
AMPR (0.018)
AXPR (0.01)
UOV (0.004)
AOV (0)
uniform (0.01)
Pr(significance)
0.00
0.10
0.20
0.000
0.004
0.008
p−value threshold
n = 50 ; B_m = −0.3 ; B_x = −0.5 ; B_y = −0.5
CCPR (0.487)
UMPR (0.245)
UXPR (0.018)
AMPR (0.055)
AXPR (0.008)
UOV (0.005)
AOV (0)
uniform (0.01)
Pr(significance)
0.0
0.1
0.2
0.3
0.4
0.5
0.6
0.7
0.000
0.004
0.008
p−value threshold
n = 100 ; B_m = −0.3 ; B_x = −0.5 ; B_y = −0.5
CCPR (0.814)
UMPR (0.471)
UXPR (0.016)
AMPR (0.173)
AXPR (0.01)
UOV (0.009)
AOV (0.004)
uniform (0.01)
Pr(significance)
0.0
0.2
0.4
0.6
0.8
1.0
0.000
0.004
0.008
p−value threshold
n = 500 ; B_m = −0.3 ; B_x = −0.5 ; B_y = −0.5
CCPR (0.996)
UMPR (0.994)
UXPR (0.018)
AMPR (0.963)
AXPR (0.01)
UOV (0.018)
AOV (0.009)
uniform (0.01)
Pr(significance)
0.0
0.4
0.8
1.2
0.000
0.004
0.008
p−value threshold

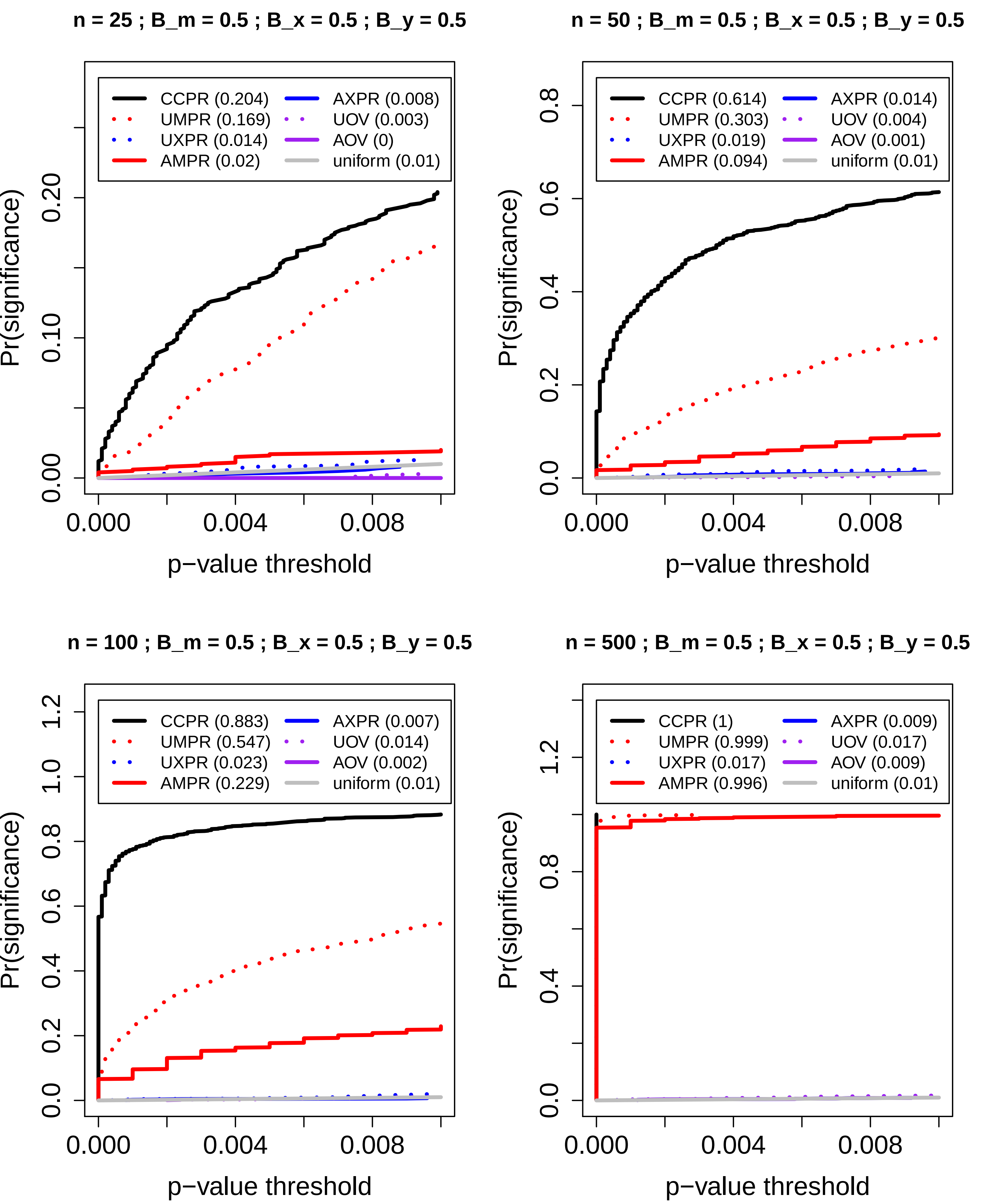

n = 25 ; B_m = 0.5 ; B_x = 0.5 ; B_y = 0.5
CCPR (0.204)
UMPR (0.169)
UXPR (0.014)
AMPR (0.02)
AXPR (0.008)
UOV (0.003)
AOV (0)
uniform (0.01)
n = 50 ; B_m = 0.5 ; B_x = 0.5 ; B_y = 0.5
CCPR (0.614)
UMPR (0.303)
UXPR (0.019)
AMPR (0.094)
AXPR (0.014)
UOV (0.004)
AOV (0.001)
uniform (0.01)
n = 100 ; B_m = 0.5 ; B_x = 0.5 ; B_y = 0.5
CCPR (0.883)
UMPR (0.547)
UXPR (0.023)
AMPR (0.229)
AXPR (0.007)
UOV (0.014)
AOV (0.002)
uniform (0.01)
n = 500 ; B_m = 0.5 ; B_x = 0.5 ; B_y = 0.5
CCPR (1)
UMPR (0.999)
UXPR (0.017)
AMPR (0.996)
AXPR (0.009)
UOV (0.017)
AOV (0.009)
uniform (0.01)
Pr(significance)
p−value threshold

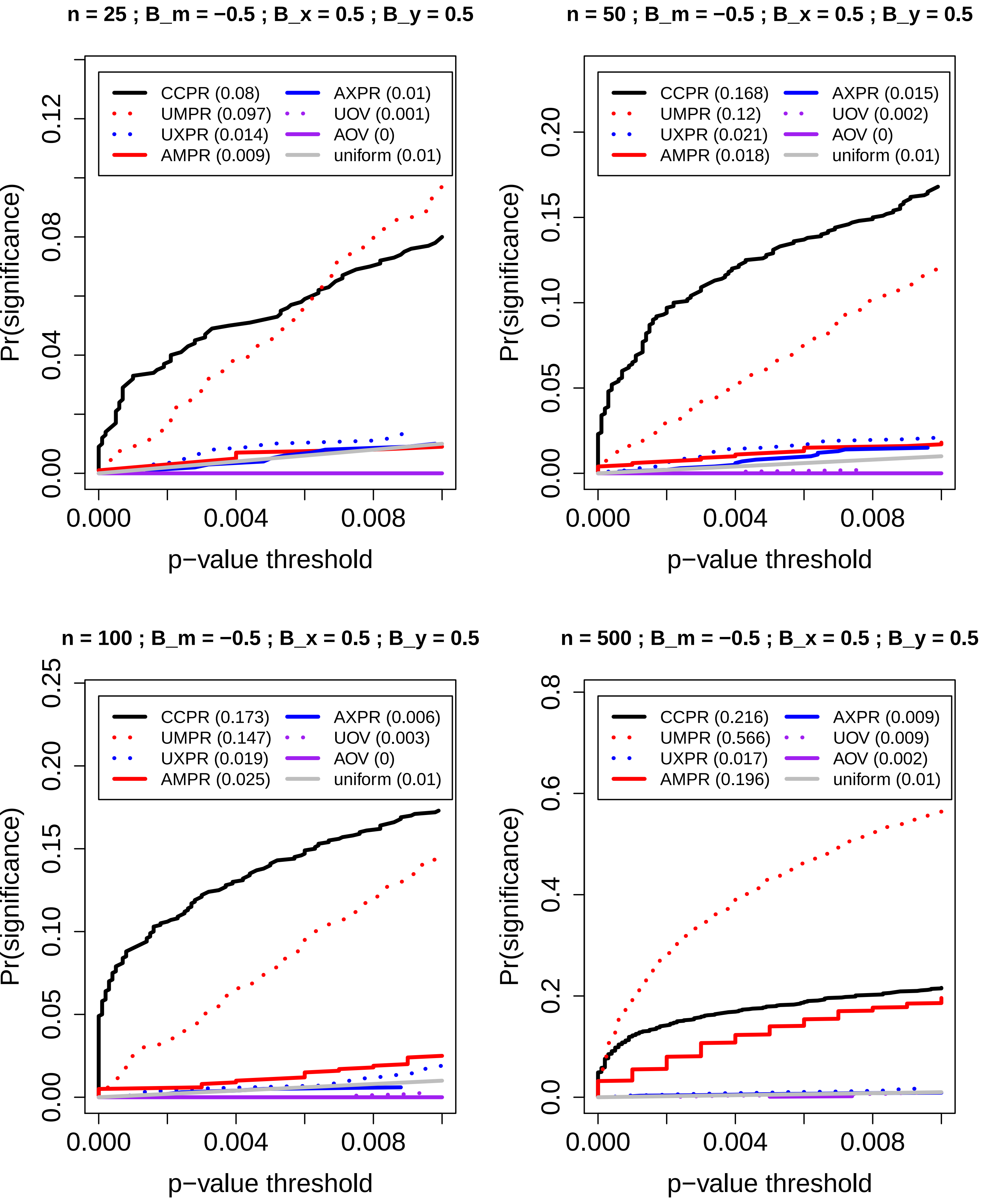
n = 25 ; B_m = −0.5 ; B_x = 0.5 ; B_y = 0.5
CCPR (0.08)
UMPR (0.097)
UXPR (0.014)
AMPR (0.009)
AXPR (0.01)
UOV (0.001)
AOV (0)
uniform (0.01)
Pr(significance)
0.00
0.04
0.08
0.12
0.000
0.004
0.008
p−value threshold
n = 50 ; B_m = −0.5 ; B_x = 0.5 ; B_y = 0.5
CCPR (0.168)
UMPR (0.12)
UXPR (0.021)
AMPR (0.018)
AXPR (0.015)
UOV (0.002)
AOV (0)
uniform (0.01)
Pr(significance)
0.00
0.05
0.10
0.15
0.20
0.000
0.004
0.008
p−value threshold
n = 100 ; B_m = −0.5 ; B_x = 0.5 ; B_y = 0.5
CCPR (0.173)
UMPR (0.147)
UXPR (0.019)
AMPR (0.025)
AXPR (0.006)
UOV (0.003)
AOV (0)
uniform (0.01)
Pr(significance)
0.00
0.05
0.10
0.15
0.20
0.25
0.000
0.004
0.008
p−value threshold
n = 500 ; B_m = −0.5 ; B_x = 0.5 ; B_y = 0.5
CCPR (0.216)
UMPR (0.566)
UXPR (0.017)
AMPR (0.196)
AXPR (0.009)
UOV (0.009)
AOV (0.002)
uniform (0.01)
Pr(significance)
0.0
0.2
0.4
0.6
0.8
0.000
0.004
0.008
p−value threshold

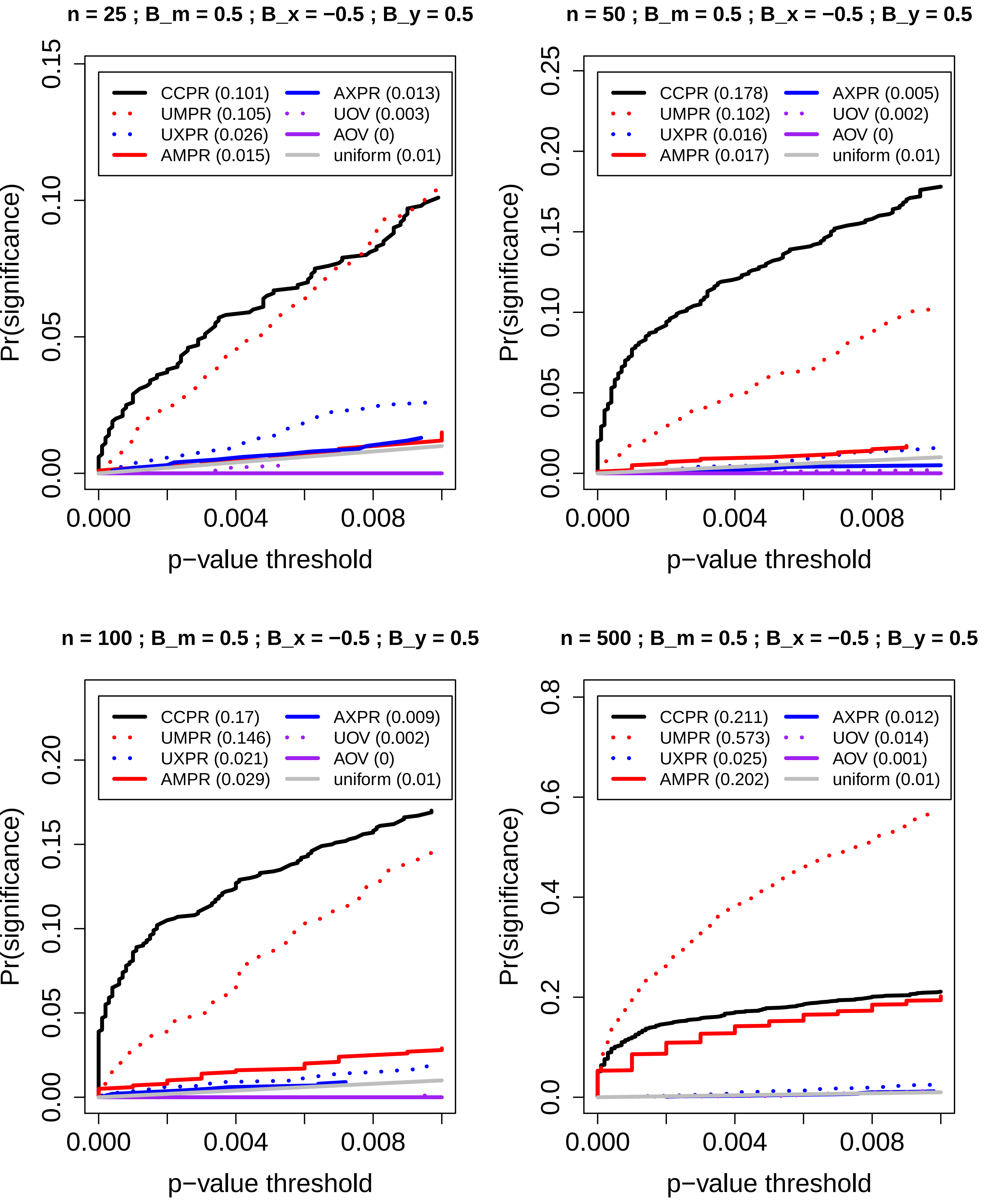

n = 25 ; B_m = 0.5 ; B_x = −0.5 ; B_y = 0.5
CCPR (0.101)
UMPR (0.105)
UXPR (0.026)
AMPR (0.015)
AXPR (0.013)
UOV (0.003)
AOV (0)
uniform (0.01)
n = 50 ; B_m = 0.5 ; B_x = −0.5 ; B_y = 0.5
CCPR (0.178)
UMPR (0.102)
UXPR (0.016)
AMPR (0.017)
AXPR (0.005)
UOV (0.002)
AOV (0)
uniform (0.01)
n = 100 ; B_m = 0.5 ; B_x = −0.5 ; B_y = 0.5
CCPR (0.17)
UMPR (0.146)
UXPR (0.021)
AMPR (0.029)
AXPR (0.009)
UOV (0.002)
AOV (0)
uniform (0.01)
n = 500 ; B_m = 0.5 ; B_x = −0.5 ; B_y = 0.5
CCPR (0.211)
UMPR (0.573)
UXPR (0.025)
AMPR (0.202)
AXPR (0.012)
UOV (0.014)
AOV (0.001)
uniform (0.01)
Pr(significance)
p−value threshold

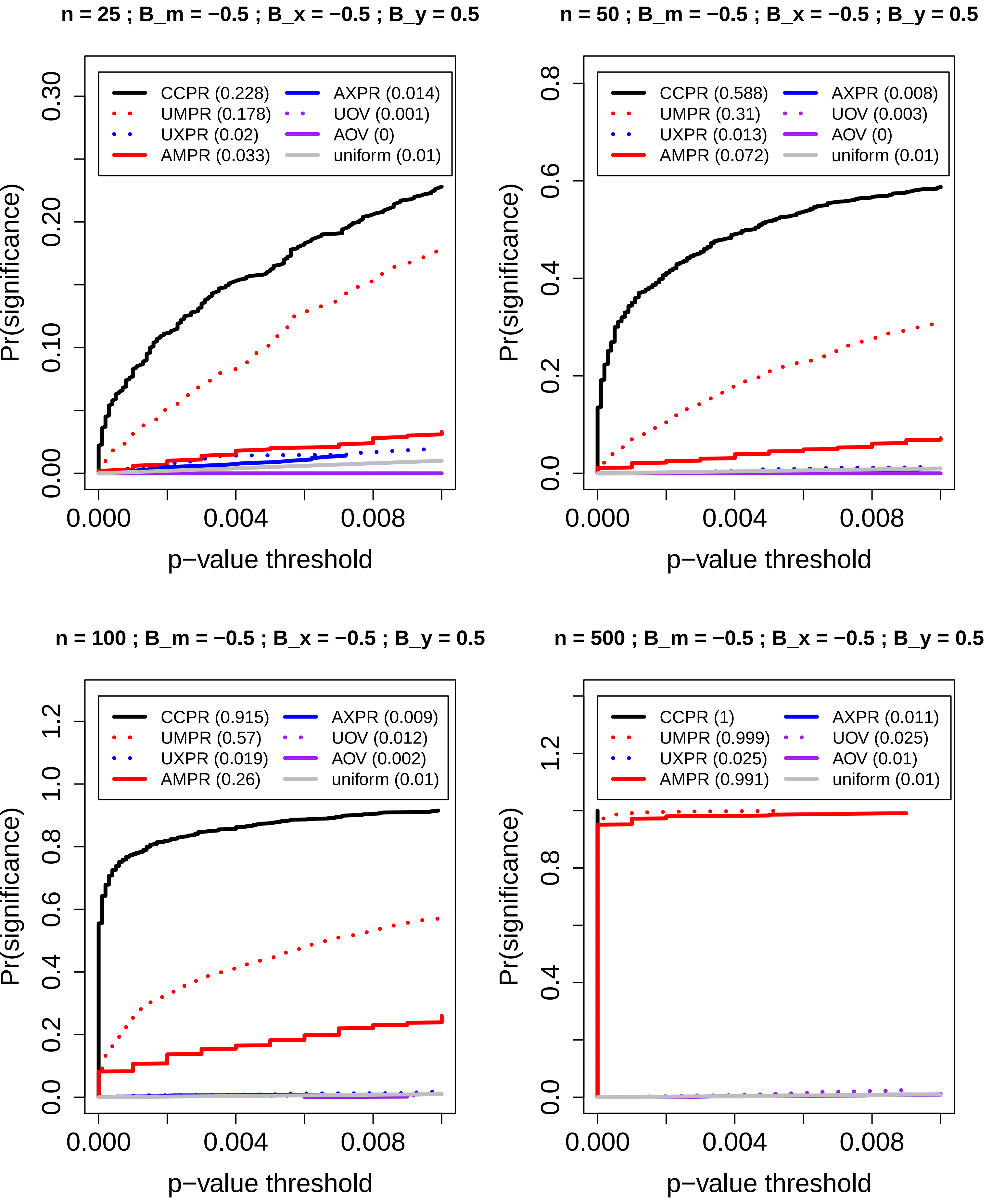

n = 25 ; B_m = −0.5 ; B_x = −0.5 ; B_y = 0.5
CCPR (0.228)
UMPR (0.178)
UXPR (0.02)
AMPR (0.033)
AXPR (0.014)
UOV (0.001)
AOV (0)
uniform (0.01)
Pr(significance)
p−value threshold
n = 50 ; B_m = −0.5 ; B_x = −0.5 ; B_y = 0.5
CCPR (0.588)
UMPR (0.31)
UXPR (0.013)
AMPR (0.072)
AXPR (0.008)
UOV (0.003)
AOV (0)
uniform (0.01)
Pr(significance)
p−value threshold
n = 100 ; B_m = −0.5 ; B_x = −0.5 ; B_y = 0.5
CCPR (0.915)
UMPR (0.57)
UXPR (0.019)
AMPR (0.26)
AXPR (0.009)
UOV (0.012)
AOV (0.002)
uniform (0.01)
Pr(significance)
p−value threshold
n = 500 ; B_m = −0.5 ; B_x = −0.5 ; B_y = 0.5
CCPR (1)
UMPR (0.999)
UXPR (0.025)
AMPR (0.991)
AXPR (0.011)
UOV (0.025)
AOV (0.01)
uniform (0.01)
Pr(significance)
p−value threshold

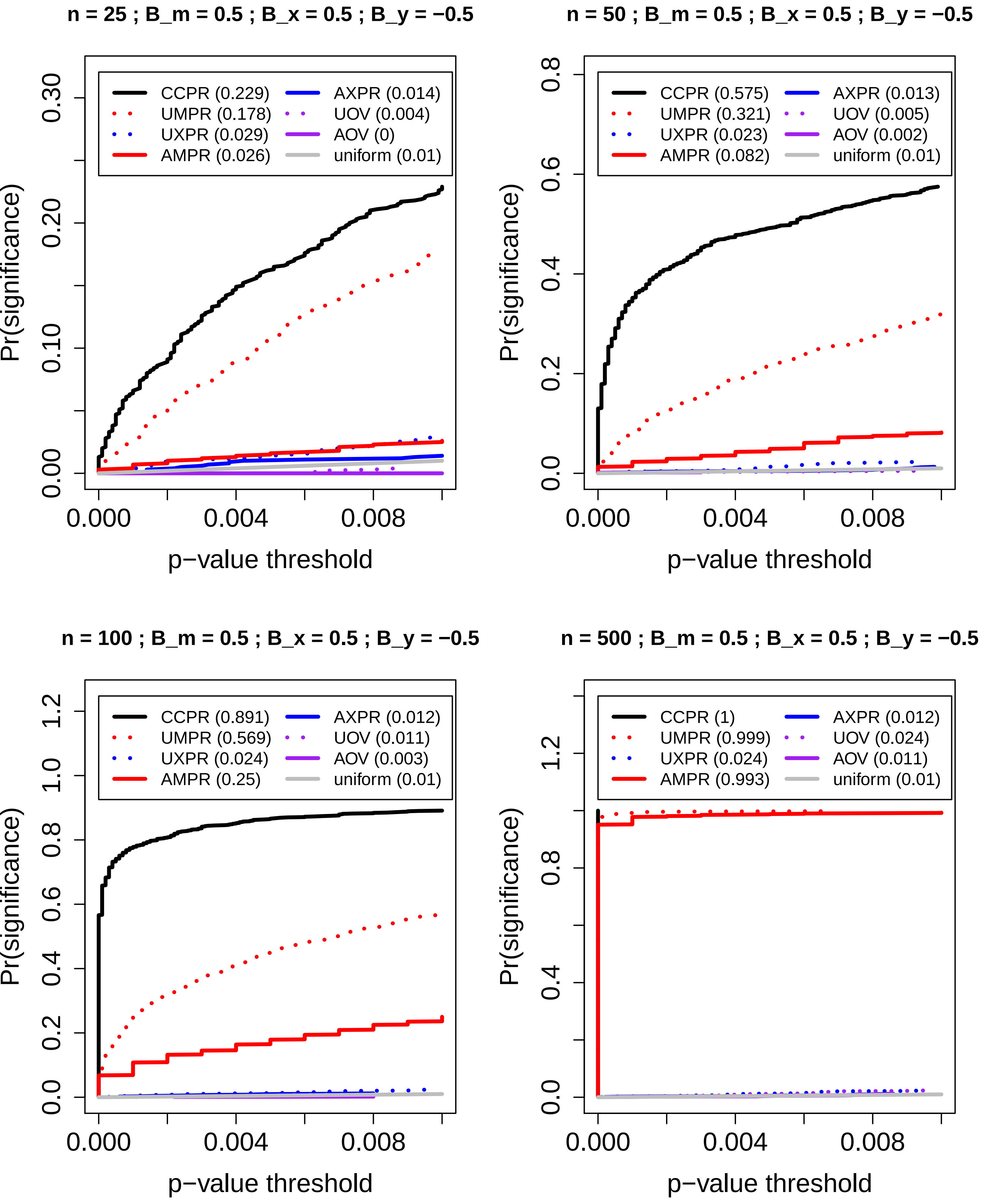

n = 25 ; B_m = 0.5 ; B_x = 0.5 ; B_y = −0.5
CCPR (0.229)
UMPR (0.178)
UXPR (0.029)
AMPR (0.026)
AXPR (0.014)
UOV (0.004)
AOV (0)
uniform (0.01)
Pr(significance)
0.00
0.10
0.20
0.30
0.000
0.004
0.008
p−value threshold
n = 50 ; B_m = 0.5 ; B_x = 0.5 ; B_y = −0.5
CCPR (0.575)
UMPR (0.321)
UXPR (0.023)
AMPR (0.082)
AXPR (0.013)
UOV (0.005)
AOV (0.002)
uniform (0.01)
Pr(significance)
0.0
0.2
0.4
0.6
0.8
0.000
0.004
0.008
p−value threshold
n = 100 ; B_m = 0.5 ; B_x = 0.5 ; B_y = −0.5
CCPR (0.891)
UMPR (0.569)
UXPR (0.024)
AMPR (0.25)
AXPR (0.012)
UOV (0.011)
AOV (0.003)
uniform (0.01)
Pr(significance)
0.0
0.2
0.4
0.6
0.8
1.0
1.2
0.000
0.004
0.008
p−value threshold
n = 500 ; B_m = 0.5 ; B_x = 0.5 ; B_y = −0.5
CCPR (1)
UMPR (0.999)
UXPR (0.024)
AMPR (0.993)
AXPR (0.012)
UOV (0.024)
AOV (0.011)
uniform (0.01)
Pr(significance)
0.0
0.4
0.8
1.2
0.000
0.004
0.008
p−value threshold

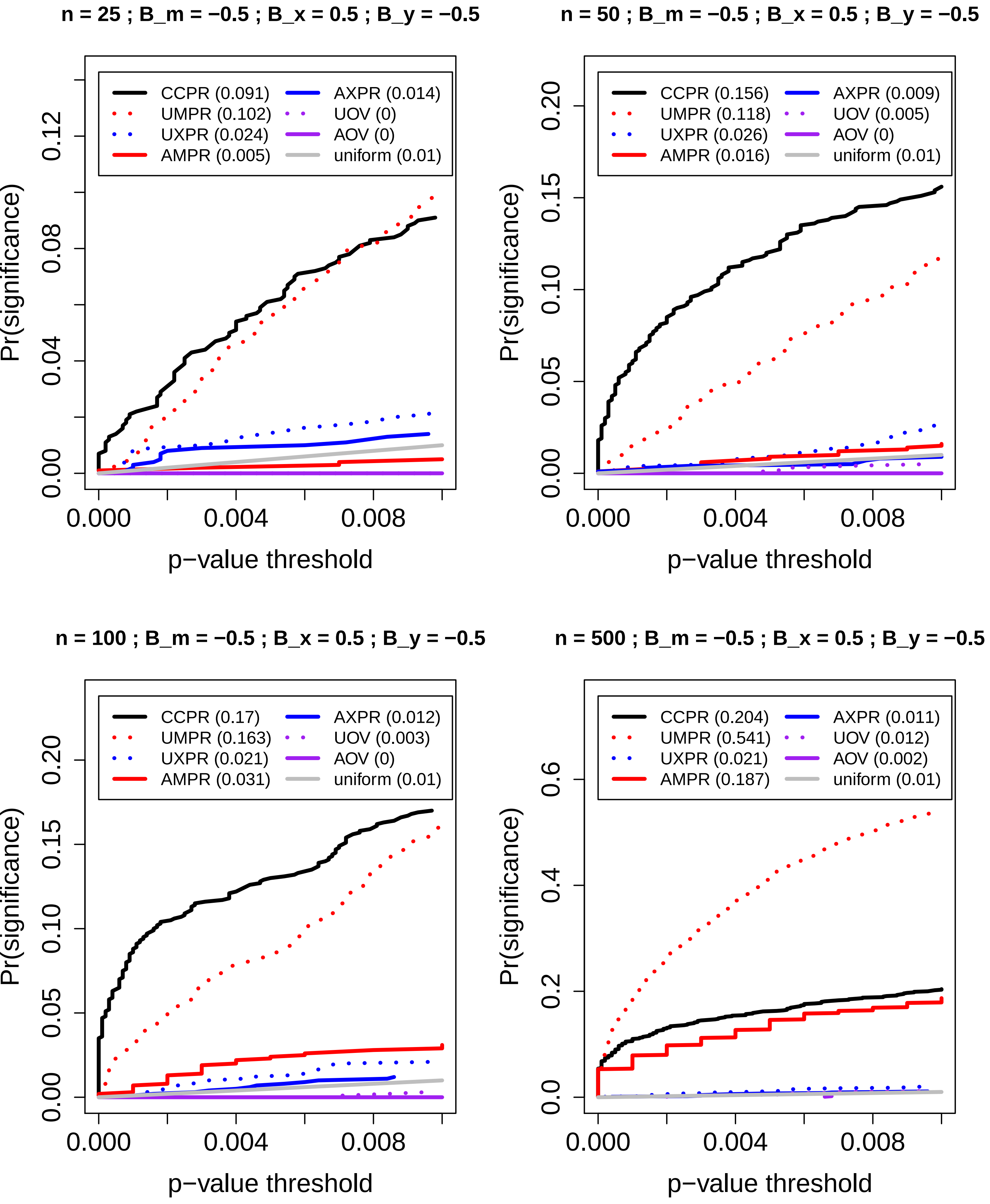

n = 25 ; B_m = −0.5 ; B_x = 0.5 ; B_y = −0.5
CCPR (0.091)
UMPR (0.102)
UXPR (0.024)
AMPR (0.005)
AXPR (0.014)
UOV (0)
AOV (0)
uniform (0.01)
n = 50 ; B_m = −0.5 ; B_x = 0.5 ; B_y = −0.5
CCPR (0.156)
UMPR (0.118)
UXPR (0.026)
AMPR (0.016)
AXPR (0.009)
UOV (0.005)
AOV (0)
uniform (0.01)
n = 100 ; B_m = −0.5 ; B_x = 0.5 ; B_y = −0.5
CCPR (0.17)
UMPR (0.163)
UXPR (0.021)
AMPR (0.031)
AXPR (0.012)
UOV (0.003)
AOV (0)
uniform (0.01)
n = 500 ; B_m = −0.5 ; B_x = 0.5 ; B_y = −0.5
CCPR (0.204)
UMPR (0.541)
UXPR (0.021)
AMPR (0.187)
AXPR (0.011)
UOV (0.012)
AOV (0.002)
uniform (0.01)
Pr(significance)
p−value threshold

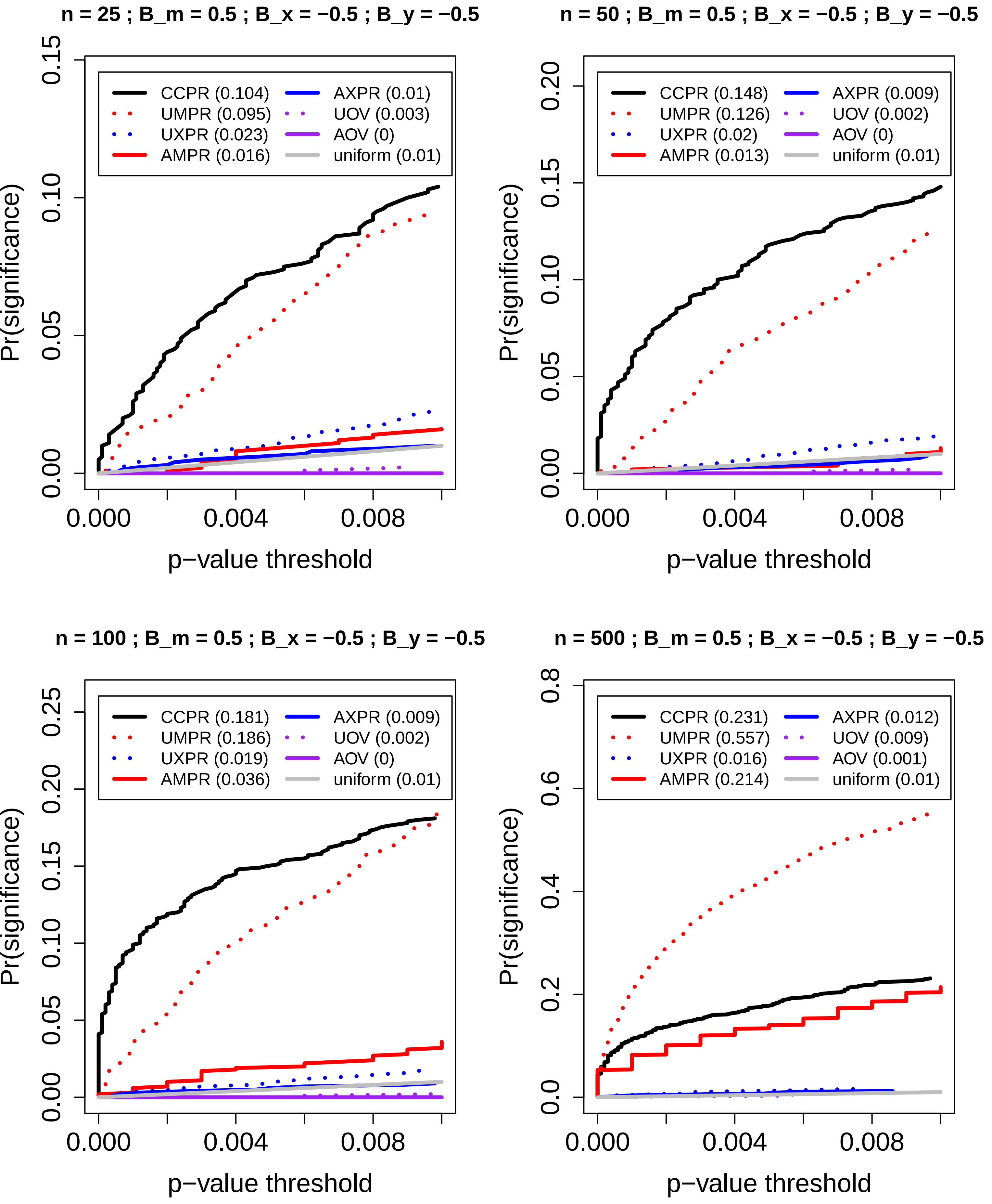
n = 25 ; B_m = 0.5 ; B_x = −0.5 ; B_y = −0.5
CCPR (0.104)
UMPR (0.095)
UXPR (0.023)
AMPR (0.016)
AXPR (0.01)
UOV (0.003)
AOV (0)
uniform (0.01)
n = 50 ; B_m = 0.5 ; B_x = −0.5 ; B_y = −0.5
CCPR (0.148)
UMPR (0.126)
UXPR (0.02)
AMPR (0.013)
AXPR (0.009)
UOV (0.002)
AOV (0)
uniform (0.01)
n = 100 ; B_m = 0.5 ; B_x = −0.5 ; B_y = −0.5
CCPR (0.181)
UMPR (0.186)
UXPR (0.019)
AMPR (0.036)
AXPR (0.009)
UOV (0.002)
AOV (0)
uniform (0.01)
n = 500 ; B_m = 0.5 ; B_x = −0.5 ; B_y = −0.5
CCPR (0.231)
UMPR (0.557)
UXPR (0.016)
AMPR (0.214)
AXPR (0.012)
UOV (0.009)
AOV (0.001)
uniform (0.01)
Pr(significance)
p−value threshold

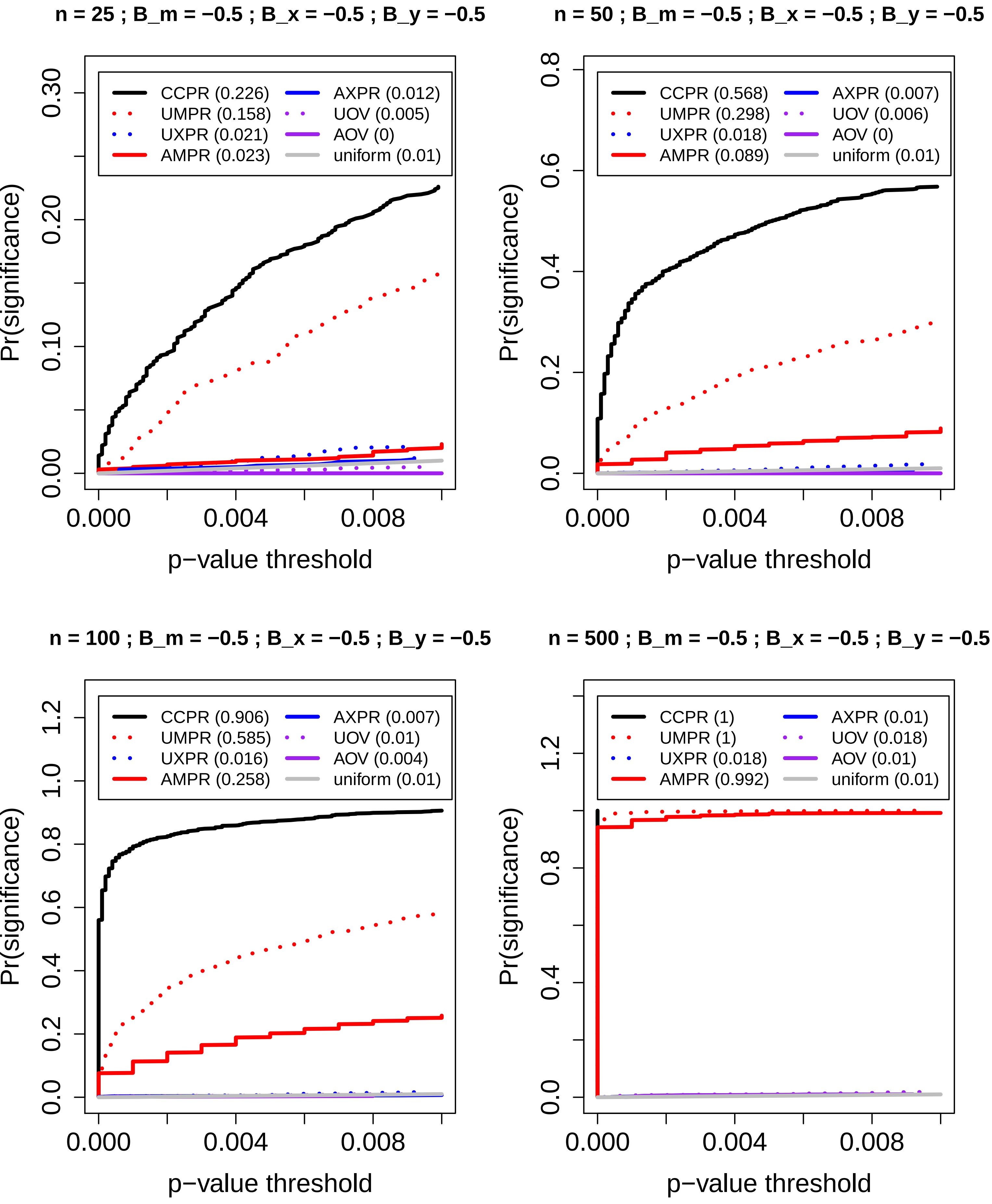

n = 25 ; B_m = −0.5 ; B_x = −0.5 ; B_y = −0.5
CCPR (0.226)
UMPR (0.158)
UXPR (0.021)
AMPR (0.023)
AXPR (0.012)
UOV (0.005)
AOV (0)
uniform (0.01)
Pr(significance)
p−value threshold
n = 50 ; B_m = −0.5 ; B_x = −0.5 ; B_y = −0.5
CCPR (0.568)
UMPR (0.298)
UXPR (0.018)
AMPR (0.089)
AXPR (0.007)
UOV (0.006)
AOV (0)
uniform (0.01)
Pr(significance)
p−value threshold
n = 100 ; B_m = −0.5 ; B_x = −0.5 ; B_y = −0.5
CCPR (0.906)
UMPR (0.585)
UXPR (0.016)
AMPR (0.258)
AXPR (0.007)
UOV (0.01)
AOV (0.004)
uniform (0.01)
Pr(significance)
p−value threshold
n = 500 ; B_m = −0.5 ; B_x = −0.5 ; B_y = −0.5
CCPR (1)
UMPR (1)
UXPR (0.018)
AMPR (0.992)
AXPR (0.01)
UOV (0.018)
AOV (0.01)
uniform (0.01)
Pr(significance)
p−value threshold
