# Supplementary material for: CC-PROMISE effectively integrates two forms of molecular data with multiple biologically related endpoints
Source: BMC Bioinformatics. 2016 Oct 6;17(Suppl 13):382. doi: 10.1186/s12859-016-1217-0 (PMC5073973; doi:10.1186/s12859-016-1217-0)
Supplement: Additional file 4 — This PDF file provides technical details regarding the CC-PROMISE analysis performed for the example application involving pediatric acute myeloid leukemia described in subsection “Acute myeloid leukemia example”. Details include description of the specific association statistics used for each molecular data set and each endpoint, the coefficients used in the analysis, and the number of permutations performed. (PDF 266 kb) [file 12859_2016_1217_MOESM4_ESM.pdf]

# CC-PROMISE integrates gene expression and DNA methylation with 3 related endpoints in pediatric AML.

## Patients

Patients of multi-center AML02 clinical trial were included in this study with approval of their respective institutional review boards.

## DNA Methylation data

DNA methylation levels of leukemia blast at diagnosis were measured by Infinium HumanMethylation450 BeadChip. The raw data were processed using minfi package from Bioconductor (http://www.bioconductor.org/packages/release/bioc/html/minfi.html). Data quality control was also performed according to minfi package. The M values were further adjusted for batch effect in analysis of variance framework. The batch adjusted M values are treated as the final methylation values.

## Gene expression data

Gene expression levels of leukemia blast at diagnosis were measured by affymetrix hg-u133a array. Data were processed by Affymetrix MicroArray Suite version 5 (*MAS5*). The signals were further log2 transformed. In total, there were 151 subjects with both expression and methylation data included in CC-PROMISE analysis.

## CC-PROMISE analysis

For each gene, canonical correlation was performed on the methylation probes and expression probes to obtain methylation score and expression score. The signs of the scores were selected so that the scores would be positively correlated.  For purposes of statistical analysis, this is equivalent to incorporating the sign of the canonical correlation statistic into the definition of the PROMISE statistic in equation (3) of the primary manuscript.

LC50 is a continuous variable. Spearman correlation statistic was used to measure association between LC50 and methylation scores $t_{M,LC50}$ or expression scores $t_{E,LC50}$.

MRD, minimal residual disease, was coded as 0 (no evidence of leukemia), 1 (0.1~1% of cells leukemic) and 2 (≥1% of cells are leukemic). Spearman correlation statistic was used to measure association between MRD and methylation scores $t_{M,MRD}$ or expression scores $t_{E,MRD}$.

EFS, event-free survival, is a time to event variable, the statistic of Jung, Owzar and George (2005) was used to measure association between EFS and methylation scores $t_{M,EFS}$ or expression scores $t_{E,EFS}$.

Lower LC50 (leukemia cells are more sensitive to cytarabine), lower MRD (less leukemia burden after one course of chemotherapy) and lower rate of relapse and disease progression (longer EFS) indicate greater sensitivity of leukemic cells to cytarabine. The corresponding PROMISE statistics were defined according to this treatment paradigm.

PROMISE test statistic for methylation score is defined as

$$T_M = -0.33t_{M,LC50} - 0.33t_{M,MRD} - 0.33t_{M,EFS}$$

PROMISE test statistic for expression score is defined as

$$T_E = -0.33t_{E,LC50} - 0.33t_{E,MRD} - 0.33t_{E,EFS}$$

CC-PROMISE test statistic is defined as

$$T_{CCPR} = 0.5T_M + 0.5T_E$$

The significance was determined by randomly assigning the subject labels 100,000 times.We chose to use a fixed number of permutations in this analysis to more accurately compare the performance of CC-PROMISE with other methods.

**Reference**

Jung SH, Owar K, George SL (2005) A multiple testing procedure to associate gene expression levels with survival. Stat. Med. 24:3077-3088.
